# mQC: A Heuristic Quality-Control Metric for High-Throughput Drug Combination Screening

**DOI:** 10.1038/srep37741

**Published:** 2016-11-24

**Authors:** Lu Chen, Kelli Wilson, Ian Goldlust, Bryan T. Mott, Richard Eastman, Mindy I. Davis, Xiaohu Zhang, Crystal McKnight, Carleen Klumpp-Thomas, Paul Shinn, John Simmons, Mike Gormally, Sam Michael, Craig J. Thomas, Marc Ferrer, Rajarshi Guha

**Affiliations:** 1 Division of Pre-Clinical Innovation, National Center for Advancing Translational Sciences (NCATS), Rockville, MD 20850, USA; 2 National Institute of Allergy and Infectious Diseases (NIAID), Rockville, MD 20852, USA; 3 Laboratory of Cancer Biology and Genetics, National Cancer Institute (NCI), Bethesda, MD 20892, USA

## Abstract

Quality control (QC) metrics are critical in high throughput screening (HTS) platforms to ensure reliability and confidence in assay data and downstream analyses. Most reported HTS QC metrics are designed for plate level or single well level analysis. With the advent of high throughput combination screening there is a need for QC metrics that quantify the quality of combination response matrices. We introduce a predictive, interpretable, matrix-level QC metric, mQC, based on a mix of data-derived and heuristic features. mQC accurately reproduces the expert assessment of combination response quality and correctly identifies unreliable response matrices that can lead to erroneous or misleading characterization of synergy. When combined with the plate-level QC metric, Z’, mQC provides a more appropriate determination of the quality of a drug combination screen. Retrospective analysis on a number of completed combination screens further shows that mQC is able to identify problematic screens whereas plate-level QC was not able to. In conclusion, our data indicates that mQC is a reliable QC filter that can be used to identify problematic drug combinations matrices and prevent further analysis on erroneously active combinations as well as for troubleshooting failed screens. The R source code of mQC is available at http://matrix.ncats.nih.gov/mQC.

The development of high throughput screening platforms has necessitated the development of quality control (QC) measures to determine assay performance at various levels. A key motivation for a QC measure is to ensure that data generated from a screen is reliable. In the absence of QC metrics, the downstream analysis of screening data can be misleading when applied to poor quality screening data. Furthermore, in long running screens, the use of QC metrics is crucial to capturing technical issues as they arise and subsequently, address them appropriately. Finally QC measures allow one to compare historical assay performance with that of current assays, and thus provide a metric against which assay and screening platform developments can be benchmarked.

Some QC measures are generally applicable to high throughput screening including the Z-factor (Z’), coefficient of variation (CV) and the signal to background (S/B). There has been much discussion on the utility of individual QC metrics focusing on what they can and cannot characterize^1^,^2^. For example, the S/B metric captures the extent of difference between sample wells and negative control, but does not quantify the variability^1^. As a result it is common to report multiple QC metrics for a given screening experiment.

QC measures can be classified into two groups. The first, and most common plate level controls characterize various aspects of the plate-level data. Examples include the Z’^3^ or SSMD (strictly standardized mean difference)^1^, both of which characterize the performance of the controls on an individual plate. Since controls are usually used for normalization of the sample area on the plate, poor control performance will lead to erroneous normalization and subsequently low quality assay readouts. This problem affects both single point screens as well as dose-response screens, though the latter can, sometimes, be more robust in the face of poor control performance. QC measures such as Z’ or SSMD operate on the well level and thus are not cognizant of signal artifacts that may be present over a region of the plate. Examples include edge effects^3^,^4^ (due to evaporation from wells on the edge of a plate) and dispense errors. Both these types of errors can manifest themselves in a signal that varies in a systematic fashion across rows or columns (or both) on a plate. These errors can be characterized by plotting the well signal from rows and columns separately or can be condensed into a single measure such as the coefficient of variation (CV)^5^. Finally, for large high throughput screens where samples are randomly laid out on a plate, it can be assumed that the signal should be close to random uniform and any outliers should be randomly distributed within the sample area. The presence of spatial artifacts can be characterized using a variety of spatial autocorrelation metrics including Geary’s C^6^ and Moran’s I^7^. Of course, this does not apply to screens with intra-plate titrations or screens where samples from different, focused libraries are insufficiently randomized. The use of spatial autocorrelation metrics also assumes that the majority of samples are inactive (or rather, have similar activity). For focused libraries, and depending on the assay system, this condition may not be satisfied.

The second class of QC parameters applies to sample level controls and report variability on biological responses in the assay throughout a screen. These controls are typically not independent of plate level controls since the sample data is usually obtained after normalization (and possibly correction) of the plate level data. For small molecule high throughput screens, the Minimum Significant Ratio (MSR)^8^ is probably the most widely used and characterizes the assay variability in terms of the variability of sample (or control compound) potencies.

Recently, Mathews-Griner *et al*.^9^ described the development of a high-throughput platform to perform pair-wise drug combination screening, cHTS. The initial version of the platform was used to screen a few hundred drug combinations. However, the platform has expanded to thousands and even tens of thousands of combinations^10^. Since the platform is based on traditional plate-reader technology, traditional plate-level QC metrics, especially those based on control performance, can be computed to provide an overall determination of the assay performance.

However, unlike single point small molecule screens or siRNA screens, where the normalized well readout is used for downstream analysis, a drug combination screen is followed by an analysis of the combination response matrix, which is deconvoluted from the plate layout, to characterize synergy, antagonism or additivity. Since the current platform lays out individual combinations as explicit *N* × *N* matrices on the plate, screening errors can result in noisy or non-random combination response matrices. Even if the response matrices are randomized across one or more plates, screening errors can result in artifacts in an individual combination response matrix, when it is deconvoluted. If not otherwise flagged, these response matrices can result in erroneous estimates of synergy. There are many ways of scoring pharmacological synergism^11^,^12^, but all of them are sensitive to noise. As an example, the Combination Index (CI)^13^ depends on accurate evaluation of the IC_50_ (half maximum inhibitory concentration) of the single agents in a combination response matrix. Thus, response matrices with poor quality or non-existent single agent dose responses should be characterized as being of lower quality and flagged as such.

This work presents a data driven method to numerically characterize the quality of an individual response matrix in a high throughput combination screening experiment. The measure is termed the matrix QC (mQC) and allows one to rank response matrices from high quality to low quality. While derived from large screens, it can be applied to small-scale combination screens as well. The current method is a more rigorous extension of the QC metric described in Mott *et al*.^10^. In particular we based the development of the QC metric on a crowd-sourced assessment of response matrix quality. The use of crowd-sourced assessments of “quality” and other abstract descriptors has precedents^14^,^15^,^16^,^17^. For example, Lajiness *et al*.^18^ analyzed the consistency of the opinions of medicinal chemists when reviewing compound sets. Similarly, Oprea *et al*.^19^ examined the quality of chemical probes from the NIH Molecular Libraries Initiative, by aggregating expert assessments.

First we describe the need for a matrix-level QC and the data used to derive the model underlying the mQC. An important component of this step was constructing a set of response matrices whose quality was manually assessed by a panel of 9 experts. We describe the construction of this training set and discuss aspects of this crowd-sourced approach to quality assessment. Next we propose the prospective use and describe the retrospective application of mQC based on an analysis of a set of 612 combination screening campaigns comprising of a total of 127,119 response matrices. In particular, we compare mQC with Z’ using a subset (119,287 response matrices) with available plate-level data and propose QC guidelines for cHTS. Finally we discuss the limitations of the mQC metric and various factors that influence its reliability and relevance.

## Results

### Z’ fails to correlate with expert opinions of matrix-level quality

**Figure 1:**
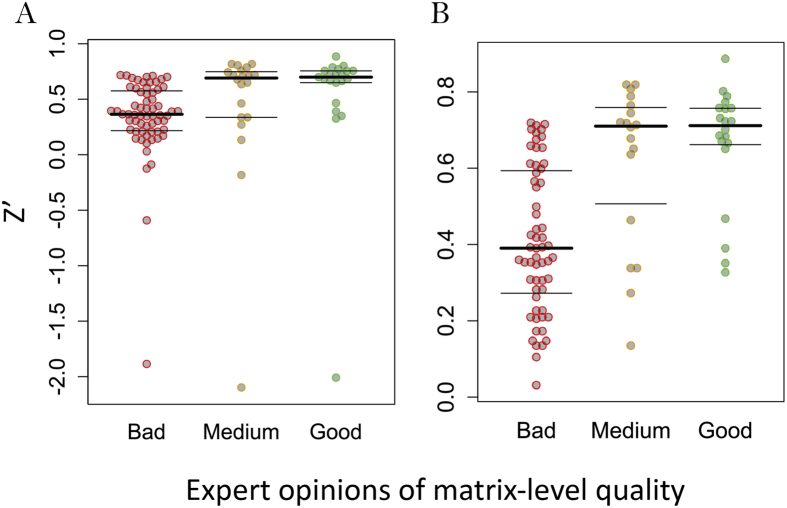
Comparison of expert opinions of matrix-level quality and plate-level QC (Z’). (**A**) A comparison from all 133 response matrices in the survey. (**B**) A comparison by removing bad-quality plates with Z’ < 0.

To justify the development of a novel QC metric for combination screening, we compared the plate-level QC, Z’, and the expert opinions of matrix-level quality polled from 9 experienced scientists at NCATS (Fig. 1A, Supplementary Dataset S1 and Supplementary Fig. S6). As expected, Z’ is insufficient to distinguish the expert opinions of matrix-level qualities using one-way ANOVA (analysis of variance), p-value = 0.26. If we remove the extremely poor-quality plates (e.g., Z’ < 0), the ANOVA p-value drops to 2.75 × 10^−7^ (Fig. 1B). However, Z’ fails to conclusively differentiate between good and medium classes (p-value = 1.0), and there are still 50% good (n = 10) and 61% medium response matrices (n = 11) that overlap in Z’ with the bad ones, although pairwise t-tests show significant difference between good and bad (p = 3.3 × 10^−6^), or medium and bad (p = 2.8 × 10^−4^). This highlights the need for a more sensitive and robust assessment of the quality of a combination screen.

### The mQC metric accurately models the crowdsourced opinions

**Figure 2:**
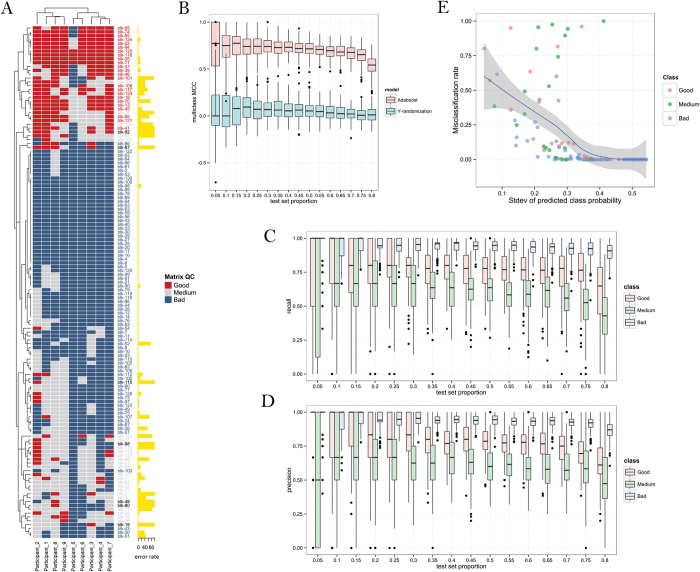
Performance of mQC. (**A**) Heatmap of survey results and average error rate for each response matrix. (**B**) The multiclass MCC at different test set proportion using the original dataset (red) and Y-randomized dataset (blue). (**C**,**D**) The recall and precision of each matrix-level QC label at different test set proportion. (**E**) The confidence of mQC prediction as a function of the standard deviation of the predicted probabilities across mQC labels.

**Figure 3:**
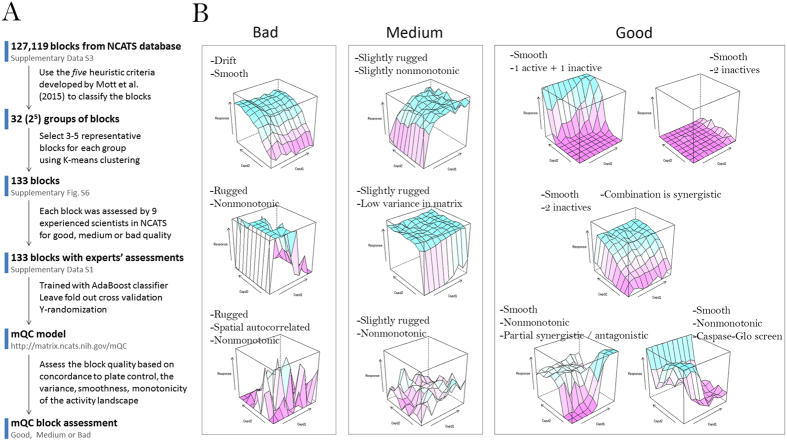
(**A**) Schematic workflow summarizing the steps involved in selecting the 133 combinations for the training set used to construct the mQC model. (**B**) Examples of response matrices and their mQC. Here we show 11 activity landscape in 3D and their corresponding “Bad”, “Medium” or “Good” classification predicted by mQC. Each activity landscape is transformed from the response matrix (see Methods for details) and annotated with respective surface features. We expect zero response on DMSO treatment (negative control), and a maximum 100% response (adjusted by the positive control) in CellTiter-Glo screens or −100% response (adjusted by the positive control) in Caspase-Glo screens.

The mQC is an Adaboost ensemble decision tree model, trained using a crowdsourcing effort consisting 9 experts in which each expert individually labeled a set of 133 response matrices as ‘Good’, ‘Medium’ or ‘Low’ quality (Fig. 2A). Figure 3A illustrates how these 133 blocks were selected to construct the training set. mQC model was trained based on a subset of 126 response matrices that reached the consensus opinion between the raters. Given a response matrix, mQC evaluates 7 response matrix-derived features characterizing the concordance to plate control, and the variance, smoothness, monotonicity of the activity landscape (see Table 1 and Methods for details), and predicts a QC label (i.e., good, medium or bad) and an associated confidence score. To assess the predictive power of mQC, we performed training-testing validation protocols as described in the Methods. Figure 2B revealed that the multiclass-MCC (Matthews Correlation Coefficient), which is a balanced measure of classification accuracy regardless of the class composition, is consistently high (~0.75) using 5–50% of 126 response matrices as the test set. The multiclass-MCC remained greater than 0.5 using 55–80% of 126 response matrices as the test set. This indicates that mQC does not overfit the crowdsourced responses and can be generalized to unseen matrix responses. In comparison, Y-randomization significantly compromised the multiclass-MCC at all test set proportions, indicating that the mQC model was not obtained due to chance correlations (Fig. 2B).

Figure 2C,D summarizes the recall and precision performance for the different quality classes. mQC exhibited excellent accuracy in identifying “Bad” and “Good” quality matrices, as the recall and precision were both greater than 0.75 for these two classes using 5–70% as the test set. Mediocre performance (~0.62) was observed for “Medium” quality matrices. This is not surprising because by definition the boundary between “Good” and “Medium” or “Bad” and “Medium” is somewhat ambiguous, and individuals disagreed significantly for “Medium” quality matrices (Fig. 2A). Furthermore, we observed that a majority of the prediction error was attributable to the matrices in which polled results had significant disagreements, even when we took the majority votes for the classifier (Fig. 2A). To take the predictive accuracy into account, we fit a probabilistic confidence score using the standard deviation of the Adaboost-predicted class probabilities, as shown in Fig. 2E. As expected, the higher the variation in the class probability, the lower the prediction error. The classification error, on average, remains below 25% when mQC makes a decisive good/medium/bad prediction (e.g., Stdev (prob_good_, prob_medium_, prob_bad_) > 0.25). This confidence score will be useful when performing large-scale statistical analysis.

Figure 3B depicts examples of response matrices with different mQC features. mQC is built upon an ensemble tree using these features, and Table 1 summarizes the feature importance for the mQC model. It highlights that dmso.v, smoothness.p, moran.p are the most critical features for matrix-level quality. This prediction is consistent with the intuition that the presence of anomalous control values, lack of smoothness, and random/negative spatial autocorrelation within the combination response submatrix suggests major technical issues than low activity variance and non-monotonic dose responses. It is also reasonable not to overestimate the importance of sa.min, sa.max, sa.matrix and mono.v, because either low activity variance or non-monotonic dose response can be a result of real biology other than technical failure. As demonstrated in Fig. 3B, mQC did not flag the non-prototypical biological responses (e.g., bell-shaped dose response, non-monotonic activity landscape due to synergy/antagonism) or inactive combination responses, as long as the activity landscape is smooth and absence of drift.

### Using mQC prospectively: mQC suggests the reliability of response matrices

**Figure 4:**
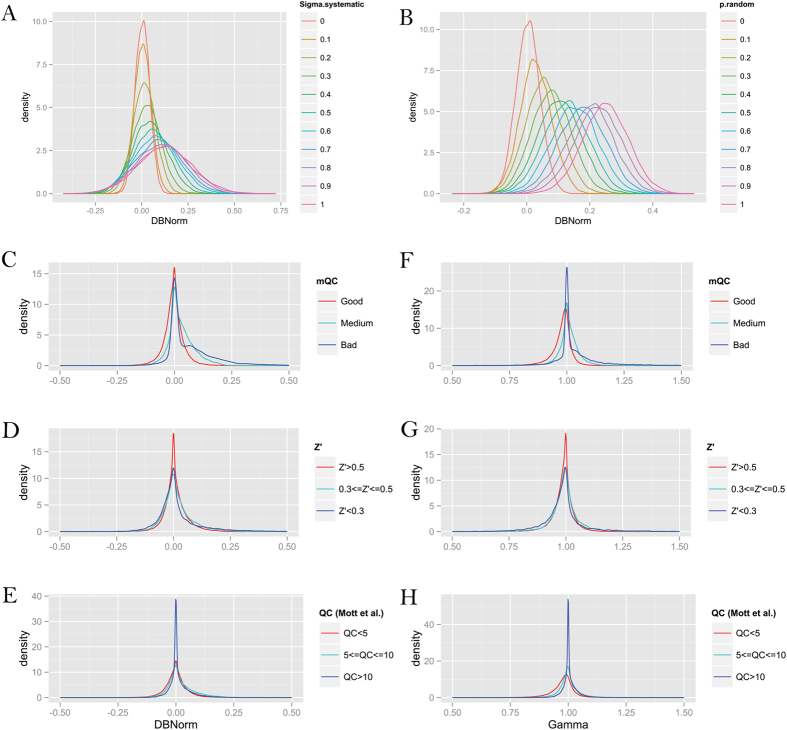
QC metric and synergy distribution. (**A**) Distribution of normalized delta-Bliss (DBNorm) with different levels of systematic error 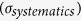. (**B**) Distribution of DBNorm with different fractions of random error (p.random) introduced in the model. (**C**–**E**) Distribution of DBNorm based on mQC, Z’ or QC Mott *et al*. (**F**–**H**) Distribution of gamma based on mQC, Z’ or QC Mott *et al*.

In this section we further explored whether mQC, which is trained using subjective crowdsourced opinions, prospectively correlated with the reliability of a matrix screen and enriched for real signals of synergism/additivity/antagonism signals. Assume we have a population of response matrices from CellTiter-Glo assays, where the last row/column is the single agent dose response, and 100% exposure represents no cell killing and 0% represents full cell killing. The synergism/additivity/antagonism associated with a response matrix 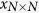 can be computed based on the Bliss independence model^20^. Using a deviation from the Bliss model (equation 8), we find that the normalized delta-Bliss (DBNorm) keeps the normal distribution when the systematic error 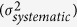 is small (Fig. 4A). However, the distribution will be skewed to positive if 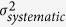 becomes large (Fig. 4A). If unpredictable random error is introduced, the DBNorm distribution will be centered at 0.25 regardless of the original DBNorm distribution (Fig. 4B). Hence, our simulation has showed that the positive skewness of the DBNorm distribution correlates with the level of random error and systematic error: the more systematic or random error introduced in the screening, the synergy distribution will be skewed more to the antagonistic area.

Then we analyzed 127,119 response matrices collected from NCATS database, and based on the mQC model we observed that DBNorm for the “Good” matrices displayed a slightly left-skewed distribution while “Medium” and “Bad” matrices displayed more positive-skewed distributions (Fig. 4C). The negative-skewed distribution of “Good” matrices is primarily due to the bias toward synergistic combinations during assay planning and validation. The “Bad” distribution, however, exhibited a second peak around DBNorm = 0.12, indicating a subpopulation of response matrices consisting of large systematic error or random error. In comparison, the DBNorm distributions using Z’ or another matrix-level QC (Mott *et al*.^10^) overlap significantly and show no skewness for bad matrices (e.g., Z’ < 0.3 or QC > 10) (Fig. 4D,E). A similar trend was also found for another synergy metric, γ, which is based on Gaddums non-interaction model^21^ (Fig. 4F–H). Taken together these data implied that mQC, rather than Z’ or QC (Mott *et al*.), models better the noise level in the response matrix and as a consequence, it is a more reliable indicator of the confidence of synergy or antagonism discovery.

**Figure 5:**
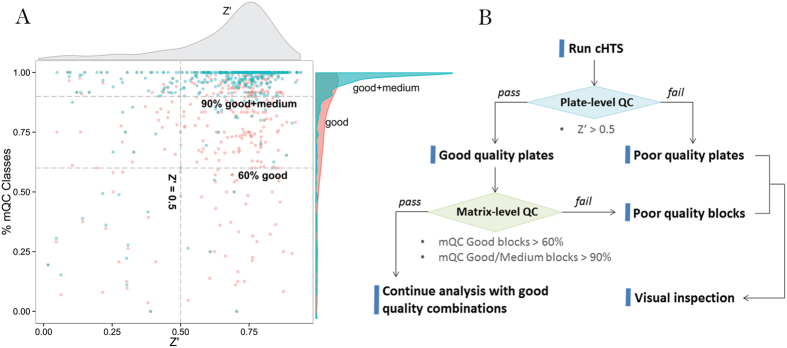
Proposed QC guideline for drug combination screening. (**A**) Each combination screening is represented by two independent points: a red point (Z’ as X-axis value and percentage of “Good” matrices as Y-axis), and a green point (Z’ as X-axis and percentage of “Good” plus “Medium” quality matrices as Y-axis). The distribution associated with Z’, Good%, Good + Medium% are beside the scatter plot. The dashed lines indicate the best practice cutoff for Z’ and mQC levels given a screening. (**B**) The best practice workflow for quality control of a cHTS campaign.

This large-scale analysis was in line with our initial hypothesis that Z’ alone is insufficient to indicate the overall quality of a response matrix. In addition, from comparing Z’ and mQC using a subset which has trackable plate-level data (totally 119,287 blocks available in Supplementary Dataset S3), we observed weak correlation between Z’ and mQC using Spearman correlation (ρ^good^ = 0.23, ρ^medium^ = −0.008, ρ^bad^ = −0.38 when Z’ is aggregated by screen, Fig. 5A). Z’ and SSMD also have poor correlation with mQC if we analyze the QC breakdown by plate (Supplementary Fig. S2). Noticing the fact that Z’ or SSMD may not hold if the controls are placed on one side in the presence of dramatic plate effect, we also calculated Z’ (sample) and SSMD (sample) using the block DMSO controls and original positive controls. However, we are still unable to find a reasonable correlation between plate-level QC metrics (Z’ (sample) or SSMD (sample)) and mQC (Supplementary Fig. S3), although Z’ (sample) and SSMD (sample) only achieves a mediocre correlation with Z’ (plate) or SSMD (plate) (Supplementary Fig. S4). Therefore, it is reasonable to define a combined criterion as the basis of a QC guideline for cHTS. The conventional criterion for a good HTS is Z’ > 0.5, and here we found that ~85% screenings met this QC requirement. Based on this 85th quantile that defines an excellent HTS assay based on plate-level quality, the corresponding matrix-level mQC criterion should be “screen with >60% Good response matrices and >90% Good or Medium response matrices” (the horizontal dashed lines in Fig. 5A). Herein we suggest that the quality of a cHTS campaign be judged by both plate-level and matrix-level QC metrics: (1) Z’ > 0.5 and (2) >60% “Good” response matrices and (3) >90% “Good” or “Medium” response matrices (Fig. 5B). If only plate-level QC is satisfied, it suggests that major matrix-level issues are involved, such as low cell viability, wrong time points, unstable readout, problems in chemical selection/handling/concentration, etc. Otherwise, it suggests a failed control or biased layout as a majority of response matrices satisfy the matrix-level QC criteria.

### Using mQC retrospectively: mQC identifies source of variability

In this section, we specifically examine the potential use of mQC to identify sources of variability that are specific to combination screening in matrix format or may not be identified by the conventional plate-level QC metrics. We will elaborate on 11 cases to show how mQC further enhance the quality assessment of cHTS (case summary can be found in Table 2 and Supplementary Fig. S5).

#### Readout

**Figure 6:**
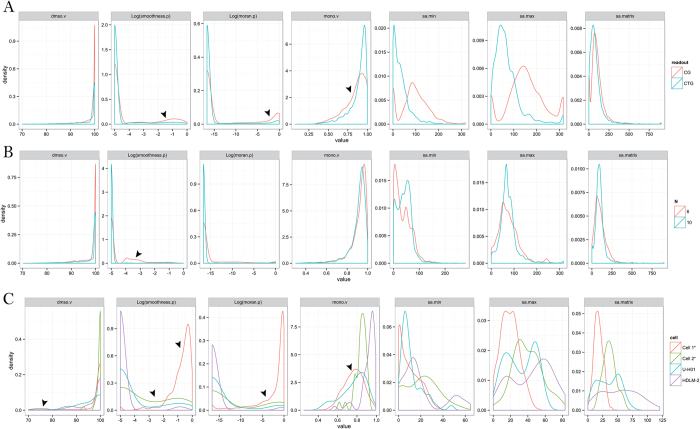
Comparison of readout, size of matrix and cell with respect to 7 feature distributions. (**A**) Readout (Caspase-Glo(CG) vs. CellTiter-Glo(CTG)). (**B**) Size of matrix (6 × 6 vs. 10 × 10). (**C**) Cell. * = contaminated cell line. The arrows indicate the major difference between groups which significantly affects the mQC assessment.

Very often Z’ is determined based on the effect of a positive control on the assay. However, in some cases, the positive control is not available or cannot produce the maximum change in signal that the assay can measure. For example, Promega Caspase-Glo 3/7 (CG) used in many of our combination screens measures the induction of apoptosis as an increase in luminescence signal. Bortezomib is a proteasome inhibitor which is a potent cytotoxic compound for most of the cells tested and it is used as a positive control in the cell proliferation assay. However, Bortezomib does not produce cytotoxic effects by induction of apoptosis in all cells, and therefore, for its use as a positive control for Caspase-Glo assay readout is not appropriate for some cell lines. For example, we are able to confirm several synergistic combinations against L1236 cell line in a CG screen (assay ID 3785 in Table 2)^22^, although Bortezomib failed to induce significant Caspase activity compare with DMSO (Supplementary Fig. S5A). Besides, mQC offers an alternative QC metric to compare different assay readouts independent of the availability of the positive control. When comparing Promega CellTiter-Glo (CTG) and CG, we observed that the quality of CTG is significantly better than CG from 3,084 paired comparisons of response matrices, in which mQC of CTG was found better in 949 cases, worse in 191 cases and equal in 1944 cases (p-value = 1.18 × 10^−63^). Compared with CTG, CG has a significantly higher occurrence of rugged activity pattern (smoothness.p > 10^−4^), random spatial autocorrelation (moran.p < 10^−7^) and non-monotonic dose response (mono.v < 0.7) (Fig. 6A). This result indicates that the assay readouts which measure conditional enzymatic activity (e.g., apoptosis via caspase activity) can be more challenging to optimize and less stable than simple readouts that measure the baseline metabolites (e.g., cell viability via ATP amount) for cHTS.

#### Size of matrix

Ideally, the dimension of a matrix in a combination screening experiment (i.e., the number of doses of the single agents) should not affect the matrix-level QC. That is, a cHTS using the same cell line, chemical library and readout should have similar mQC, irrespective of matrix size. However, we still observed that 10 × 10 response matrices had statistically better mQC than 6 × 6 screenings from 1937 comparisons (p = 2.9 × 10^−7^), in which mQC of 10 × 10 format was better in 309 cases, worse in 129 cases and equal in 1499 cases. Compared with matrices in 10 × 10 format, 6 × 6 format has a higher occurrence of rugged activity pattern (smoothness.p > 10^−4^) (Fig. 6B). However, we note that the screening workflow employed at NCATS tends to select 6 × 6 combinations that exhibit high quality and robust response matrices for follow-up in a 10 × 10 format, which biases the observed results.

#### Cell quality

Cell quality is another major source of variation in HTS. Z’ alone may be unable to flag the poor cell quality for reasons such as low cell viability or contamination in a cHTS because these factors might not have a large effect on the assay window determined using plate wells with negative and positive controls, but may impact the combination responses because of effects in the sample field. For example, mQC, but not Z’, successfully identified the only two documented cHTS campaigns where the cell lines were found contaminated (assay ID 5021 and 6028 in Table 2). In comparison, the screenings using cell in good condition usually yield a majority of “Good” response matrices. For example, we observed 96.8% “Good” and 100% “Good” response matrices from two public datasets (assay ID 142 and 447 in Table 2)^9^. In addition, mQC flagged a cHTS using Hodgkin’s lymphoma cell line U-H01 (Z’ = 0.70 ± 0.03) (assay ID 2852 in Table 2), whose response matrices obtained significantly worse mQC assessments than those from a parallel screen using HDLM-2 (assay ID 2850 in Table 2). This is due to the fact that on the day of plating, the viability of U-H01 was 60%, whereas HDLM-2 was 100% according to the lab notes. According to usual practice, however, these screens are treated as excellent screens with respect to their high Z’. Further analysis of feature distribution showed that cell contamination or low viability resulted in significant increase of abnormal control signal, rugged and autocorrelated matrices and non-monotonic dose response (Fig. 6C). mQC once again demonstrated the ability to pinpoint the unreliable screenings due to cell contamination, poor viability, etc., which cannot be reliably identified by the conventional Z’ metric.

#### Drift

**Figure 7:**
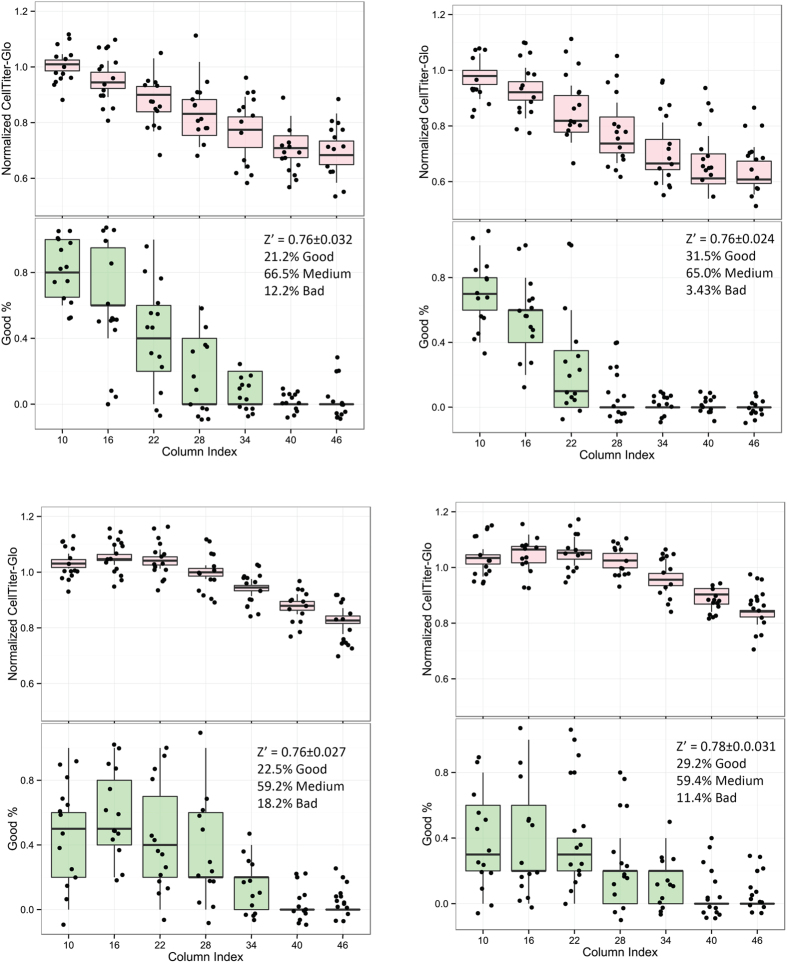
Drift effect identified by mQC. Each plot represents an independent screen consisting of 14 plates. The point in the upper part of plot represents the median response of matrix-level negative control (DMSO) on *i*th column. The point in the upper part of plot represents the proportion of “Good” response matrices on *i*th column, according to mQC assessment.

Drift is one of the systematic sources of variability that cannot be easily identified by Z’. HTS guideline suggests scatterplots to diagnose layout-dependent responses, but this can be infeasible for large scale cHTS due to different layout of dose combinations. Figure 7 and Table 2 showed four screens (assay ID 702, 703, 704, 705) from which we have observed significant left-to-right drift effects. In these cases, Z’ failed to identify such drift effect because the negative and positive controls were placed at the left four columns (see Supplementary Dataset S2 for plate layout and Supplementary Fig. S5H–K for QC summary). mQC which assesses the negative control and variation of does responses in the response matrices, on the other hand, have successfully flagged these four screens for violation of “screenings containing >60% Good response matrices and >90% Good or Medium response matrices” criteria. We found that the proportion of the “Good” response matrices correlated with the drift trend across the columns (lower plots in Fig. 7).

## Discussion

In this article we have introduced a predictive, interpretable, matrix-level screening QC metric, mQC, based on heuristic features. mQC has the potential to serve as a QC filter for prioritizing drug combinations and a tool for troubleshooting failed combination screens. Our analysis also suggests that the combination of plate-level QC and matrix-level QC will provide a more accurate assessment of the quality of a drug combination screen.

mQC focused on identifying unreliable response matrices that lead to erroneous or misleading characterization of synergy, as we showed in the “Z’ fails to correlate with expert opinions of matrix-level quality” and “prospective use of mQC” sections. However, mQC is still a conservative model that tolerates sporadic random error and non-monotonic dose responses in the activity landscape that can ultimately result in counterintuitive synergy pattern. We allow this flexibility in mQC because (1) we are trying to avoid overfitting the crowdsourced evaluations of response matrix quality; (2) for some assay readouts, such as apoptosis assays using Caspase-Glo, bell-shaped dose responses can be observed (Caspase activity is high when cells are dying, but low when cells are dead); and (3) there are special cases where synergy and antagonism coexist in a concentration-dependent fashion^23^. In addition, screening performed using a smaller matrix is more likely to miss the signal or bias the error at some concentration(s). This is another reason that 10 × 10 response matrices obtained smoother activity landscape than 6 × 6 (see retrospective use of mQC section), and this also highlights the importance of accurate single agent dose response experiments run before the a combination screening program.

Our retrospective analysis has confirmed that Z’ alone is insufficient to evaluate the overall quality of a response matrix or a cHTS campaign. Because of the focused nature of the collection and high number of actives, a Z-factor based on the sample field activity is not meaningful. Due to this reason, we developed an orthogonal metric, mQC, to suggest overall reliability of cHTS based on the assumption that the probability of a failed cHTS having a majority number of non-random response matrices is extremely low. Another motivation to implement matrix-level QC metric is that the large combinatorial space remarkably limits the number of control wells in combination screening. A cost-efficient plate layout shown in Supplementary Dataset S2 has 128 control wells, which accounts for only 8.3% in a 1536-well plate and therefore may not indicate the plate effect or other technical issue (such as spotting error) occurred in the rest of 91.7% plate. We experience a significant amount of screenings where Z’ < 0.3 but reliable response matrices dominate, and cases where only controls work due to various technical issues such as cell contamination, which led to a misleading Z’ > 0.5. Hence we have proposed a best practice guideline to evaluate the quality of a cHTS campaign using both plate-level and matrix-level QC metrics (Fig. 5B).

A limitation of this QC measure is that it is still unable to characterize the consistency of dose responses and synergy across the matrices. We observed some cases in a malaria screen^10^ where the replicate response matrices varied significantly even when mQC classifies them all as “Good” quality. Implementing a consistent QC metric at matrix-level remains challenging because (1) Combination screening data is still limited in terms of large scale availability; (2) While many synergy metrics have been defined, the question of which metric can serve as a well-defined endpoint, analogous to IC50, EC50 or AUC for single agent dose response, that can be used to compare between independent replicates is still open^24^. A candidate replacement is the minimum significant ratio (MSR)^25^ to indicate the consistency for “all versus all” combination screenings. However, MSR is restricted to the single agent part of the matrix, and consistency in single agent responses may not necessarily indicate the consistency in the combination sub-matrix. A robust statistical model is needed to translate the MSR concept to 2D response data. A second limitation is that mQC is restricted to pair-wise drug combination screening in a surface format. As this format is not well suited for the assessment of combinatory effect of two or more compounds, a more robust model needs to be designed for more complex combination screening platform. Finally, in “prospective use of mQC” section we have justified mQC for its use in quality control of cHTS based on Bliss independence model. Knowing that mQC is trained based on subjective human assessments, we use DBNorm as a more objective reference and confirmed that mQC suggests the experimental noise. Further validation using other additivity model (e.g., Loewe model) or experimental data may be required, as Bliss independence may not necessarily represent the mechanisms of action of drug combination^24^,^26^.

Quality is a result of both biological (e.g., robust assay window) and technical (e.g., accuracy and reproducibility in reagent/compound addition) effects. We have discussed some cases where mQC successfully identified technical issues in cell lines, assay readouts and compound batches. On the other hand, it can be hard to pinpoint the biological/non-biological cause unless documented. In addition, our analysis is based on a naïve model in which the factors (cell, compound, readout, size of matrix) are non-interacting. Instead, there are many more factors to consider, such as concentration, time point, layout, and interactions between these factors. For instance, one of the major reasons for the poor quality of Caspase-Glo readout is that apoptosis usually occurs within a time window, and this window can vary by cell line, compound mechanism of action and concentration. This makes Caspase-Glo assay extremely challenging for cHTS as the time point is always fixed in a screening (which can be addressed by running the experiment at multiple time points, which is obviously resource intensive).

Another source of poor quality combination response data is low cell viability on the day of assay plating or overall grows slowly in culture. In most cases, assay optimization prior to screening is limited which results in the majority of screens being tested at 500 cells per well and 48 hour time point. These parameters work for most medium sized, adherent and fast growing cancer cell lines; however, there are slow growing, large and/or suspension cell lines that may need more than 500 cells per well and/or a longer time point. Another technical issue unique to cHTS compared to conventional single-agent HTS is that compounds are usually preplated to reduce the time-dependent variation and therefore cells are added directly to the plates containing compound. There are cases where cell line cannot handle this transition very well, so we need to switch the plating order to let such cell adapt to the stress of dissociation and plating (4 hours or 24 hours) before adding compounds.

## Methods

### Crowdsourcing survey

To ensure the diversity of the checkerboard pattern, we first separated 127,119 response matrices from NCATS database into 32 groups using the 5 heuristic criteria described by Mott *et al*.^10^ (see below). Then we performed K-means clustering analysis for each group and finally selected 133 matrices which are closest to respective cluster center. Figure 3A illustrates the paradigm of how 133 blocks were selected in this study. In order to mitigate the bias, we randomized the order of blocks, and give an overview of all 133 blocks at the start of the survey to ensure that the participants define their criteria before polling and remain consistent during the survey. The participants labeled each response matrix as “Good”, “Medium” or “Bad”. Here we can justify that nine participants are enough to achieve robust consensus because the inter-rater agreements (measured by Fleiss’ κ^27^) converge to 0.35 as the number of raters increases (Supplementary Fig. S1). The final label for each response matrix was computed using the majority vote rule and these final labels were used to as the training set. The class breakdown showed 78 “Bad”, 24 “Medium” and 24 “Good” matrices. The remaining 7 received ambiguous votes (equal votes were received for two or more labels), hence excluded from the training set. The response matrices and survey results are available in Supplementary Dataset S1.

### Matrix-level QC metric, mQC

The combination response matrices (blocks) were performed in *N* × *N* matrix format, in which each axis corresponds to the treatment of a respective compound at a certain concentration. Here we denote 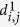 and 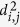 as the respective concentration of first and second compound and 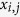 as the corresponding effect at 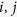. Specifically, we place DMSO control at the bottom right corner 
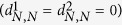
. The single-agent treatments are placed on the bottom row (*N*, *) and right column (*, *N*) (* = any coordinate except *N*). The dose combinations are placed in the (*N* − 1) × (*N* − 1) top left submatrix in which the individual drug concentration is in descending order, namely 
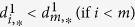
, 
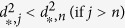
. See Supplementary Dataset S2 for a block design example.

We consider a robust matrix-level QC metric to be:

1. An orthogonal metric to the plate-level QC (e.g., Z’ or Z-factor);
2. A generalized metric that makes robust quality prediction for responses with any matrix dimension (that is, 6 × 6, 10 × 10 and so on), normalization scheme (normalized by activator/inhibitor/neutral control), assay readout (CellTiter-Glo, Caspase-Glo, etc), and compound concentrations;
3. An interpretable metric that represents the basic quality and usability of a combination screening block and is consistent with human intuition;
4. A metric to be used as a QC filter for prioritizing drug combinations and a tool for debugging failed screens based on simple statistics.

An initial attempt at defining the mQC metric was described by Mott *et al*.^10^. This metric assesses the matrix-level quality using a weighted-sum of five heuristic criteria (assuming plate data were normalized to the 100% (DMSO) and 0% (full cell kill)):

1. DMSO response 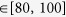;
2. Valid IC50 for both single agent dose responses;
3. Relative standard deviations for both single agent dose responses > 20;
4. Relative standard deviation for dose combination sub-matrix > 25;
5. Spatial autocorrelation p-value (tested by Moran’s I) < 0.05.

In the current implementation, we extend this QC using the following feature set:

1. (dmso.v) Normalized response of the negative control. For CellTiter-Glo, ROS-Glo, SYBR green readouts in which the positive control lowers the signal, the expected signal of negative control (DMSO) is 100. To make it consistent, we use (100-negative control) for those readouts where positive control enhances the signal, such as Caspase-Glo and other reporter assay.
2. (sa.min) The smaller relative standard deviation of the single-agent dose response.
3. (sa.max) The larger relative standard deviation of the single-agent dose response.
4. (sa.matrix) The relative standard deviation of the dose combination sub-matrix.
5. (moran.p) p-value for spatial autocorrelation (tested by Moran’s I).
6. (smoothness.p) p-value for smoothness: We perform hypothesis testing based on the following hypotheses: H_0_ = the activity landscape is rugged and Ha = the activity landscape is smooth. The matrix responses are fitted to a generalized additive model (GAM) using R mgcv package. The smoothness of the activity landscape is measured by the RMSD between fitted and the measured values such that a smooth landscape should be fitted perfectly using GAM model (RMSD = 0), while a rugged landscape has a non-zero RMSD, with larger values corresponding to increasing ruggedness. To generalize the model, we bootstrap the matrix 10,000 times and calculate the empirical cumulative distribution function (ecdf). The p-value is calculated as the probability of being a random (usually rugged) landscape having an RMSD smaller than the observed one.
7. (mono.v) Likelihood of monotonic dose responses. The monotonic dose response is based on the common observation that the higher the dose, the greater the effect. Although non-monotonic dose response exists in nature^28^, we assume, for simplicity, that the combination dose response is monotonic. For CellTiter-Glo assay, we expect a monotonically decreasing CellTiter-Glo signals in the dose combination submatrix and denote the likelihood of monotonic dose responses as

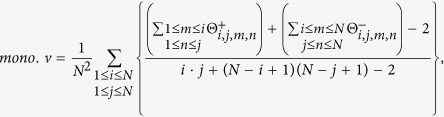



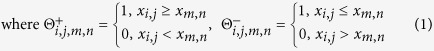


For Caspase-Glo, we expect a monotonically increasing Caspase activity in the dose combination submatrix, therefore we exchange 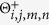 and 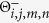 in the equation (1). This feature is designed so that the mono.v for monotonic dose response that is consistent with common sense is 1, monotonic dose response that completely violates the common sense is 0, and 0.5 for a random response matrix. This metric by definition will tolerate the local violation of monotonicity, which tends to be the usual case of non-monotonic dose responses^28^.

The mQC Adaboost classifier^29^ was trained based on the aforementioned 7 features and the 126 crowdsourcing responses. To validate the predictive ability, we performed 200 random splits for each of 16 test set proportion ranging 16 proportion of test set ranging from 5% to 80%. We used multiclass Matthews Correlation Coefficient (MCC) to evaluate the classification accuracy, as described in ref. 30. Briefly, we denote the confusion matrix 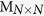 in which 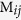 is the number of cases that belongs to true class *i* and the classifier assigned as class *j*. Then the multiclass MCC is


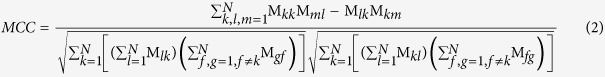


The recall and precision of class *i* are defined as
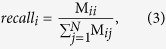



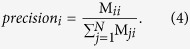


The standard deviation of three-class probability (good, medium or bad) was fitted with respect to the error rate for each case using local polynomial regression (loess function in R). The source code in R is available at http://matrix.ncats.nih.gov/mQC.

### Plate-level QC, Z’ (Z-factor) and SSMD

Z’ is defined as
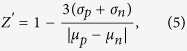


where 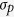, 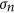 are the standard deviations of positive and negative controls, and 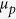, 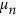 are the mean of the positive and negative controls^31^. In case of outlier, we calculate the robust SSMD as


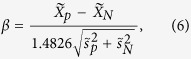


where 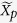, 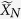, 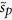, 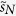 are the median and median absolute deviation (MAD) of the positive and negative control^1^. Noticing the fact that Z’ or SSMD may not hold if the controls are placed on one side in the presence of dramatic plate effect, we have also calculated Z’ (sample) and SSMD (sample) using the block DMSO controls and original positive controls. We consider Z’ (sample) and SSMD (sample) meaningful only if the plate holds ≥12 blocks.

It worth noting that some plate layout information was not available for some plates due to being old screens, which prevents the computation of Z’ and SSMD. This leads to a total of 119,287 blocks (instead of 127,119 blocks) with available Z’ and SSMD values amenable for a fair Z’-mQC comparison. All plate-level and matrix-level QC metrics plate breakdown are available in Supplementary Dataset S3.

### Synergy metrics

Bliss synergy (excess over Bliss or delta Bliss) is based on the Bliss independent model^20^. Assuming pairs of compounds that have no mechanistic interaction, the expected response of a drug combination (C) is a multiplication of fractional inhibition upon treatment with drug A and B individually, C = A + B − A × B. Given a response matrix 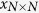 where the last row/column is the single agent dose response from drug A and B, and 100% exposure represents no cell killing and 0% represents full cell killing. The normalized delta-Bliss is defined as


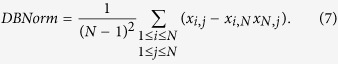


DBNorm ranges from −1 to 1 where 0, positive values and negative values represent additivity, synergism and antagonism, respectively.

To simulate the effect of systematic error and random error, we rewrite the DBNorm as


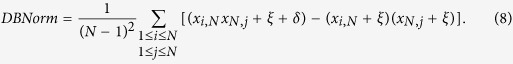


We denote 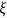 the systematic error attributable to each measurement and 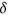 the variation of delta Bliss across the database. It is reasonable to assume normal distributions for these two sources of variation: 
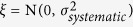
 and 
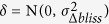
 where 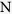 is the random deviates generator using normal distribution given the mean and variance, and 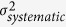 and 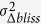 are the variance of systematic error and delta Bliss. Apart from systematic error, random error (e.g., dispense error, spotting error, poor cell health, etc.) can also present. The DBNorm distribution for random matrix responses can be written as:


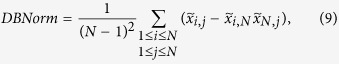


where the tilde denotes a random value ranging from 0 to 1.

γ is the parameter that minimizes the difference (*d*) between the observed combination effect and Gaddums non-interaction model^21^,


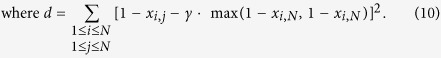


Values less than 1, greater than 1 and equal to 1 indicate synergy, antagonism and non-interaction, respectively.

### Database analysis

Here we removed ~4.8% of response matrices with mQC confidence lower than 0.6 during this analysis. We denote 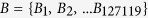 as the remaining 127,119 blocks from our in-house database, and we consider the quality of each response matrix *B*_*i*_ as a function of 4 independent variables: *B*_*i*_ ~ readout + matrix size + cell line + compound1 + compound 2. To reduce the bias caused by experimental design (e.g., some compounds were tested more frequently in combination than others), we compared different readouts or matrix size using a pairwise paired test. For example, to compare between CellTiter-Glo (CTG) and Caspase-Glo (CG) readouts, we performed the following hypothesis test:

H_0_: CellTiter-Glo performs equivalent to or worse than Caspase-Glo.

Ha: CellTiter-Glo performs better than Caspase-Glo.

In order to calculate the p-value, we exhaustively searched 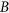 for a set of block pairs, 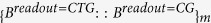, where other experimental settings (in this case, cell, matrix size and compound pairs) are identical. We denote *N*_+_*, N*_*0*_*, N*_*−*_ as the number of paired blocks where mQC of CTG is better, equal to, or worse than CG, respectively. The p-value is calculated as the probability of the mQC assessments of 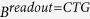 being significantly better than those of 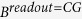:


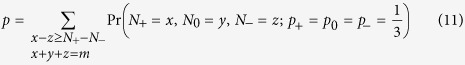


where m is the number of block pairs. The posterior probabilities (*p*_*+*_, *p*_*0*_, *p*_*−*_) for this multinomial distribution are 1/3. The p-values were adjusted by Benjamini-Hochberg procedure to control the false discovery rate^32^. We consider the adjusted p-value < 0.05 as a significant comparison. The same procedure was applied for matrix size comparison.

## Additional Information

**How to cite this article**: Chen, L. *et al*. mQC: A Heuristic Quality-Control Metric for High-Throughput Drug Combination Screening. *Sci. Rep.* **6**, 37741; doi: 10.1038/srep37741 (2016).

**Publisher’s note:** Springer Nature remains neutral with regard to jurisdictional claims in published maps and institutional affiliations.

## Supplementary Material

Supplementary Information

Supplementary Data 1

Supplementary Data 2

Supplementary Data 3

## Figures and Tables

**Table 1:** Feature importance.

Feature name	Importance	Comments
dmso.v	20.71	Normalized response of the negative control
smoothness.p	18.88	p-value for smoothness
moran.p	18.82	p-value for spatial autocorrelation (tested by Moran’s I)
mono.v	12.62	Likelihood of monotonic dose responses
sa.min	12.84	The smaller relative standard deviation of the single-agent dose response
sa.matrix	8.78	The relative standard deviation of the dose combination sub-matrix
sa.max	7.36	The larger relative standard deviation of the single-agent dose response

**Table 2:** Example drug combination screens and QC.

ID	Readout	Z’	SSMD	mQC (Good%)	Comments	Link
3785	CaspaseGlo	−0.54 ± 0.5	2.65 ± 1.62	91.7	Failed positive control, reasonable synergy	https://tripod.nih.gov/matrix-client/rest/matrix/blocks/3785/table
447	CellTiterGlo	0.65 ± 0.09	8.49 ± 1.29	100	Excellent screen	https://tripod.nih.gov/matrix-client/rest/matrix/blocks/447/table
241	CellTiterGlo	0.67 ± 0.07	9.97 ± 2.63	96.8	Excellent screen	https://tripod.nih.gov/matrix-client/rest/matrix/blocks/241/table
5021	CellTiterGlo	0.53 ± 0.19	9.56 ± 3.5	4.6	Cell contamination, good Z’ and SSMD	https://tripod.nih.gov/matrix-client/rest/matrix/blocks/5021/table
6028	CellTiterGlo	0.59 ± 0.19	8.97 ± 0.83	63.8	Cell contamination, good Z’ and SSMD	https://tripod.nih.gov/matrix-client/rest/matrix/blocks/6028/table
2850	CellTiterGlo	0.75 ± 0.02	13.1 ± 2.89	95.8	Excellent screen	https://tripod.nih.gov/matrix-client/rest/matrix/blocks/2850/table
2852	CellTiterGlo	0.70 ± 0.03	8.84 ± 1.7	54.2	Cell viability issue, good Z’ and SSMD	https://tripod.nih.gov/matrix-client/rest/matrix/blocks/2852/table
702	CellTiterGlo	0.76 ± 0.03	12.39 ± 1.9	21.2	Good Z’ and SSMD, drift	Internal data (link not available)
703	CellTiterGlo	0.76 ± 0.03	13.23 ± 2.1	22.5	Good Z’ and SSMD, drift	Internal data (link not available)
704	CellTiterGlo	0.76 ± 0.02	11.4 ± 1.6	31.5	Good Z’ and SSMD, drift	Internal data (link not available)
705	CellTiterGlo	0.78 ± 0.03	14 ± 1.6	29.2	Good Z’ and SSMD, drift	Internal data (link not available)
