# Supplementary material for: mQC: A Heuristic Quality-Control Metric for High-Throughput Drug Combination Screening
Source: Sci Rep. 2016 Nov 24;6:37741. doi: 10.1038/srep37741 (PMC5121902; doi:10.1038/srep37741)
Supplement: Supplementary Information [file srep37741-s1.pdf]

Supplementary Materials for

mQC: A Heuristic Quality-Control Metric for High-Throughput Drug Combination Screening

Lu Chen, Kelli Wilson, Ian Goldlust, Bryan T. Mott , Richard Eastman, Mindy I. Davis, Xiaohu Zhang, Crystal McKnight, Carleen Klumpp-Thomas, Paul Shinn, John Simmons, Mike Gormally, Sam Michael, Craig J. Thomas, Marc Ferrer, Rajarshi Guha

Supplementary Dataset S1. The 133 response matrices used for training and the survey results.

Supplementary Dataset S2. The layout for 6×6 and 10×10 screens (1536-well plate).

Supplementary Dataset S3. Plate-level and matrix-level QC metrics breakdown by plate.

Supplementary Figure S1. Relationship between number of raters and the inter-rater agreement (measured by Fleiss' κ).

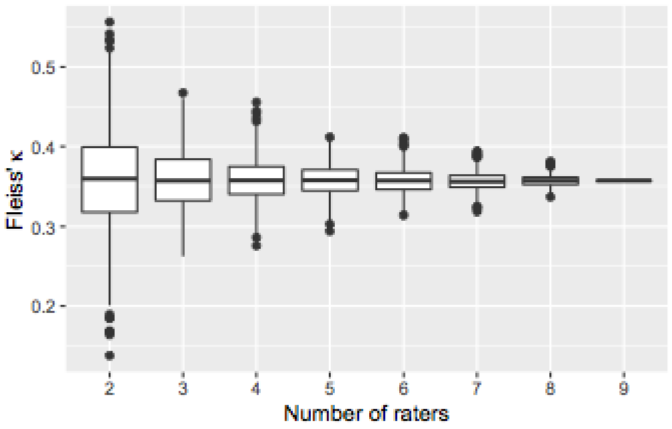

Supplementary Figure S2. Correlation between plate-level QC (i.e., Z', SSMD) and matrix-level QC (mQC) (breakdown by assay plate). The plates with negative Z' or SSMD < -75 are excluded.

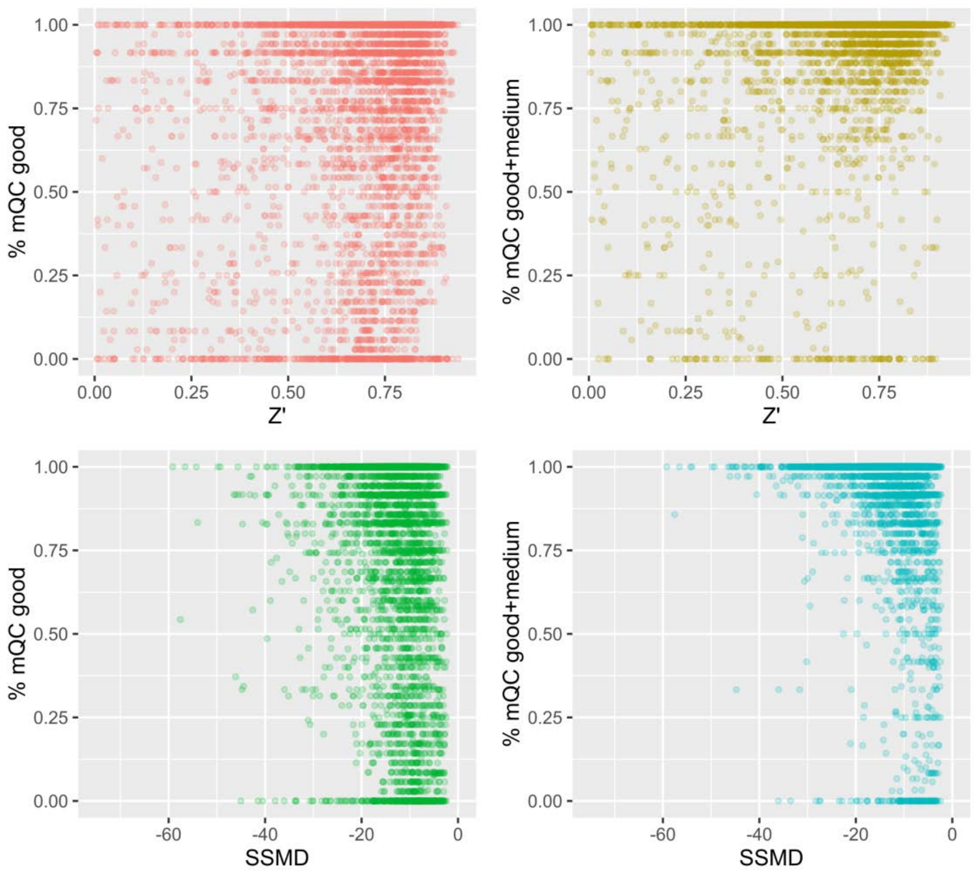

Supplementary Figure S3. Correlation between plate-level QC (i.e., Z' or SSMD calculated using plate-level positive controls and block DMSO controls) and matrix-level QC (mQC) (breakdown by assay plate). The plates with negative Z' or SSMD < -75 are excluded.

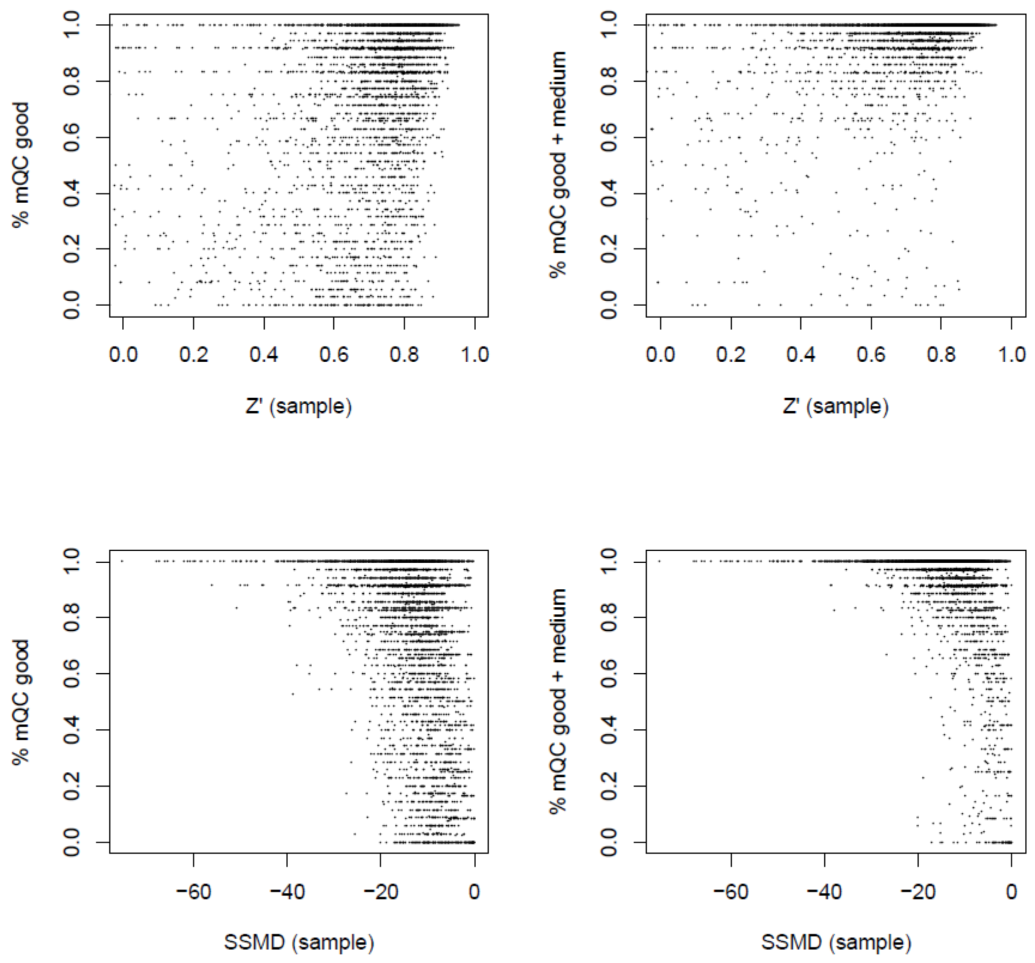

Supplementary Figure S4. Correlation between Z' (plate) or SSMD (plate) computed using plate-level positive and negative controls and Z' (sample) or SSMD (sample) computed using plate-level positive controls and block DMSO controls. The plates that hold less than 12 blocks or have negative Z' or SSMD < -40 are excluded in this analysis.

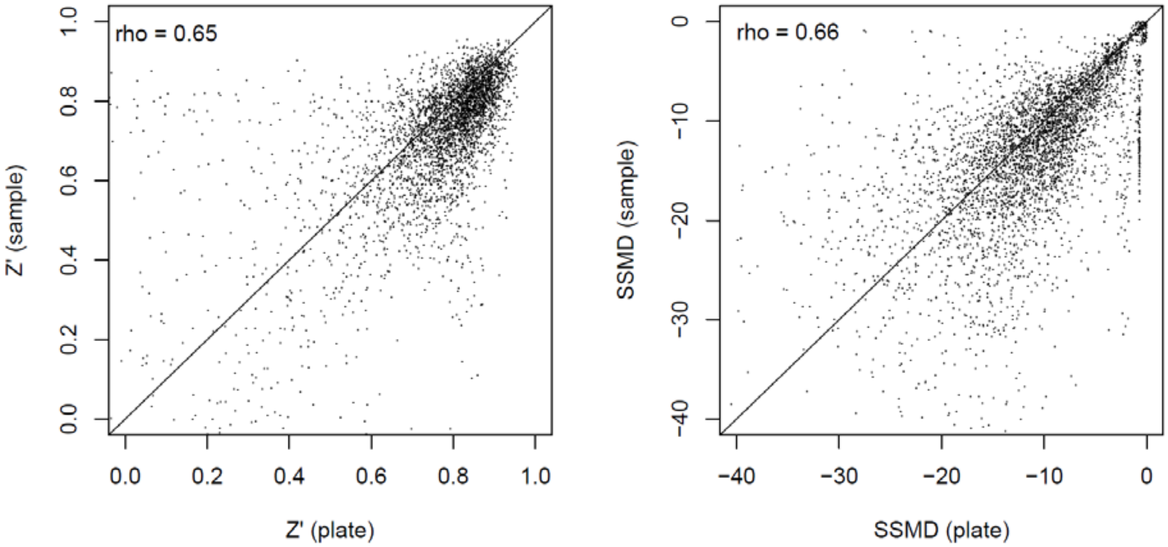

Supplementary Figure S5. Plate-level QC and mQC for 11 screens. For each assay, we show a bar plot summary comparing per-plate QC metrics (left) and scatter plots of the plate-level controls (right, red=positive controls, green=negative controls). (A) CaspaseGlo assay 3785: failed positive control, good mQC; (B) CellTiterGlo assay 447: excellent assay; (C) CellTiterGlo assay 241: excellent screen; (D) CellTiterGlo assay 5021: cell contamination; (E) CellTiterGlo assay 6028: cell contamination; (F) CellTiterGlo assay 2850: excellent screen; (G) CellTiterGlo assay 2852: low cell viability; (H) CellTiterGlo assay 702: drift; (I) CellTiterGlo assay 703: drift; (J) CellTiterGlo assay 704: drift; (K) CellTiterGlo assay 705: drift. Each assay was performed on various number of 1536-well plates, and we analyzed the quality of each plate using the median and standard deviation of positive control signals (red), negative control signals (yellow), Z'-factor, robust SSMD, and the proportion of "Good" (red) and "Medium" (green) response matrices (assessed by mQC). The dashed lines in Z' and SSMD plots are the empirical Z' or SSMD cutoffs (Z'=0.5 and SSMD=-7). For visualization purpose, we use the negative robust SSMD values.

A

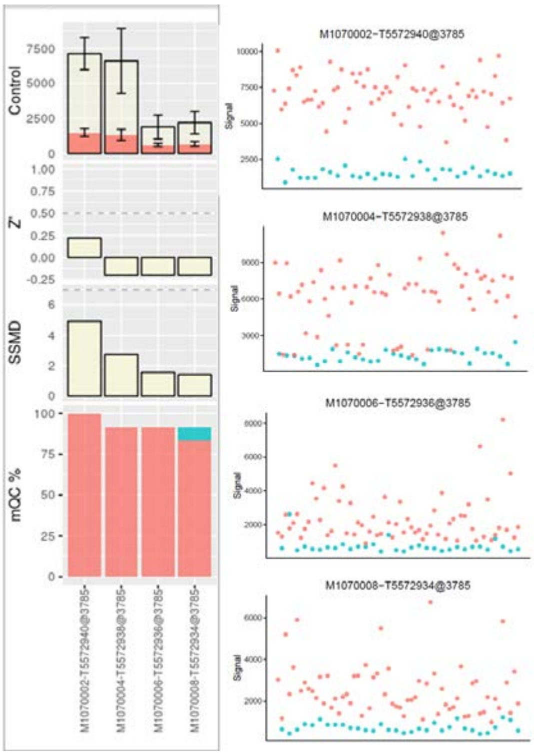

B

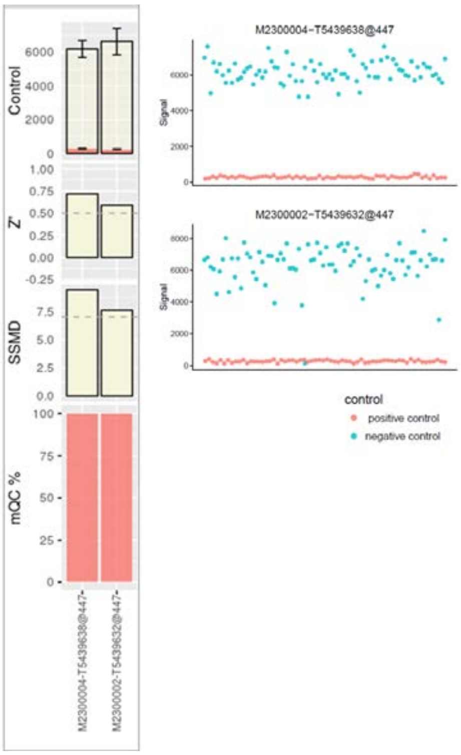

C

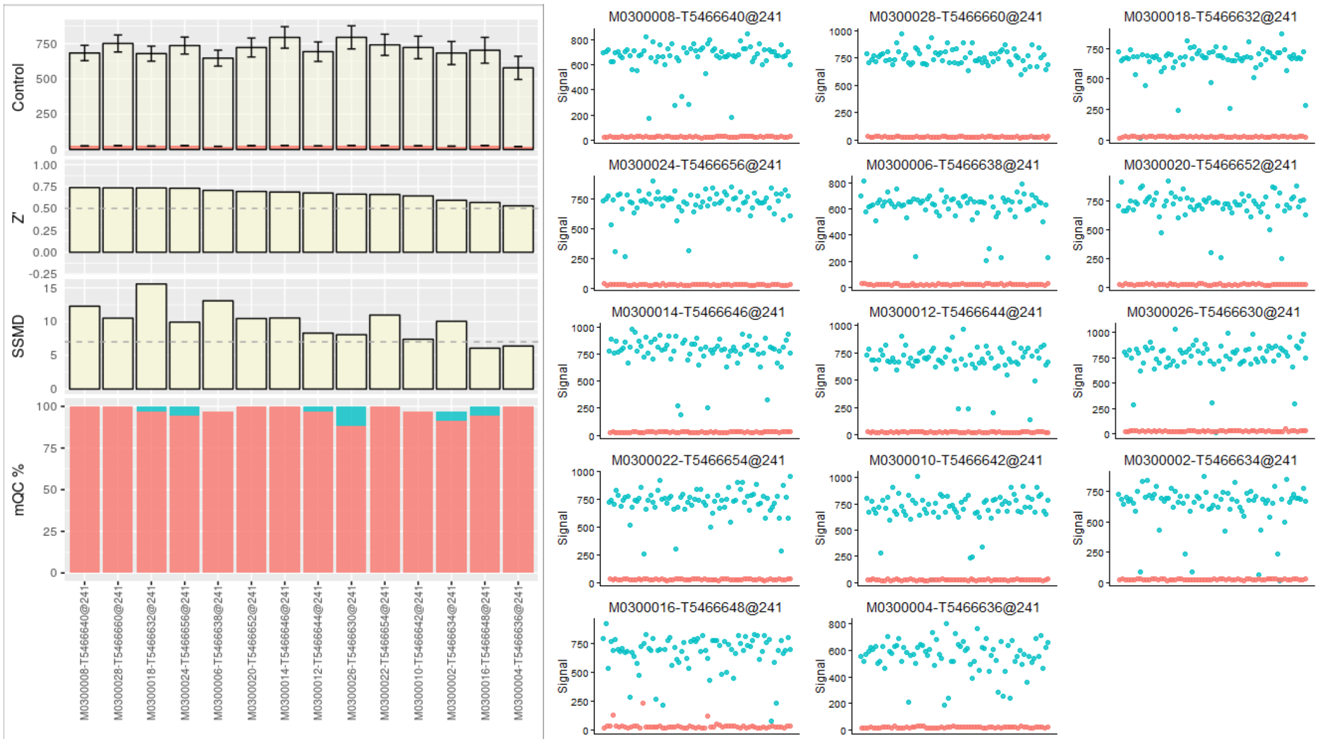

Control
Z'
SSMD
mQC %
M0300008-T5466640@241
M0300028-T5466660@241
M0300018-T5466632@241
M0300024-T5466656@241
M0300006-T5466638@241
M0300020-T5466652@241
M0300014-T5466646@241
M0300012-T5466644@241
M0300026-T5466630@241
M0300022-T5466654@241
M0300010-T5466642@241
M0300002-T5466634@241
M0300016-T5466648@241
M0300004-T5466636@241
Signal

D

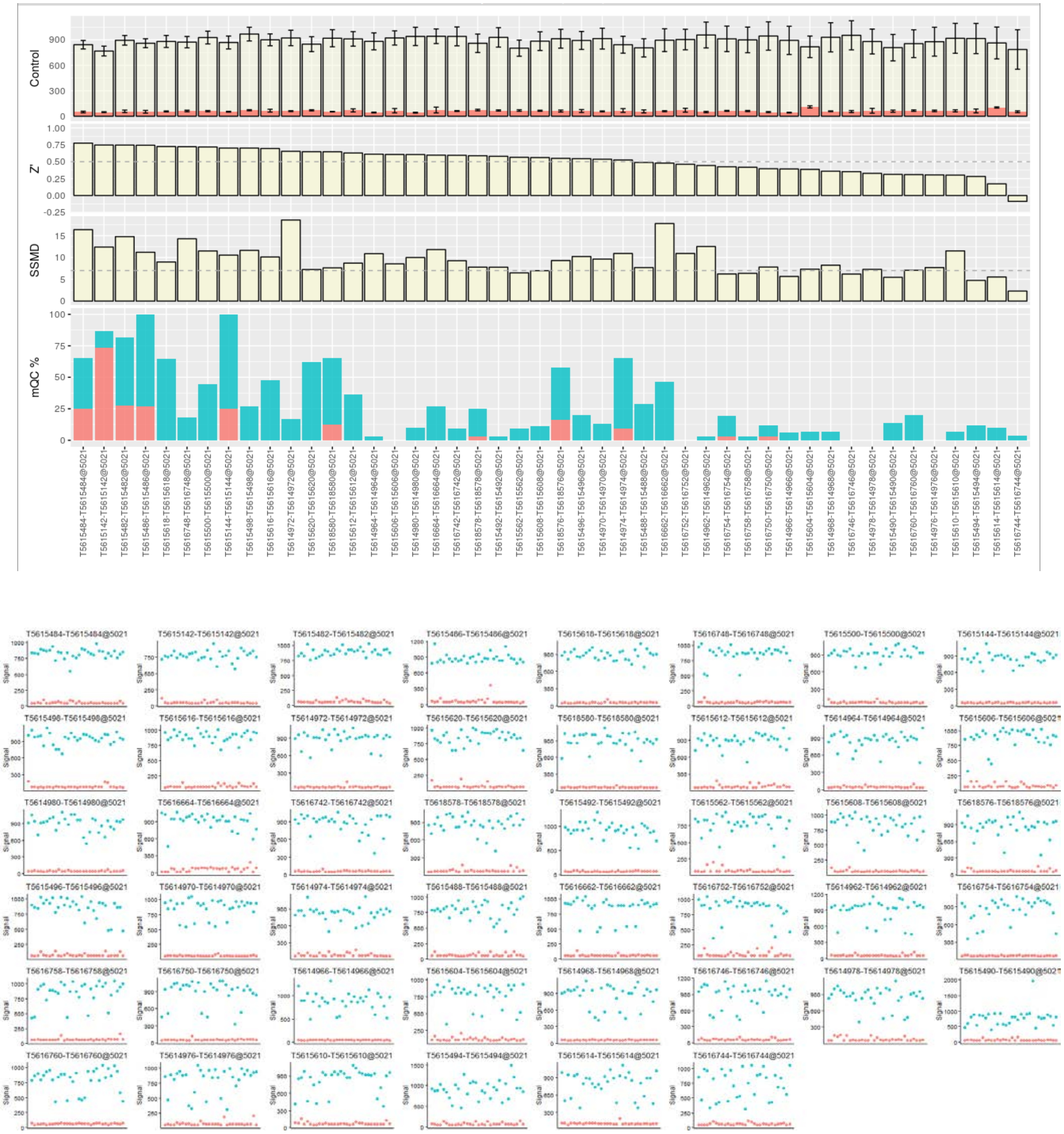
Control
Z'
SSMD
mQC %
Signal
T5615484-T5615484@5021
T5615142-T5615142@5021
T5615482-T5615482@5021
T5615486-T5615486@5021
T5615618-T5615618@5021
T5616748-T5616748@5021
T5615500-T5615500@5021
T5615144-T5615144@5021
T5615498-T5615498@5021
T5615616-T5615616@5021
T5614972-T5614972@5021
T5615620-T5615620@5021
T5618580-T5618580@5021
T5615612-T5615612@5021
T5614964-T5614964@5021
T5615606-T5615606@5021
T5614980-T5614980@5021
T5616664-T5616664@5021
T5616742-T5616742@5021
T5618578-T5618578@5021
T5615492-T5615492@5021
T5615562-T5615562@5021
T5615608-T5615608@5021
T5618576-T5618576@5021
T5615496-T5615496@5021
T5614970-T5614970@5021
T5614974-T5614974@5021
T5615488-T5615488@5021
T5616662-T5616662@5021
T5616752-T5616752@5021
T5614962-T5614962@5021
T5616754-T5616754@5021
T5616758-T5616758@5021
T5616750-T5616750@5021
T5614966-T5614966@5021
T5615604-T5615604@5021
T5614968-T5614968@5021
T5616746-T5616746@5021
T5614978-T5614978@5021
T5615490-T5615490@5021
T5616760-T5616760@5021
T5614976-T5614976@5021
T5615610-T5615610@5021
T5615494-T5615494@5021
T5615614-T5615614@5021
T5616744-T5616744@5021

E

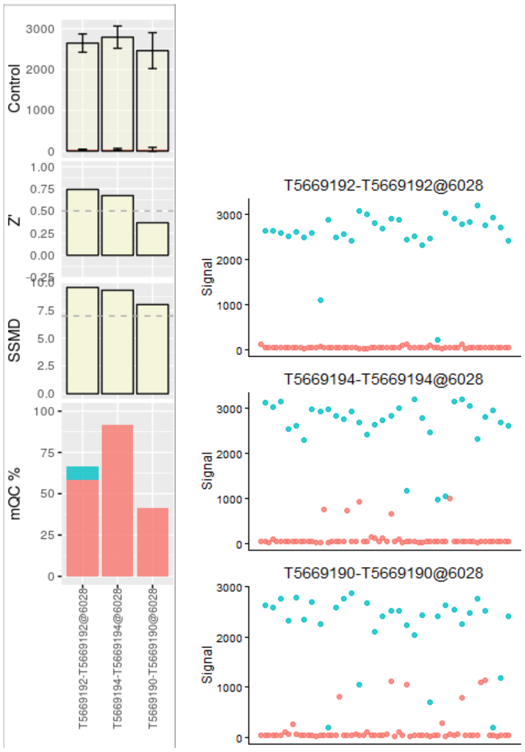
Control
Z'
SSMD
mQC %
T5669192-T5669192@6028
T5669194-T5669194@6028
T5669190-T5669190@6028
Signal

F

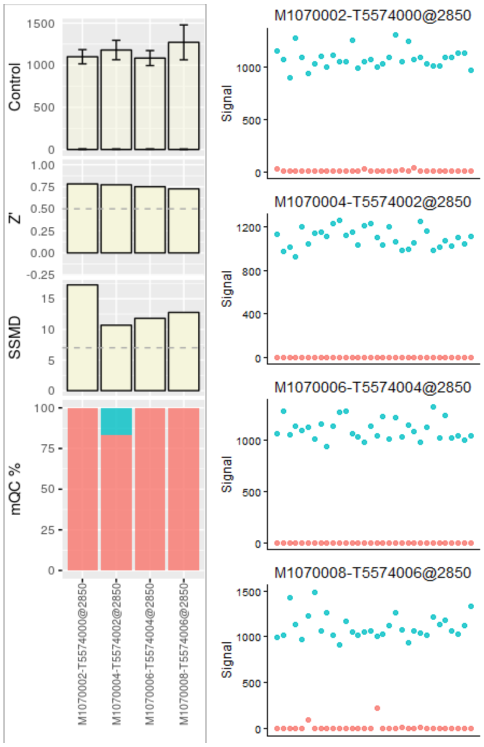
Control
Z'
SSMD
mQC %
M1070002-T5574000@2850
M1070004-T5574002@2850
M1070006-T5574004@2850
M1070008-T5574006@2850
Signal

G

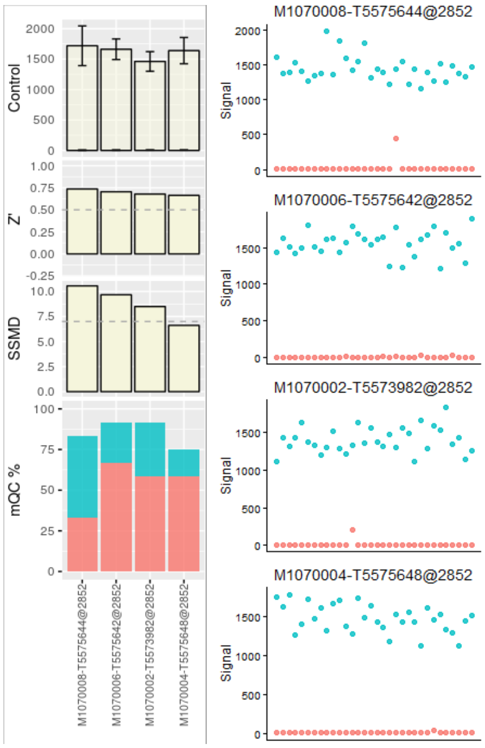
Control
Z'
SSMD
mQC %
M1070008-T5575644@2852
M1070006-T5575642@2852
M1070002-T5573982@2852
M1070004-T5575648@2852
Signal

H

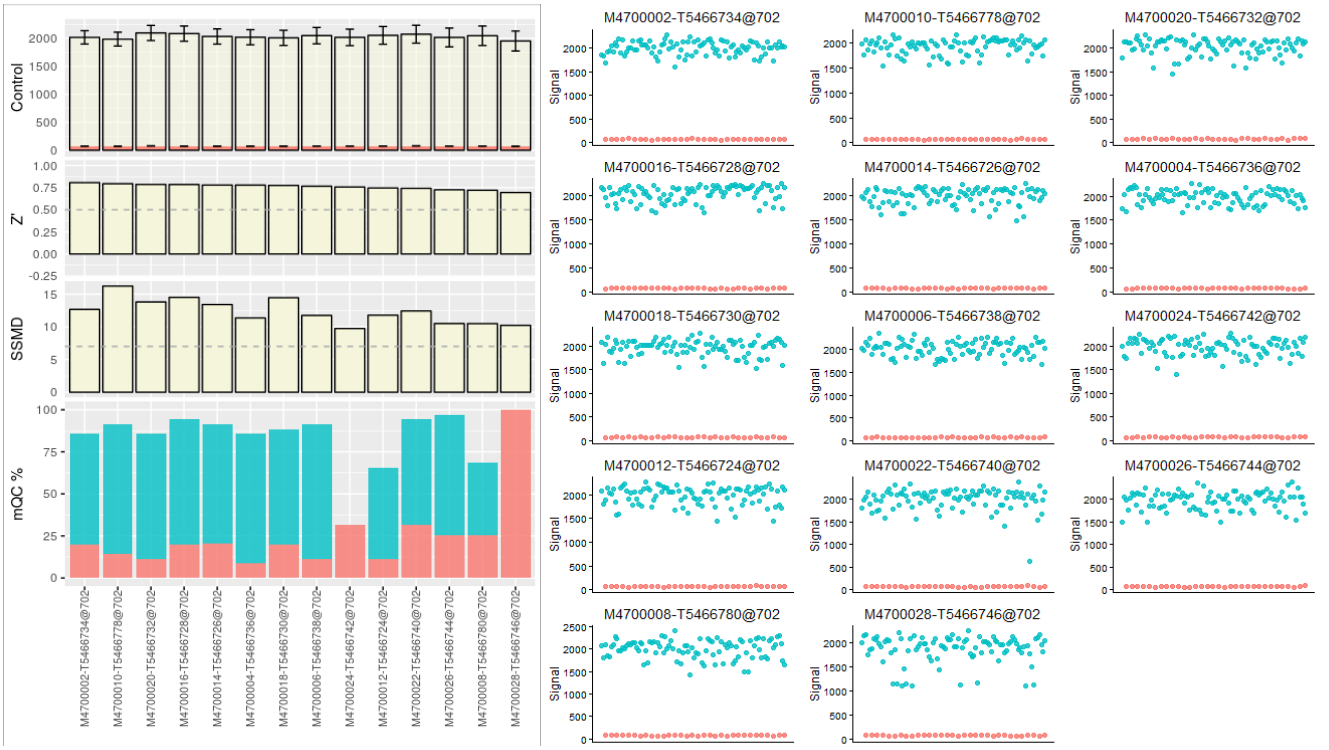
Control
Z'
SSMD
mQC %
Signal
M4700002-T5466734@702
M4700010-T5466778@702
M4700020-T5466732@702
M4700016-T5466728@702
M4700014-T5466726@702
M4700004-T5466736@702
M4700018-T5466730@702
M4700006-T5466738@702
M4700024-T5466742@702
M4700012-T5466724@702
M4700022-T5466740@702
M4700026-T5466744@702
M4700008-T5466780@702
M4700028-T5466746@702

I

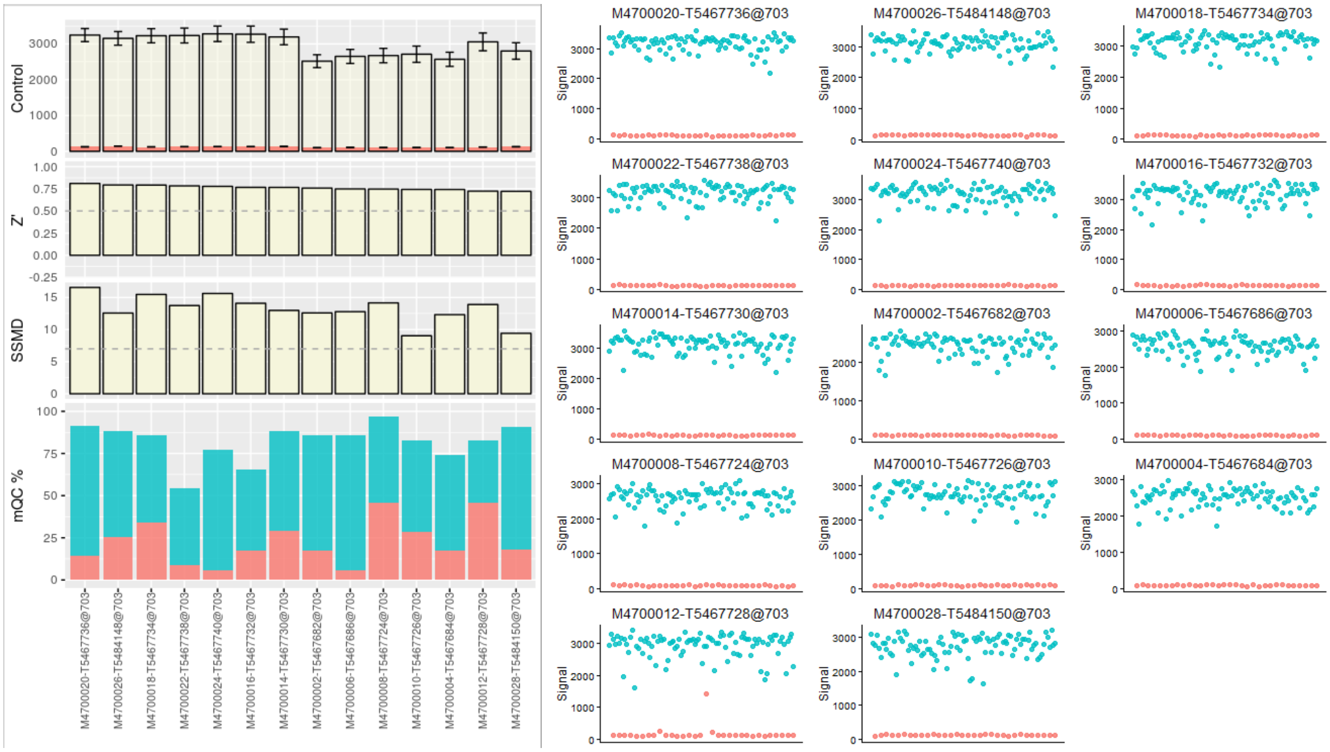
Control
Z'
SSMD
mQC %
Signal
M4700020-T5467736@703
M4700026-T5484148@703
M4700018-T5467734@703
M4700022-T5467738@703
M4700024-T5467740@703
M4700016-T5467732@703
M4700014-T5467730@703
M4700002-T5467682@703
M4700006-T5467686@703
M4700008-T5467724@703
M4700010-T5467726@703
M4700004-T5467684@703
M4700012-T5467728@703
M4700028-T5484150@703

J

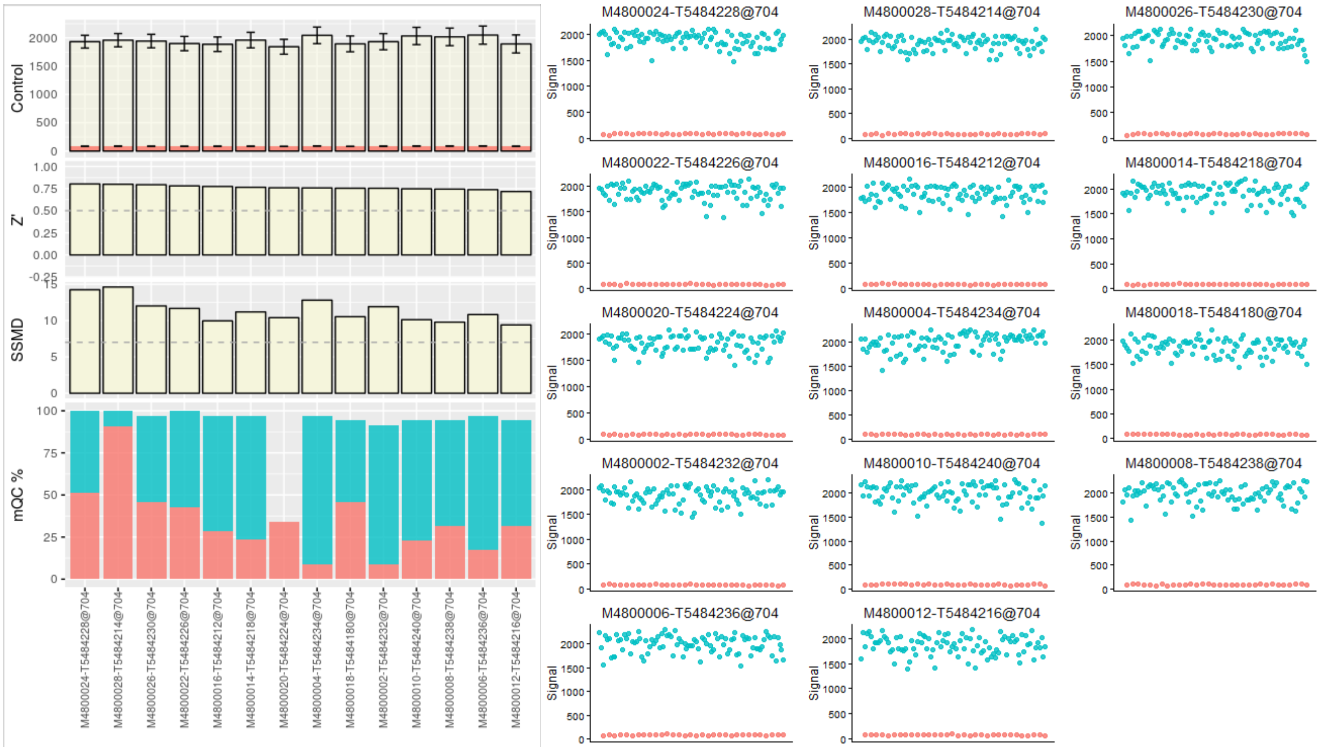
Control
Z'
SSMD
mQC %
M4800024-T5484228@704
M4800028-T5484214@704
M4800026-T5484230@704
M4800022-T5484226@704
M4800016-T5484212@704
M4800014-T5484218@704
M4800020-T5484224@704
M4800004-T5484234@704
M4800018-T5484180@704
M4800002-T5484232@704
M4800010-T5484240@704
M4800008-T5484238@704
M4800006-T5484236@704
M4800012-T5484216@704
Signal

K

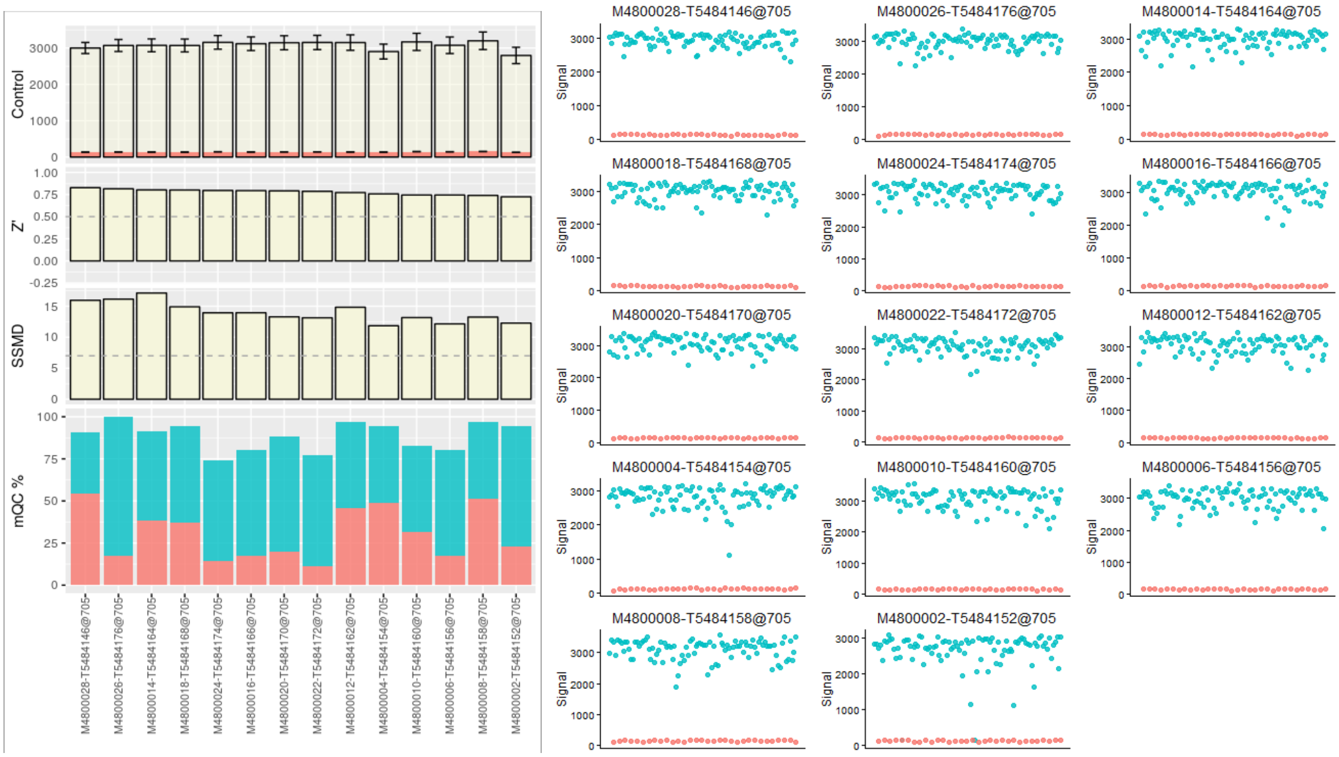
Control
Z'
SSMD
mQC %
M4800028-T5484146@705
M4800026-T5484176@705
M4800014-T5484164@705
M4800018-T5484168@705
M4800024-T5484174@705
M4800016-T5484166@705
M4800020-T5484170@705
M4800022-T5484172@705
M4800012-T5484162@705
M4800004-T5484154@705
M4800010-T5484160@705
M4800006-T5484156@705
M4800008-T5484158@705
M4800002-T5484152@705
Signal

Supplementary Figure S6. The 133 blocks used to train mQC model and their corresponding plate data (shown as heatmap). Red: plate-level positive controls. Green: plate-level negative control (e.g., DMSO). Magenta: the selected block.

Block 1 (T5615528−T5615528)

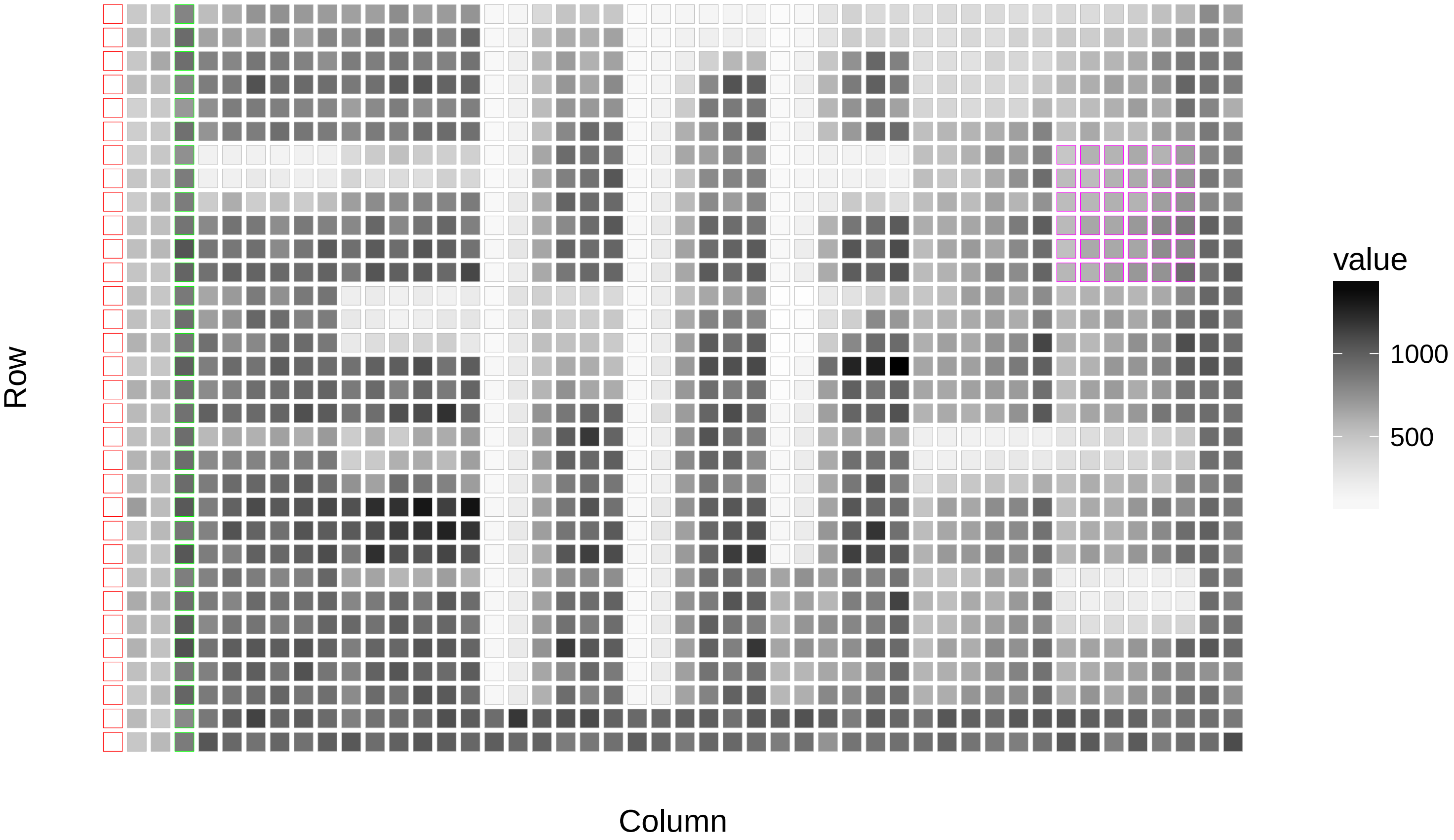

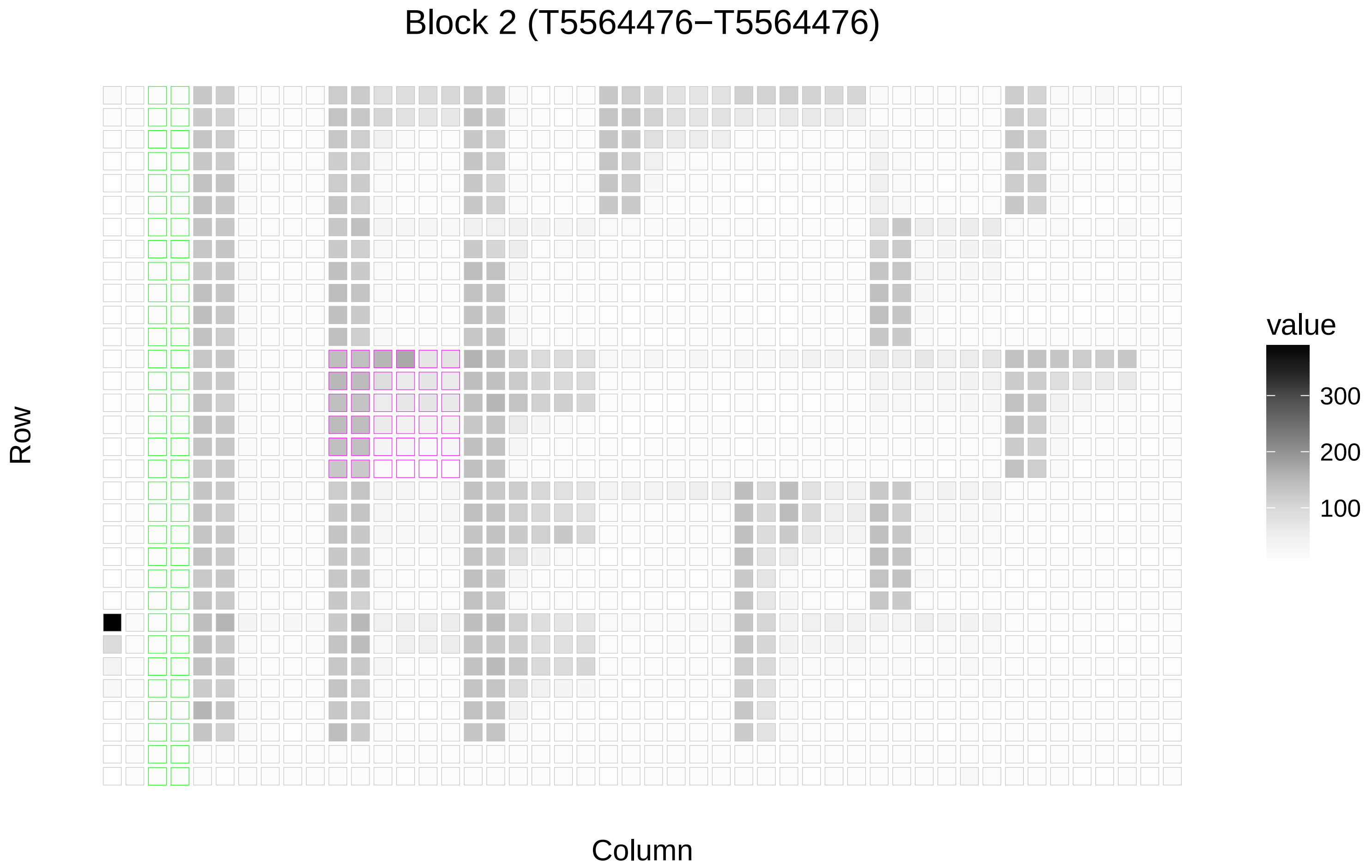
Block 2 (T5564476−T5564476)
Row
Column
value
300
200
100

Block 3 (M1020030−T5571470)

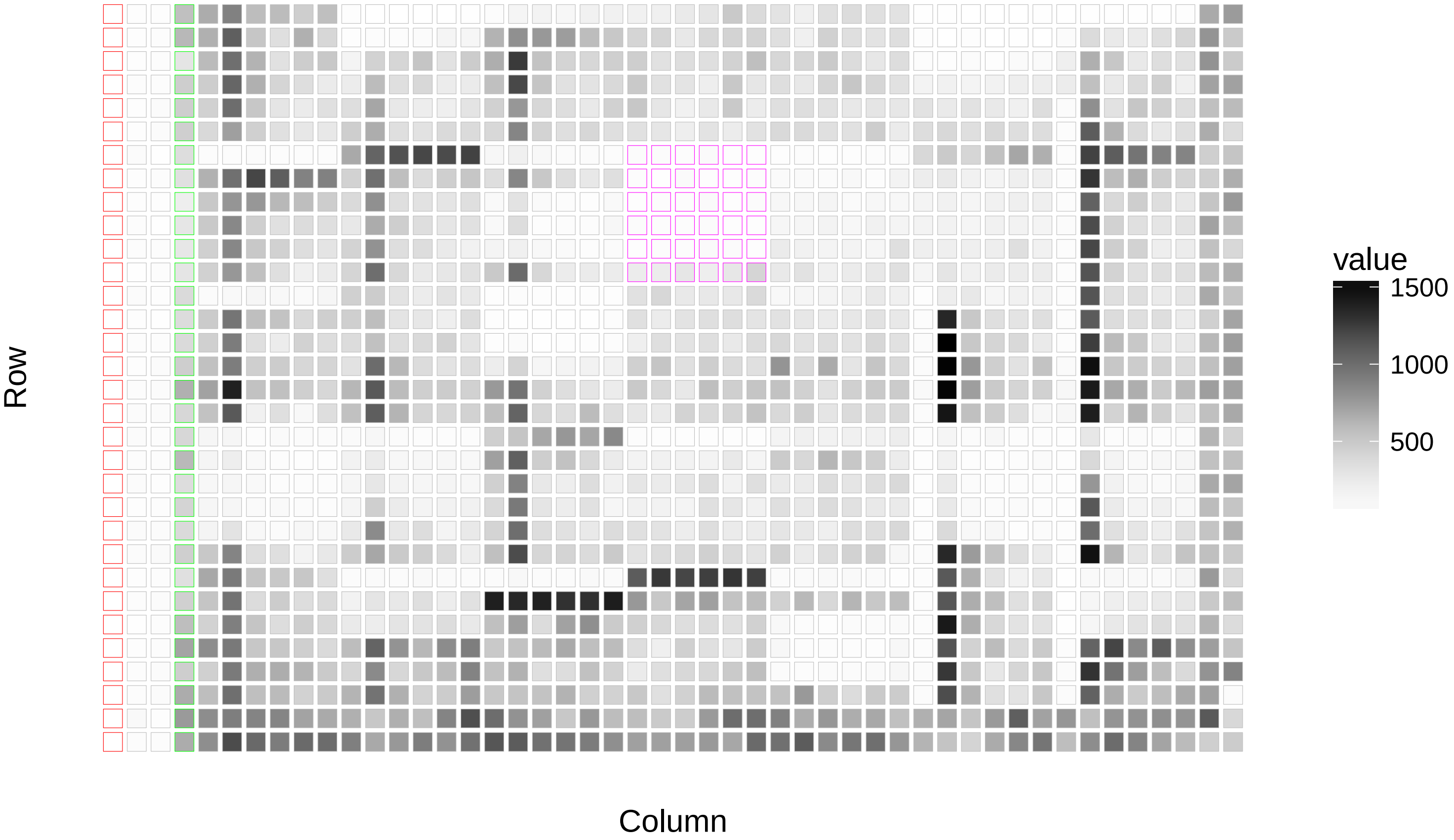

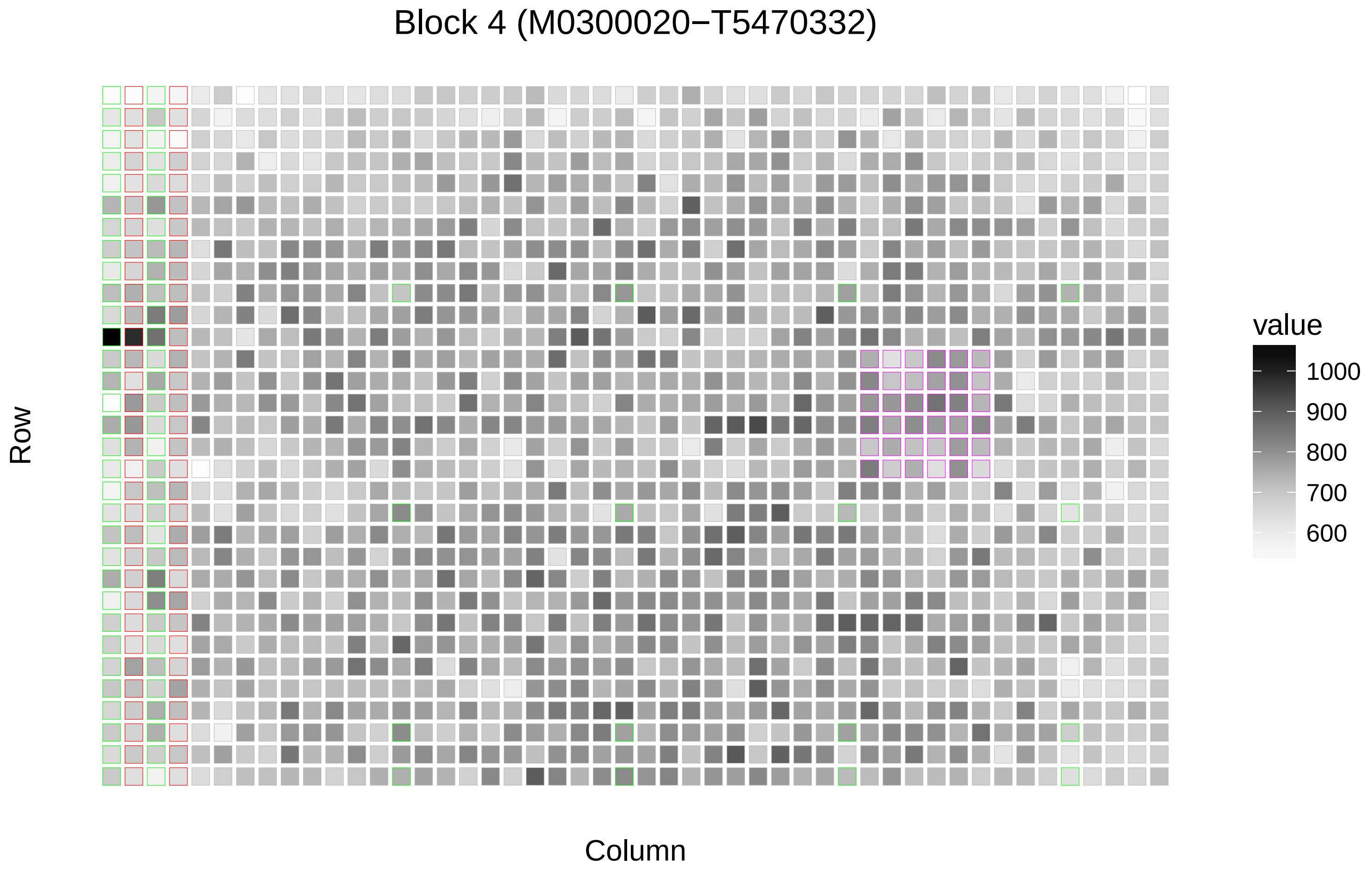
Block 4 (M0300020−T5470332)
Row
Column
value
1000
900
800
700
600

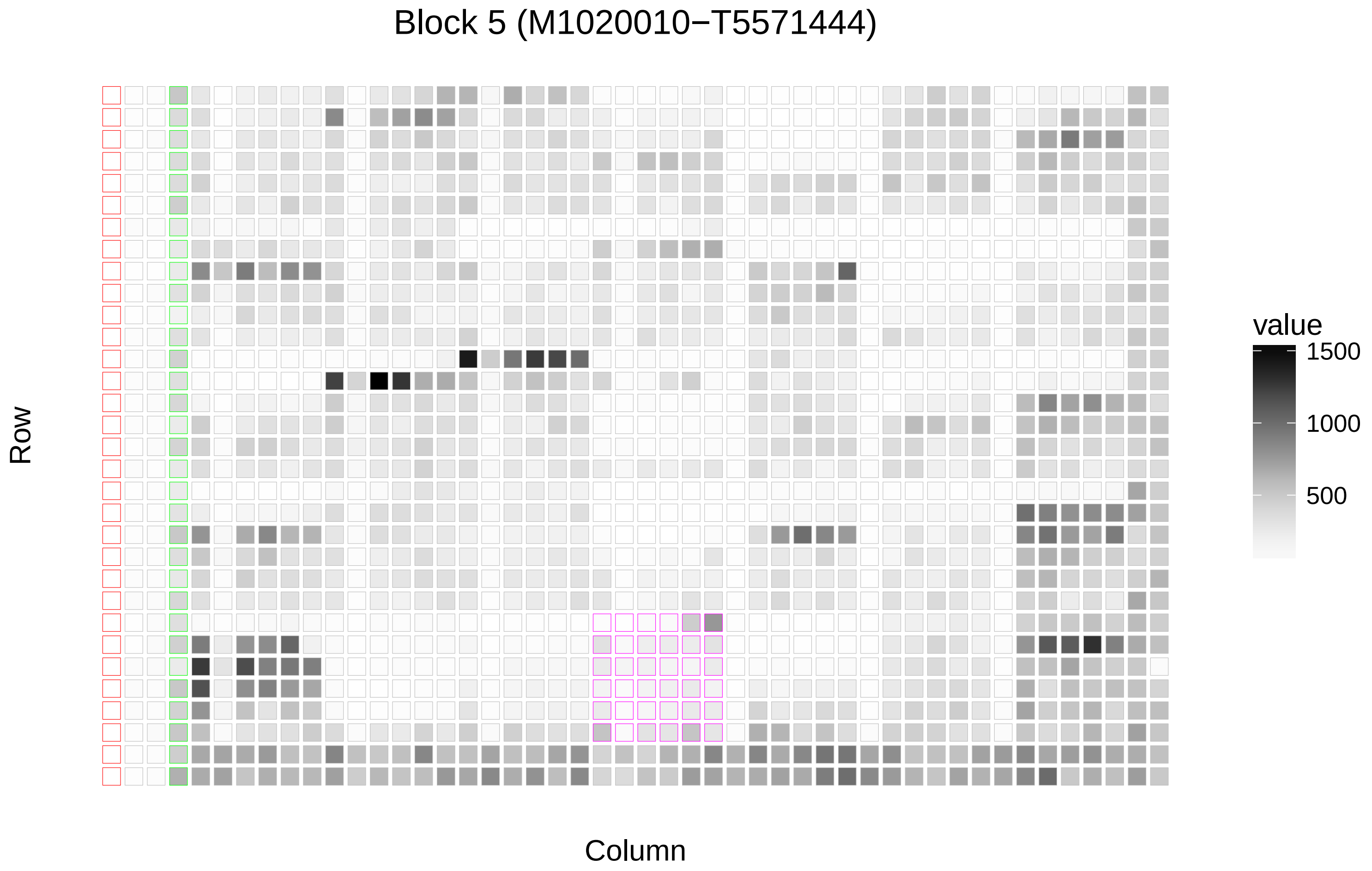

Block 5 (M1020010−T5571444)
Row
Column
value
1500
1000
500

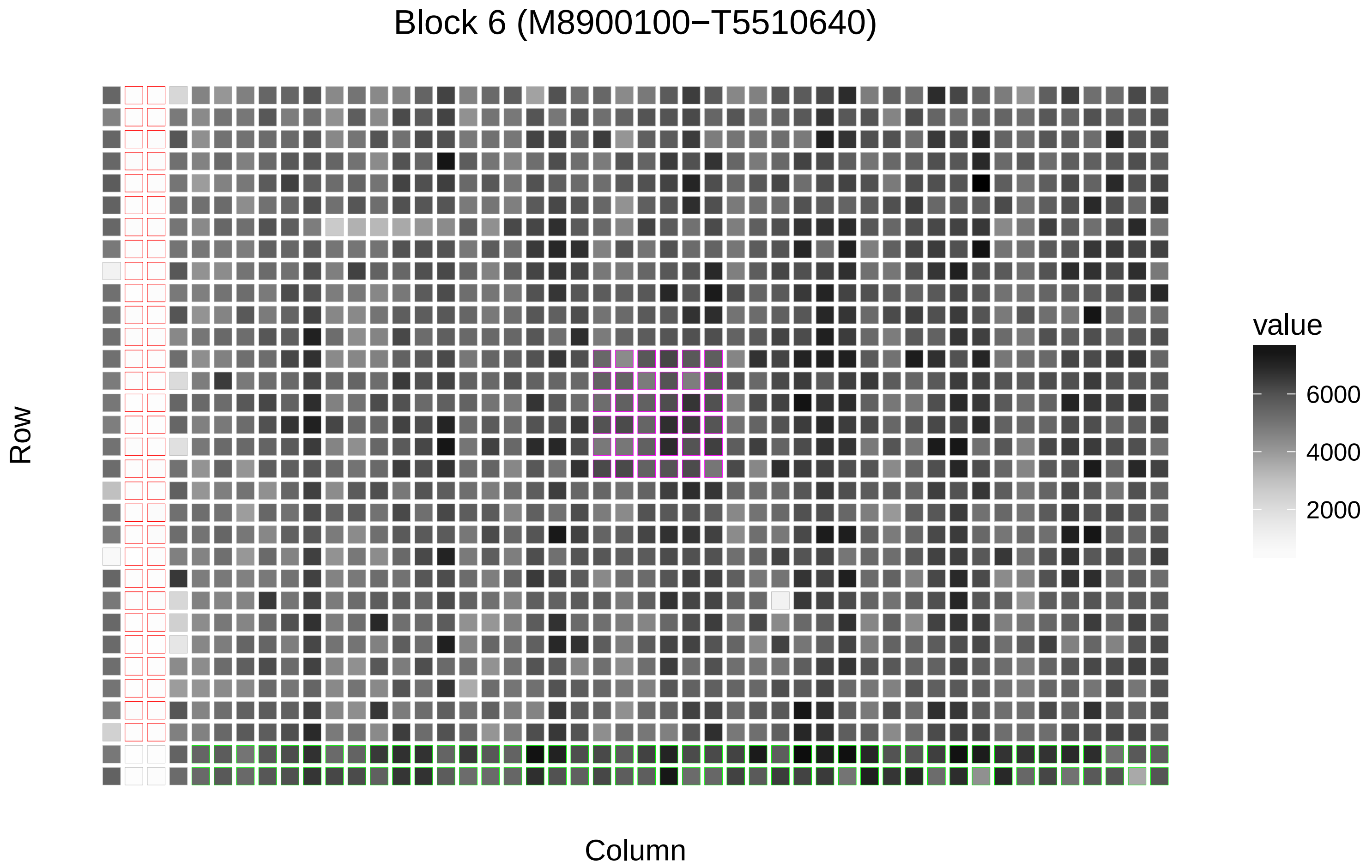
Block 6 (M8900100−T5510640)
Row
Column
value
6000
4000
2000

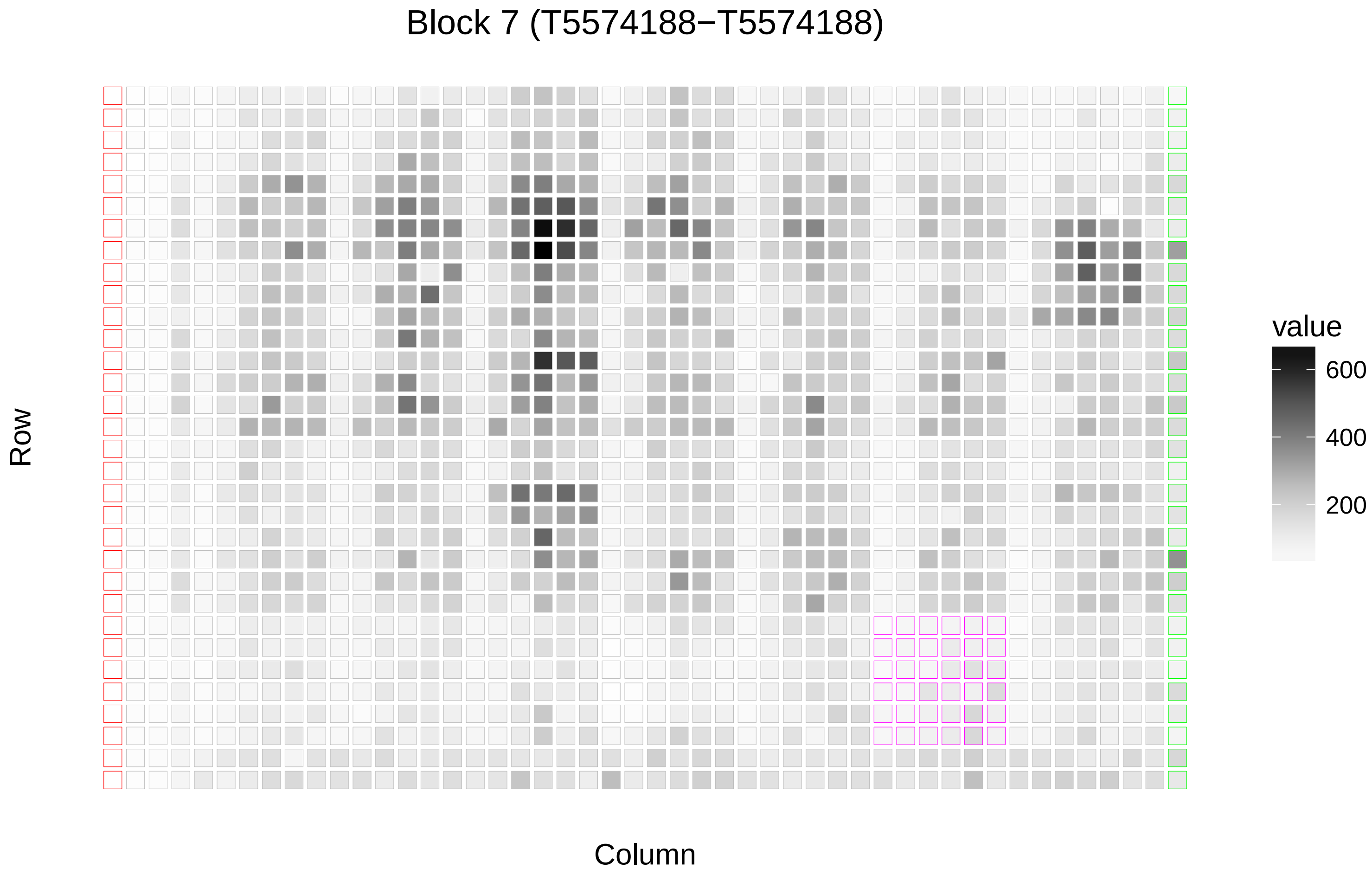

Block 7 (T5574188−T5574188)
Row
Column
value
600
400
200

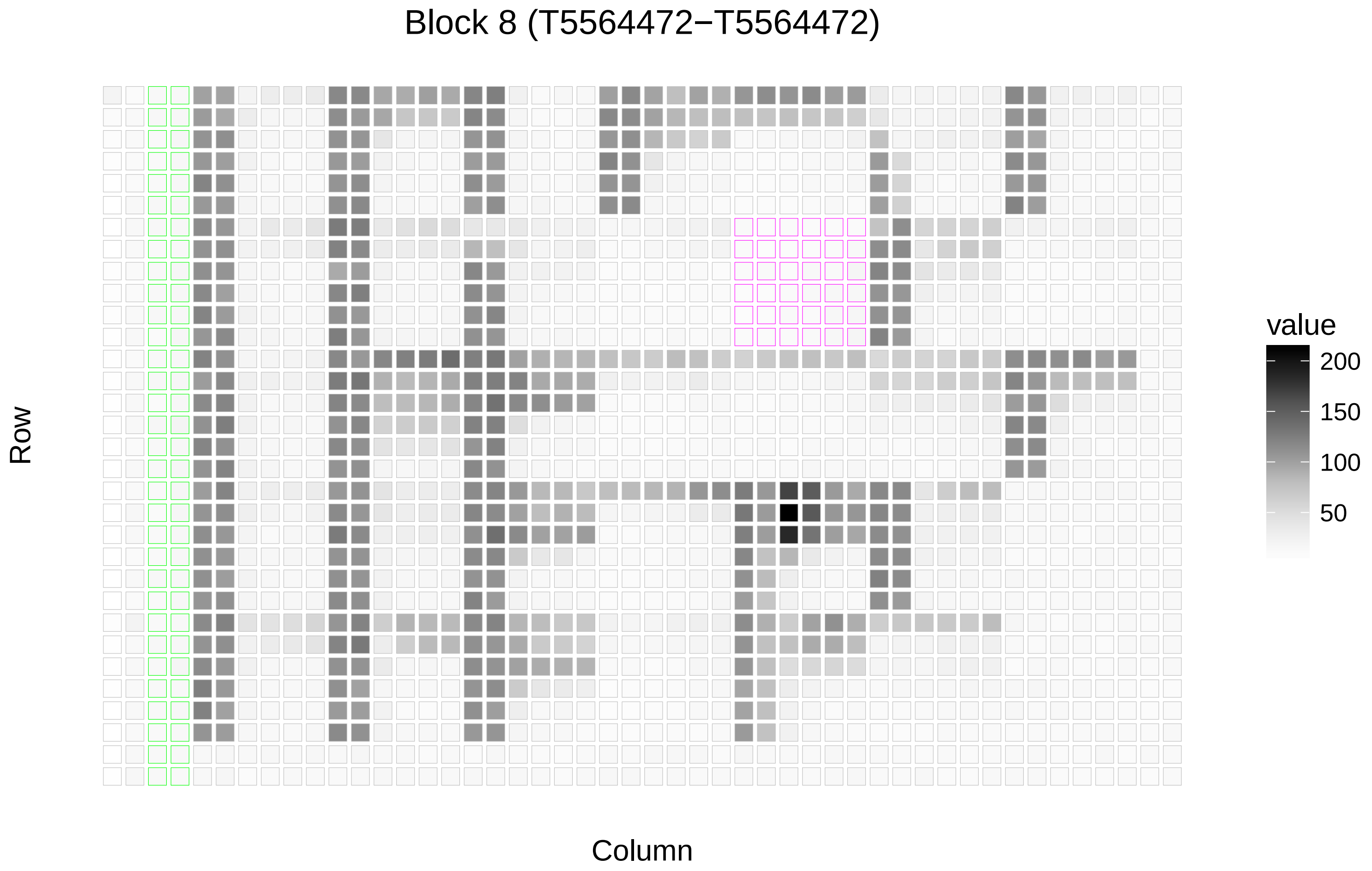

Block 8 (T5564472−T5564472)
Row
Column
value
200
150
100
50

Block 9 (T5606792−T5606792)

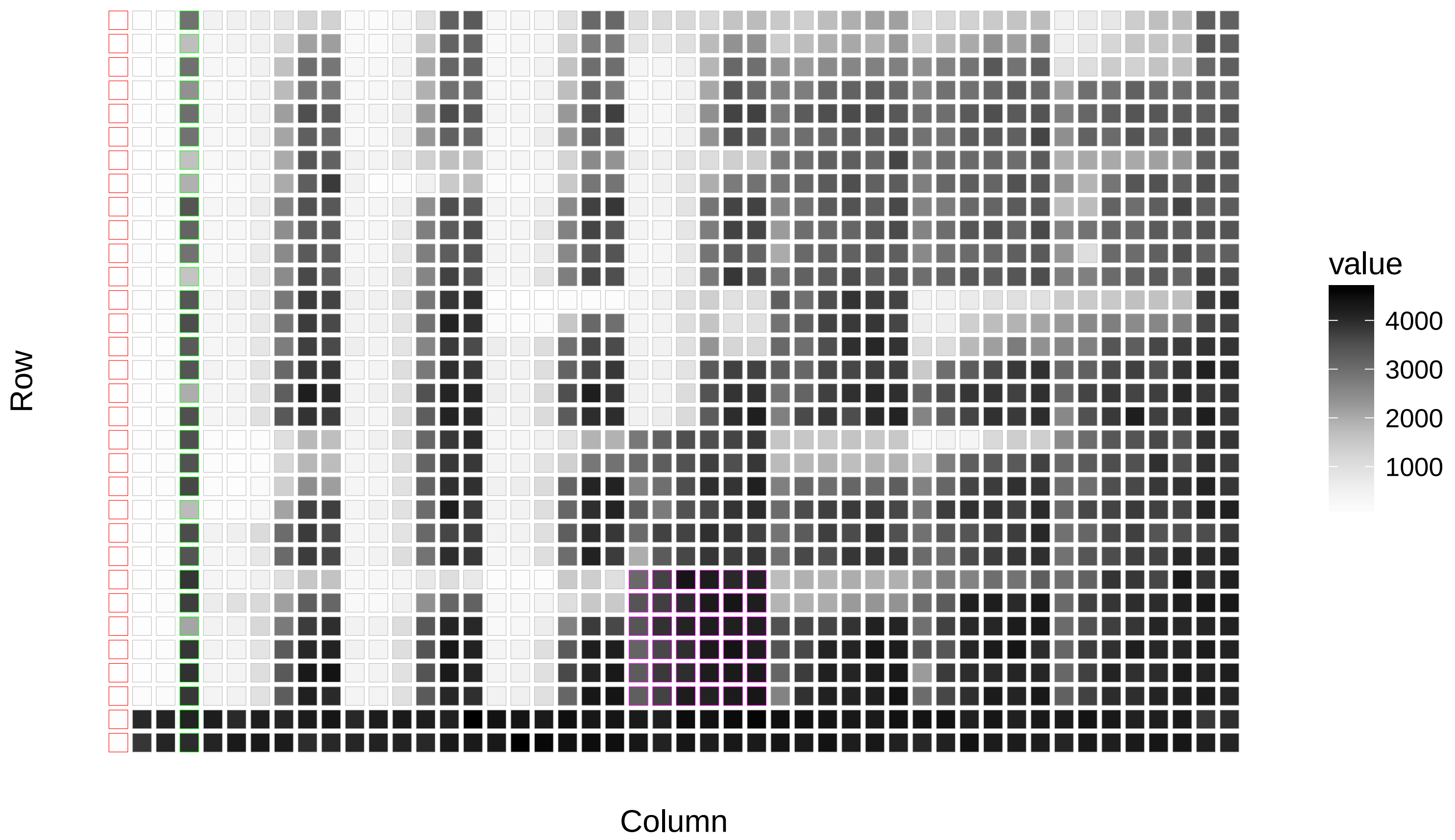

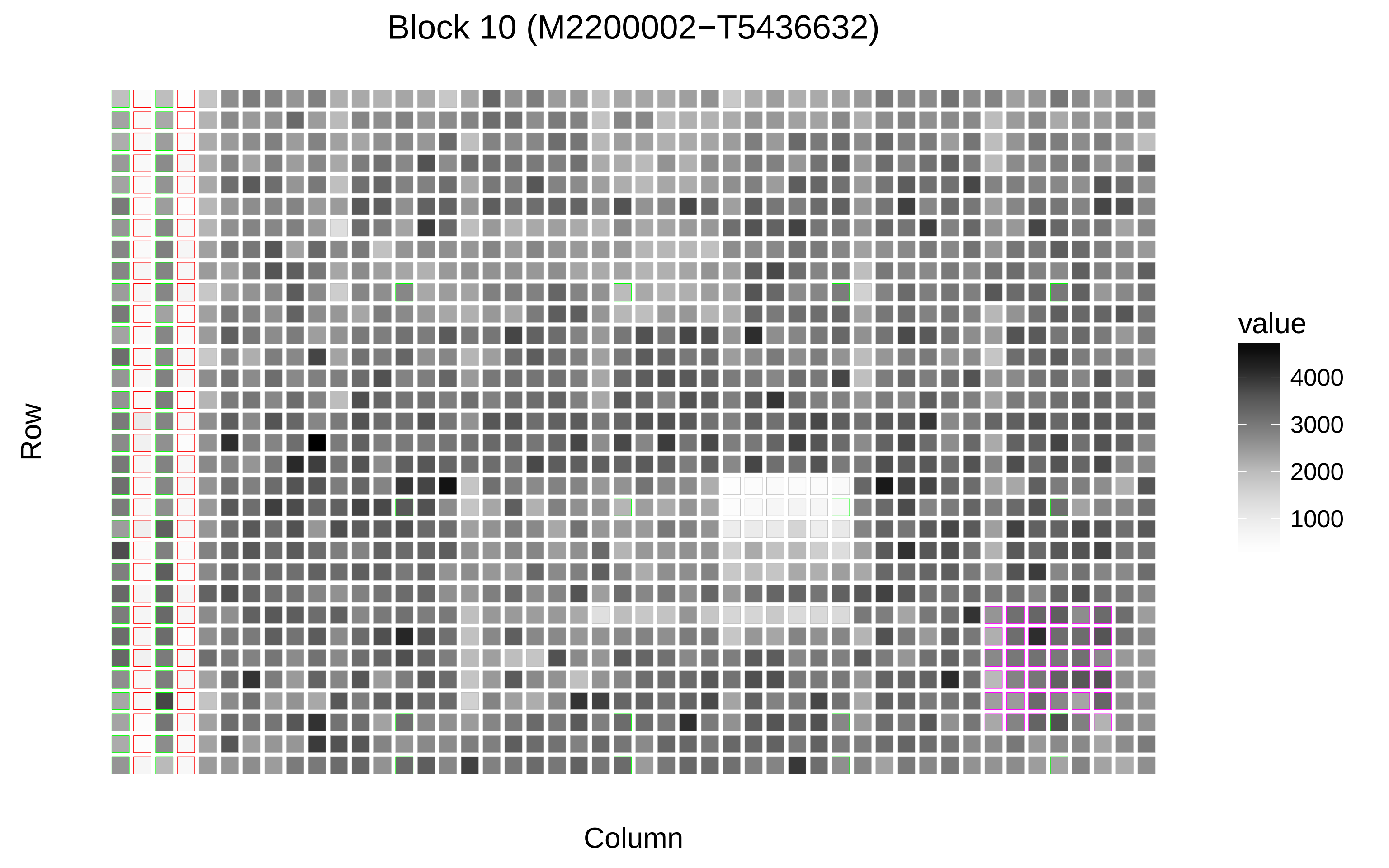
Block 10 (M2200002−T5436632)
Row
Column
value
4000
3000
2000
1000

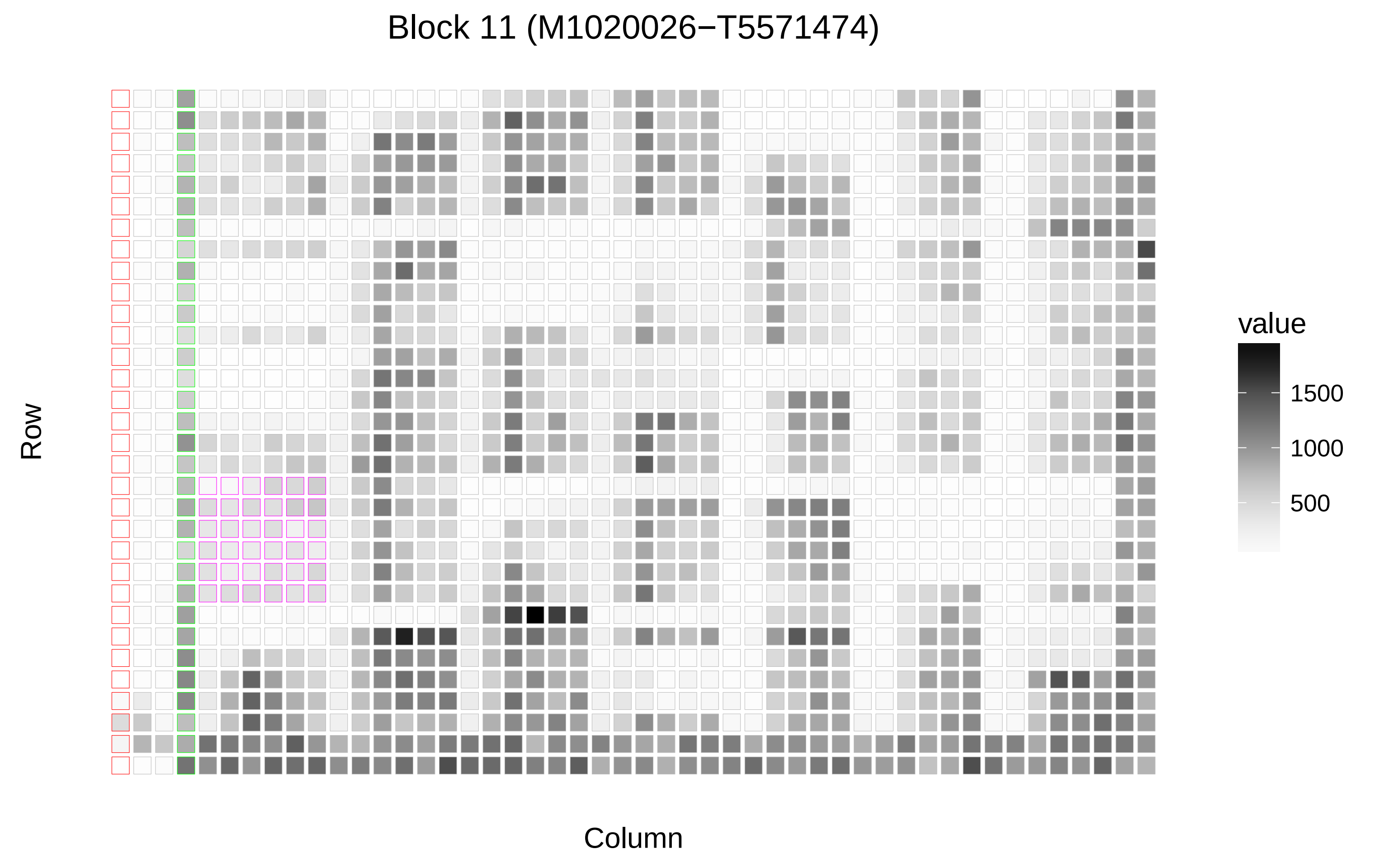

Block 11 (M1020026−T5571474)
Row
Column
value
1500
1000
500

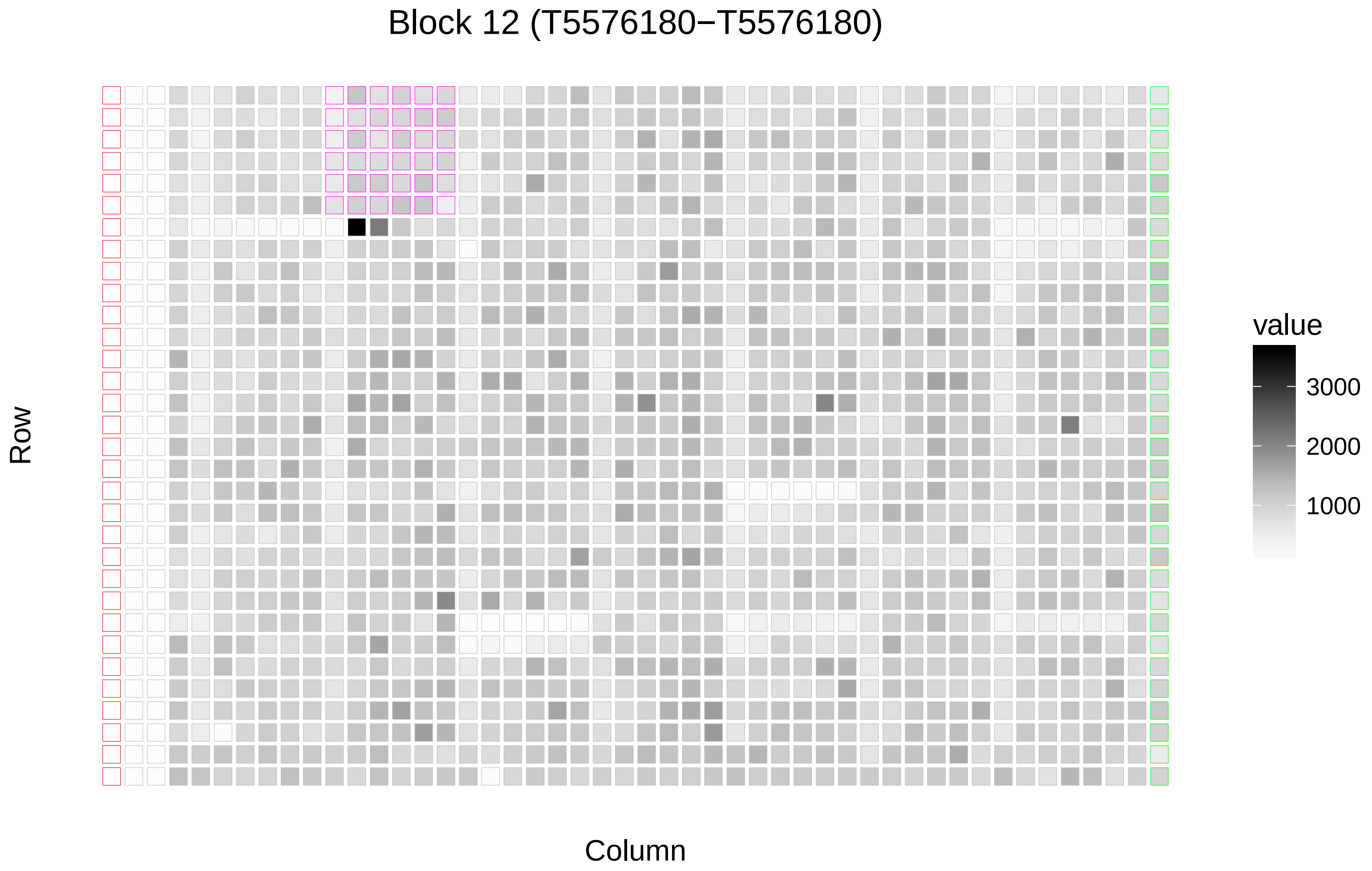
Block 12 (T5576180−T5576180)
Row
Column
value
3000
2000
1000

Block 13 (T5615576–T5615576)

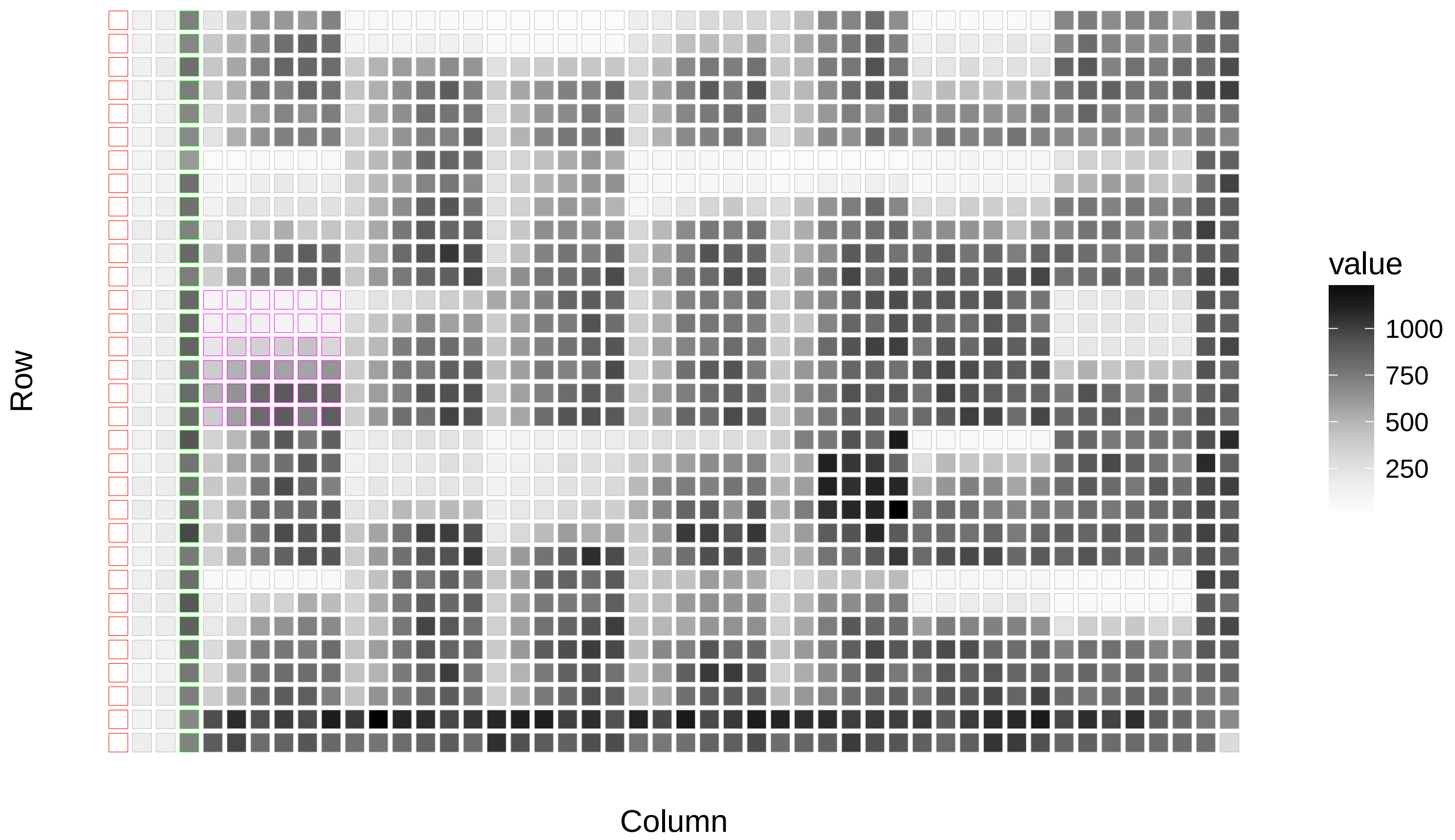

Block 14 (M1020010−T5571444)

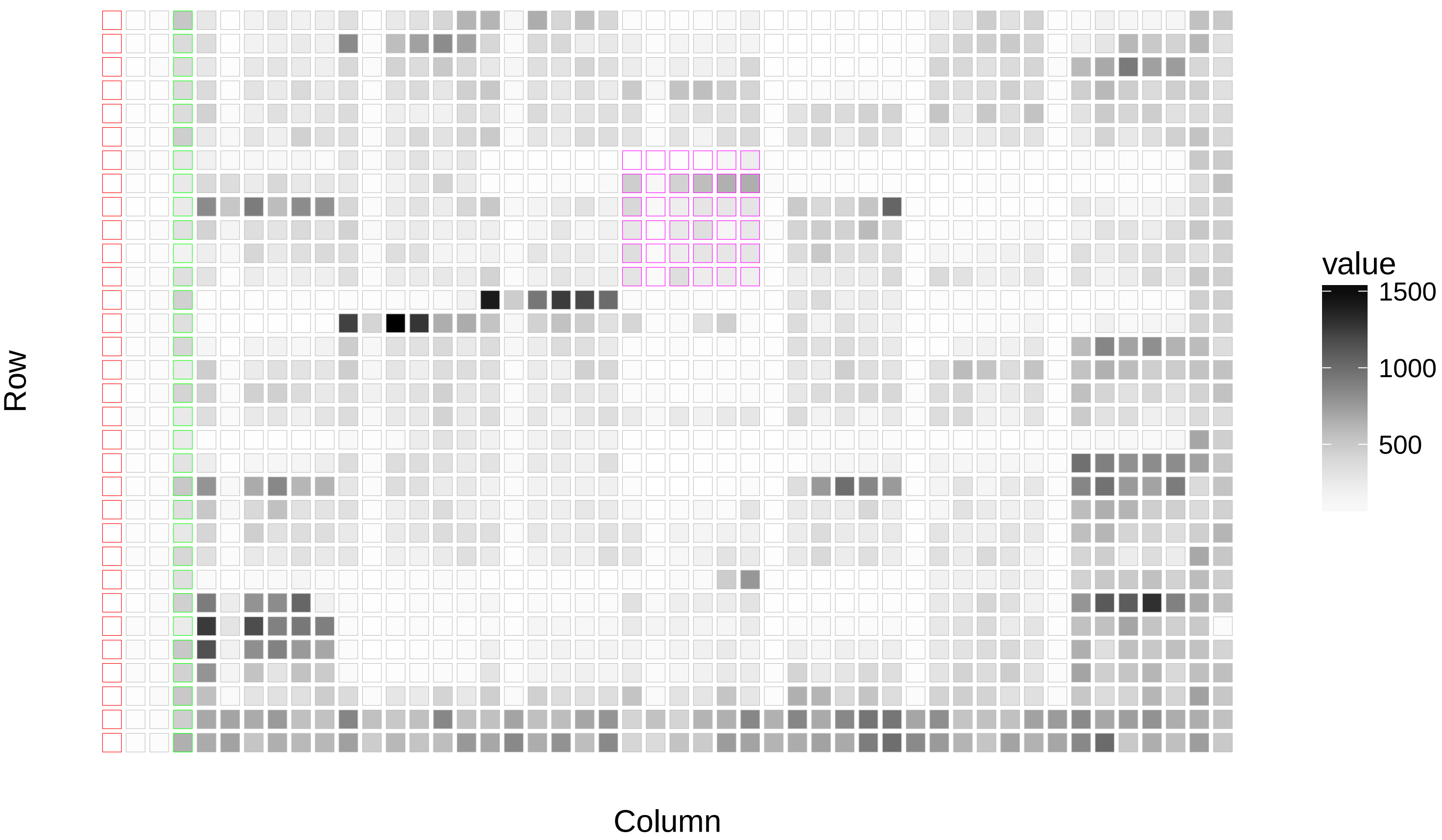

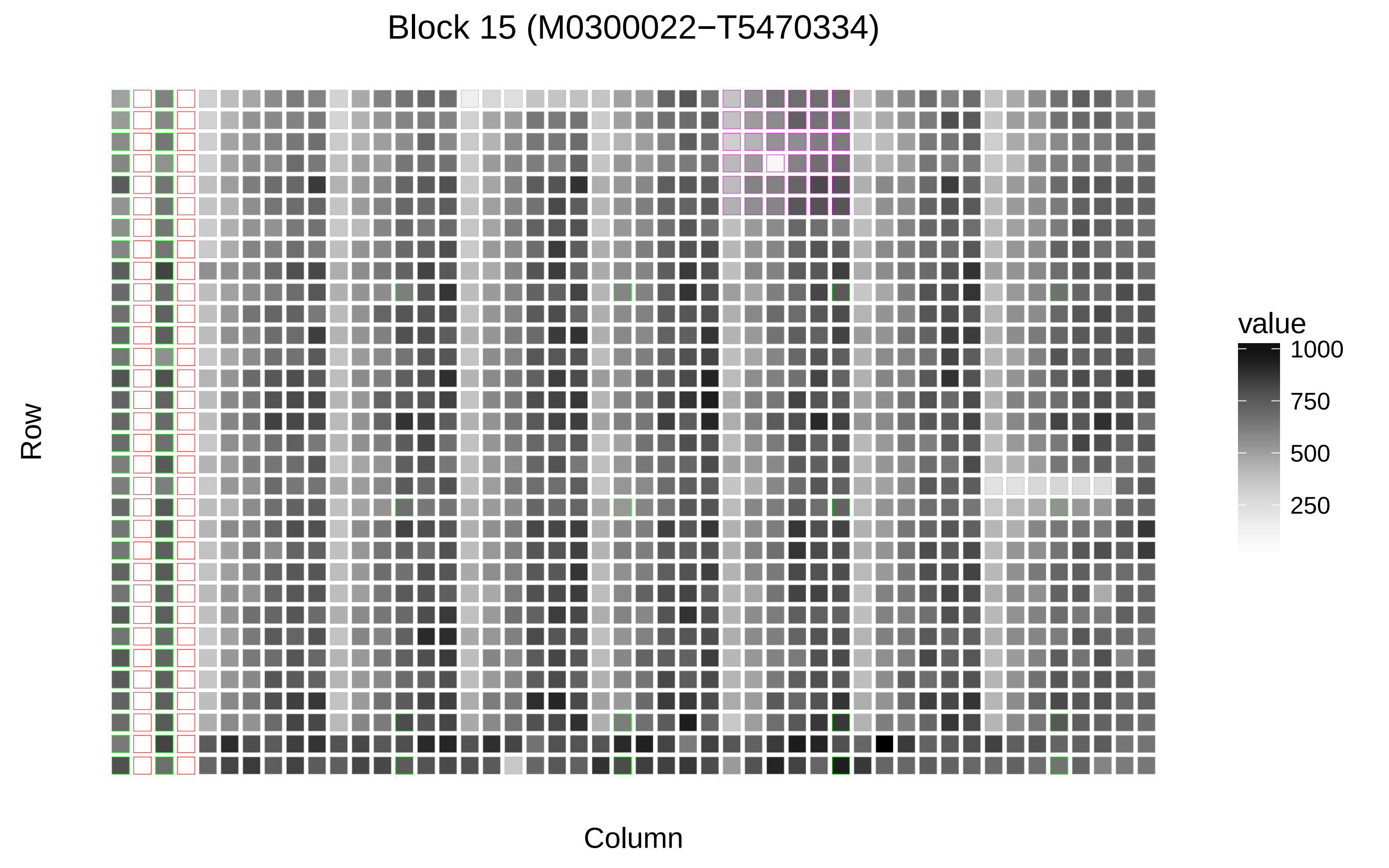
Block 15 (M0300022−T5470334)
Row
Column
value
1000
750
500
250

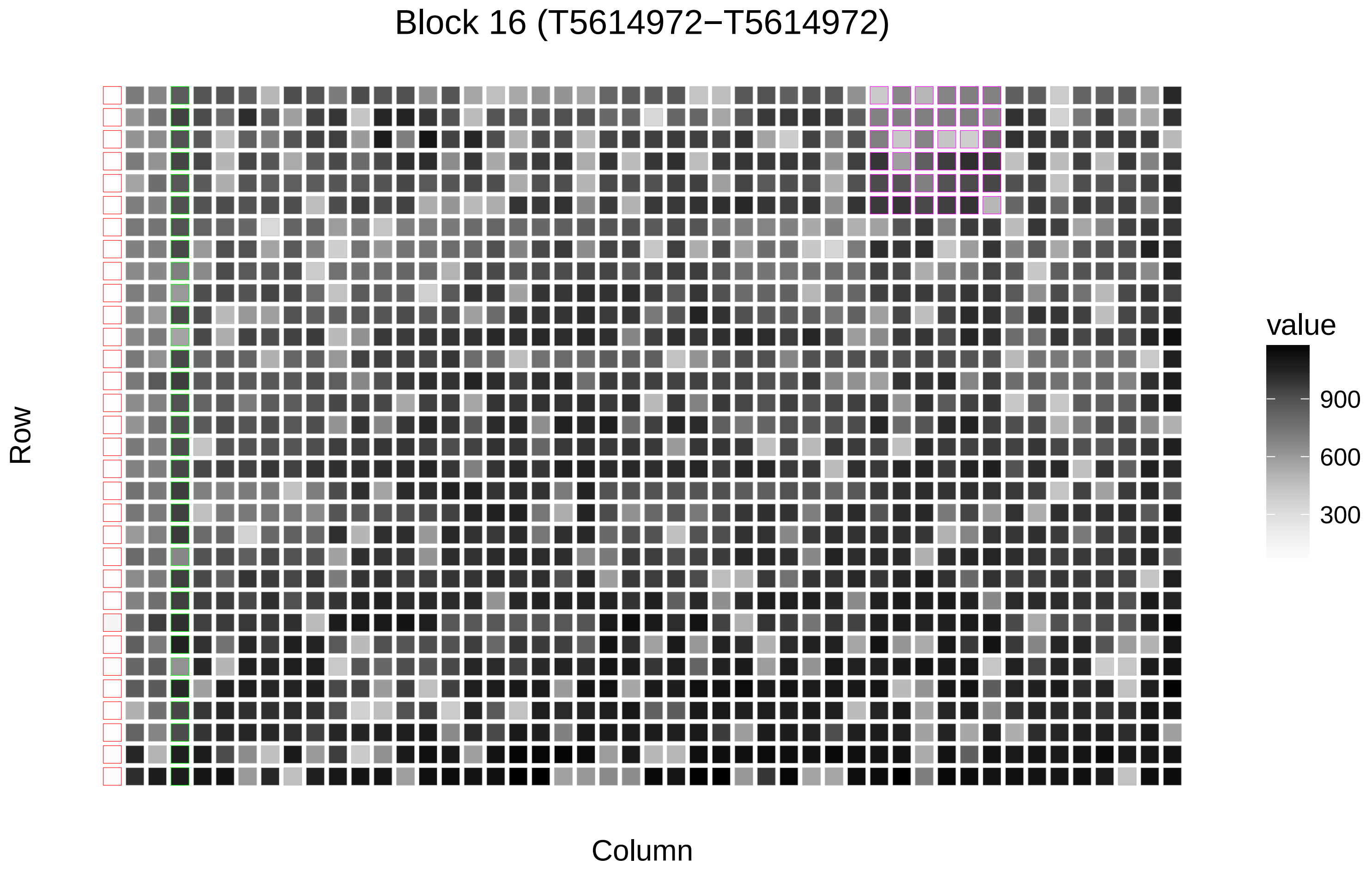
Block 16 (T5614972−T5614972)
Row
Column
value
900
600
300

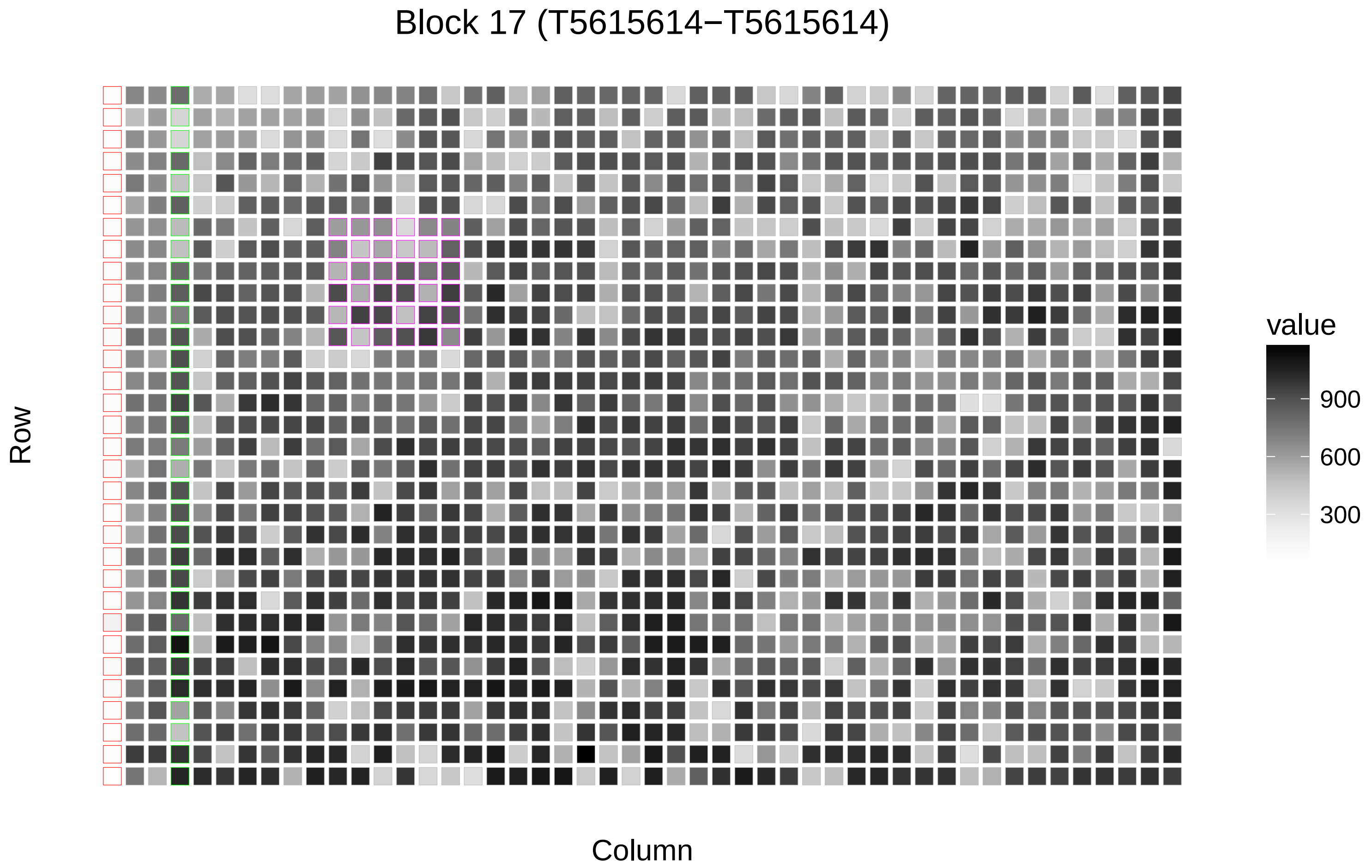
Block 17 (T5615614−T5615614)
Row
Column
value
900
600
300

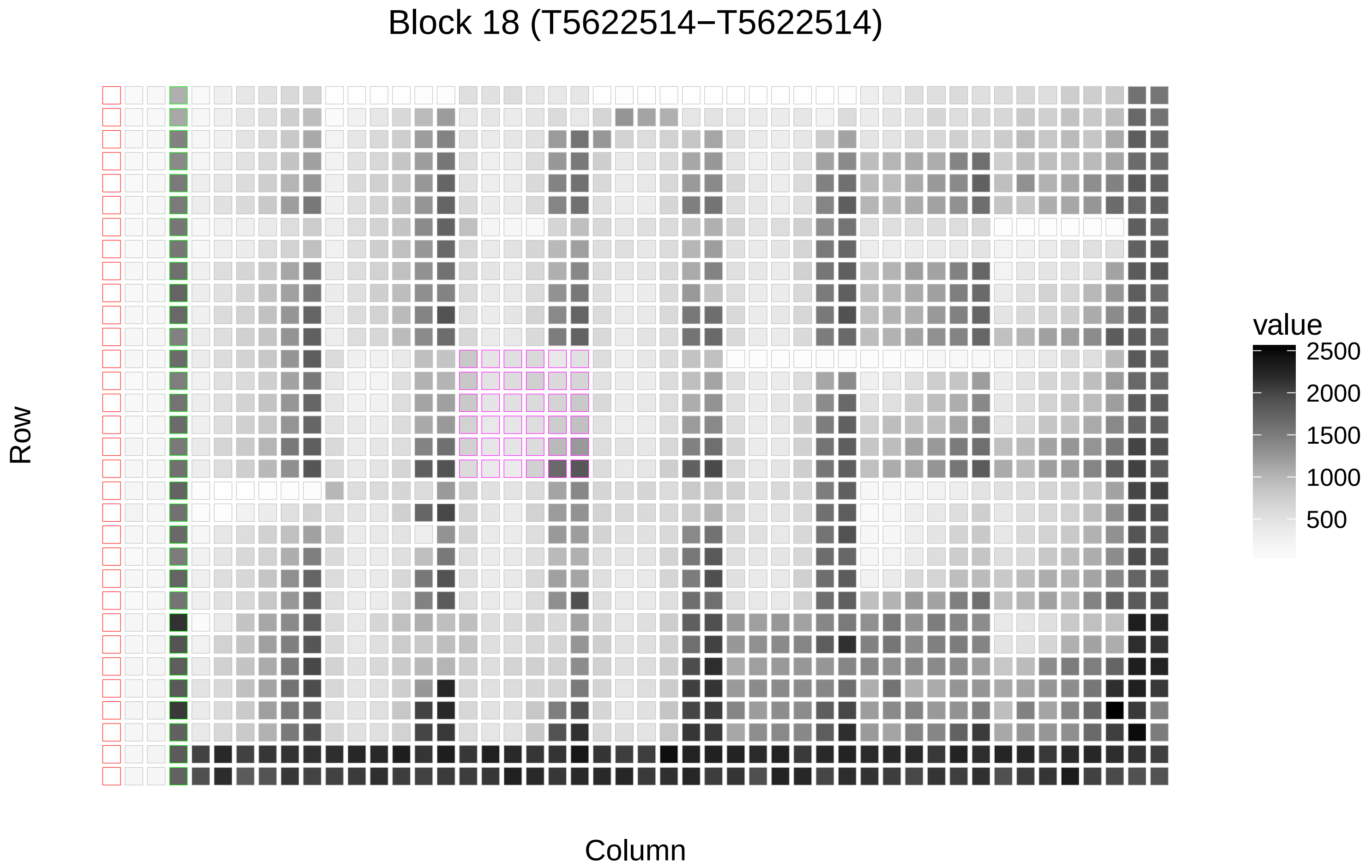
Block 18 (T5622514−T5622514)
Row
Column
value
2500
2000
1500
1000
500

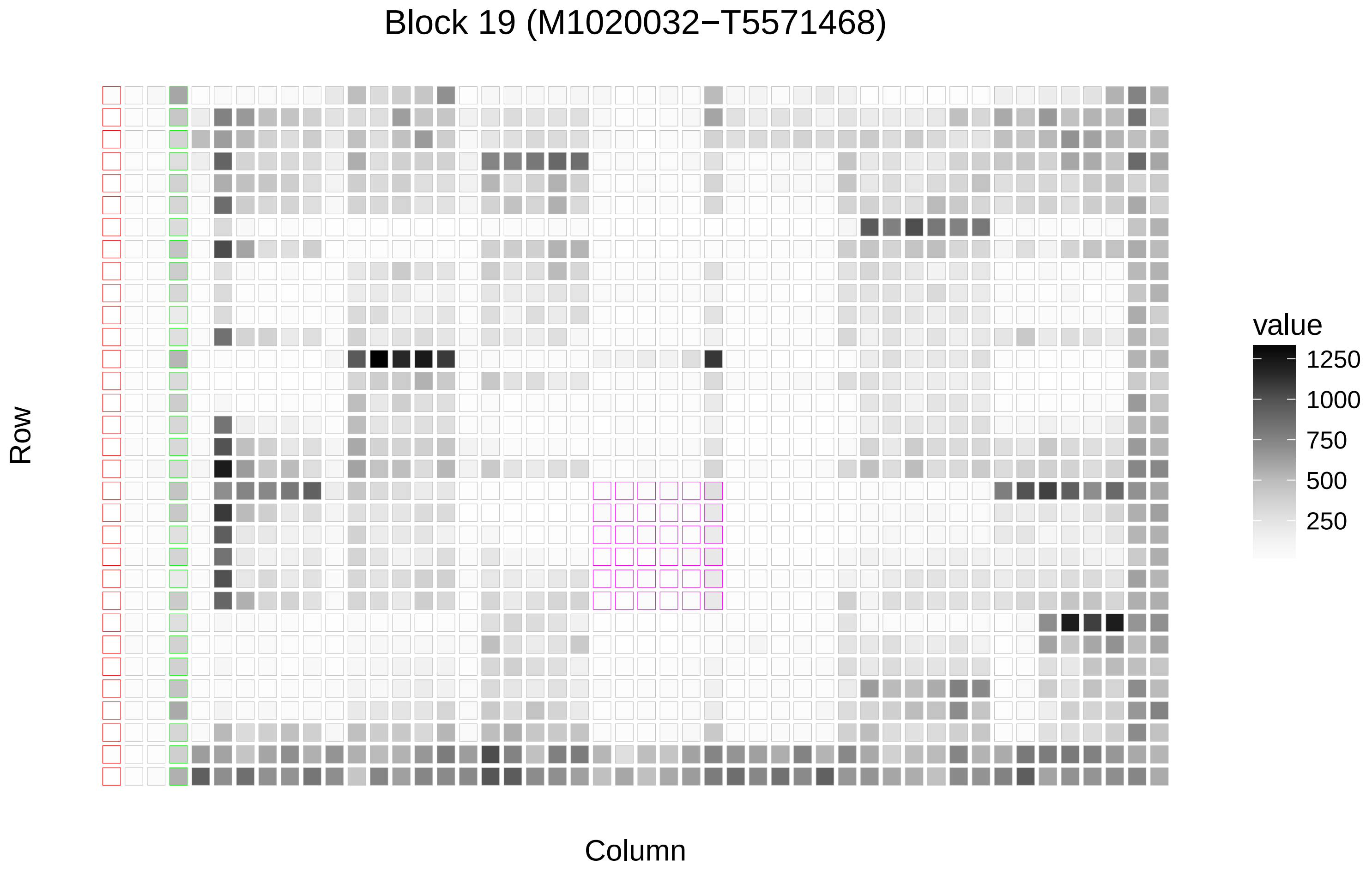
Block 19 (M1020032−T5571468)
Row
Column
value
1250
1000
750
500
250

Block 20 (M2200022−T5436604)

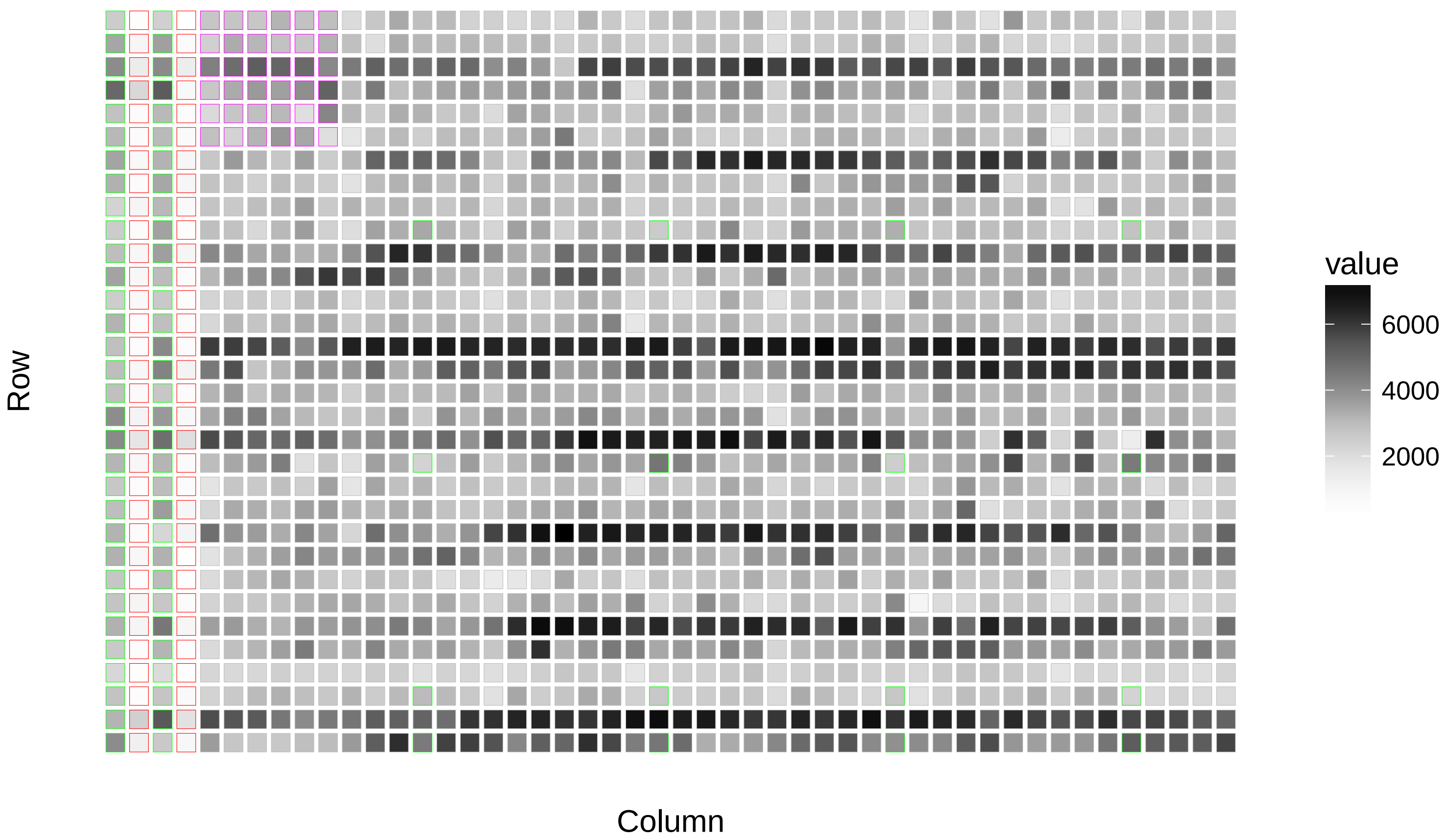

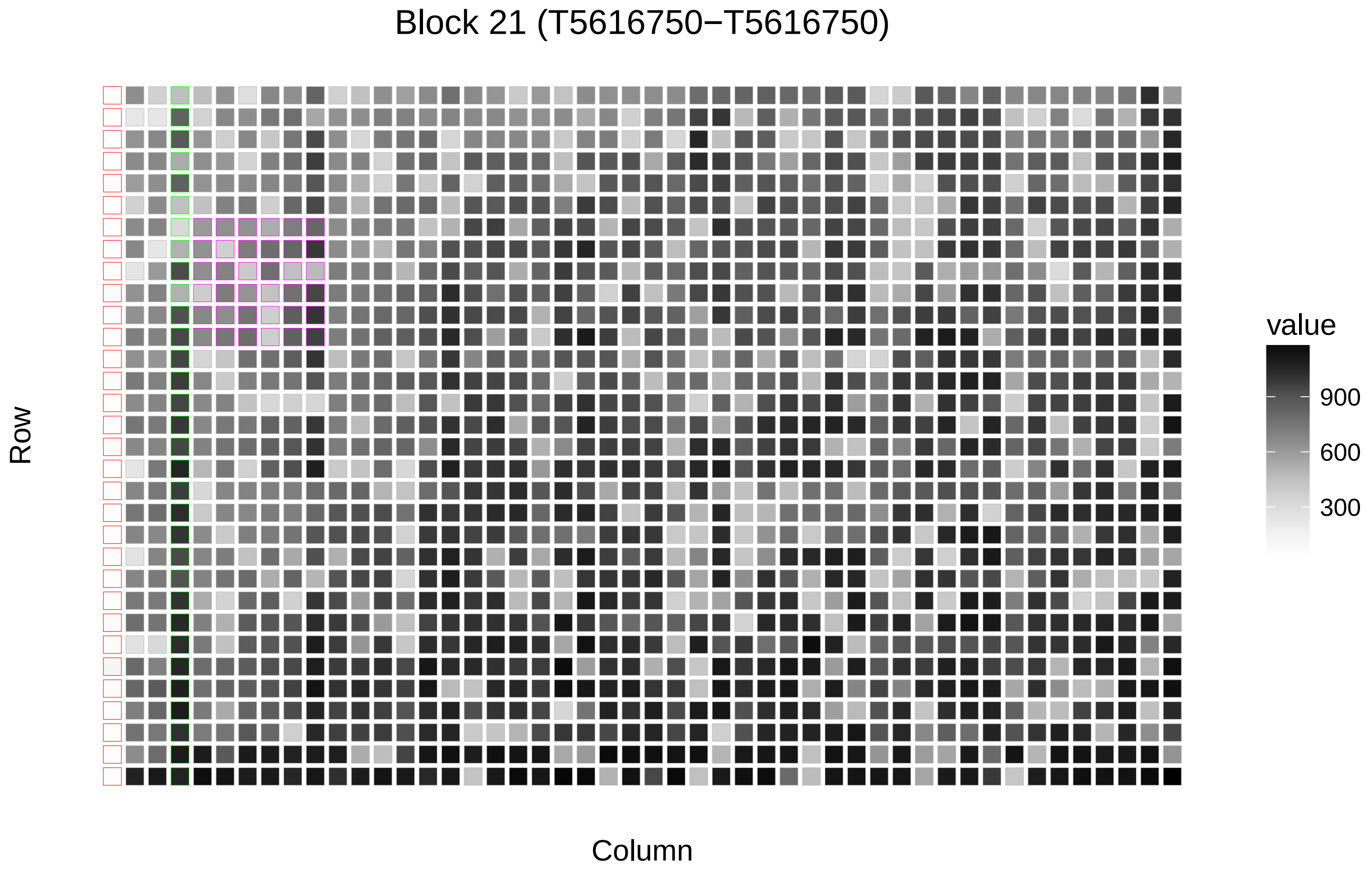
Block 21 (T5616750−T5616750)
Row
Column
value
900
600
300

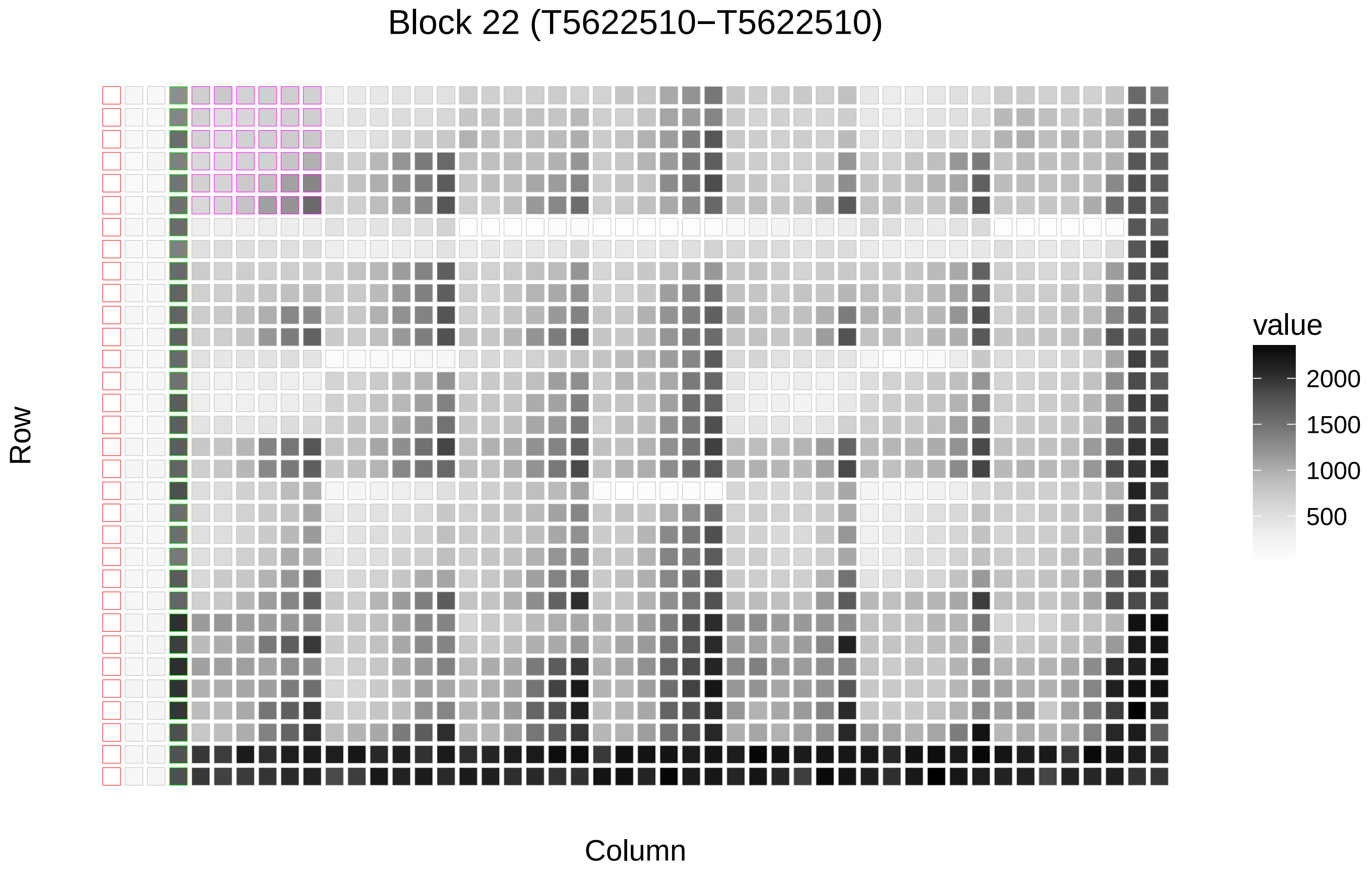

Block 22 (T5622510−T5622510)
Row
Column
value
2000
1500
1000
500

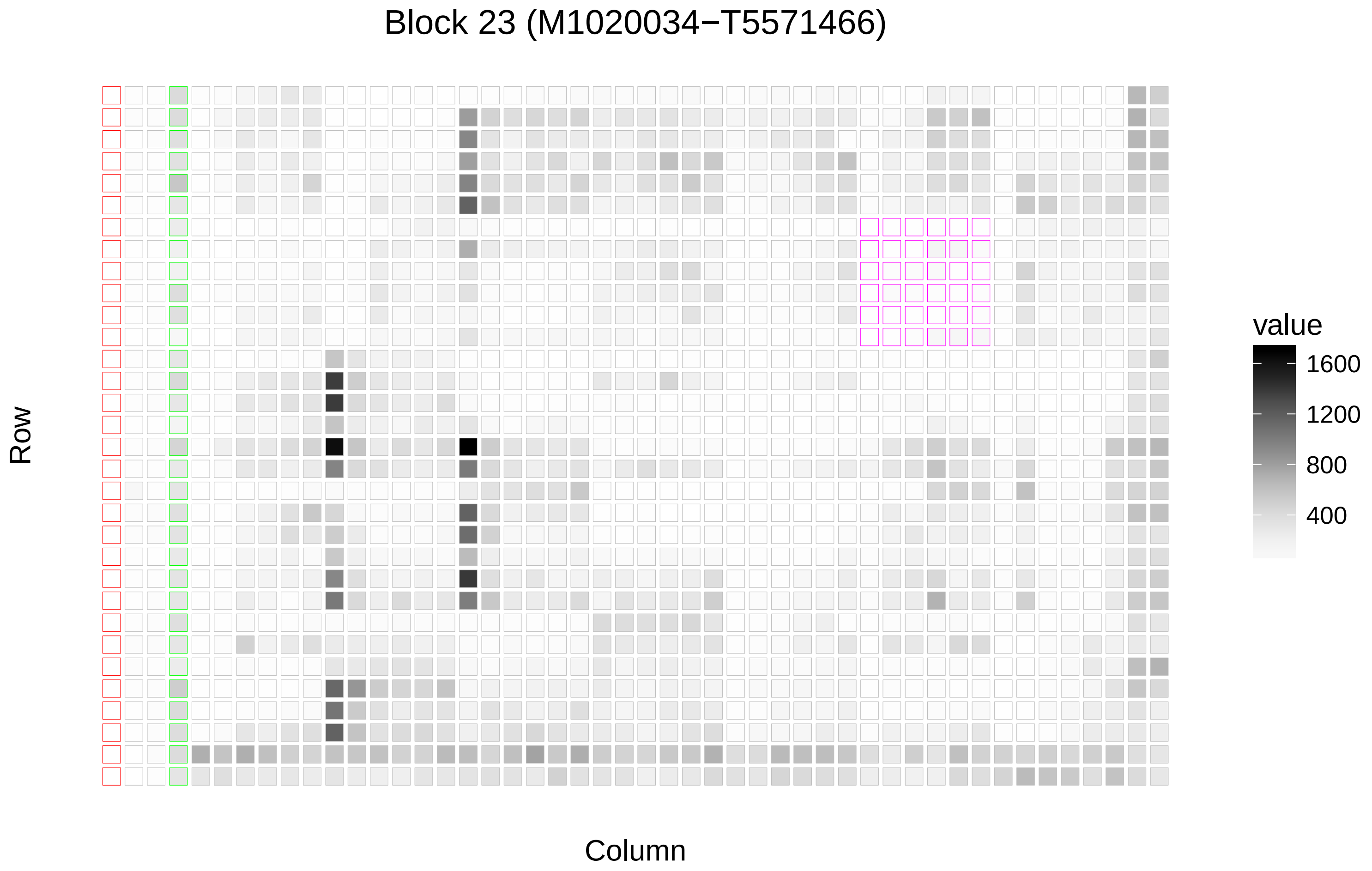

Block 23 (M1020034−T5571466)
Row
Column
value
1600
1200
800
400

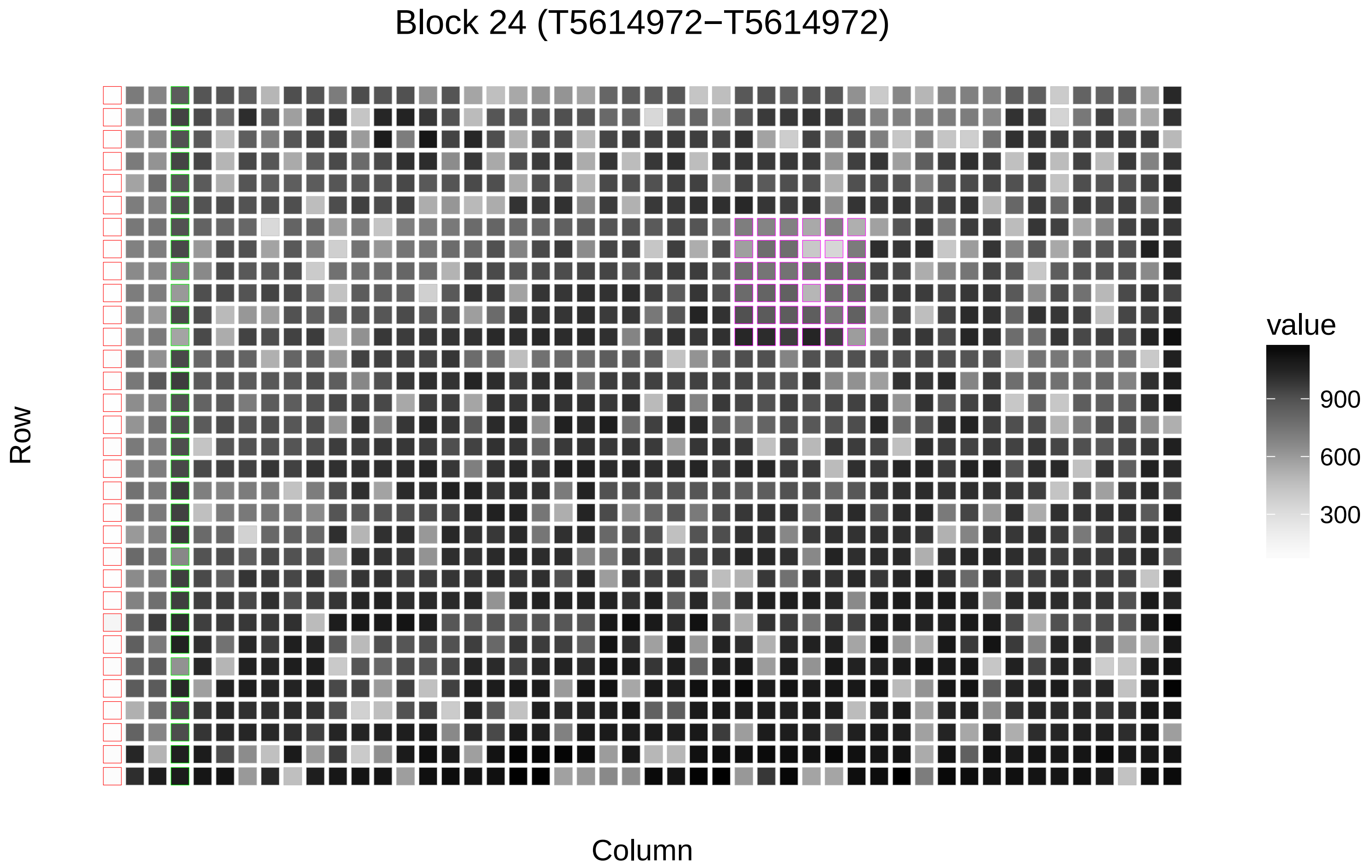

Block 24 (T5614972−T5614972)
Row
Column
value
900
600
300

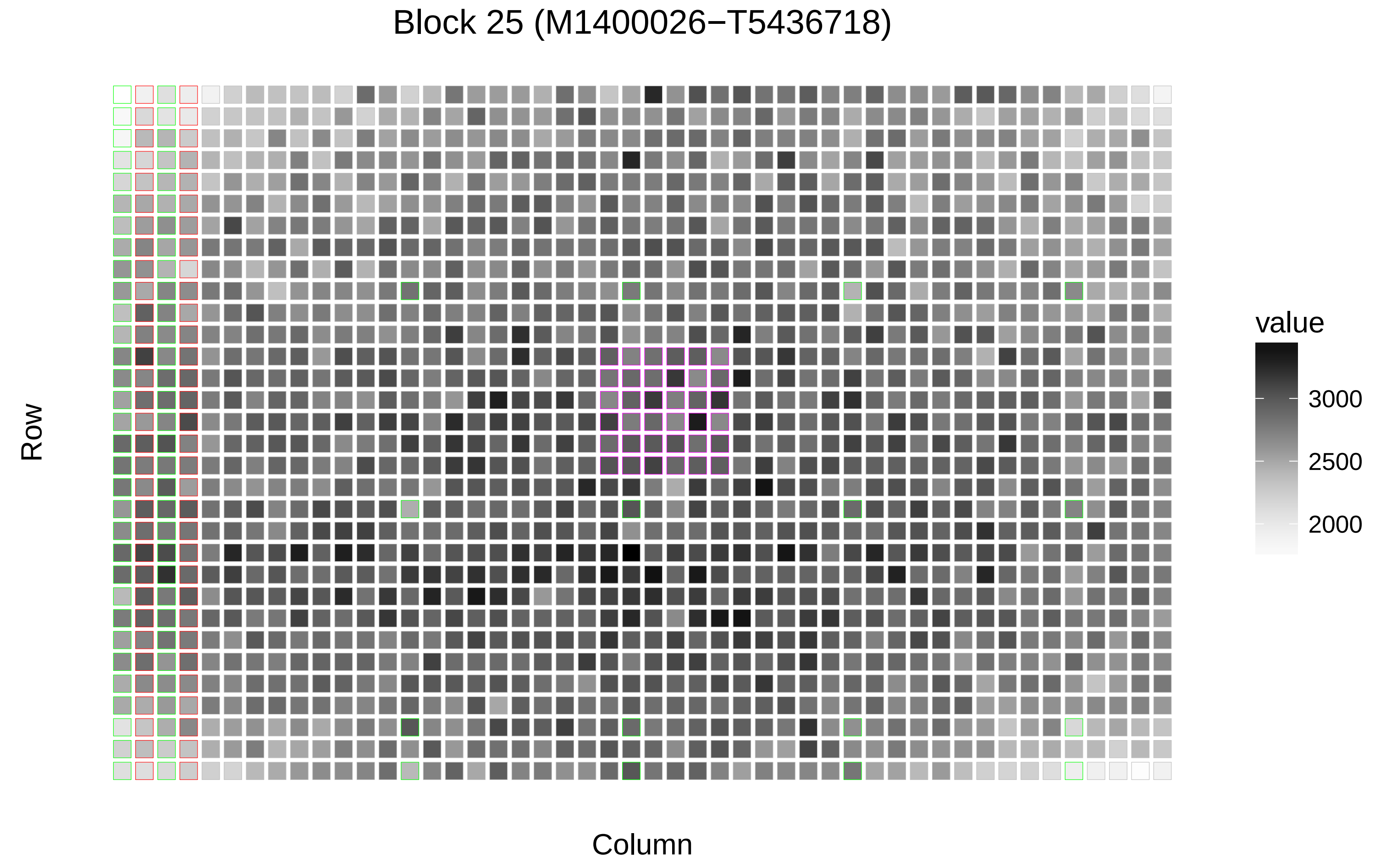

Block 25 (M1400026−T5436718)
Row
Column
value
3000
2500
2000

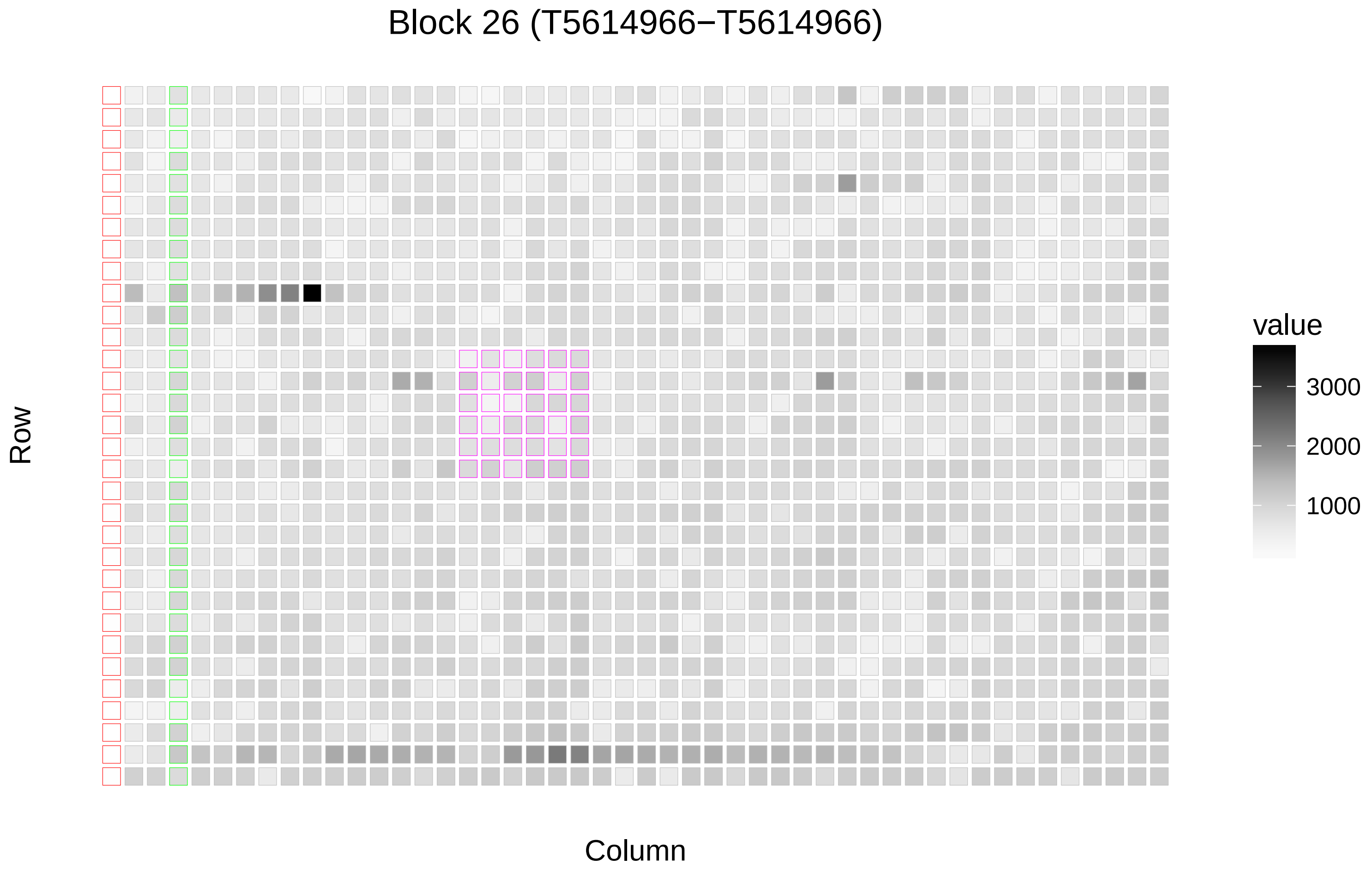
Block 26 (T5614966−T5614966)
Row
Column
value
3000
2000
1000

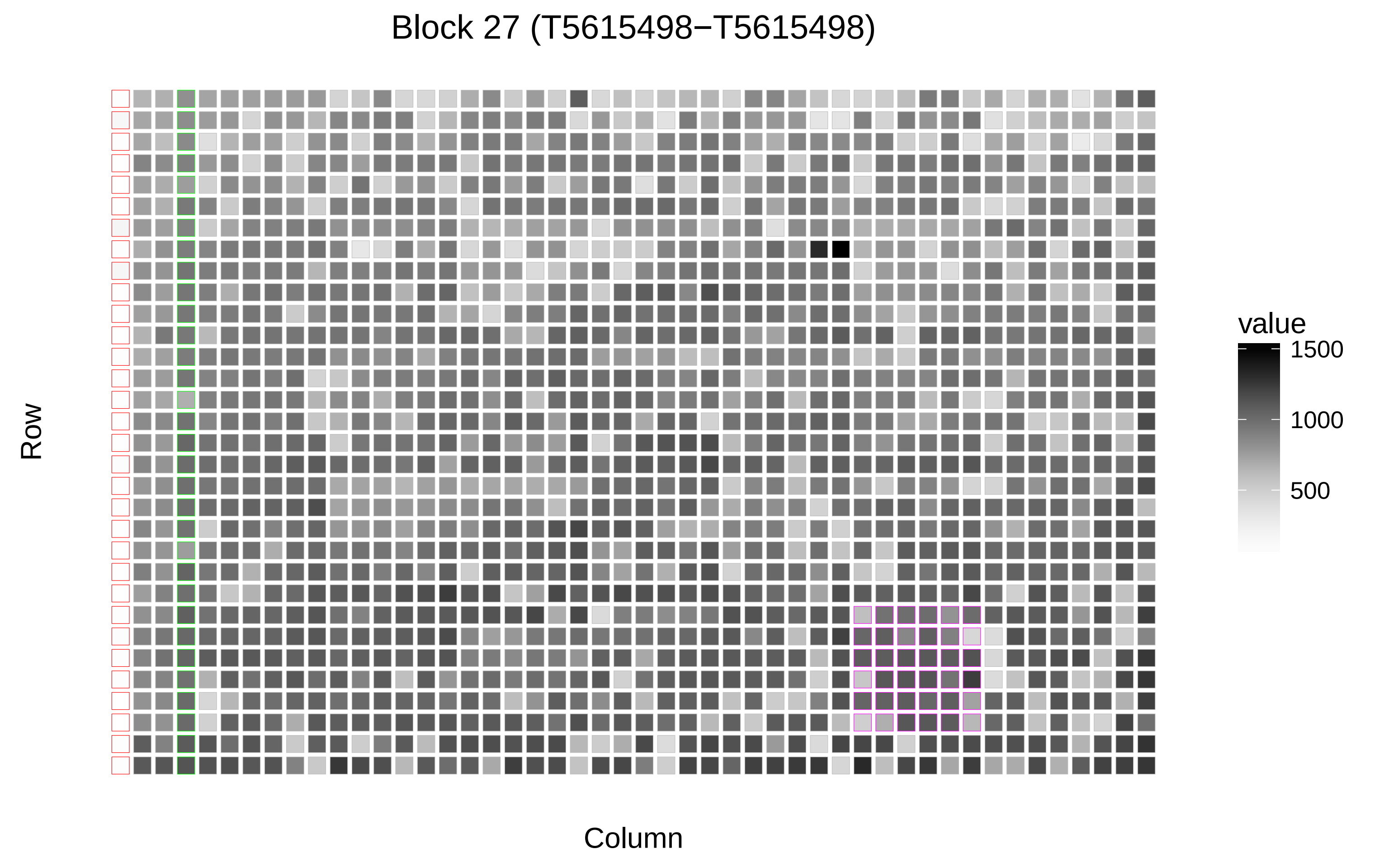
Block 27 (T5615498−T5615498)
Row
Column
value
1500
1000
500

Block 28 (M1020024−T5571454)

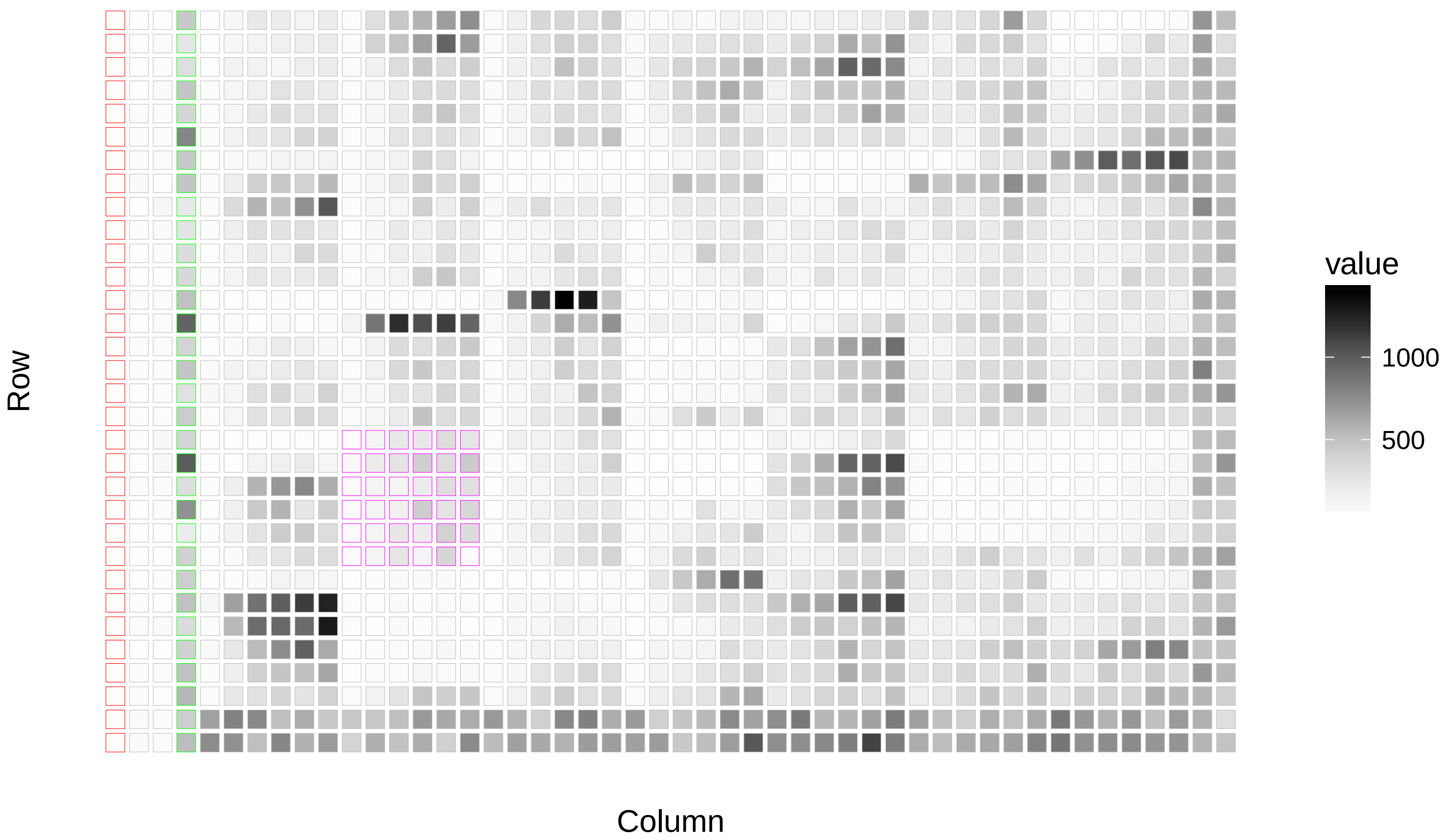

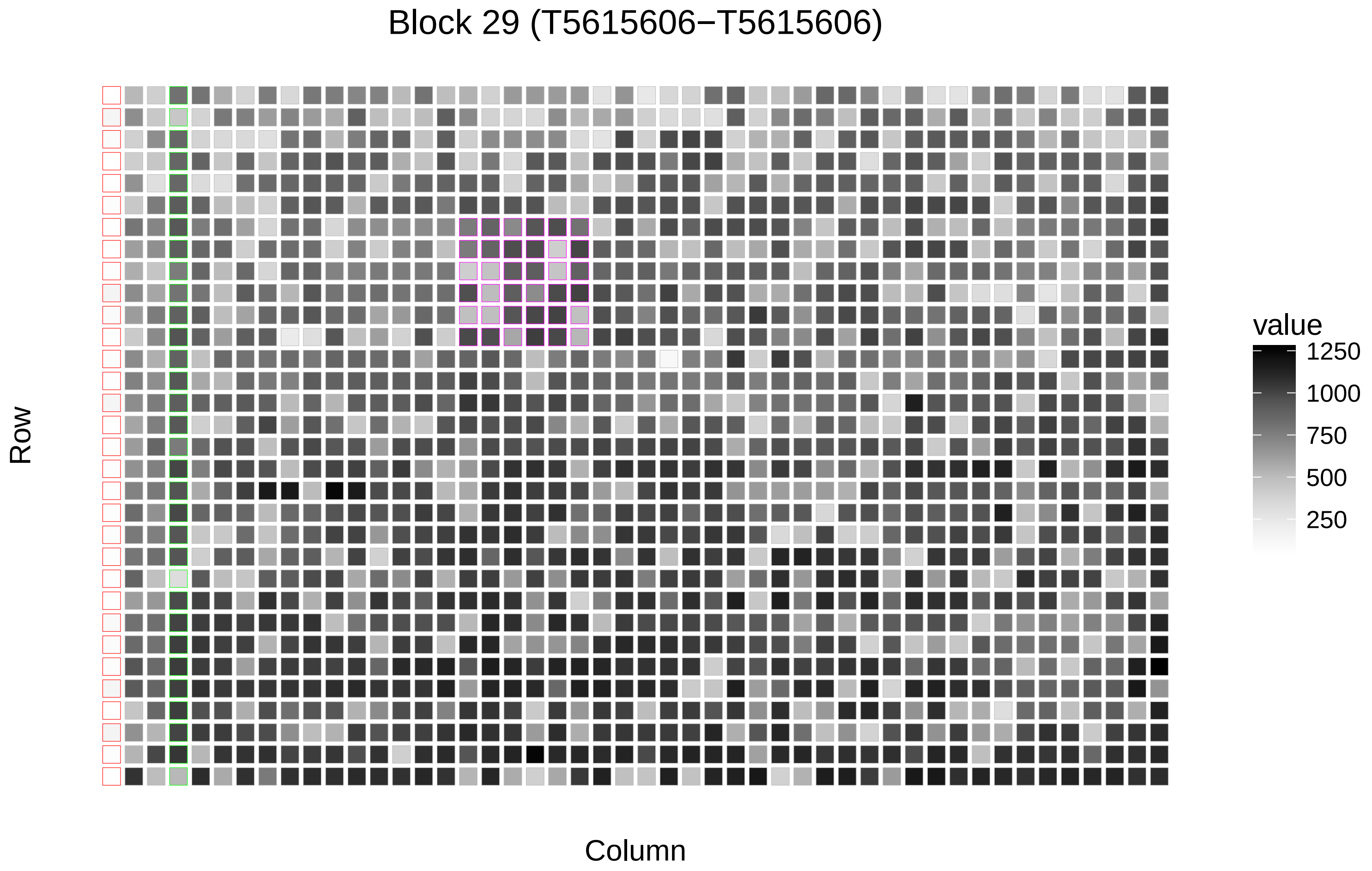
Block 29 (T5615606−T5615606)
Row
Column
value
1250
1000
750
500
250

Block 30 (T5614964−T5614964)

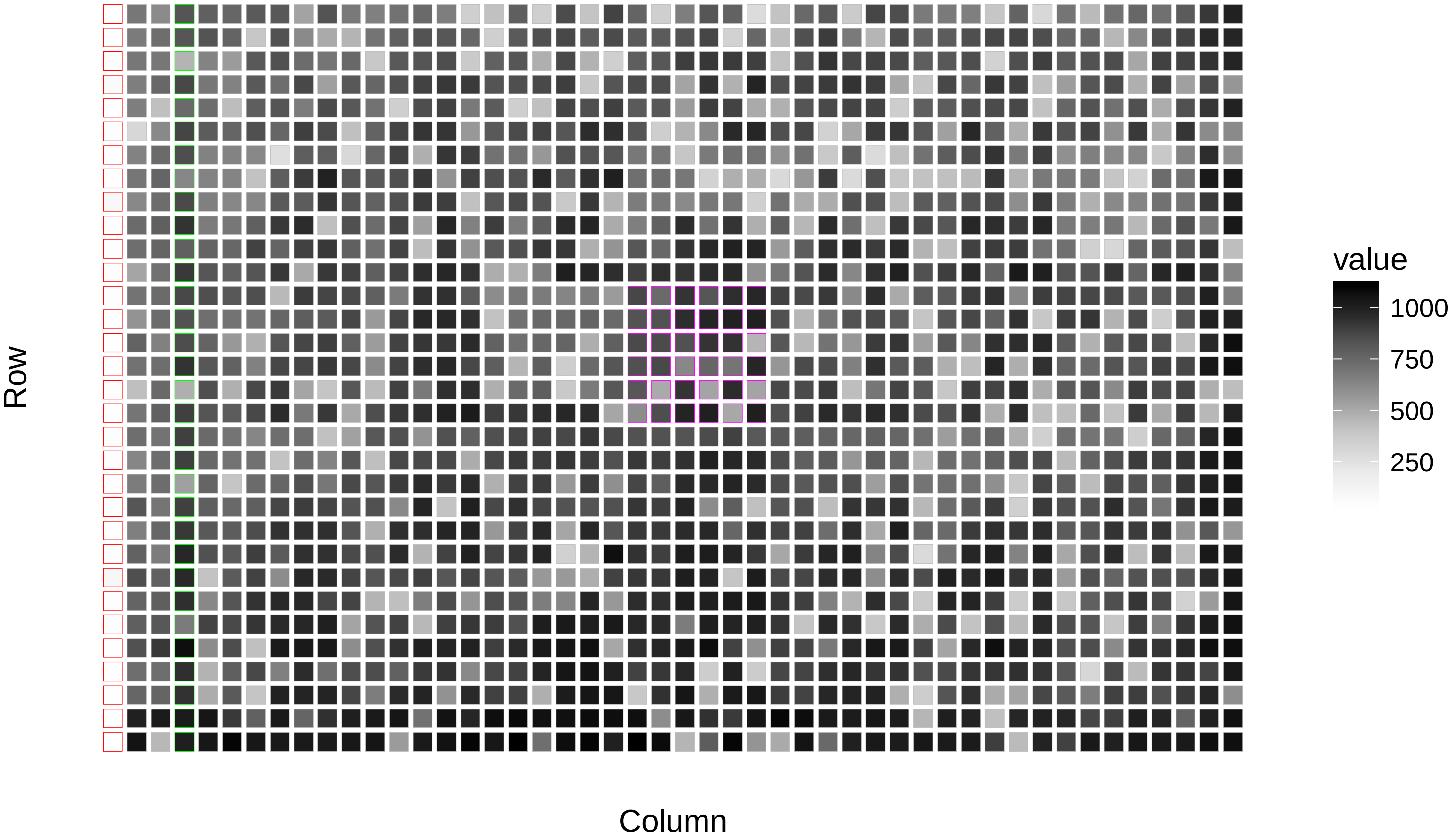

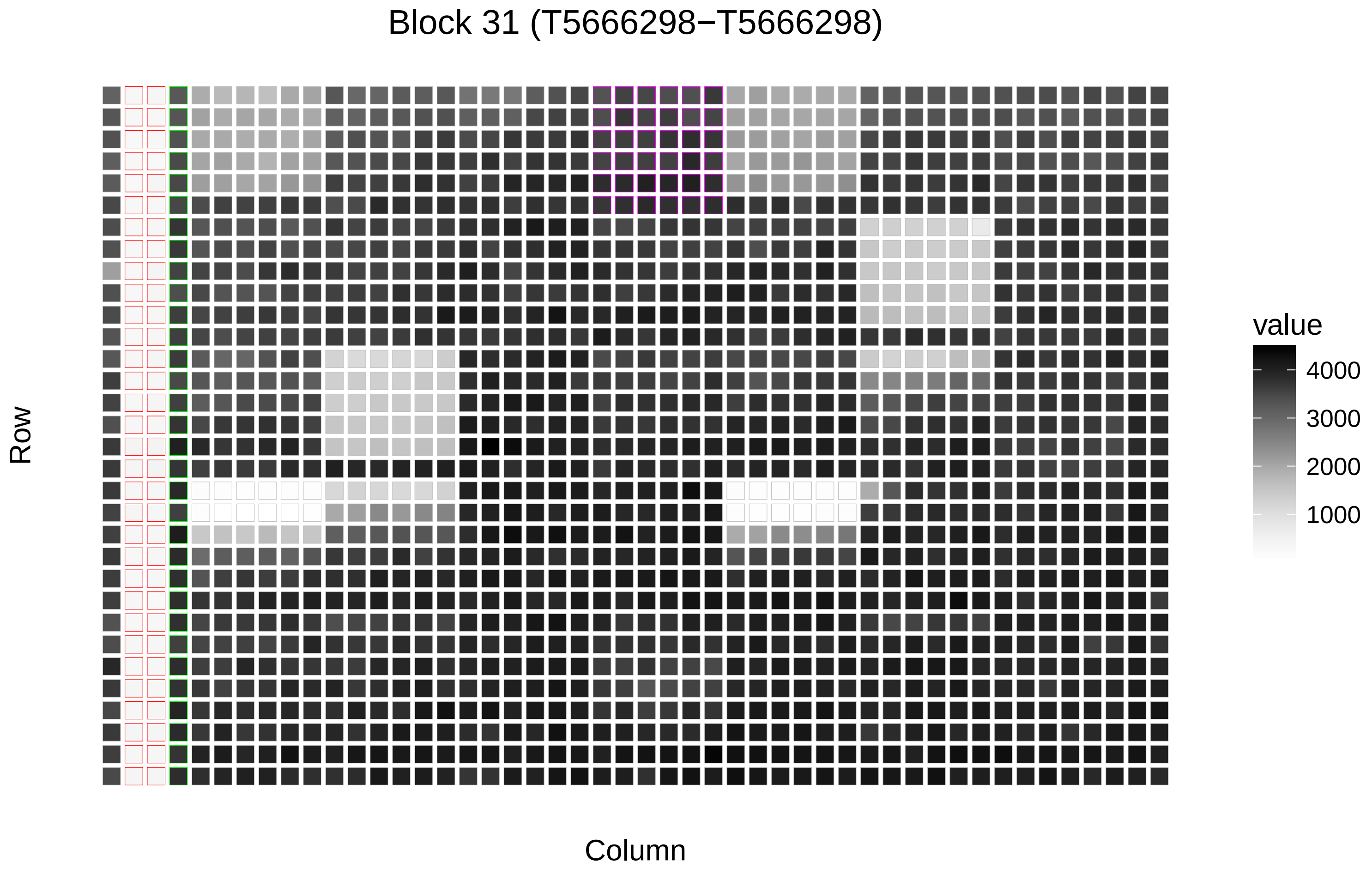
Block 31 (T5666298−T5666298)
Row
Column
value
4000
3000
2000
1000

Block 32 (M8900038−T5510610)

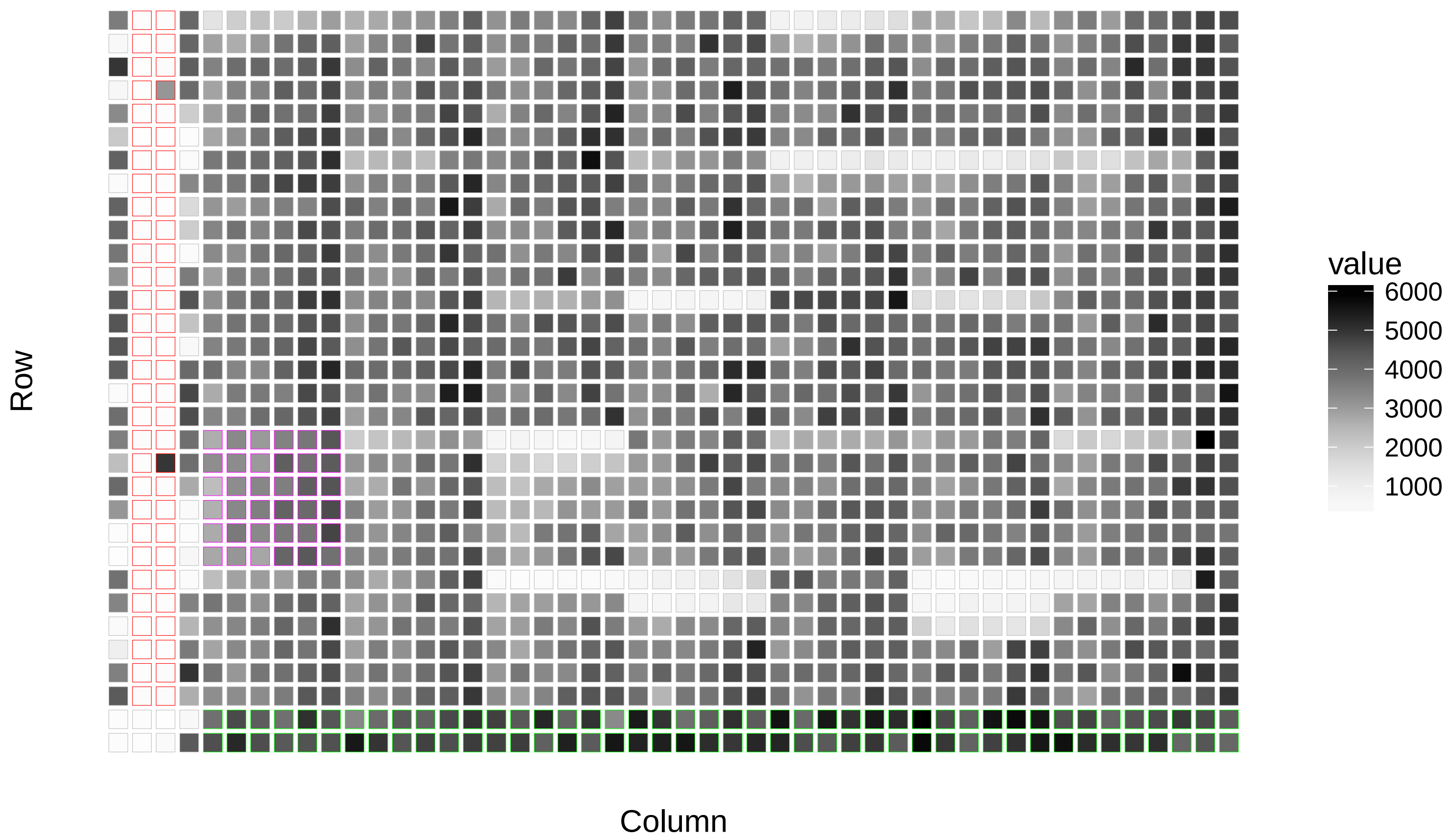

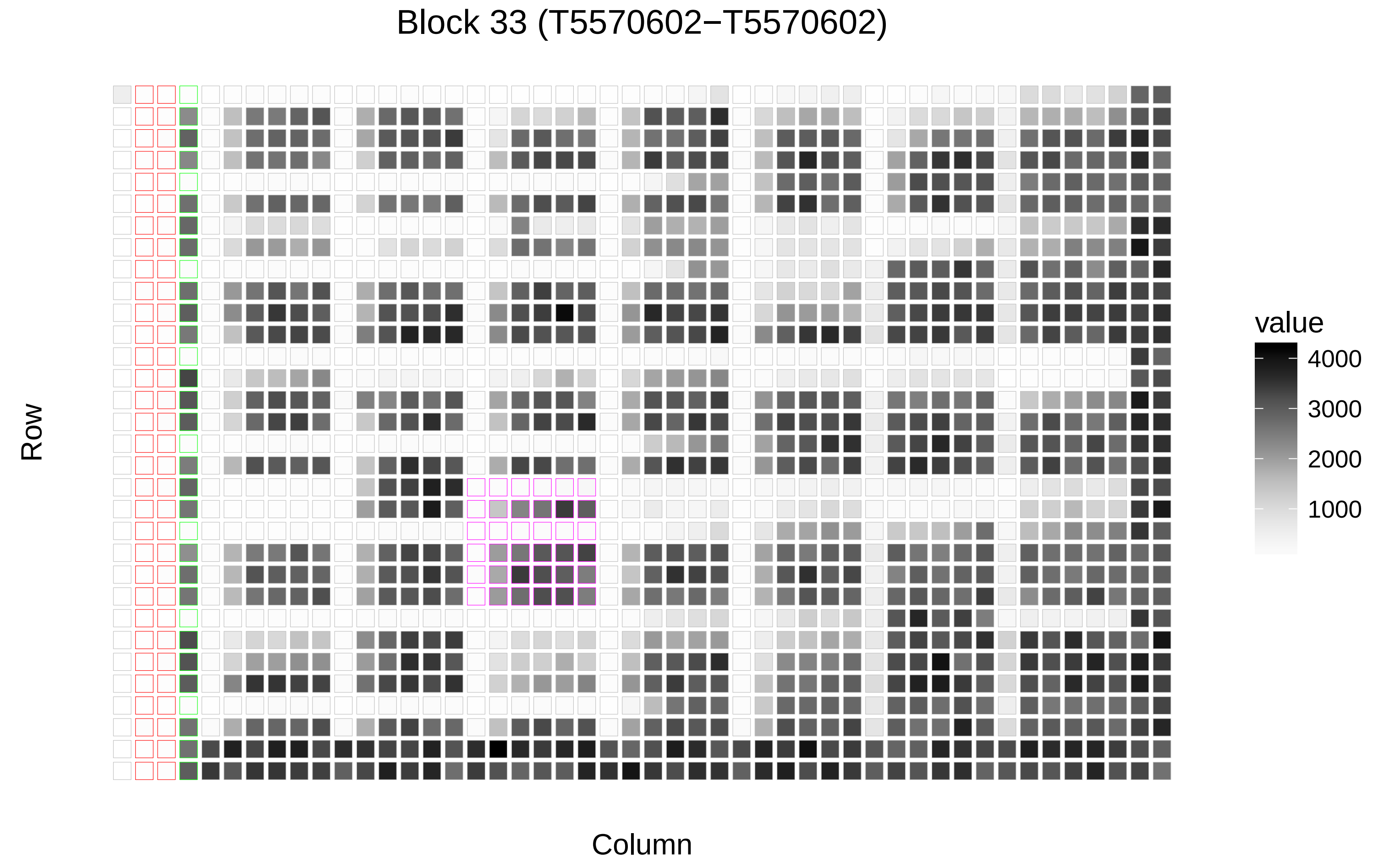
Block 33 (T5570602−T5570602)
Row
Column
value
4000
3000
2000
1000

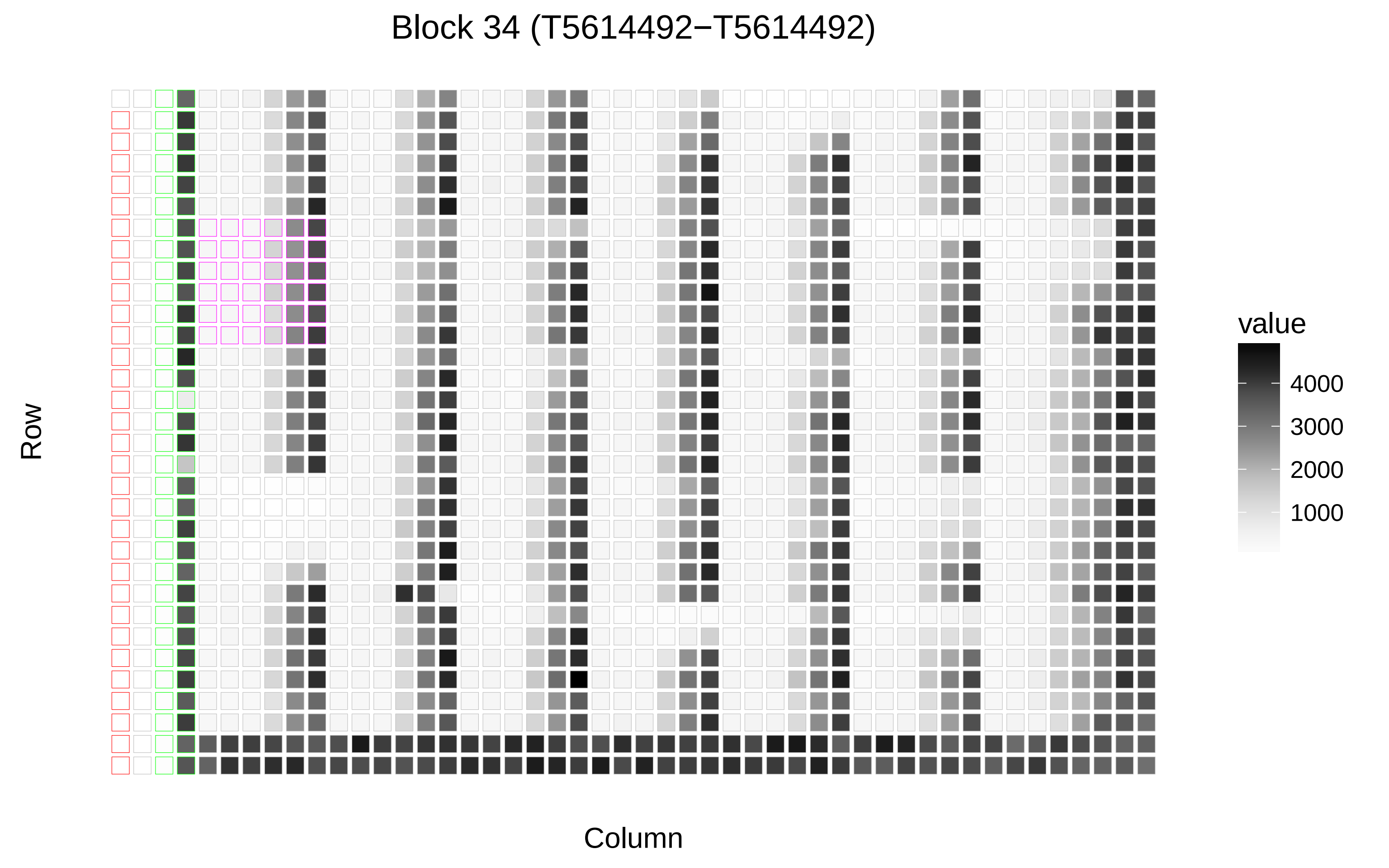

Block 34 (T5614492−T5614492)
Row
Column
value
4000
3000
2000
1000

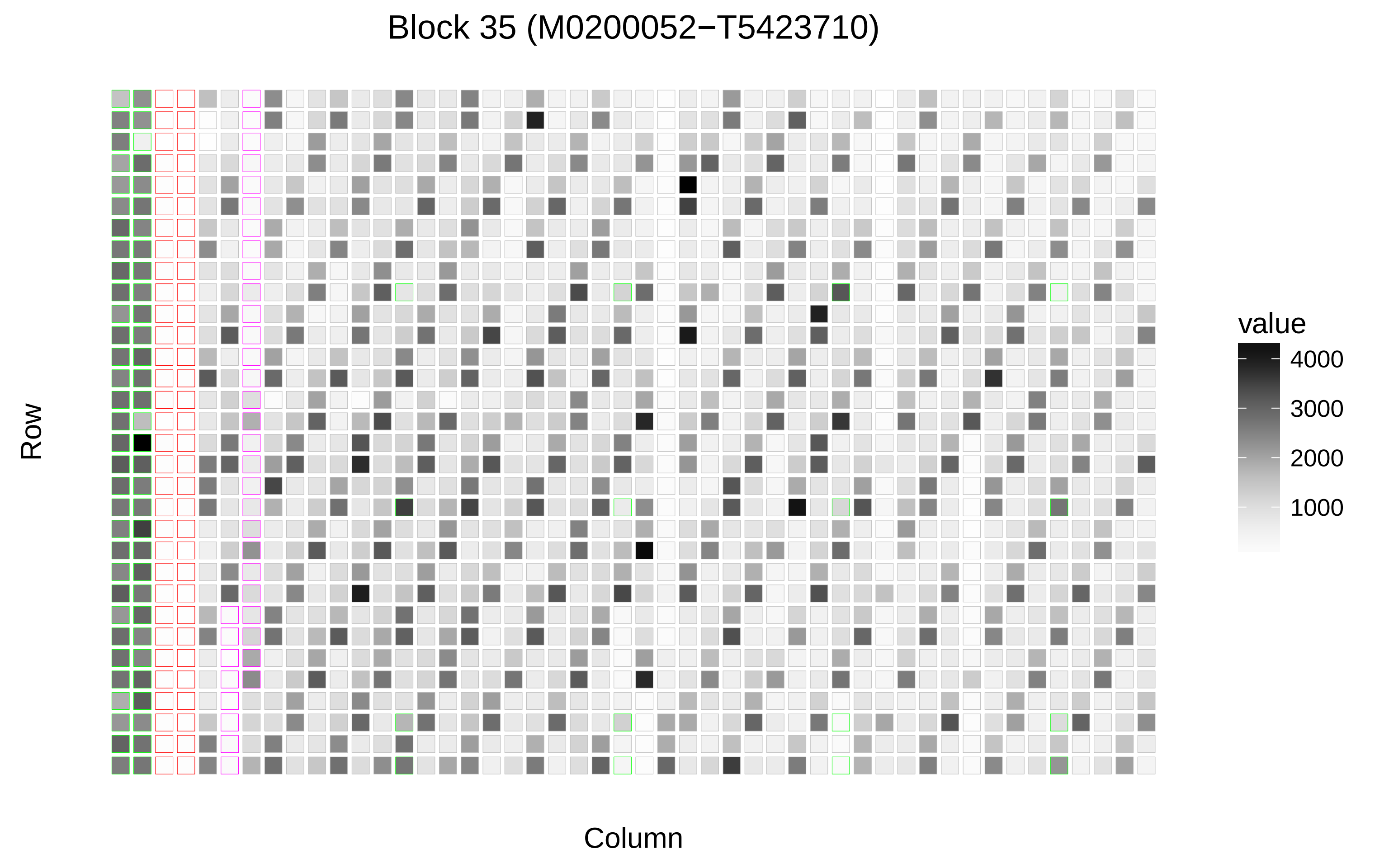
Block 35 (M0200052−T5423710)
Row
Column
value
4000
3000
2000
1000

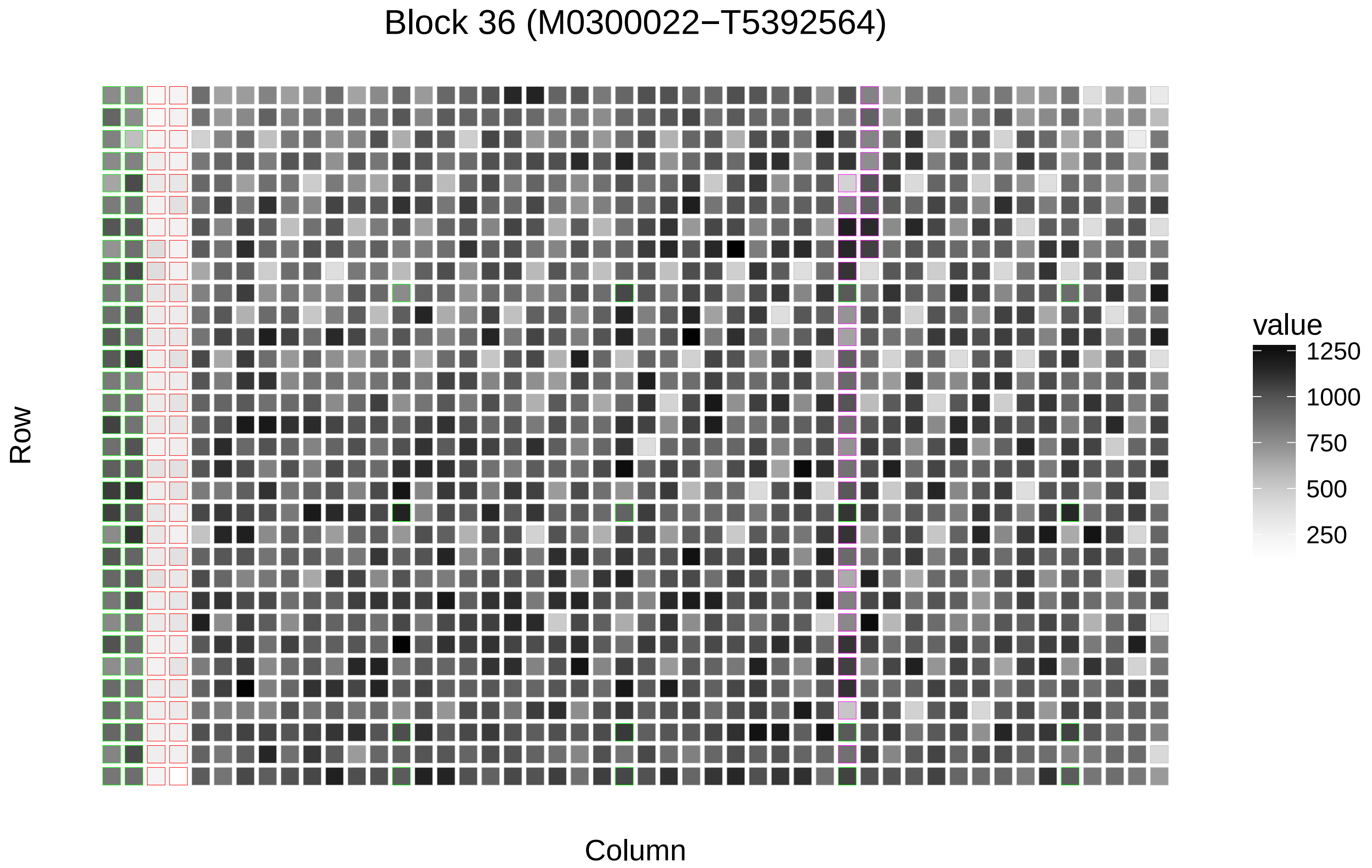
Block 36 (M0300022−T5392564)
Row
Column
value
1250
1000
750
500
250

Block 37 (T5666284−T5666284)

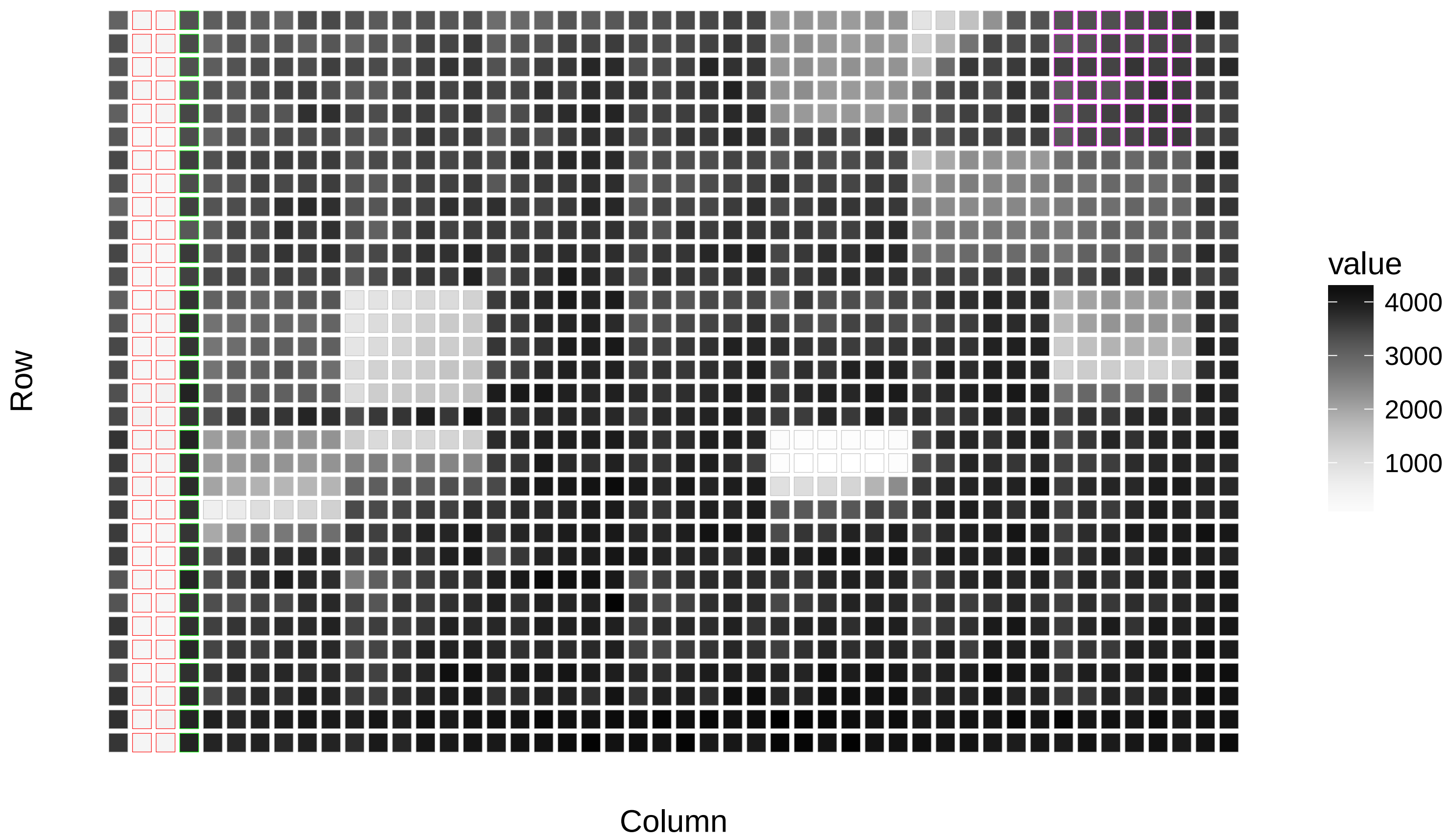

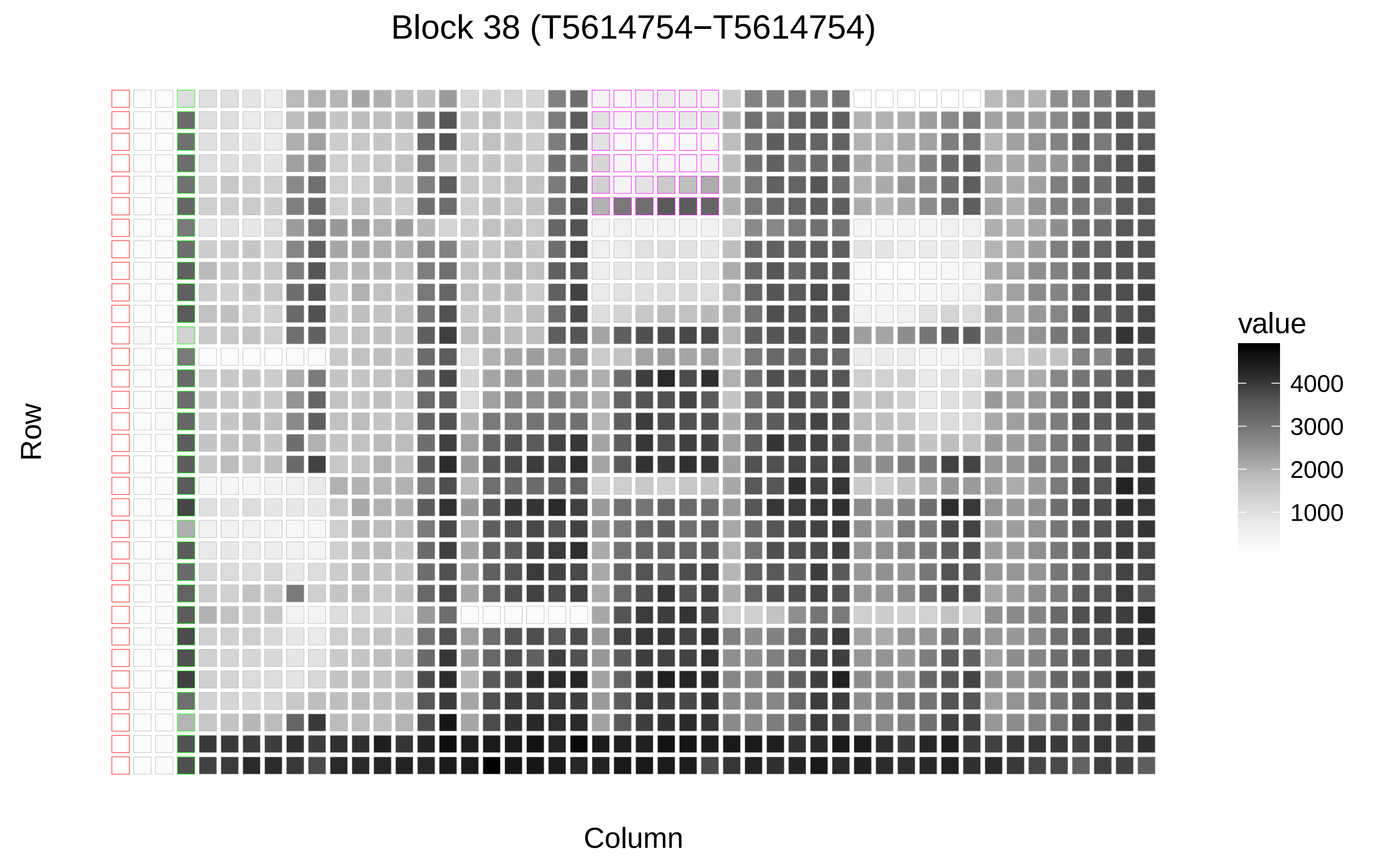
Block 38 (T5614754−T5614754)
Row
Column
value
4000
3000
2000
1000

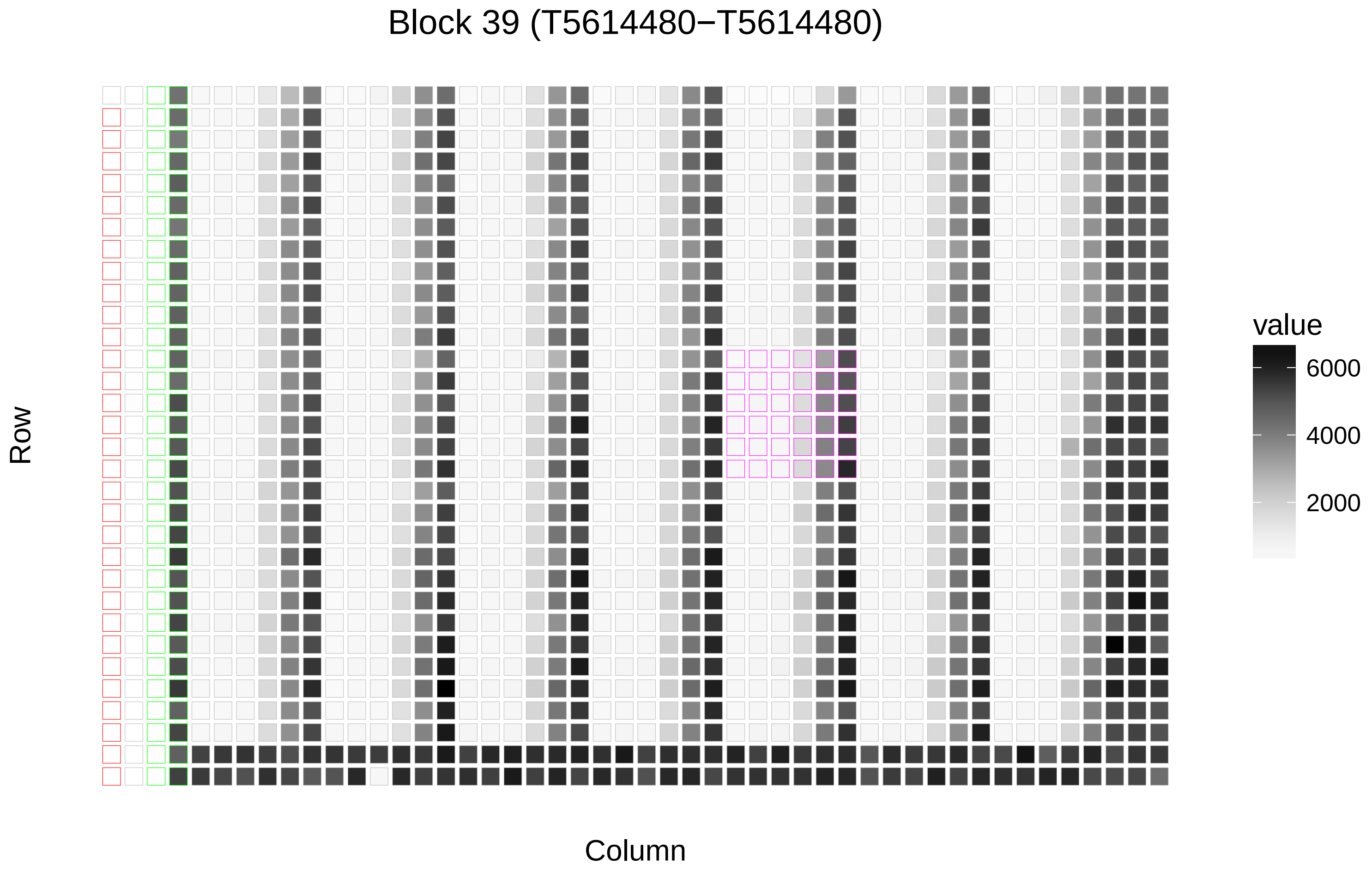
Block 39 (T5614480−T5614480)
Row
Column
value
6000
4000
2000

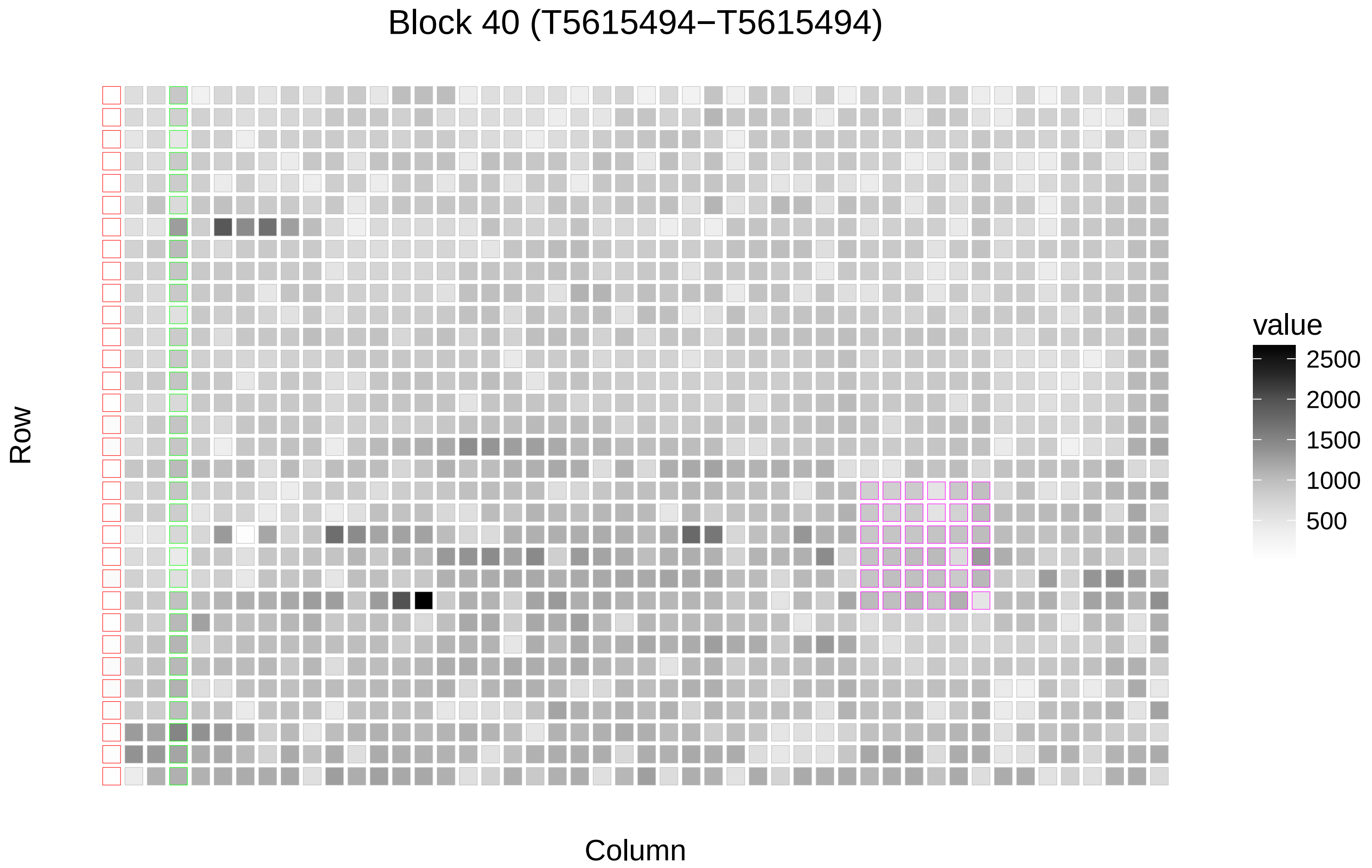
Block 40 (T5615494−T5615494)
Row
Column
value
2500
2000
1500
1000
500

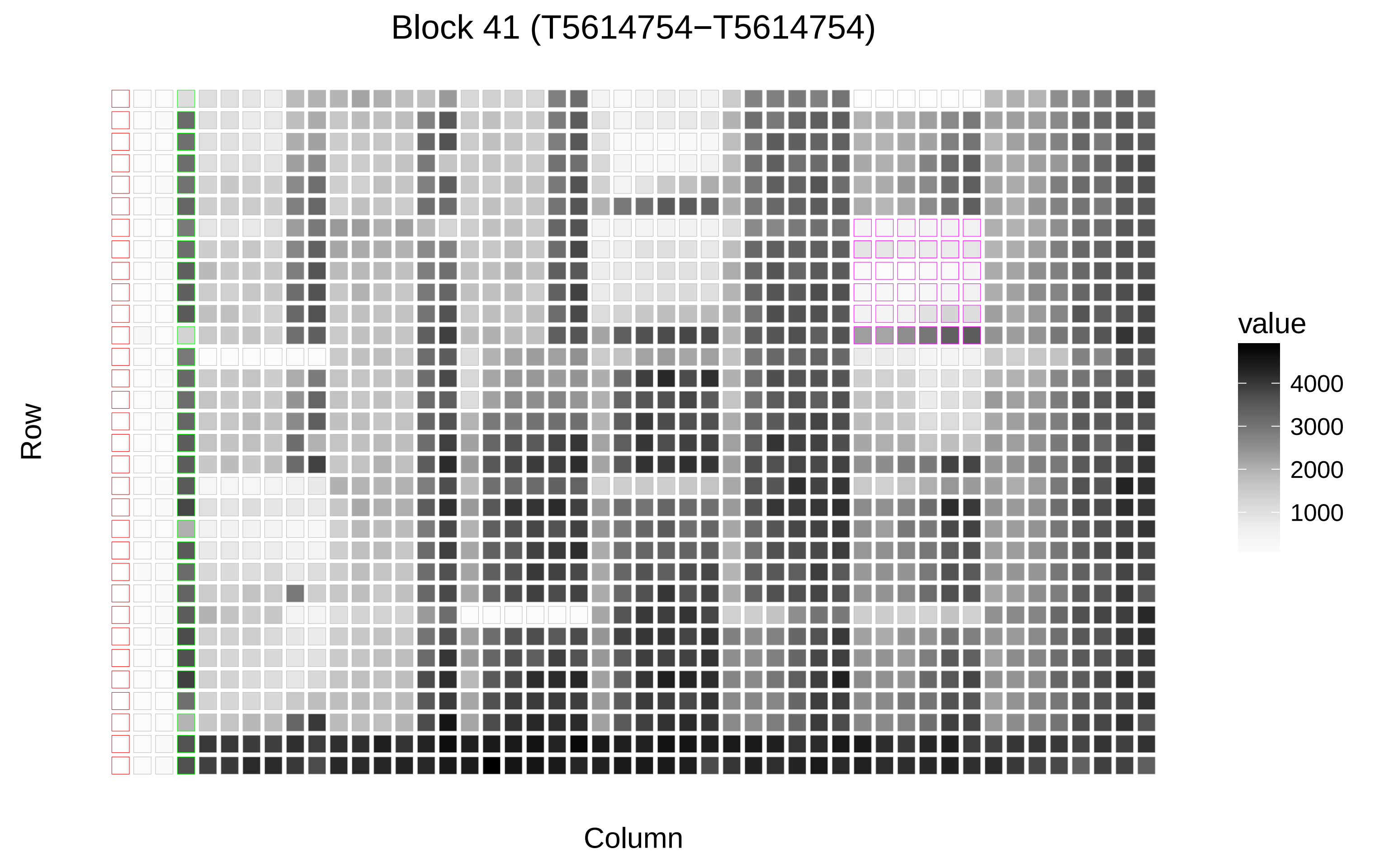

Block 41 (T5614754−T5614754)
Row
Column
value
4000
3000
2000
1000

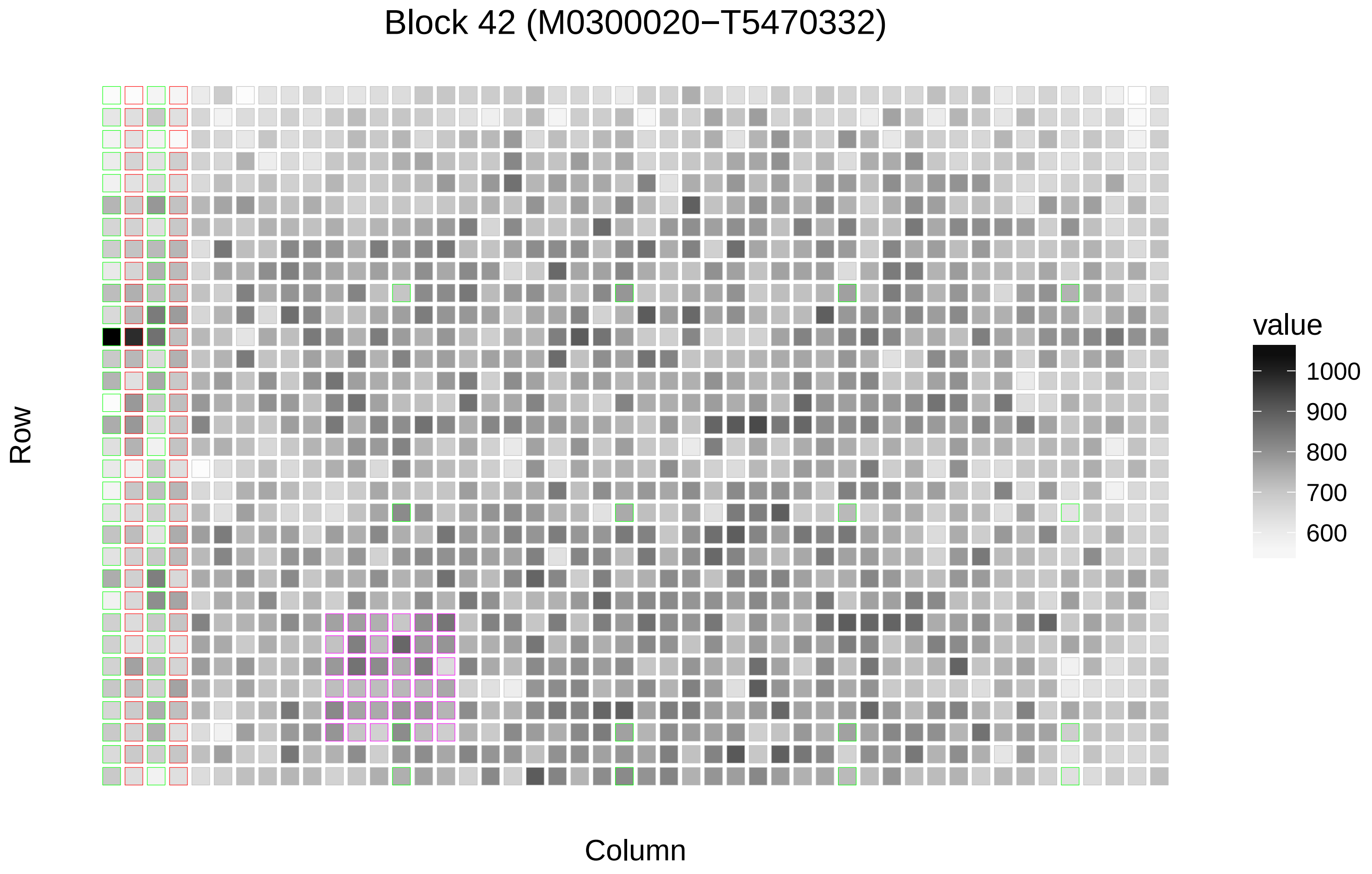
Block 42 (M0300020−T5470332)
value
1000
900
800
700
600
Row
Column

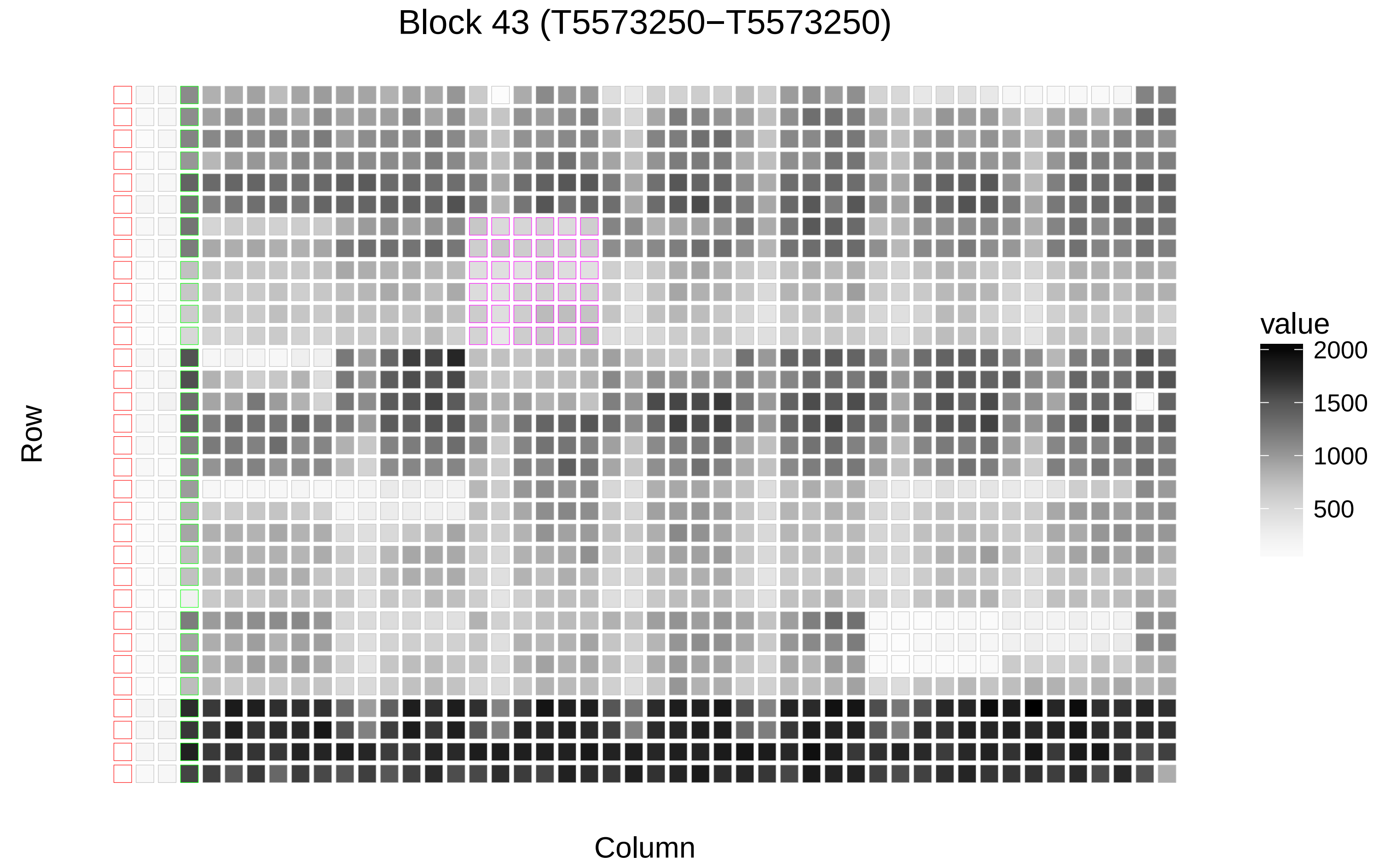

Block 43 (T5573250−T5573250)
Row
Column
value
2000
1500
1000
500

Block 44 (T5616746−T5616746)

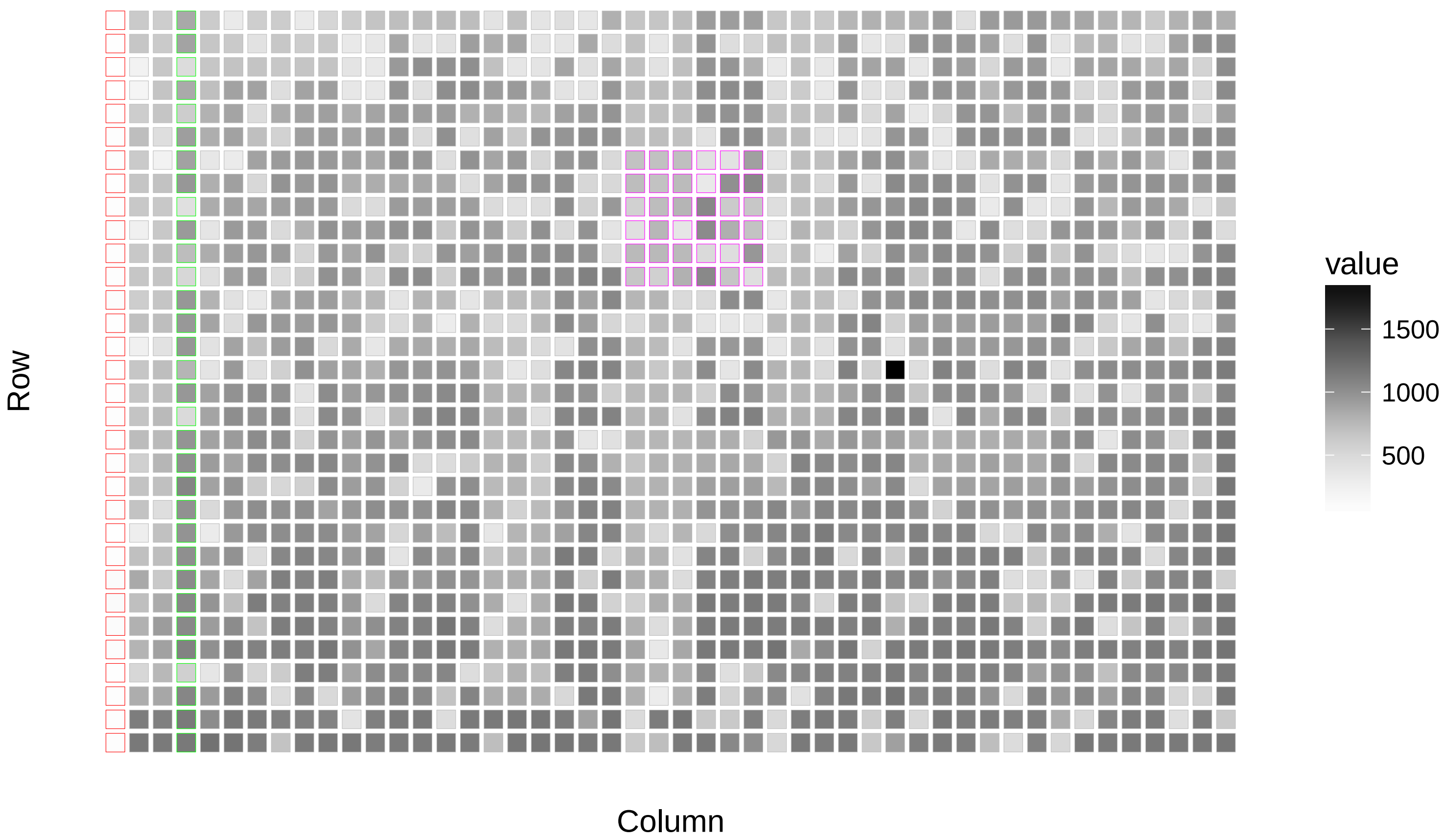

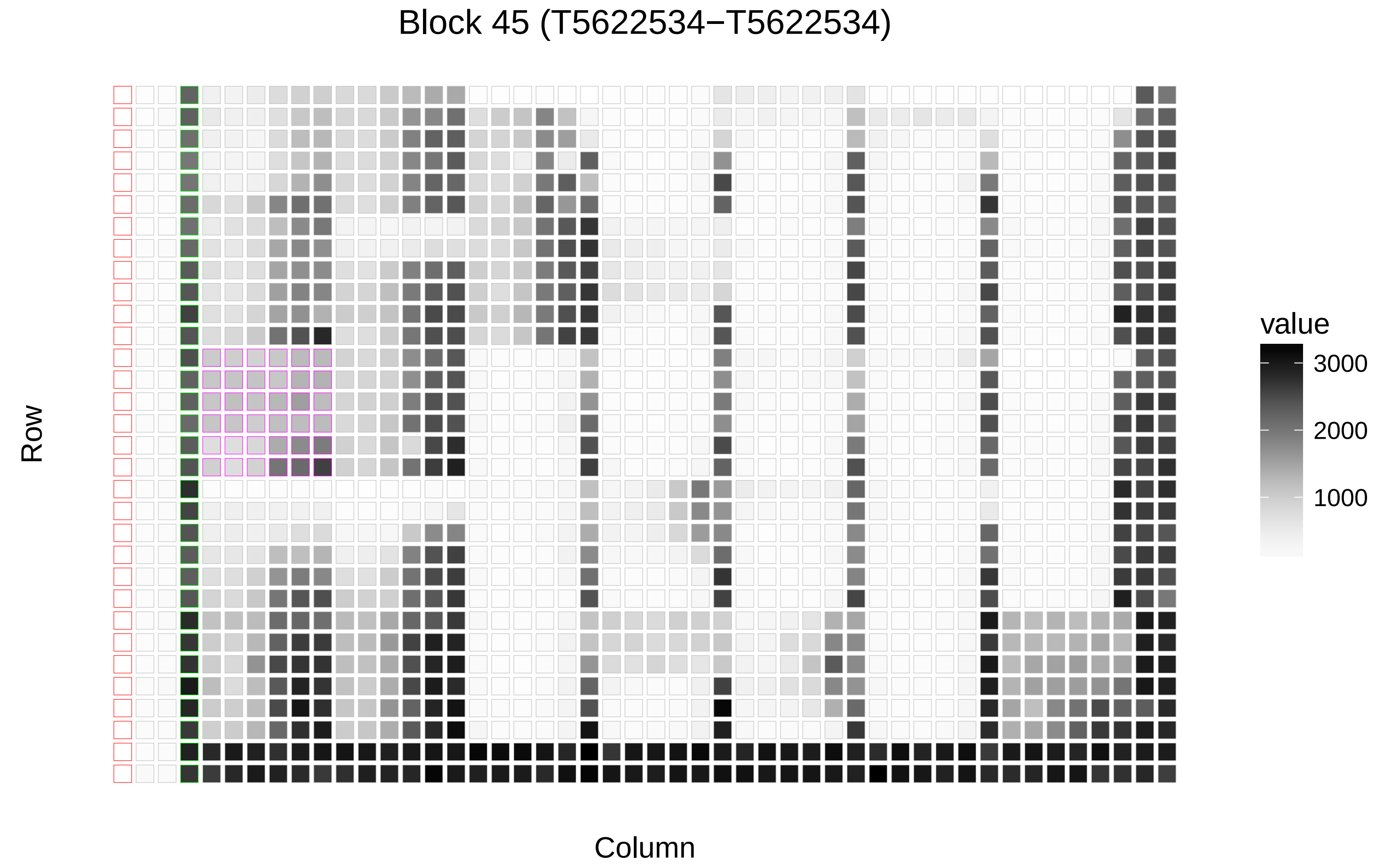
Block 45 (T5622534−T5622534)
Row
Column
value
3000
2000
1000

Block 46 (T5570410−T5570410)

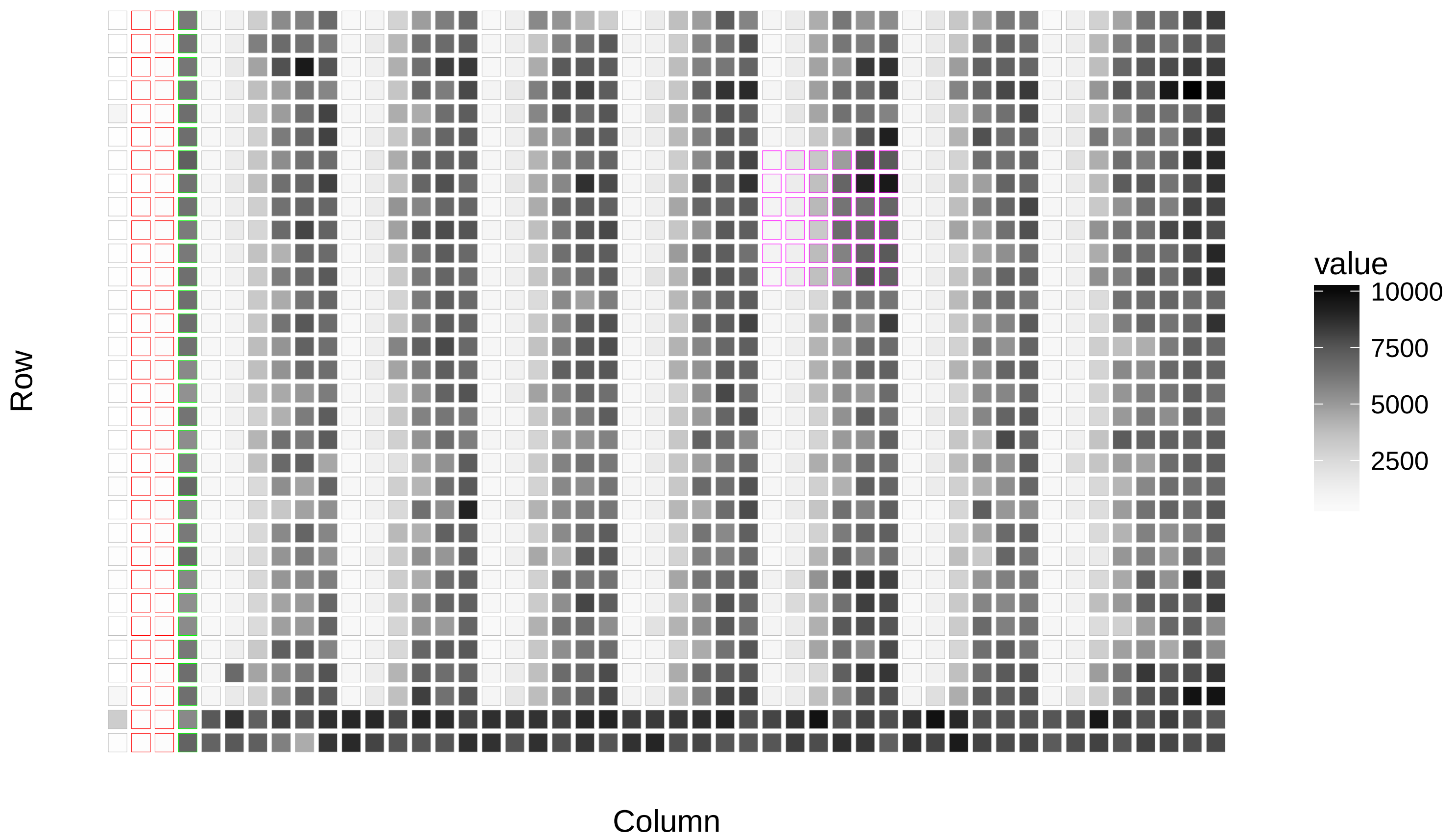

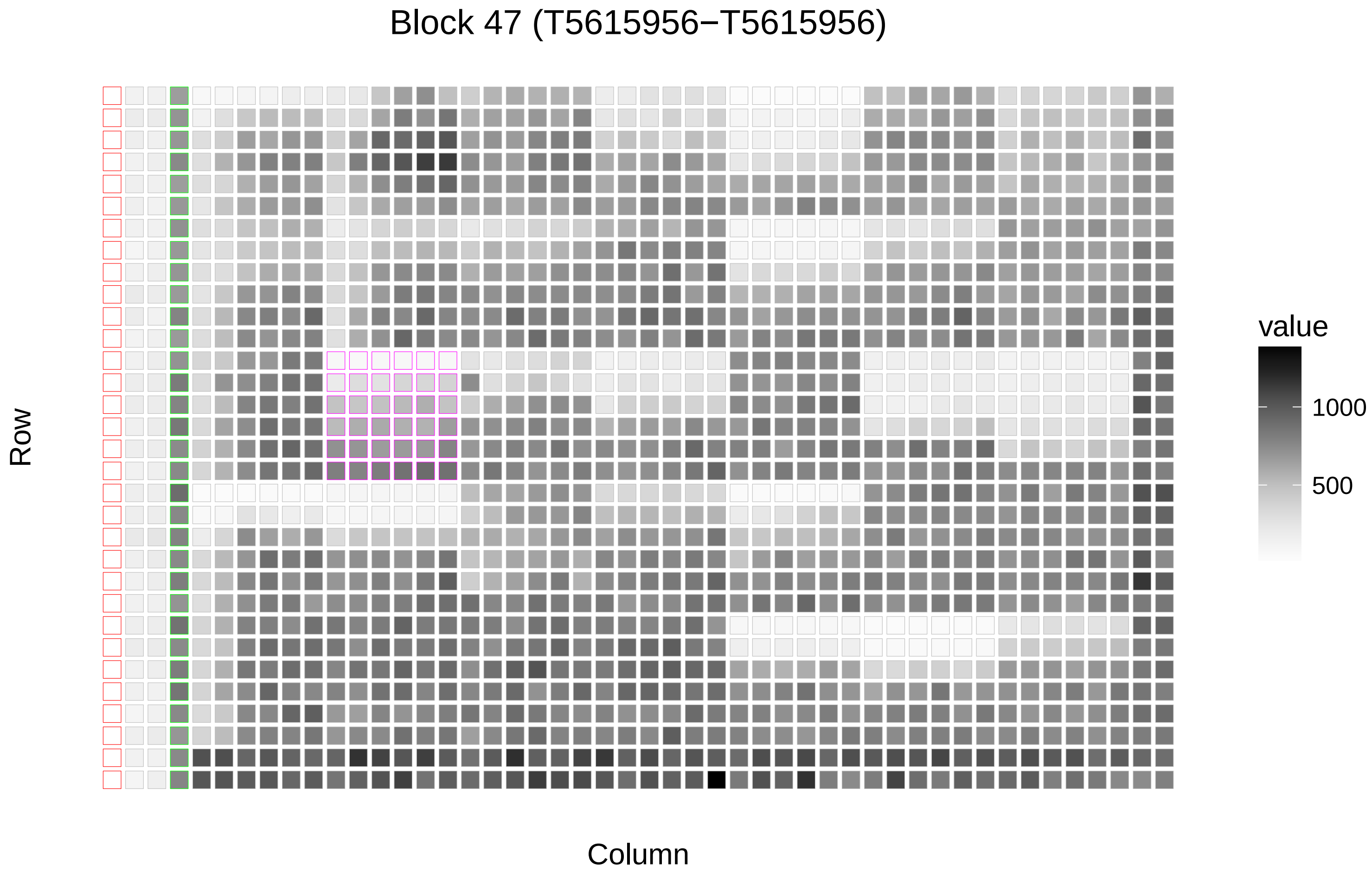
Block 47 (T5615956−T5615956)
Row
Column
value
1000
500

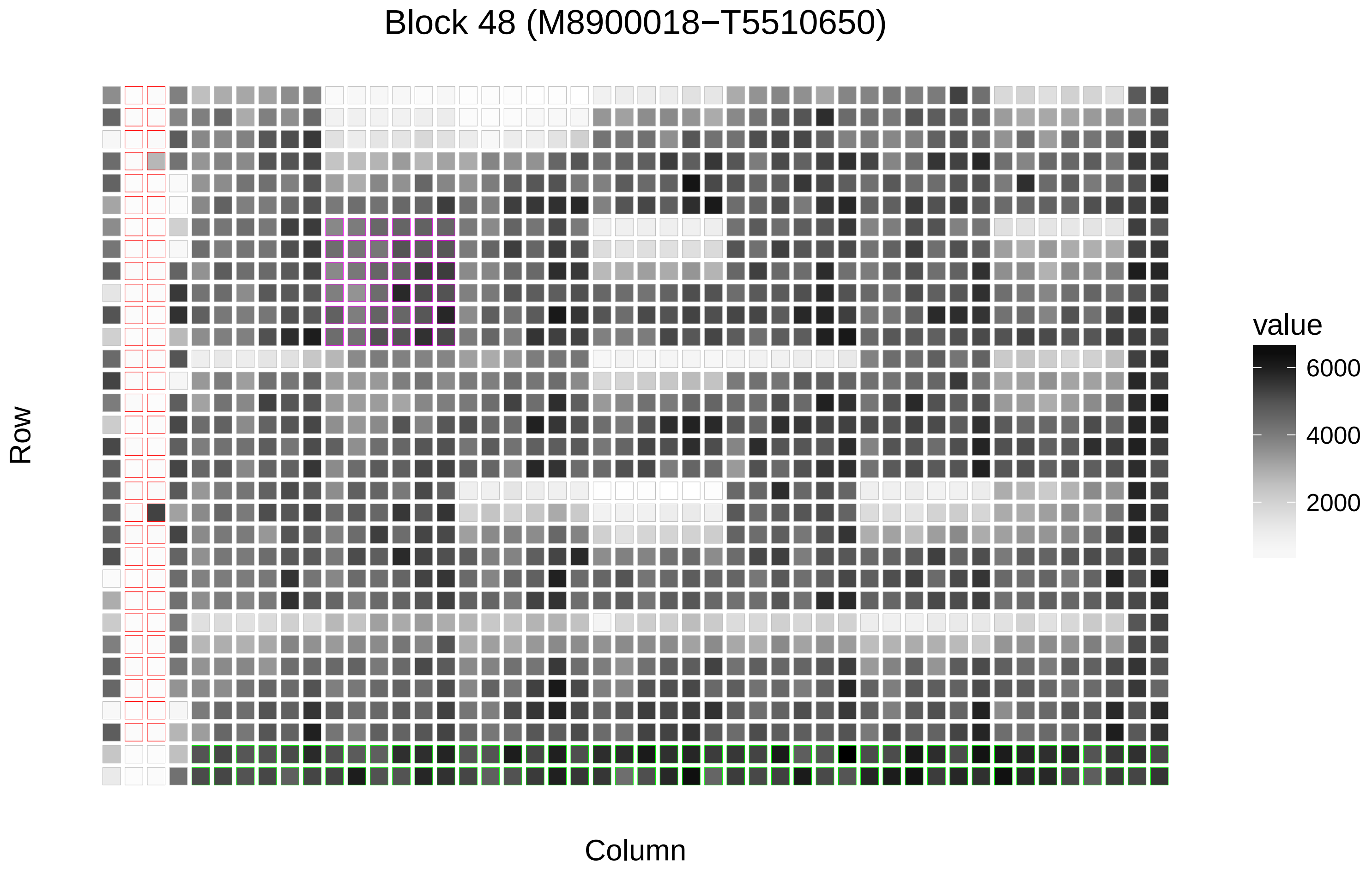
Block 48 (M8900018−T5510650)
Row
Column
value
6000
4000
2000

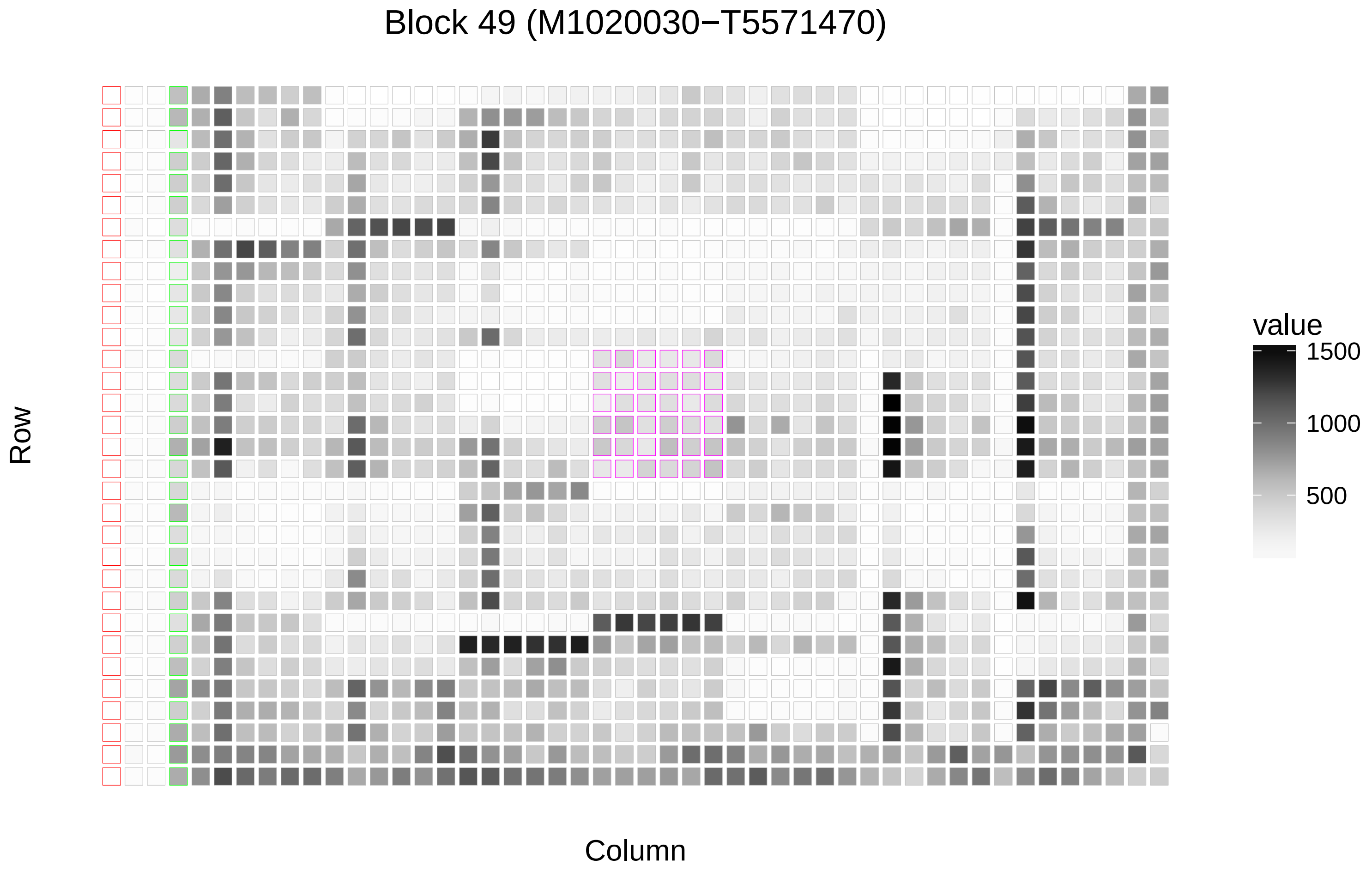
Block 49 (M1020030−T5571470)
Row
Column
value
1500
1000
500

Block 50 (M8900044−T5510608)

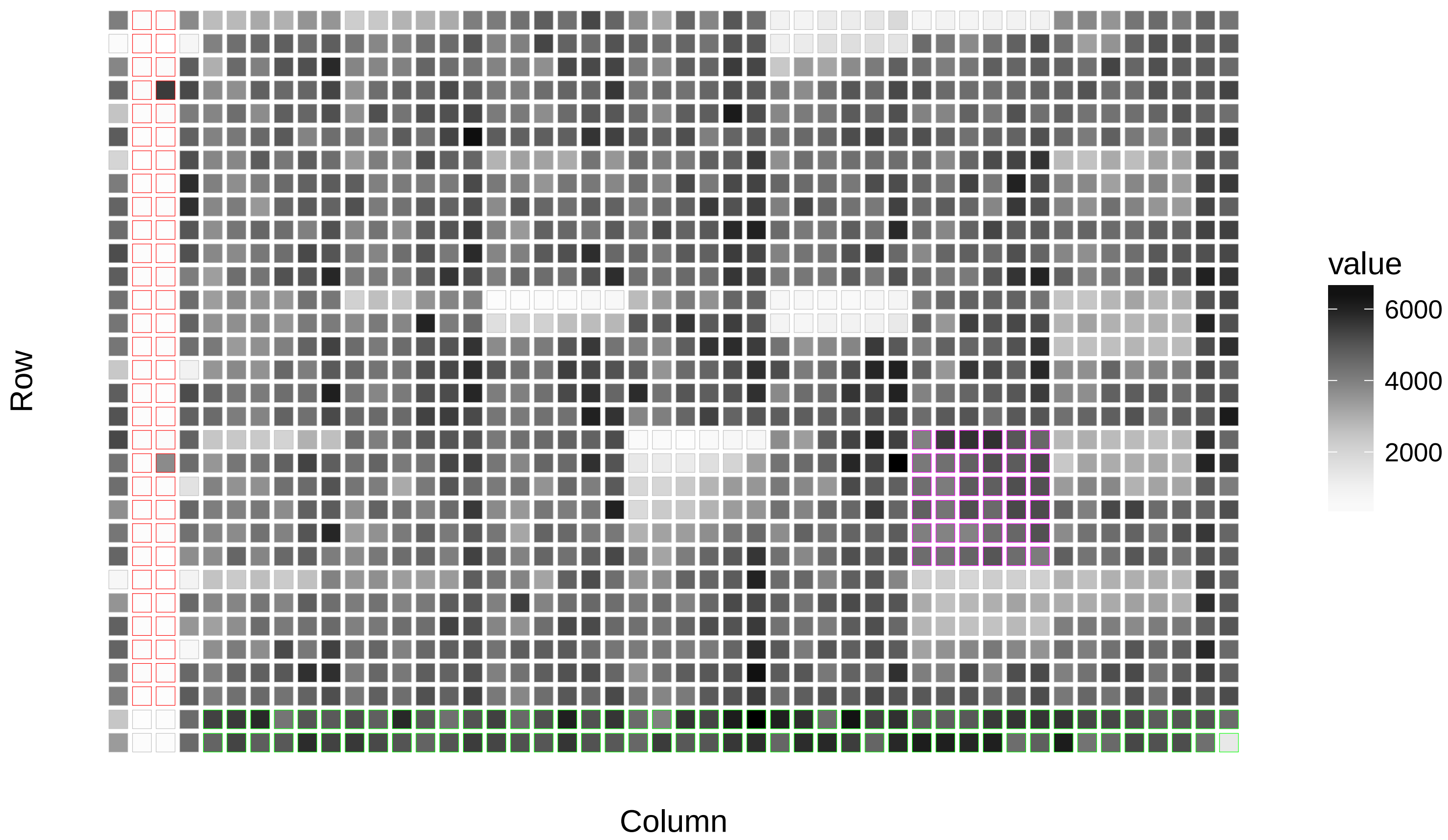

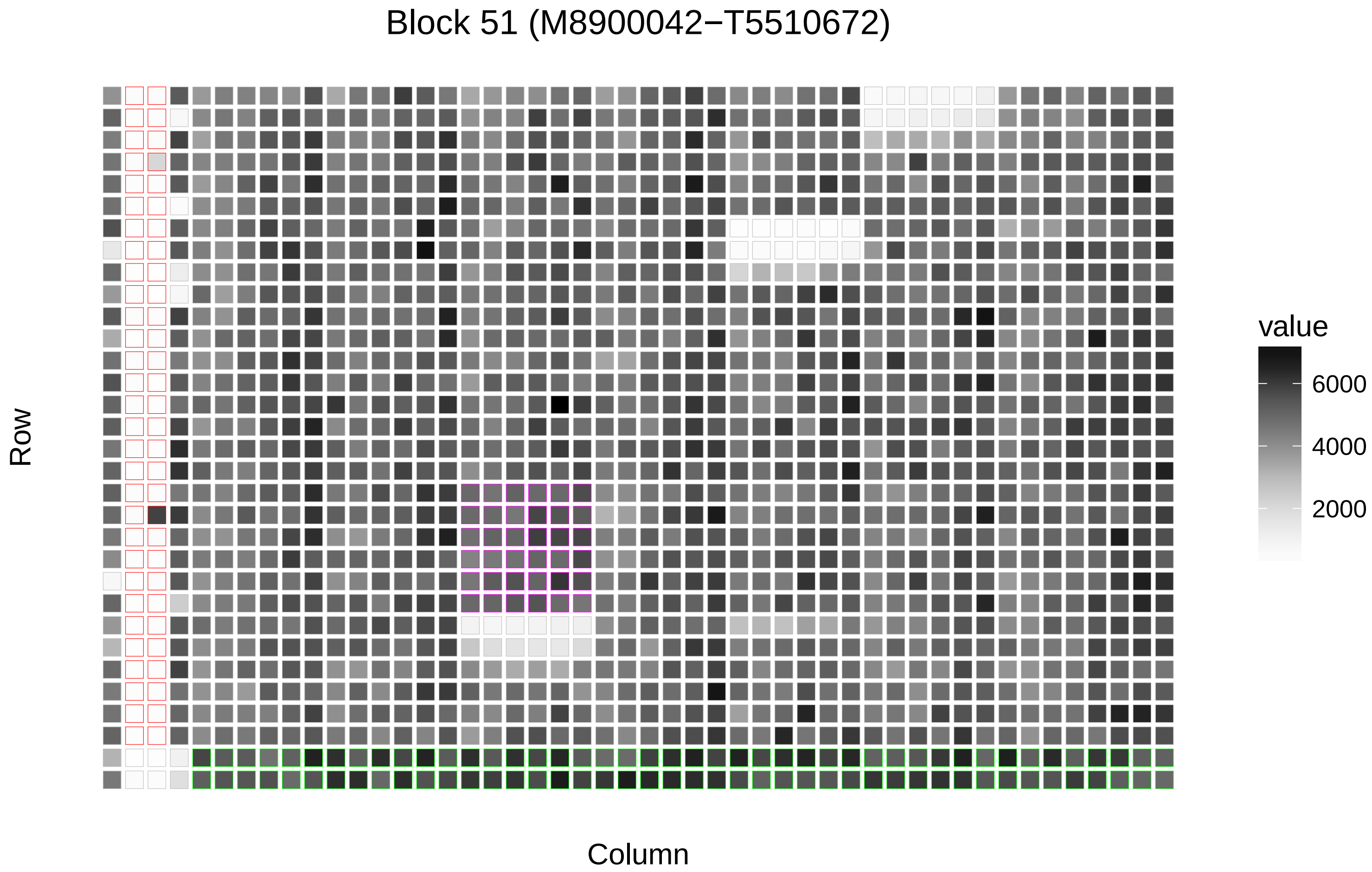

Block 51 (M8900042−T5510672)
Row
Column
value
6000
4000
2000

Block 52 (M1400018−T5436678)

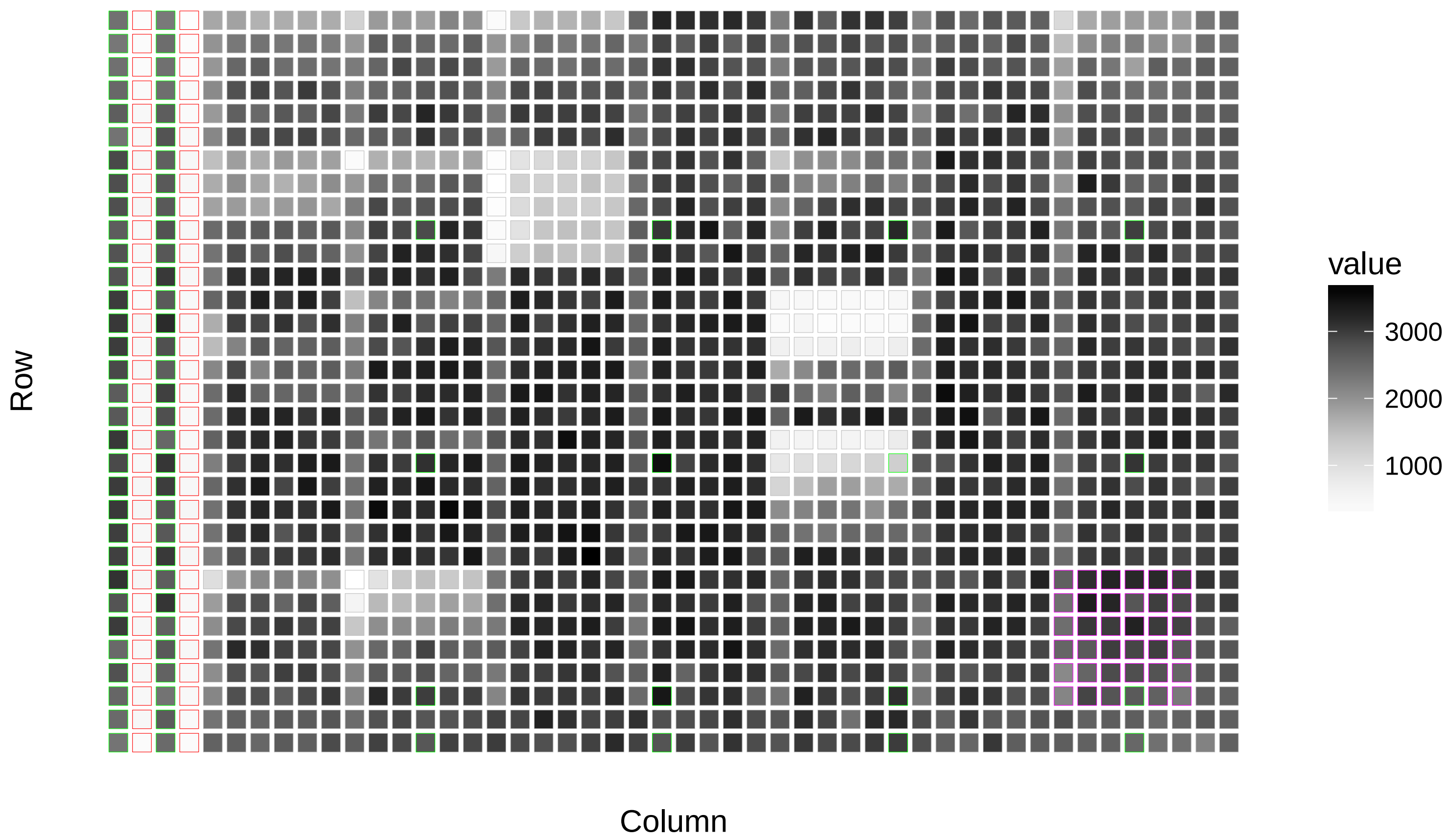

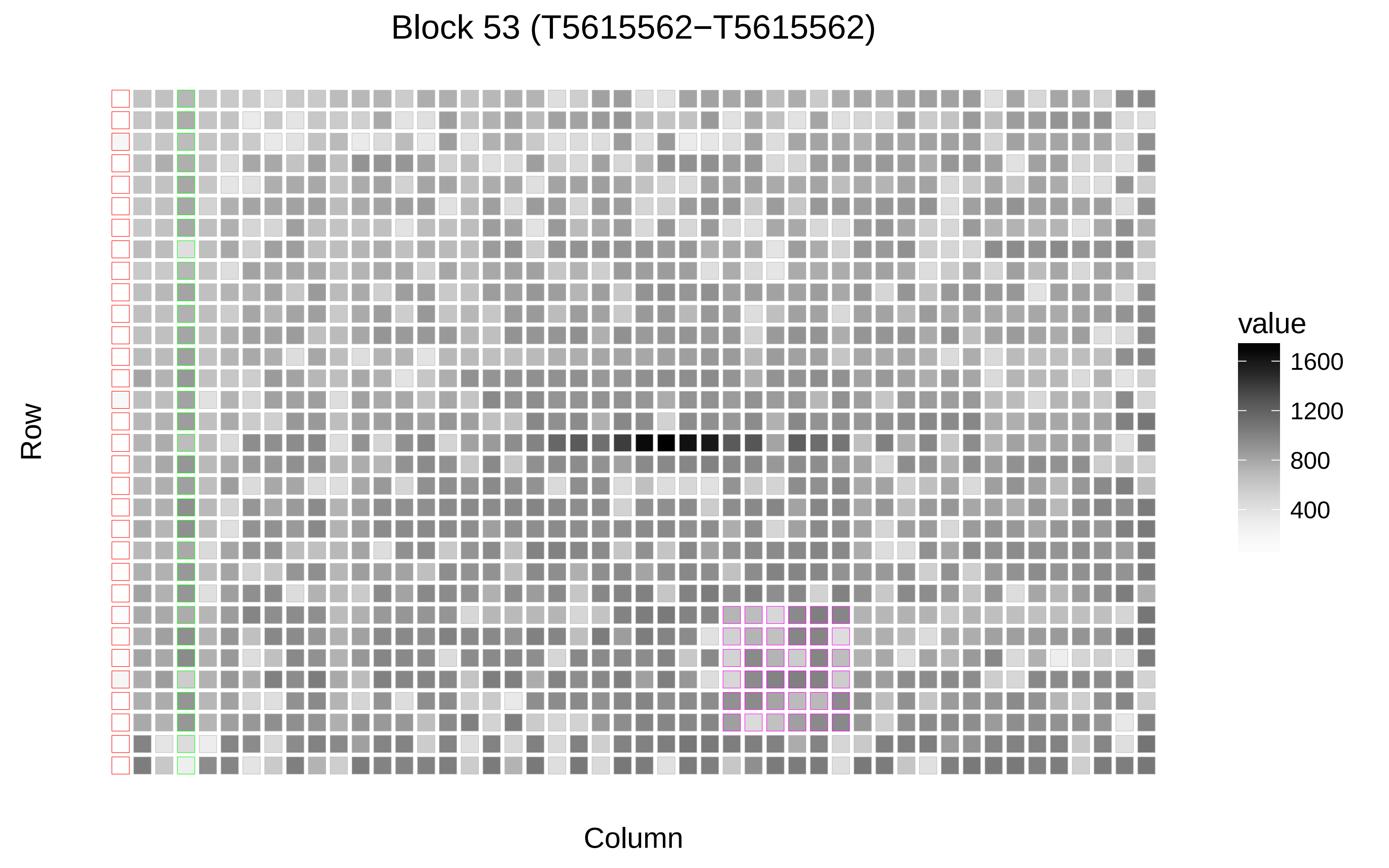

Block 53 (T5615562−T5615562)
Row
Column
value
1600
1200
800
400

Block 54 (M1020034−T5571466)

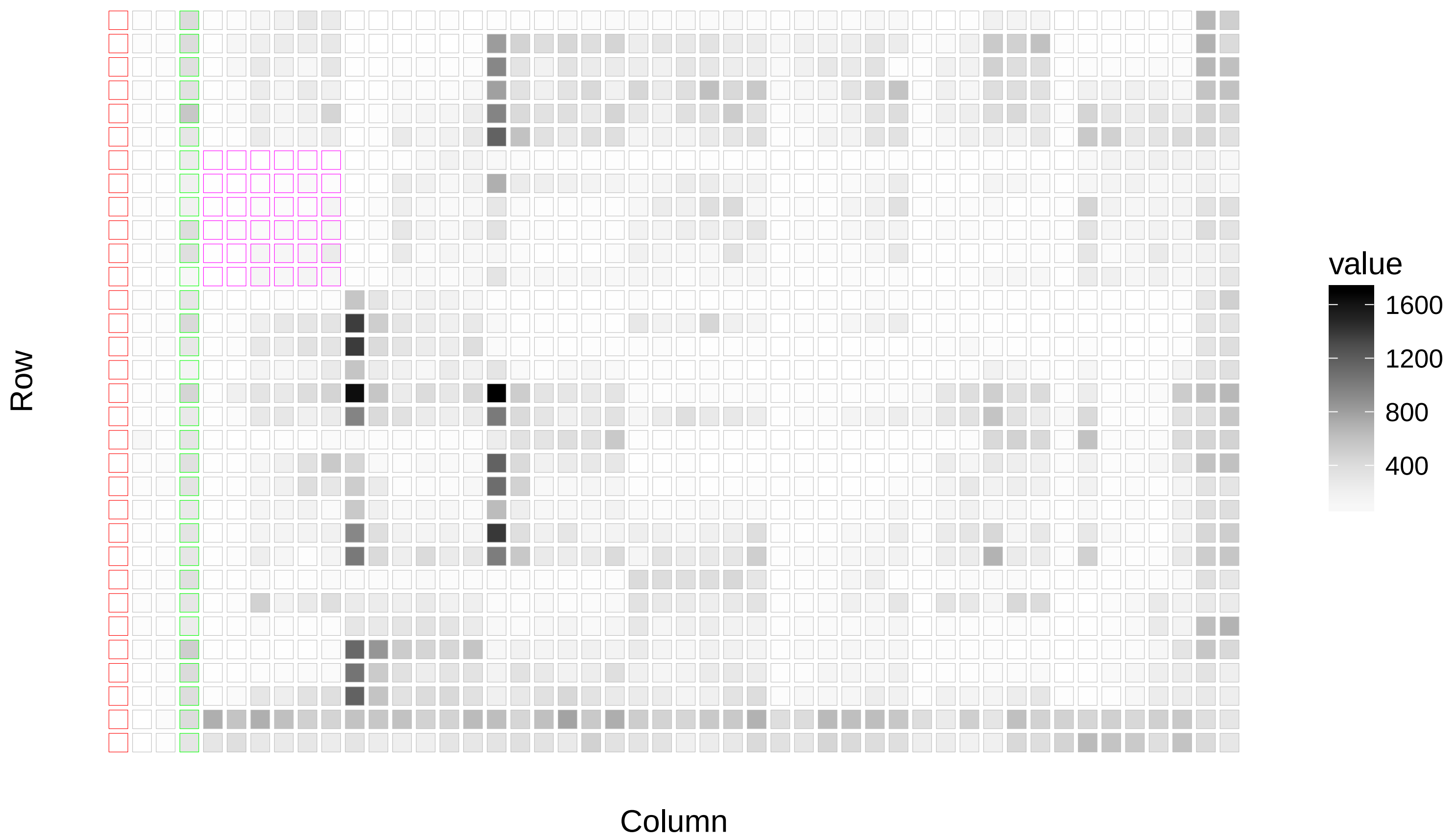

Block 55 (M8900070−T5510632)

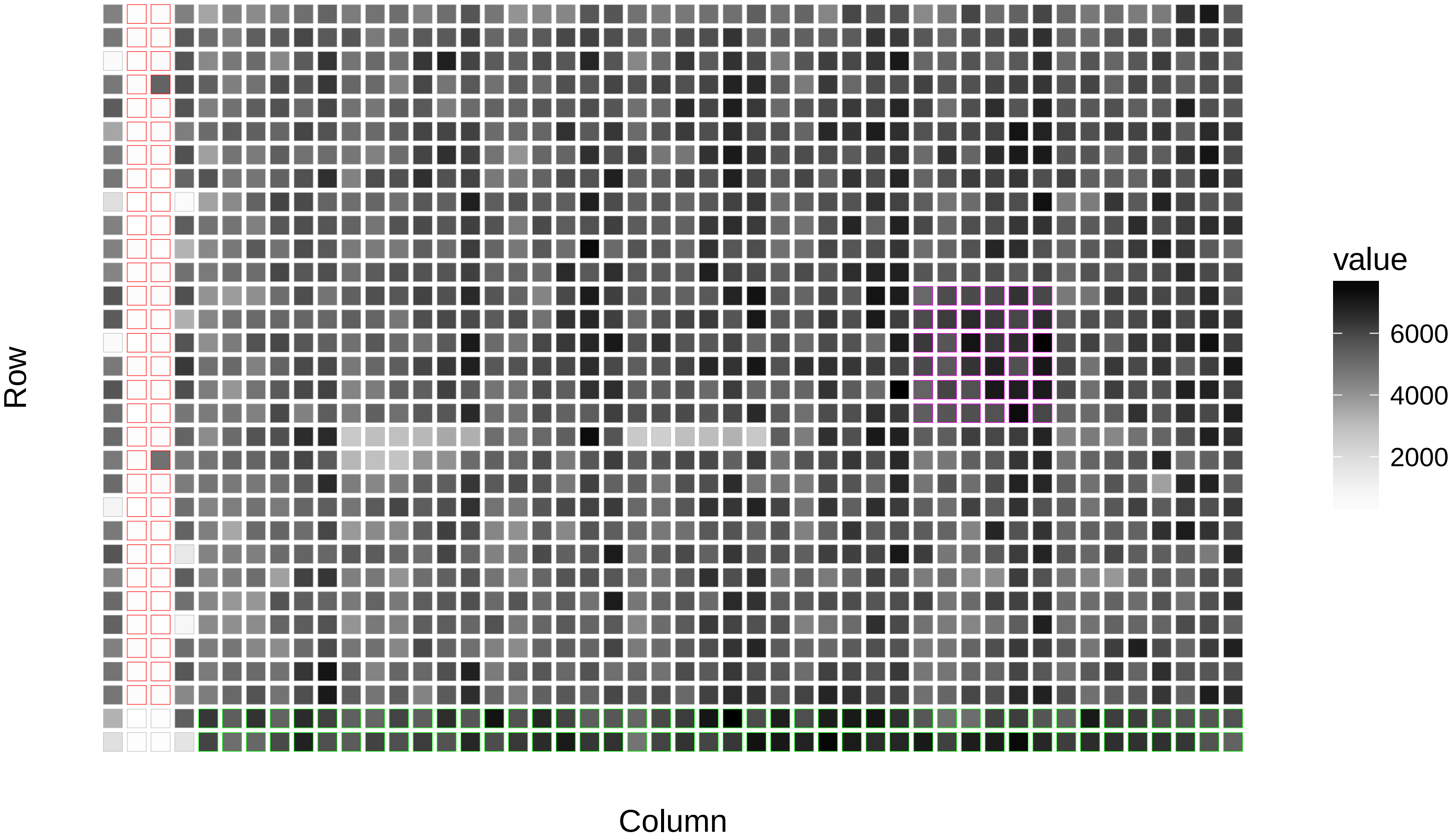

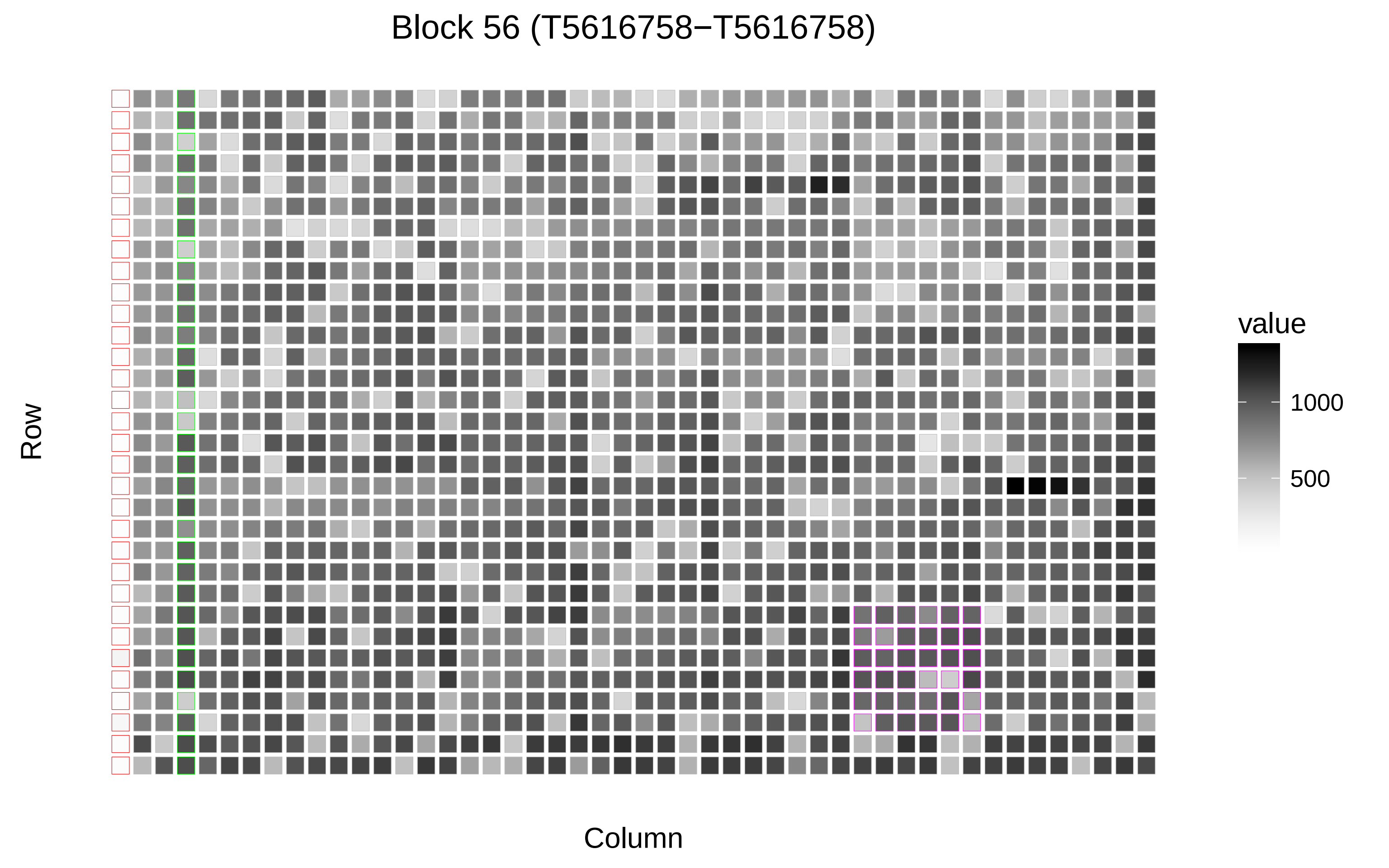
Block 56 (T5616758−T5616758)
Row
Column
value
1000
500

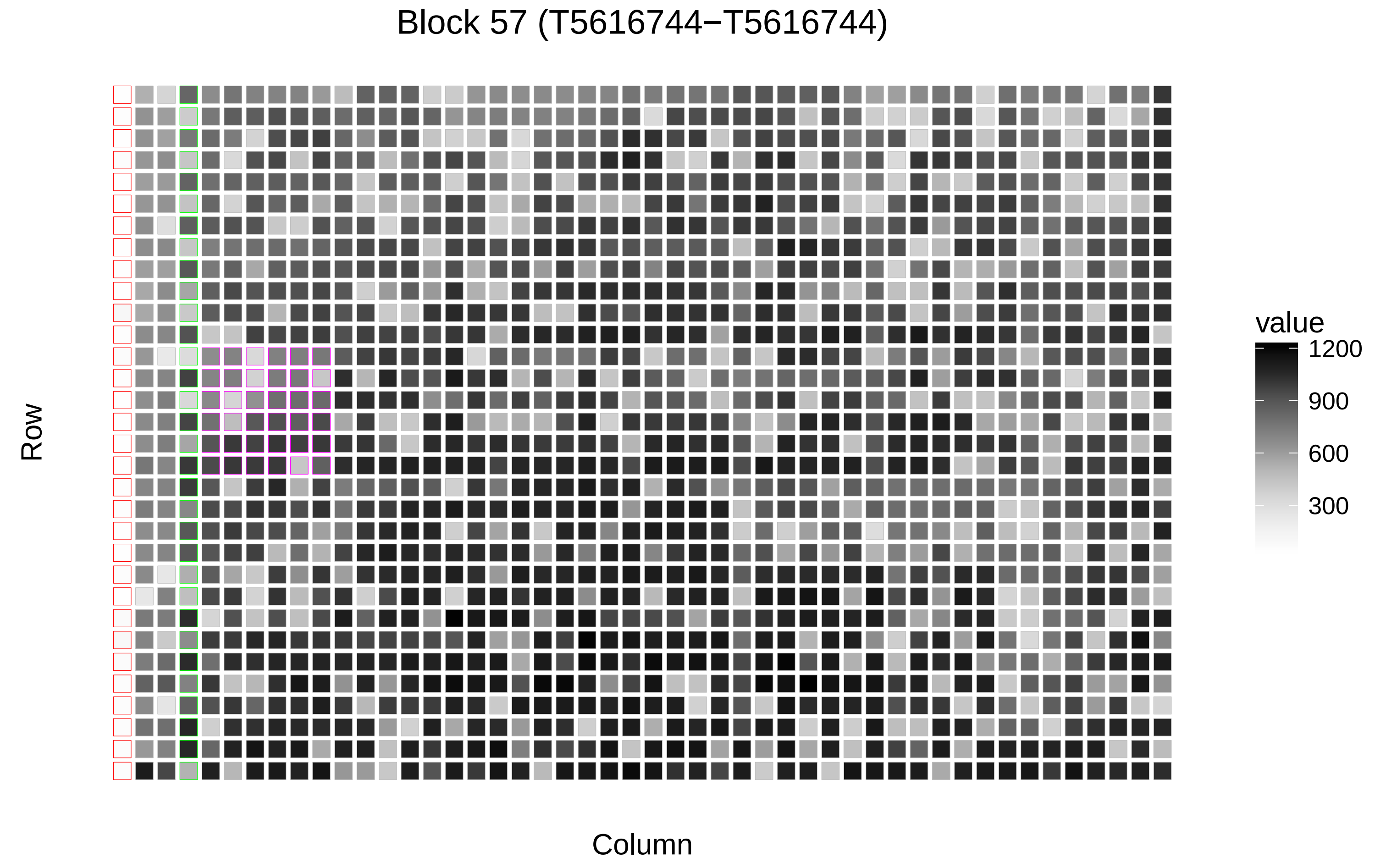

Block 57 (T5616744−T5616744)
Row
Column
value
1200
900
600
300

Block 58 (M0300042−T5423742)

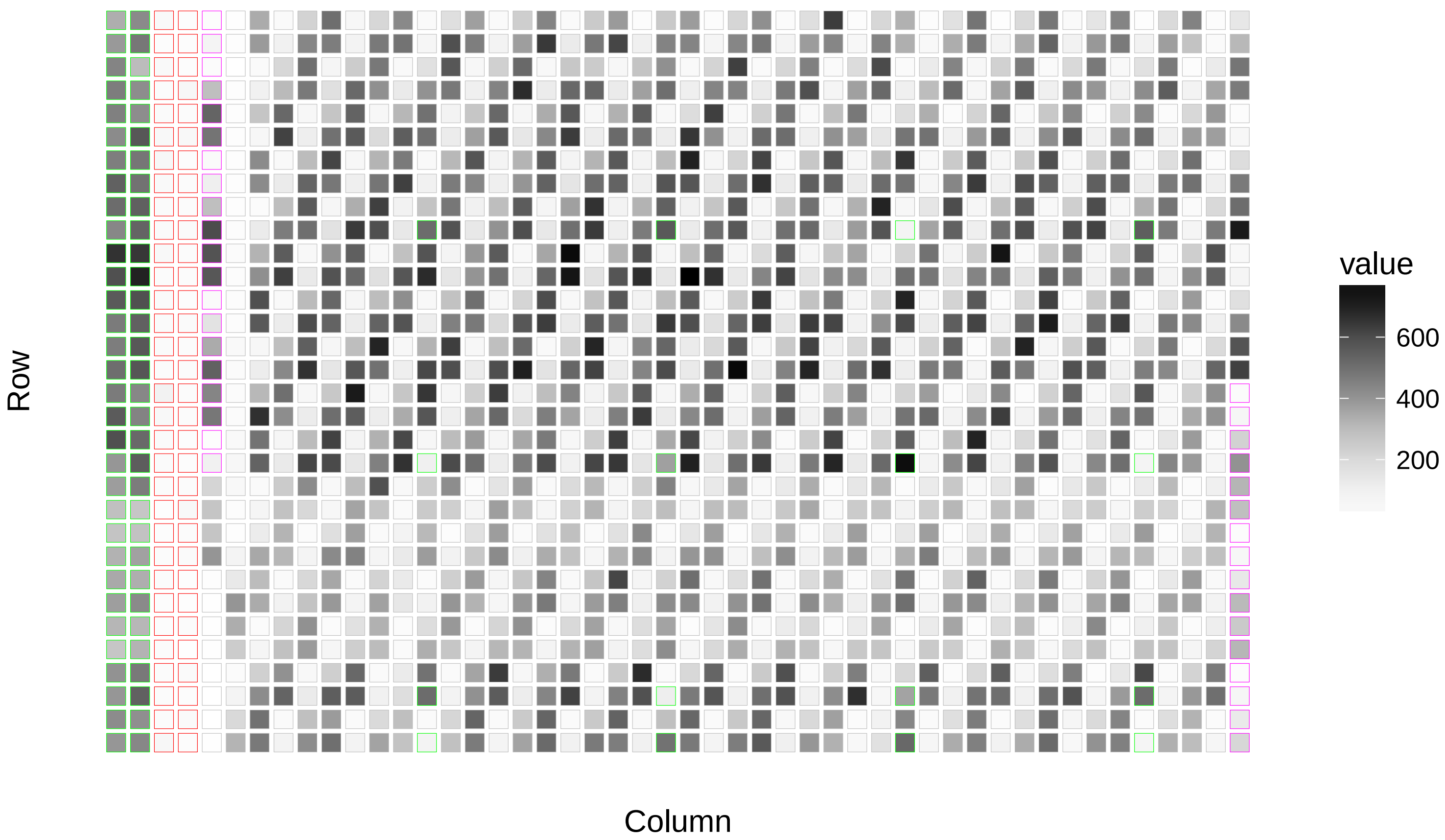

Block 59 (M1400026−T5436718)

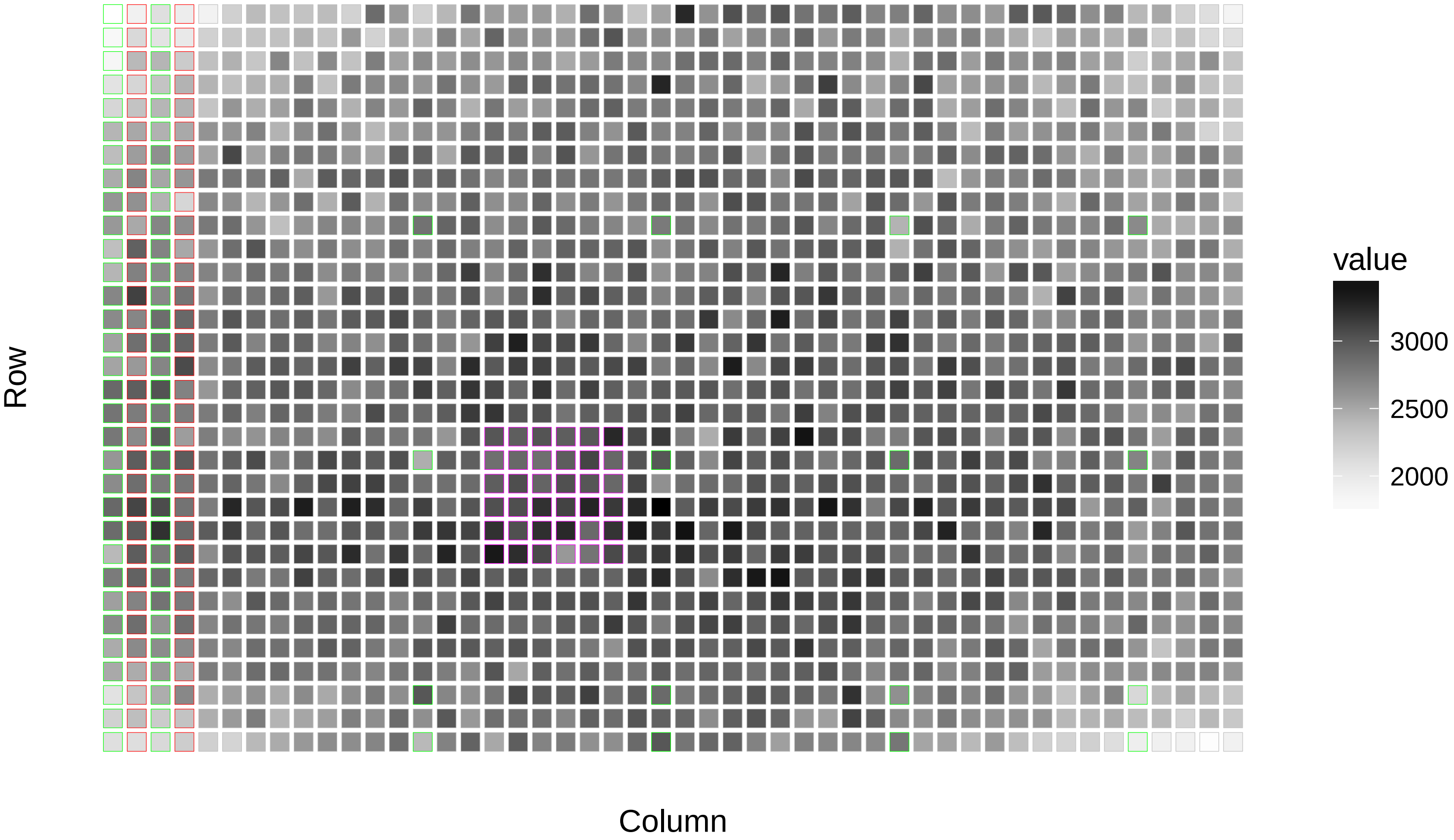

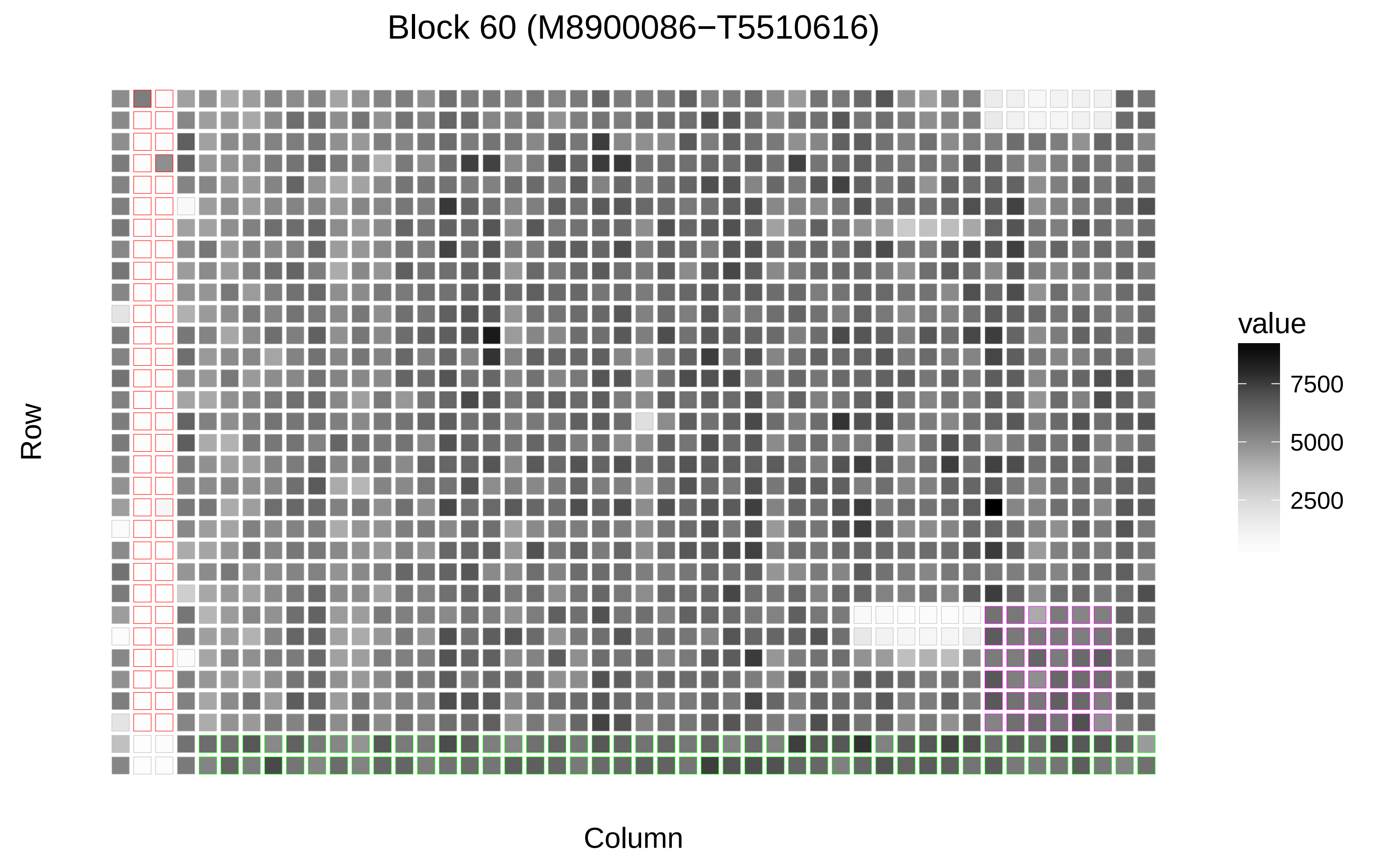

Block 60 (M8900086−T5510616)
Row
Column
value
7500
5000
2500

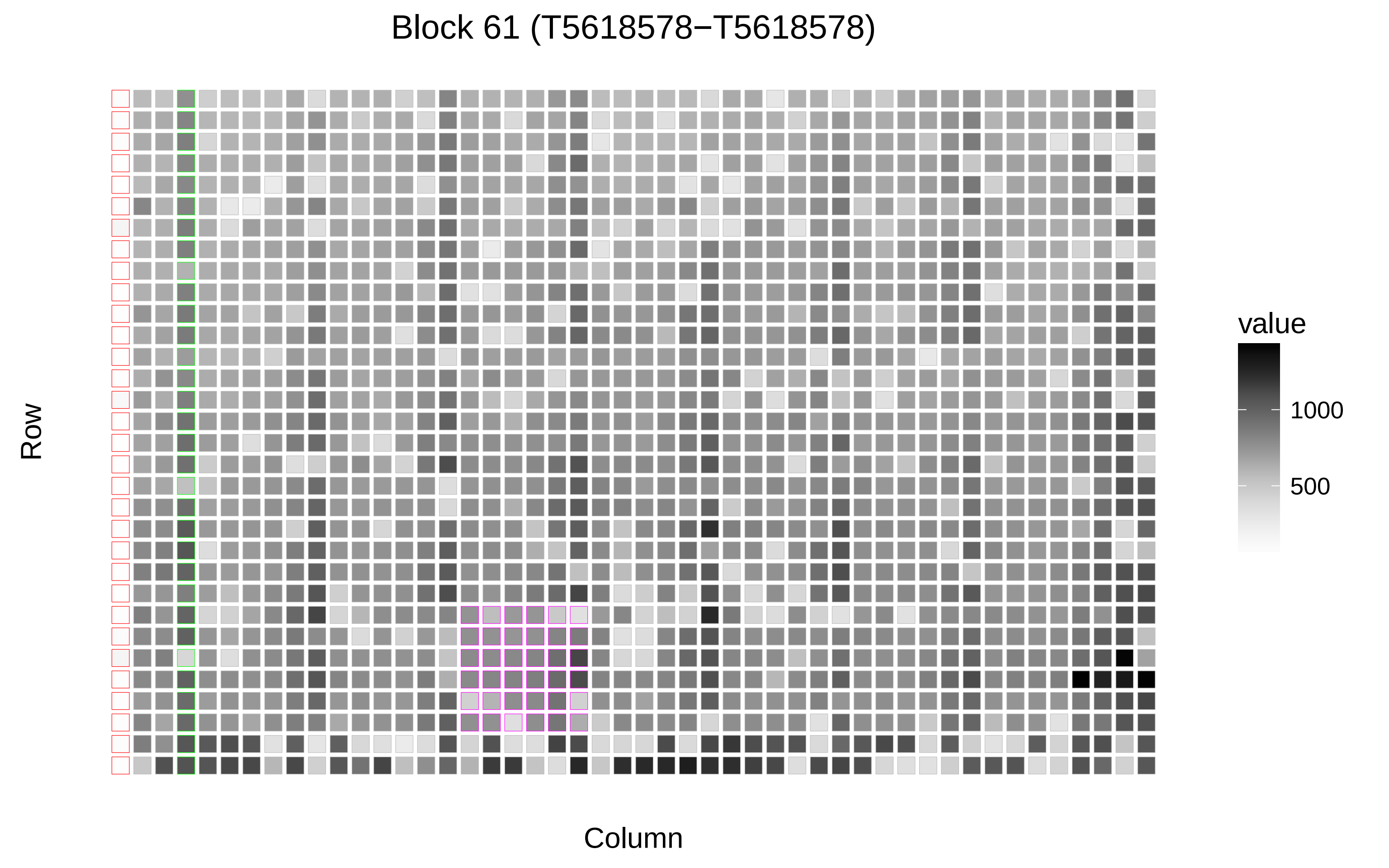

Block 61 (T5618578−T5618578)
Row
Column
value
1000
500

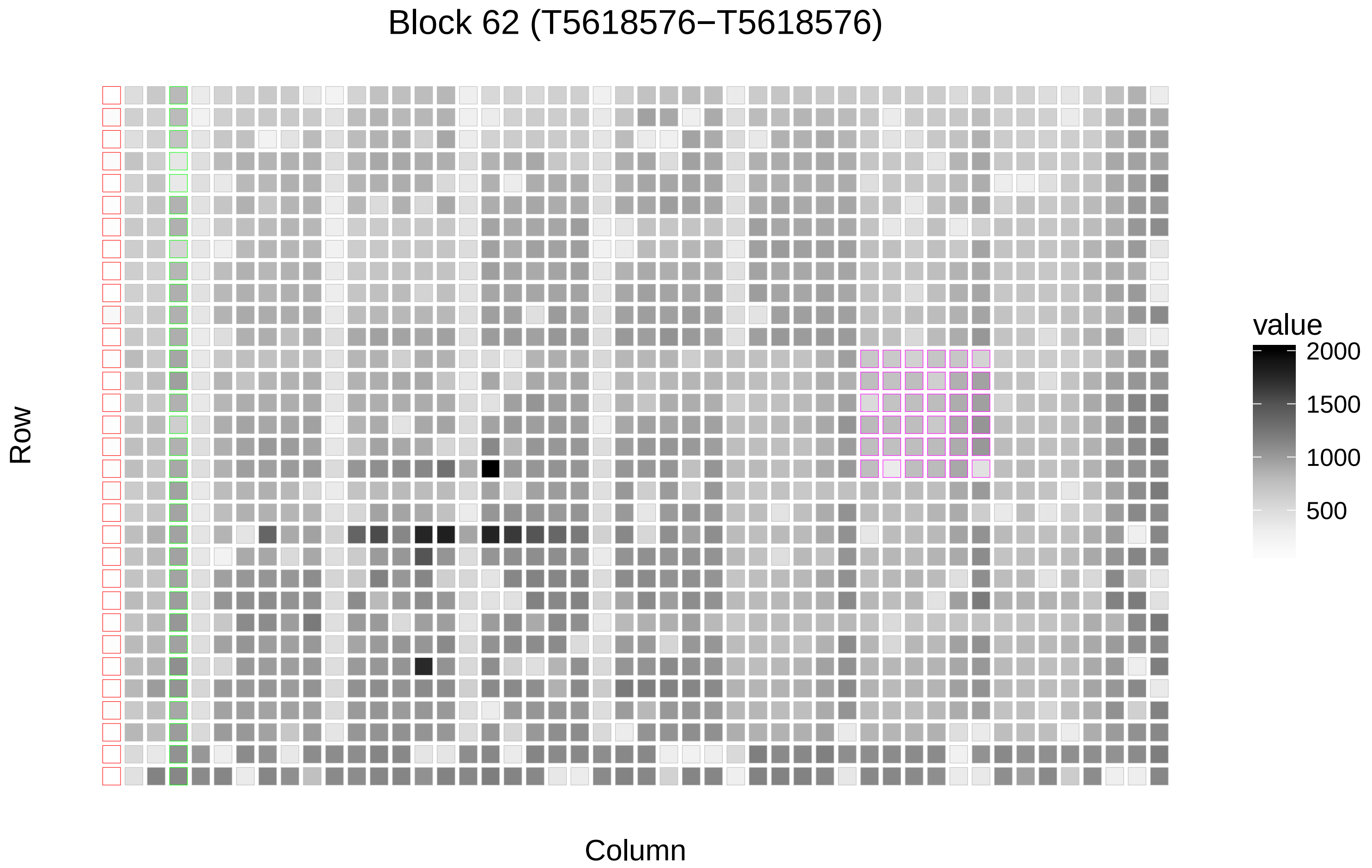
Block 62 (T5618576−T5618576)
Row
Column
value
2000
1500
1000
500

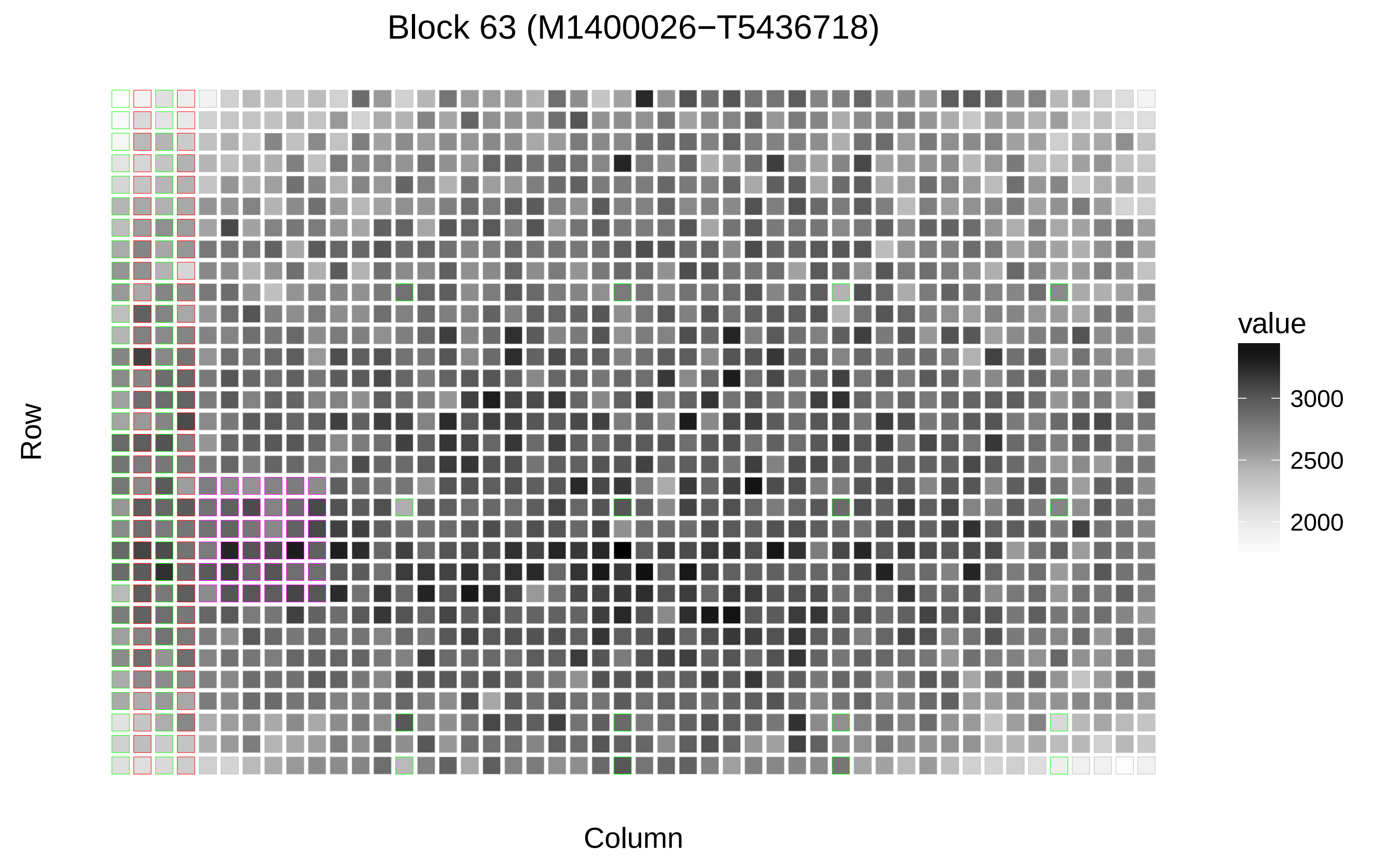
Block 63 (M1400026−T5436718)
Row
Column
value
3000
2500
2000

Block 64 (T5614964−T5614964)

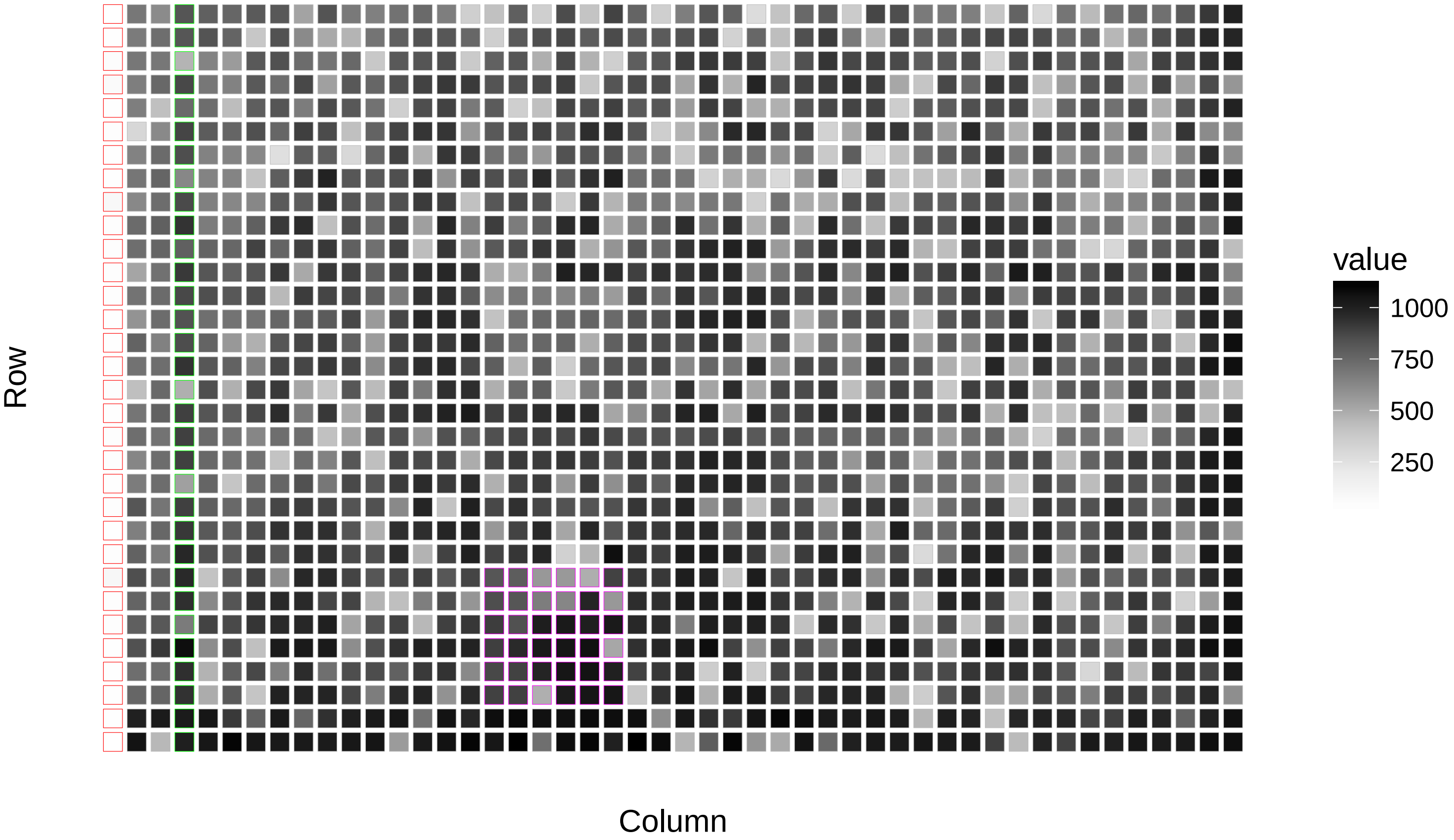

Block 65 (M1020010−T5571036)

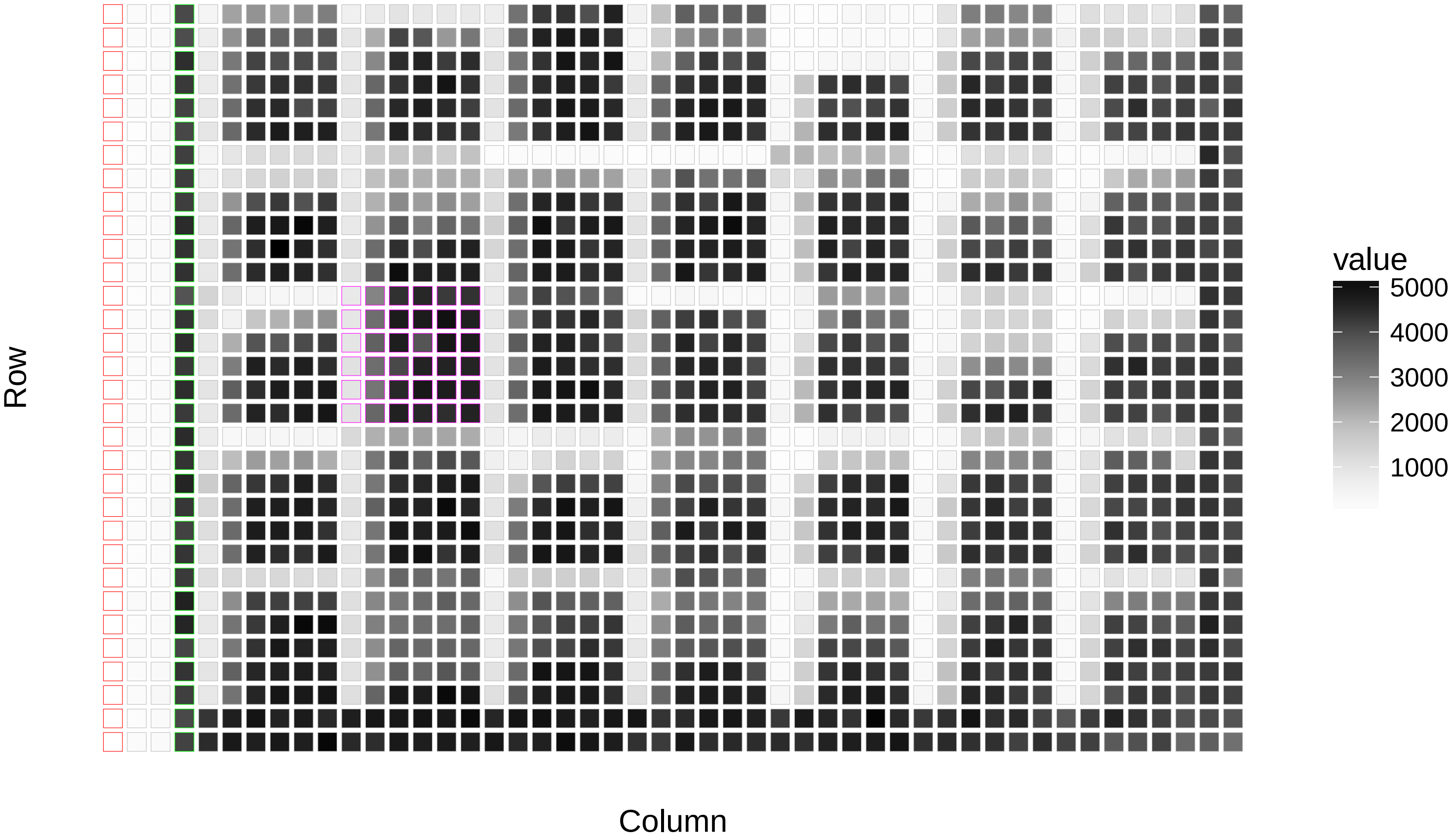

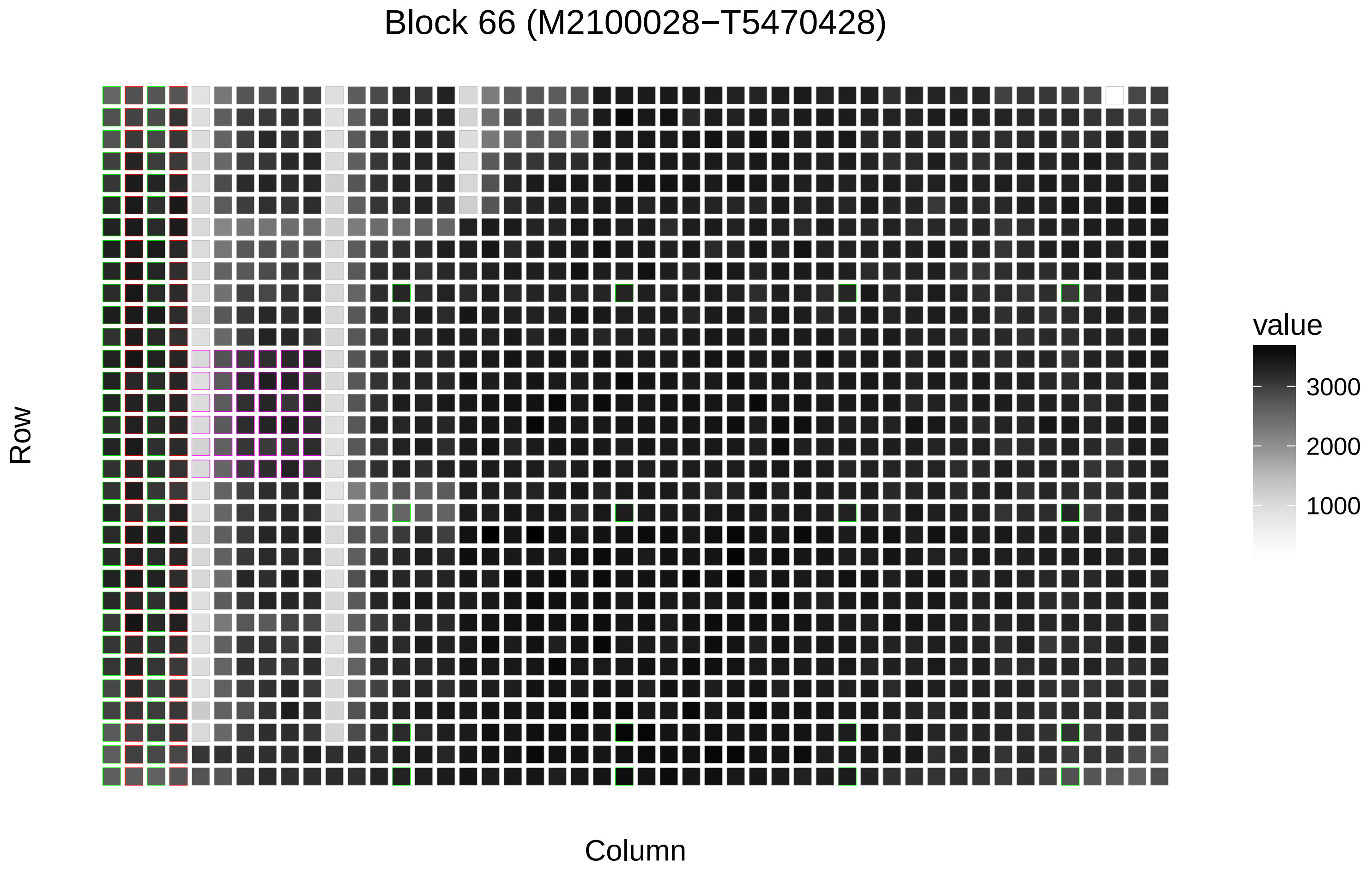

Block 66 (M2100028−T5470428)
Row
Column
value
3000
2000
1000

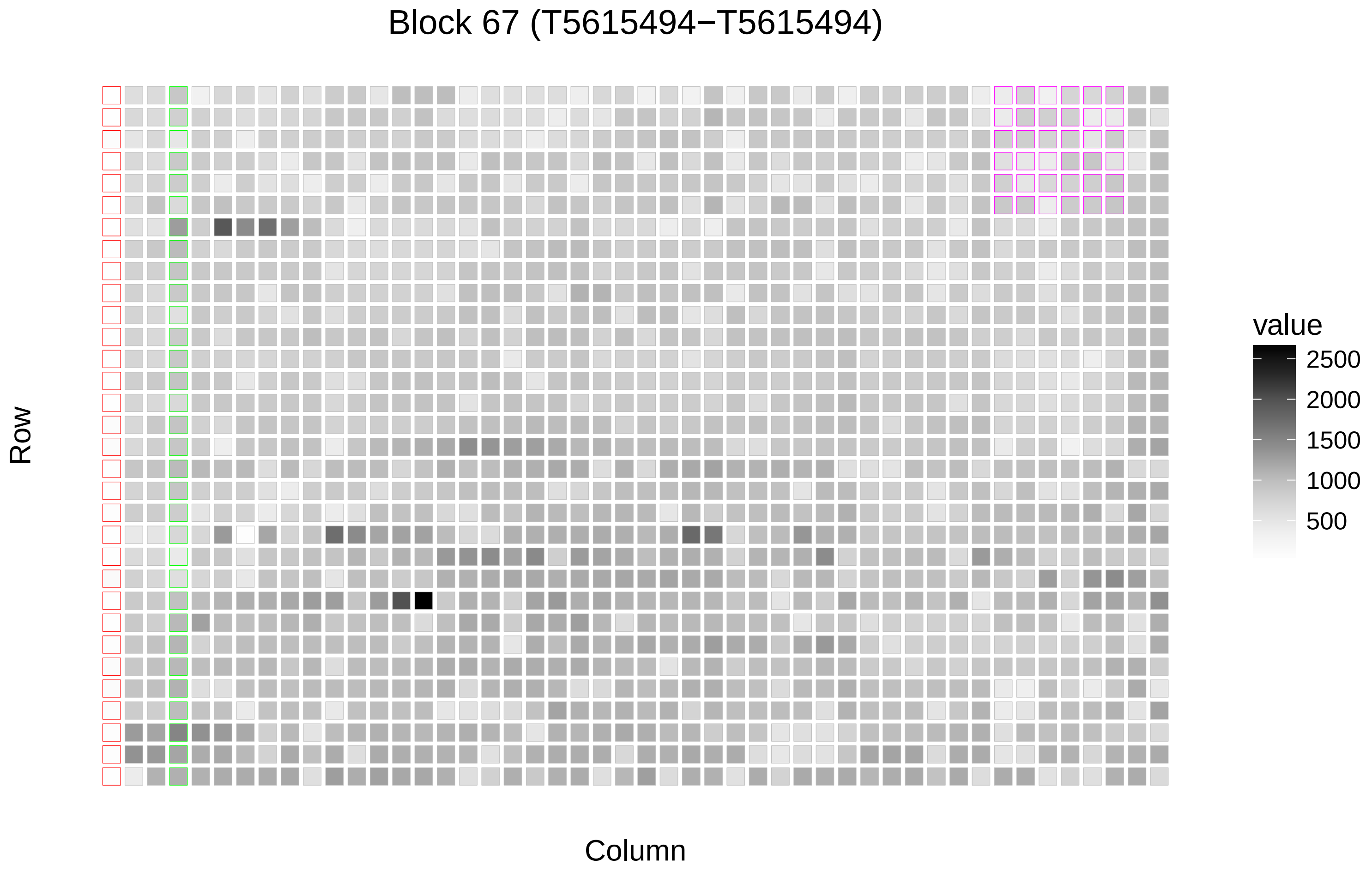

Block 67 (T5615494−T5615494)
Row
Column
value
2500
2000
1500
1000
500

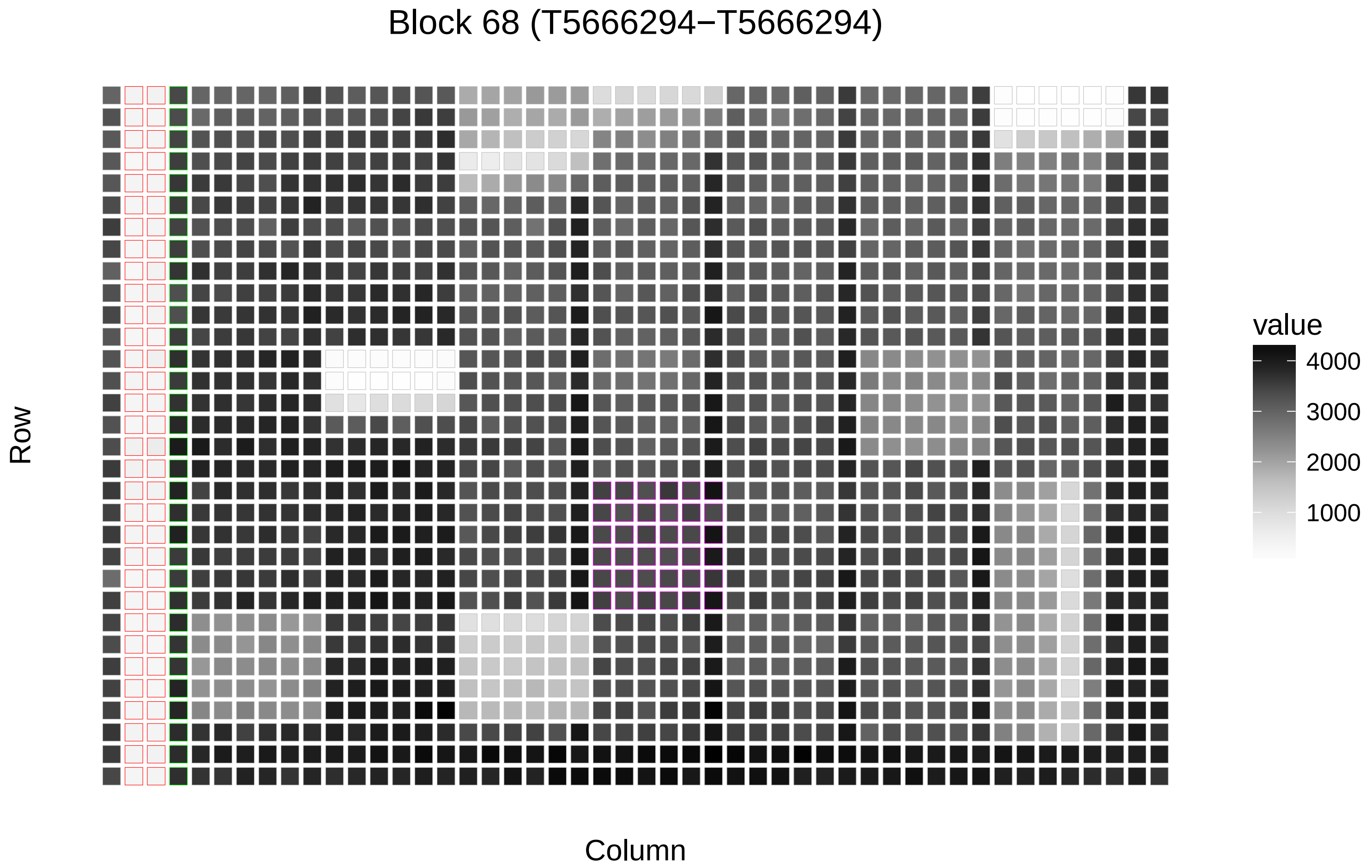
Block 68 (T5666294−T5666294)
Row
Column
value
4000
3000
2000
1000

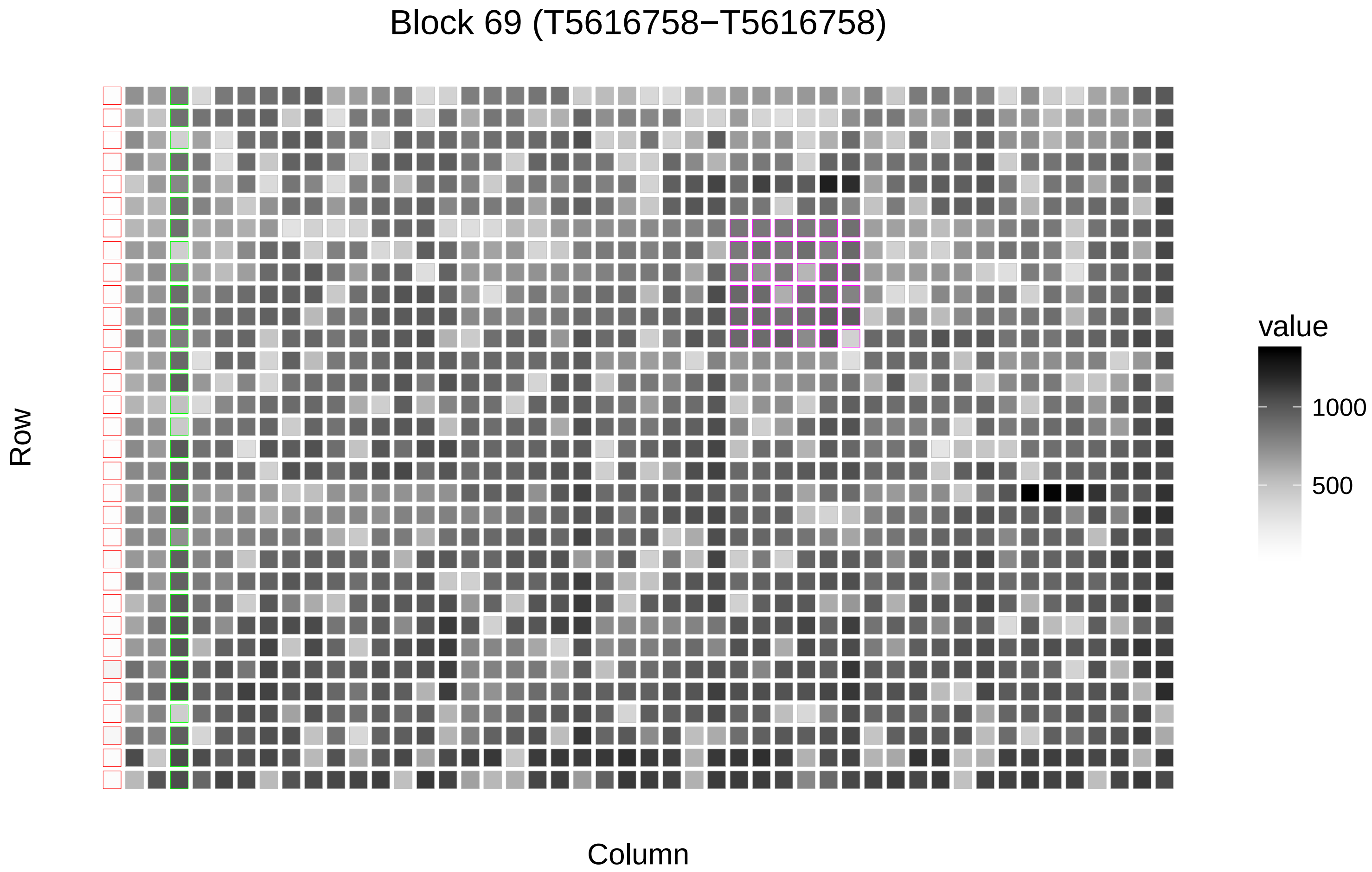
Block 69 (T5616758−T5616758)
Row
Column
value
1000
500

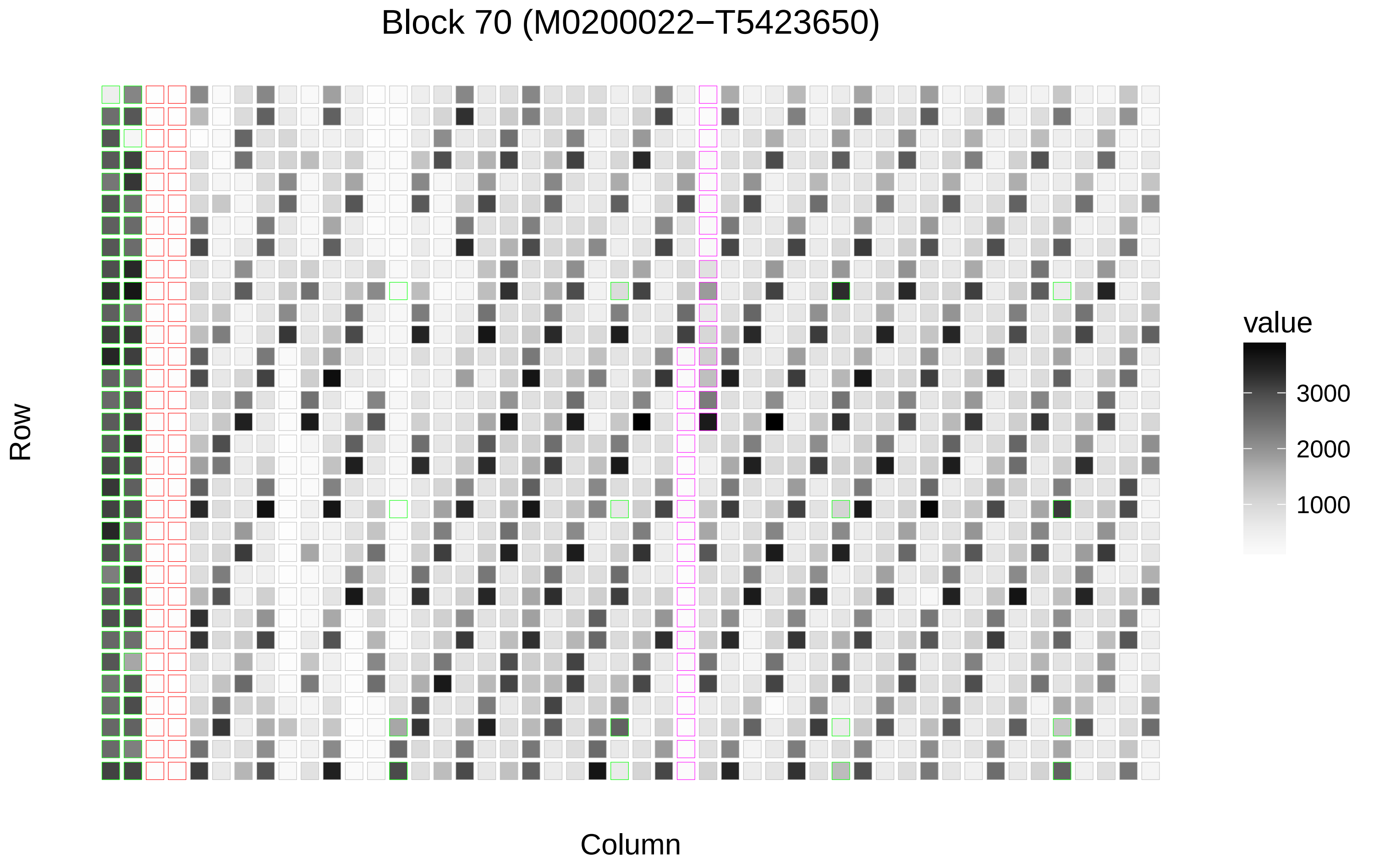
Block 70 (M0200022−T5423650)
Row
Column
value
3000
2000
1000

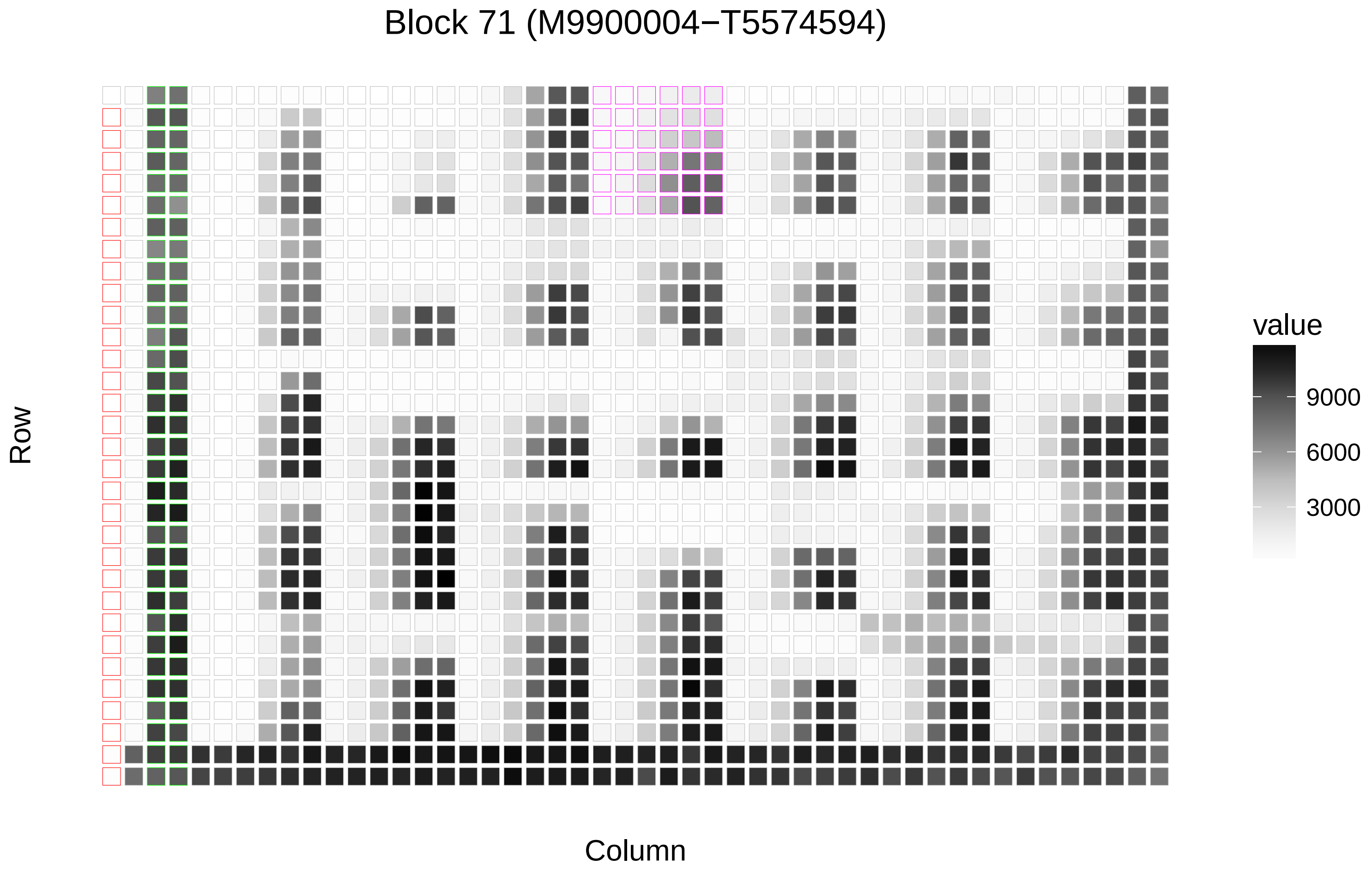
Block 71 (M9900004−T5574594)
Row
Column
value
9000
6000
3000

Block 72 (M8900010−T5510708)

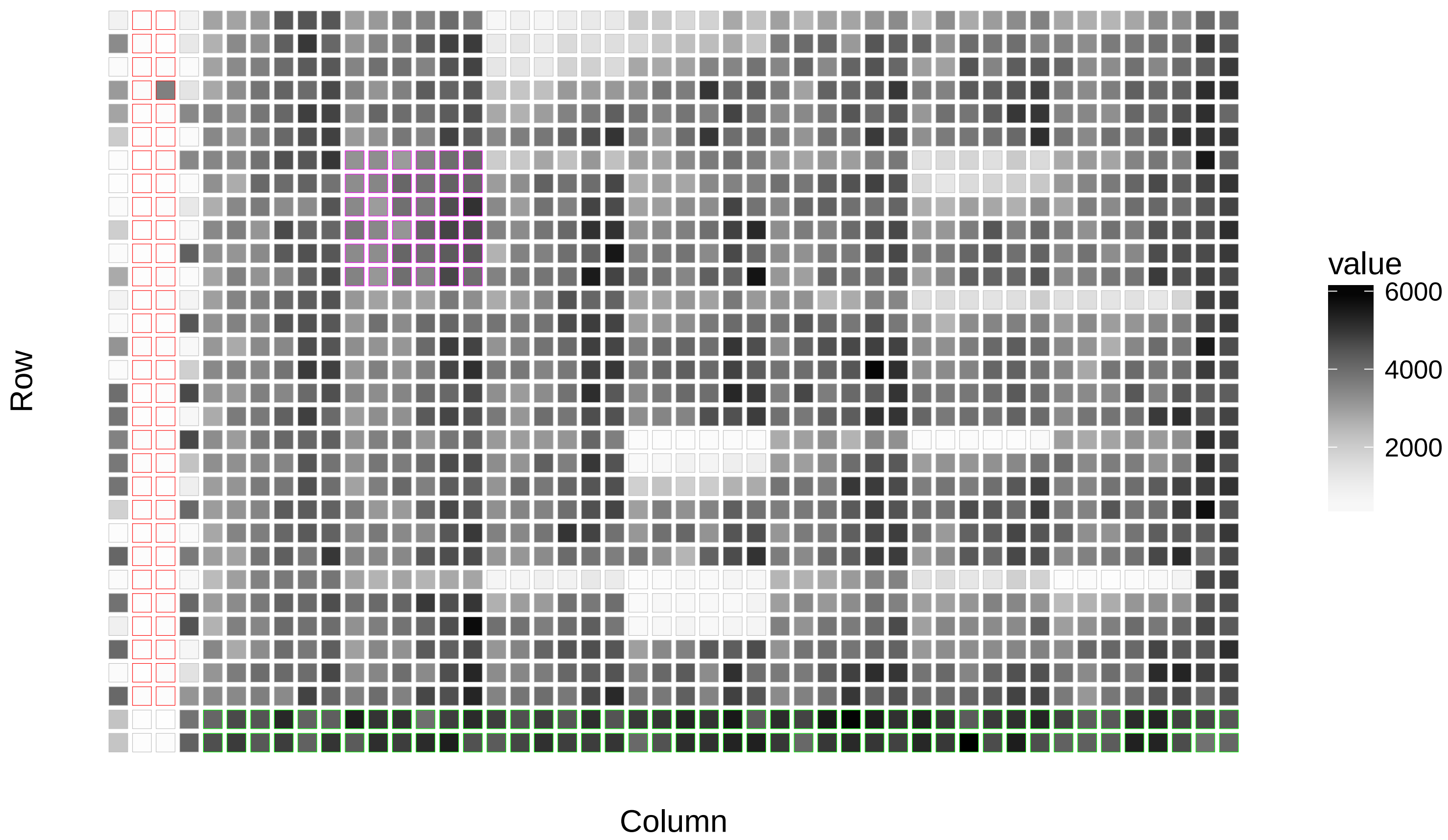

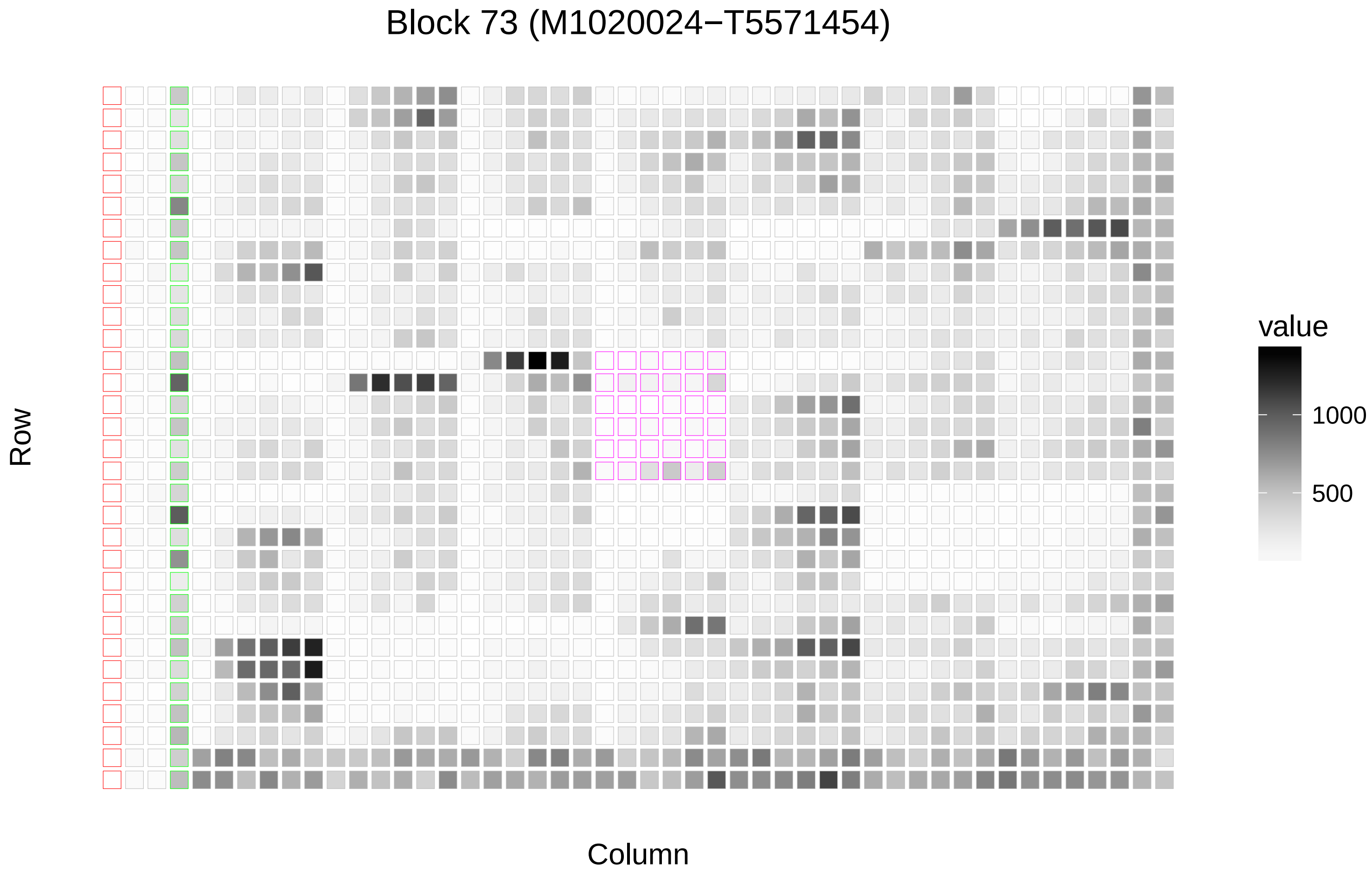

Block 73 (M1020024−T5571454)
Row
Column
value
1000
500

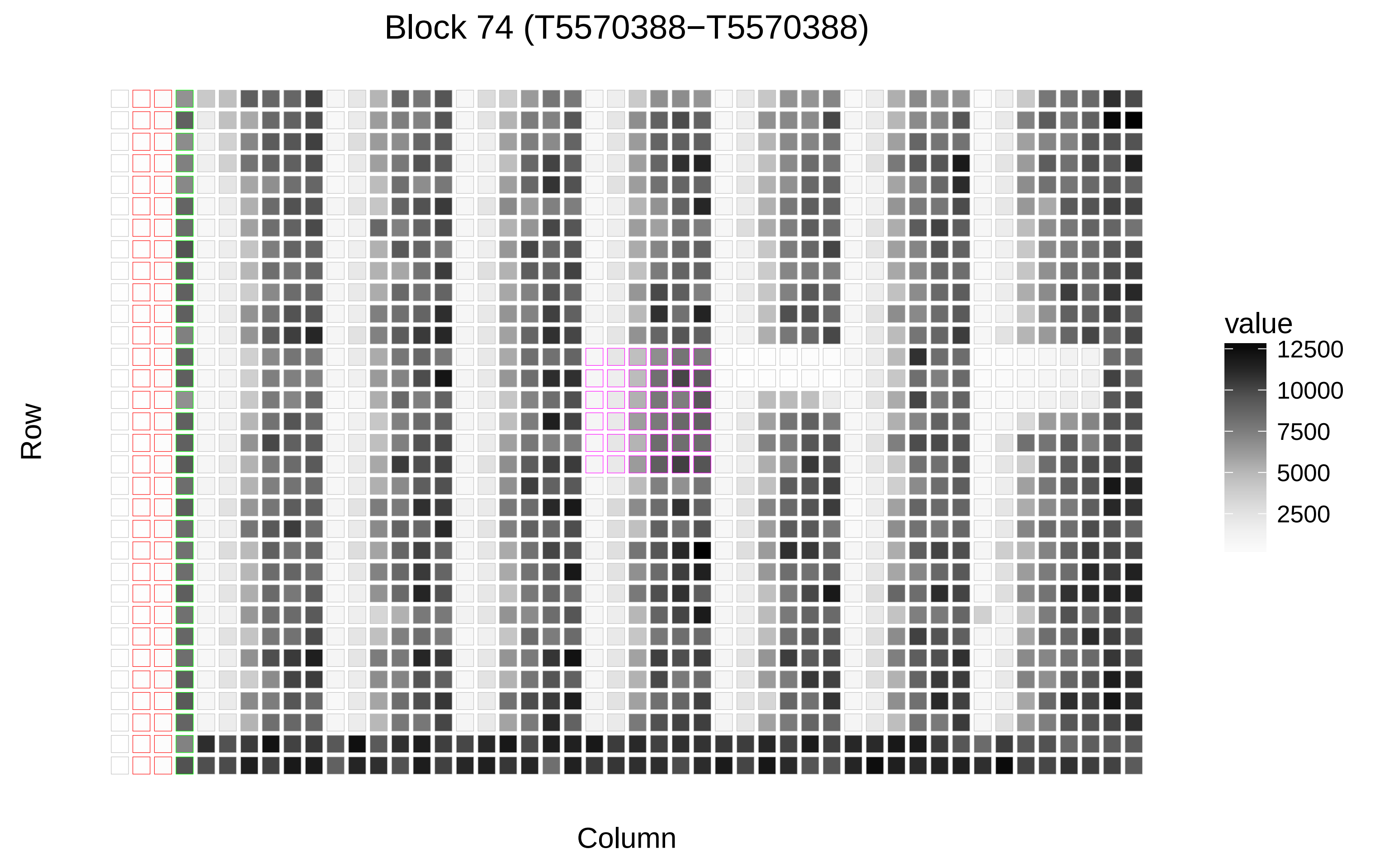
Block 74 (T5570388−T5570388)
Row
Column
value
12500
10000
7500
5000
2500

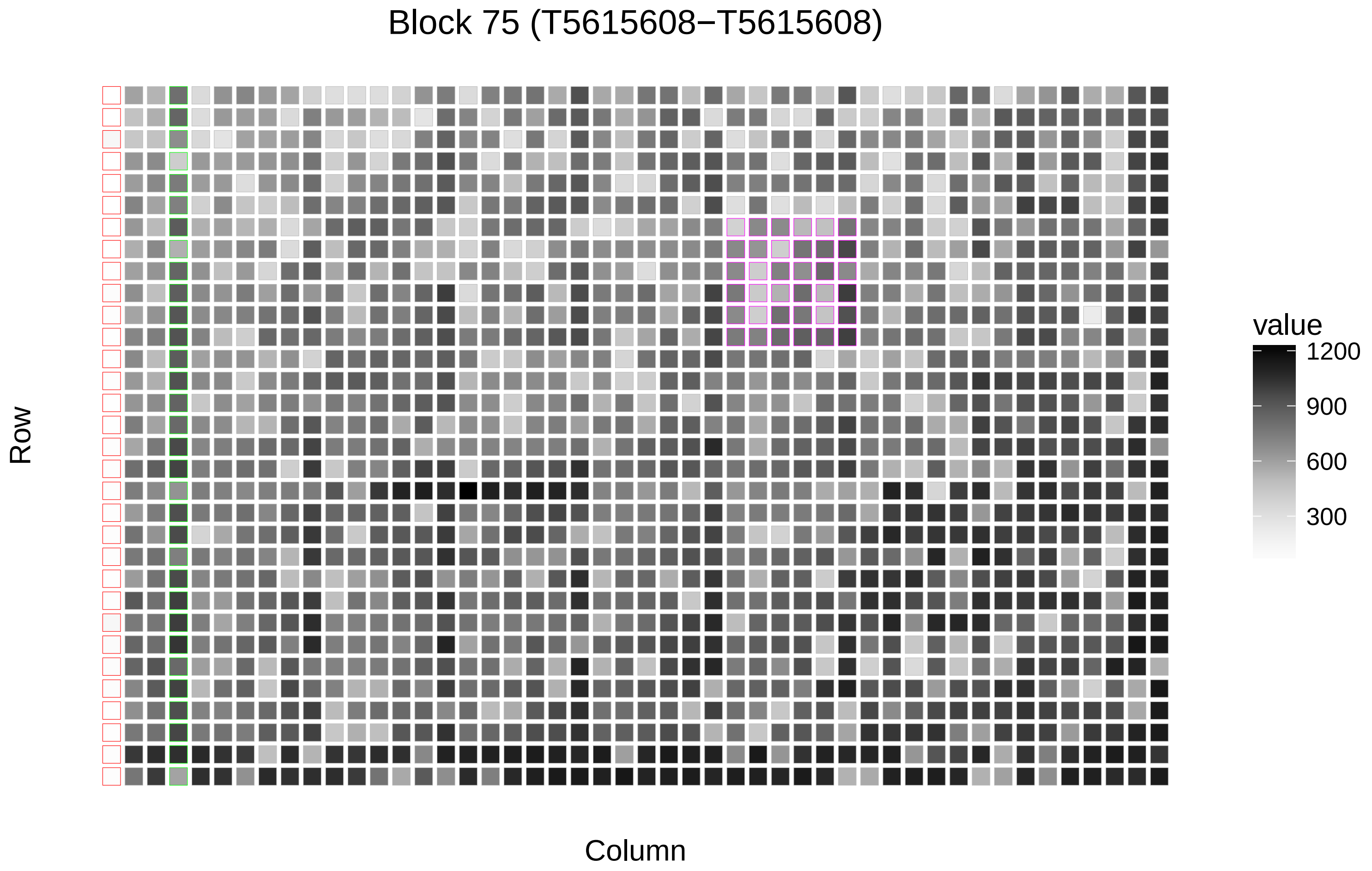
Block 75 (T5615608−T5615608)
Row
Column
value
1200
900
600
300

Block 76 (T5574276−T5574276)

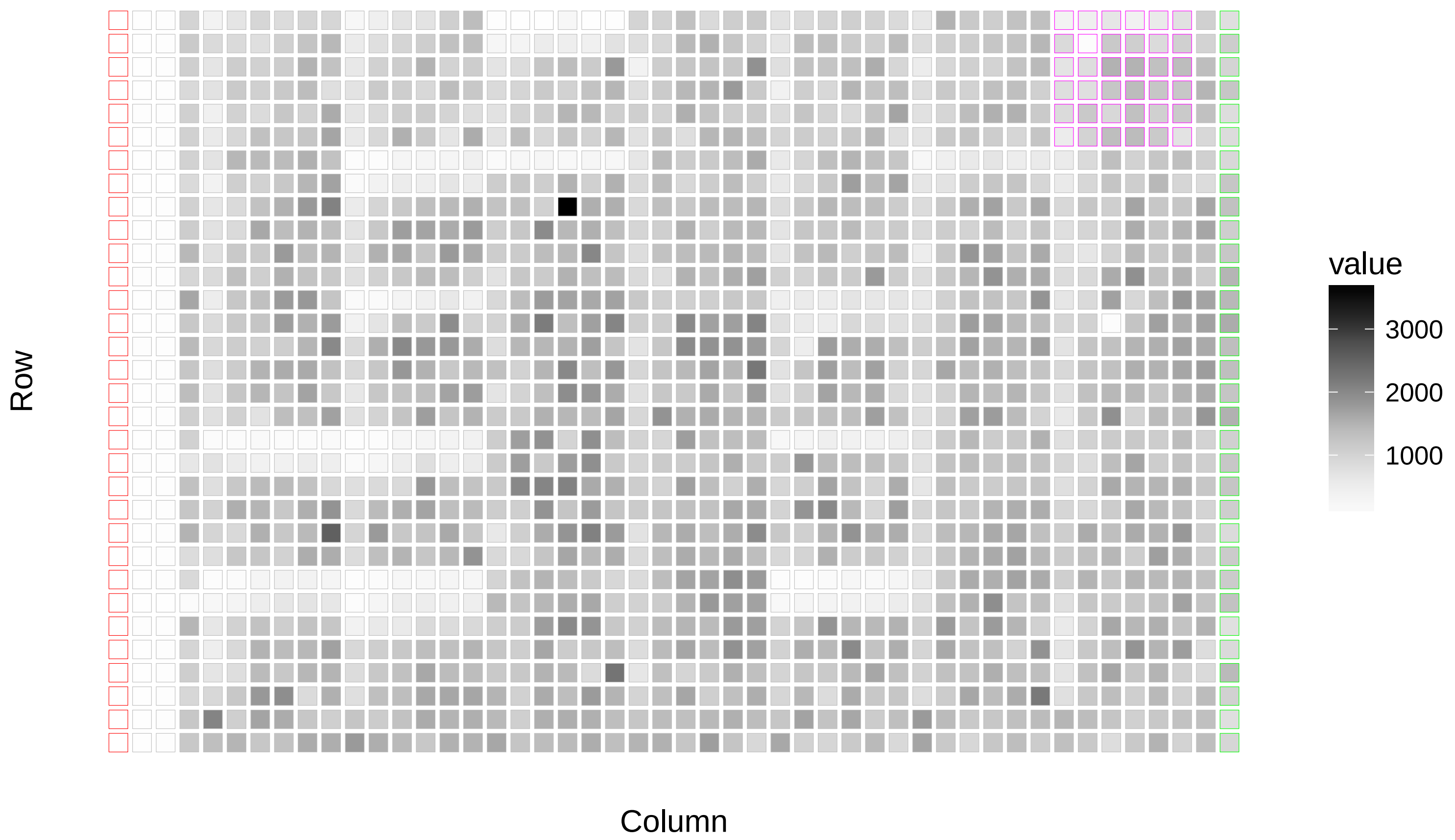

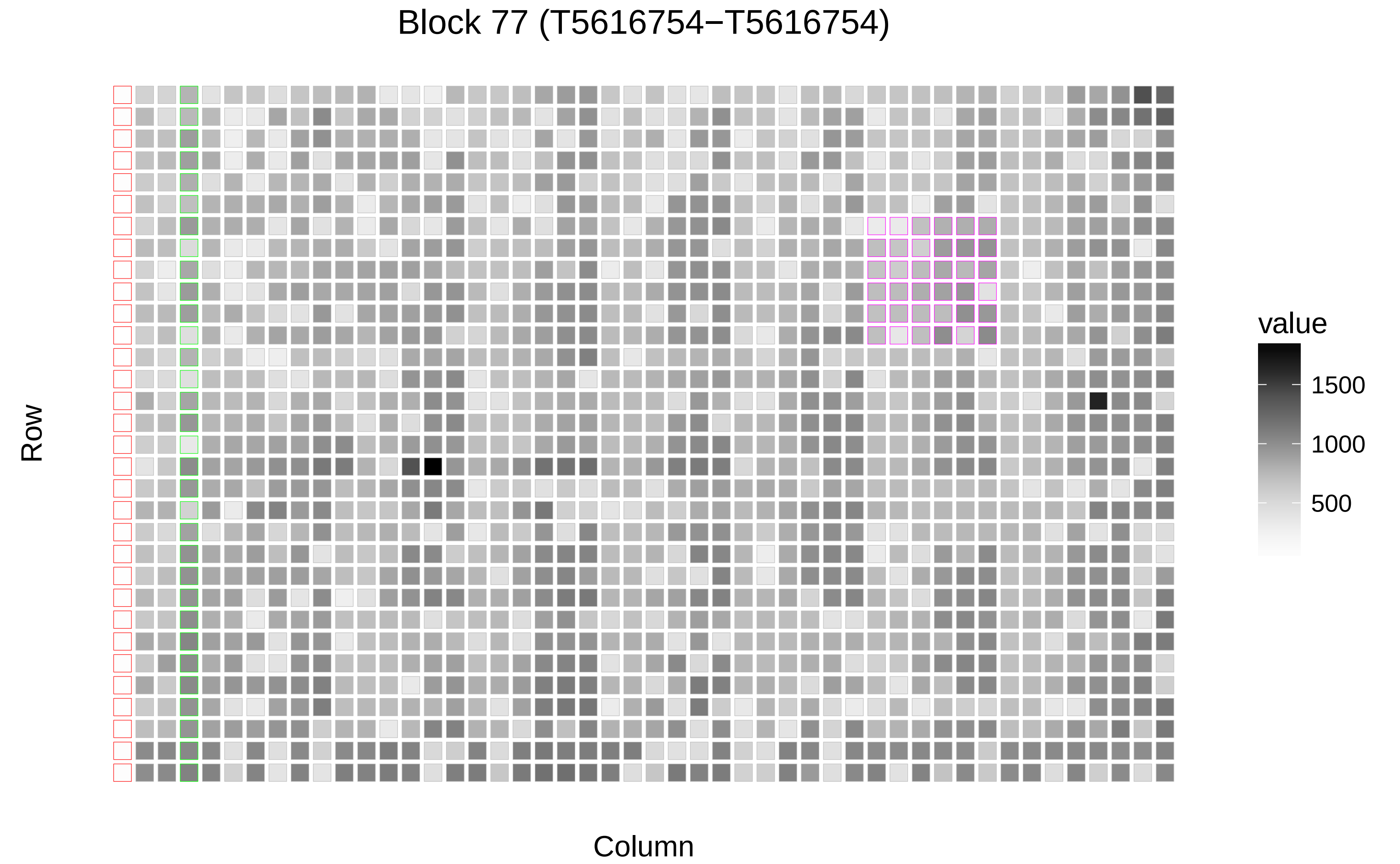
Block 77 (T5616754−T5616754)
Row
Column
value
1500
1000
500

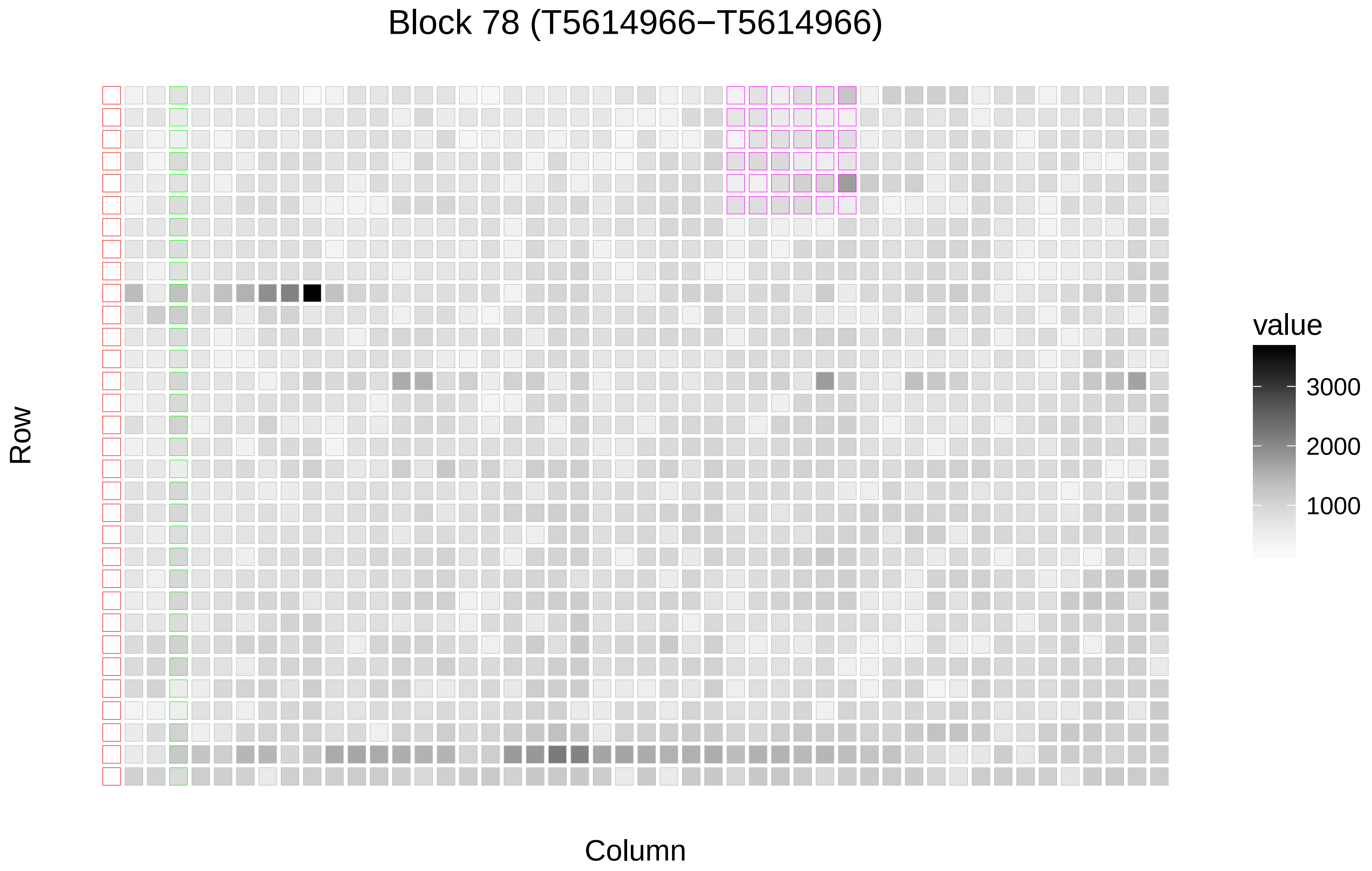
Block 78 (T5614966−T5614966)
Row
Column
value
3000
2000
1000

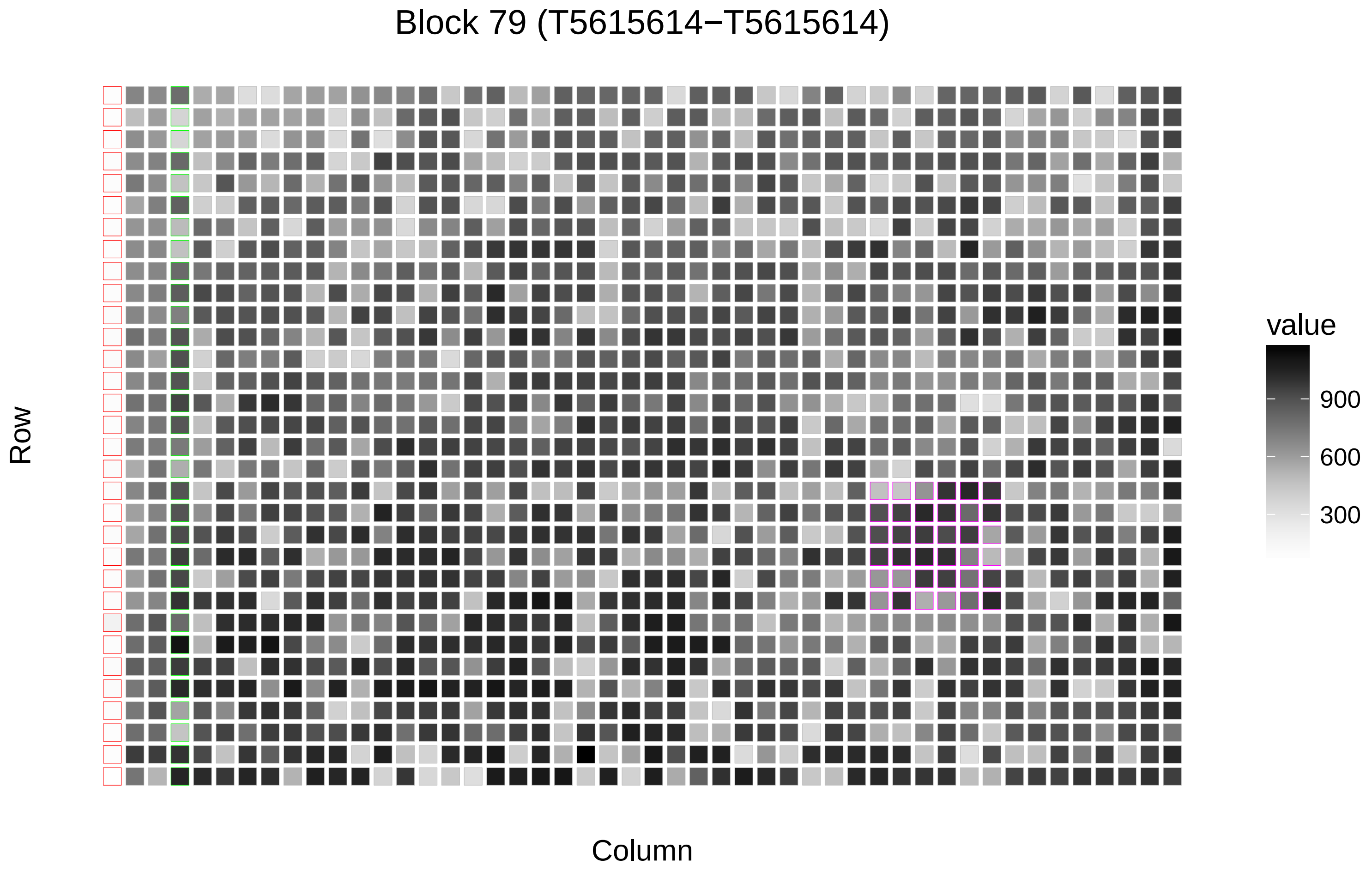
Block 79 (T5615614−T5615614)
Row
Column
value
900
600
300

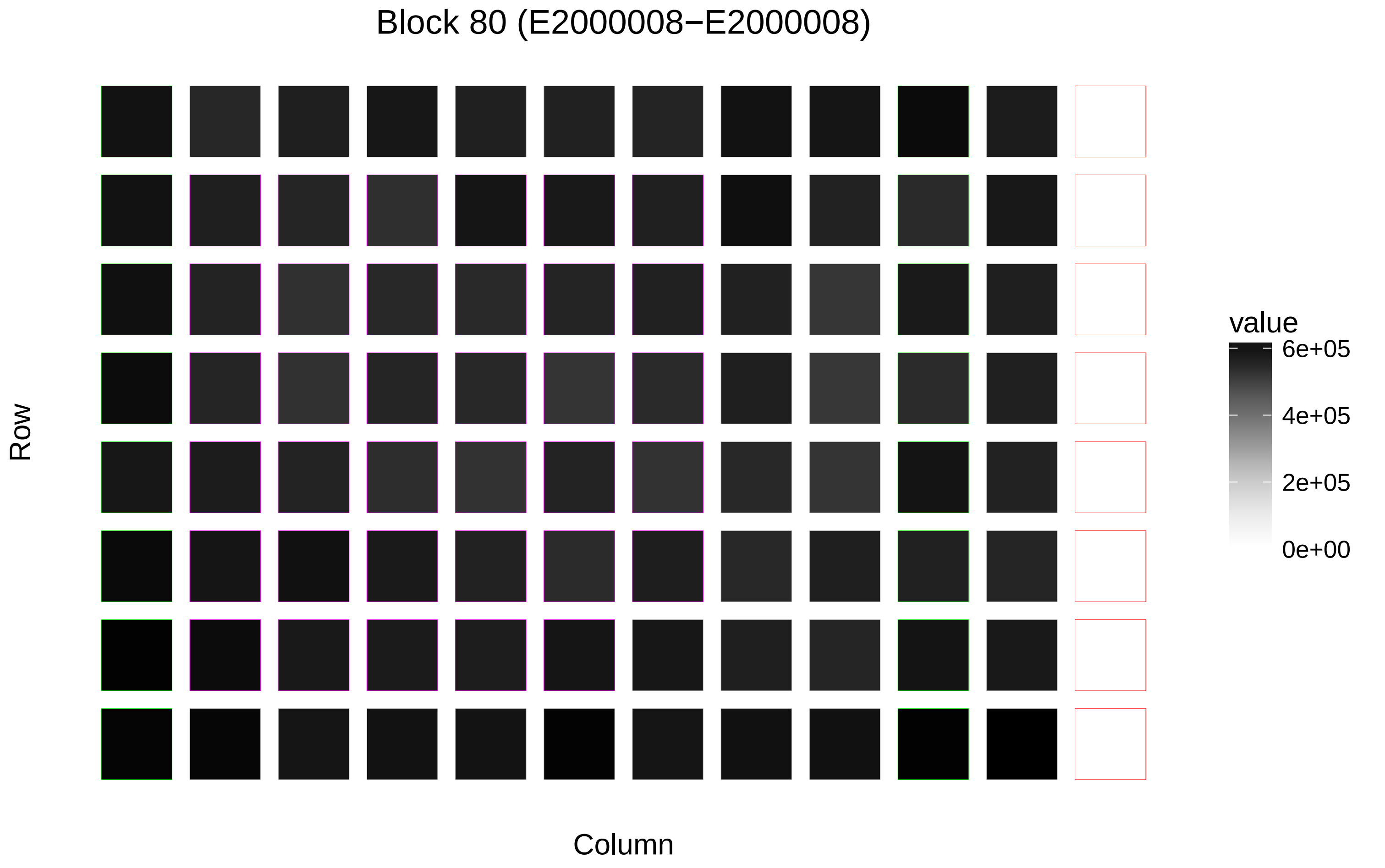
Block 80 (E2000008−E2000008)
Row
Column
value
6e+05
4e+05
2e+05
0e+00

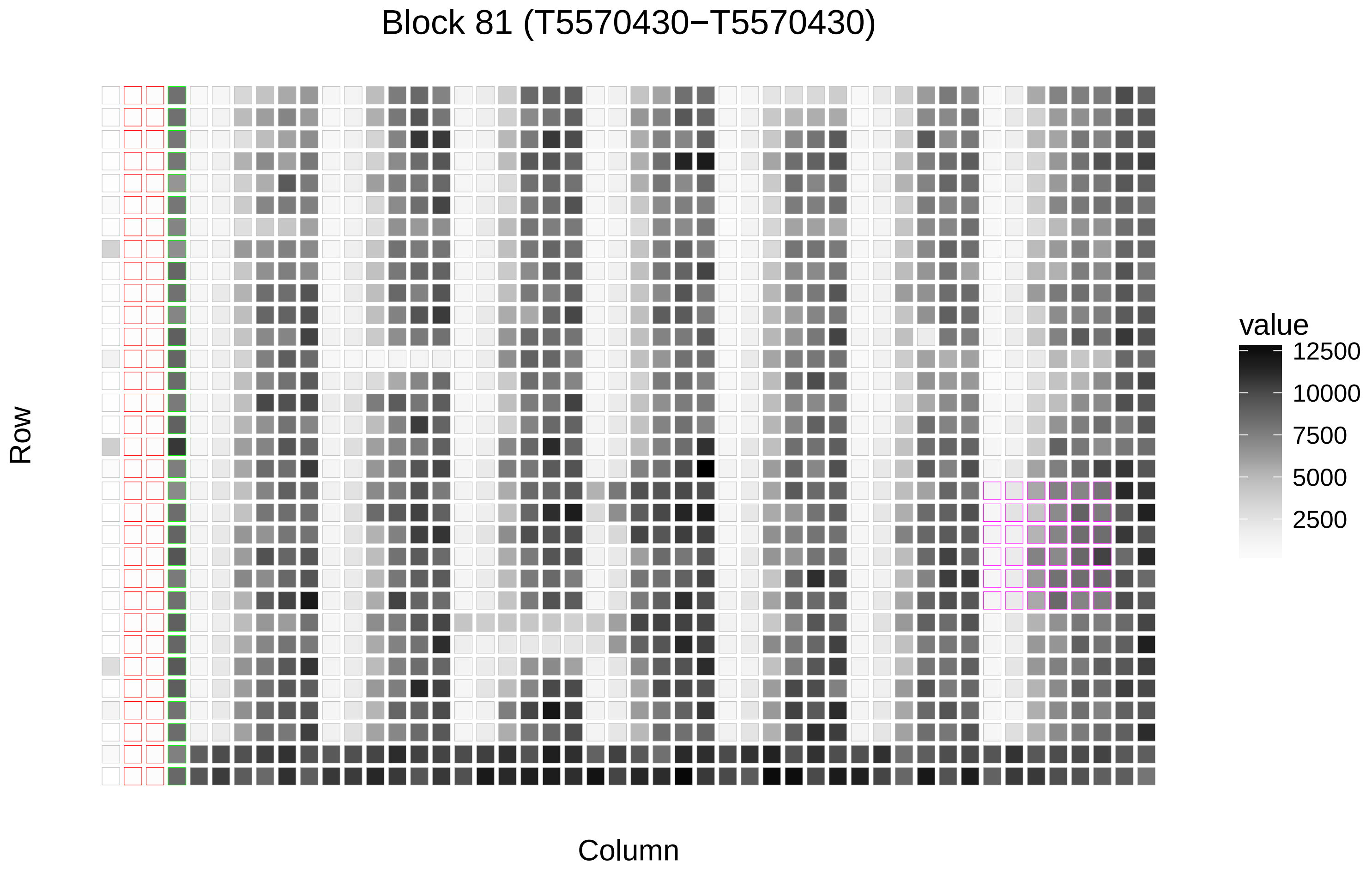
Block 81 (T5570430−T5570430)
Row
Column
value
12500
10000
7500
5000
2500

Block 82 (T5574270−T5574270)

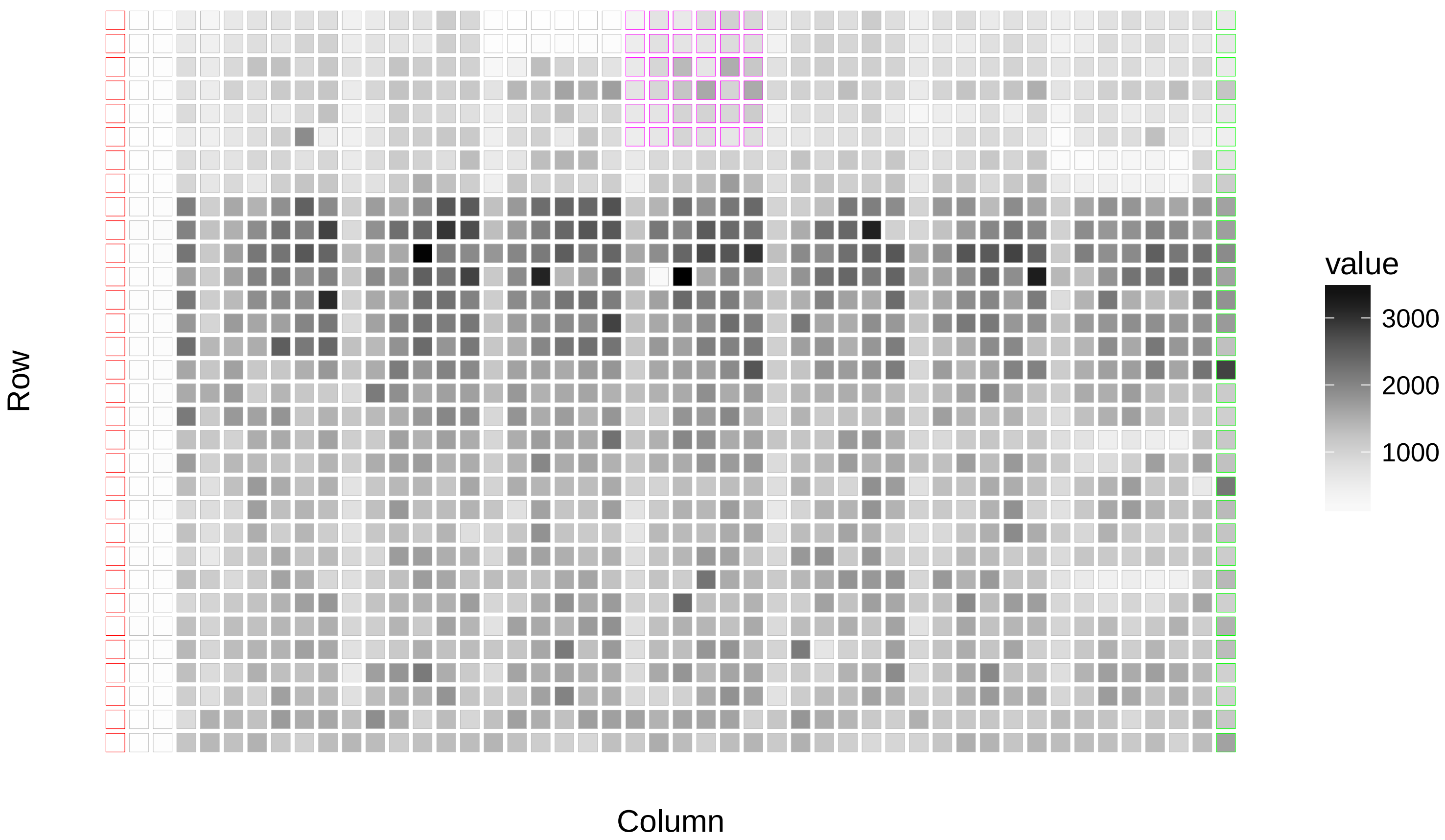

Block 83 (T5666306−T5666306)

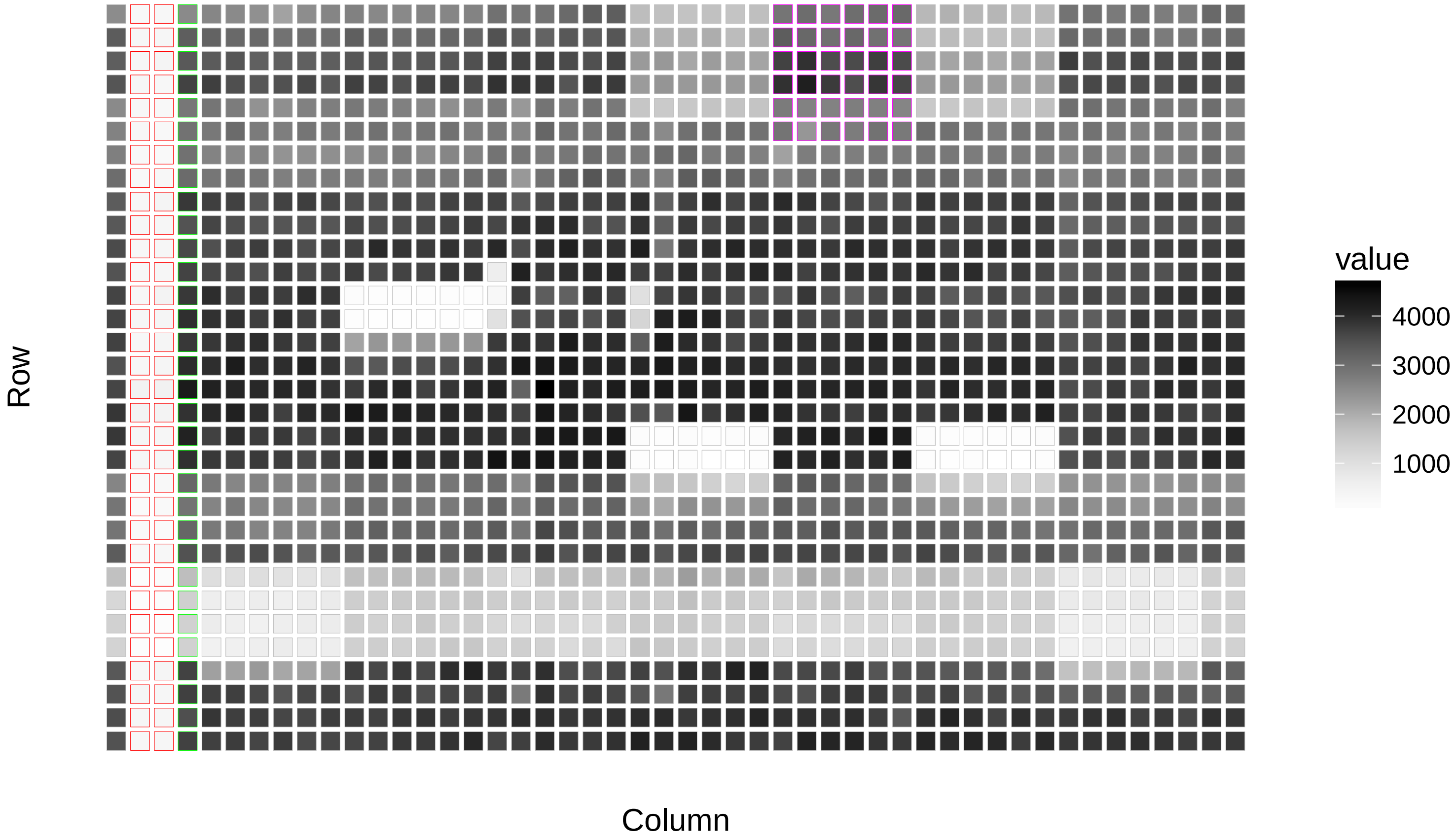

Block 84 (M1020014−T5571464)

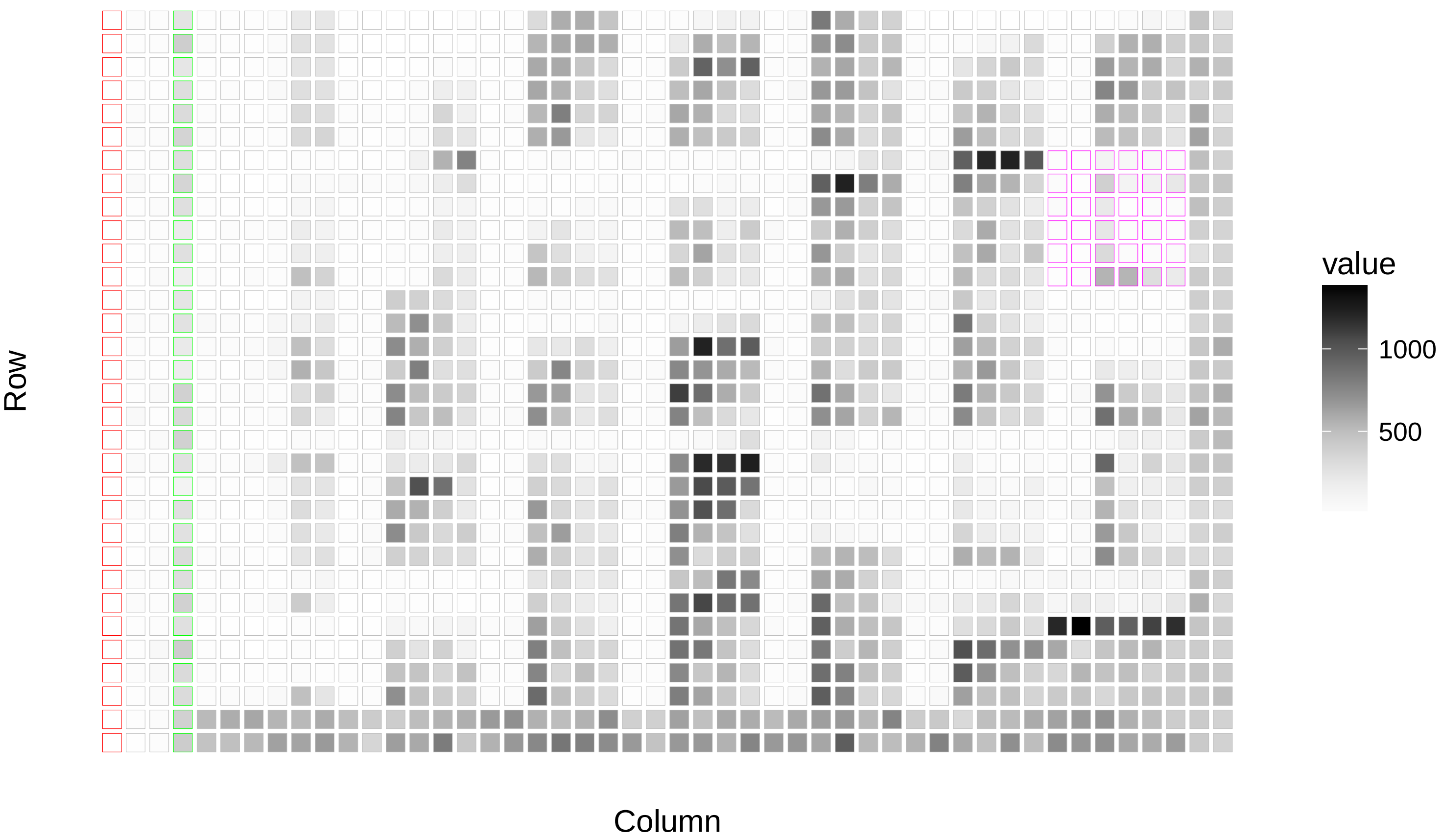

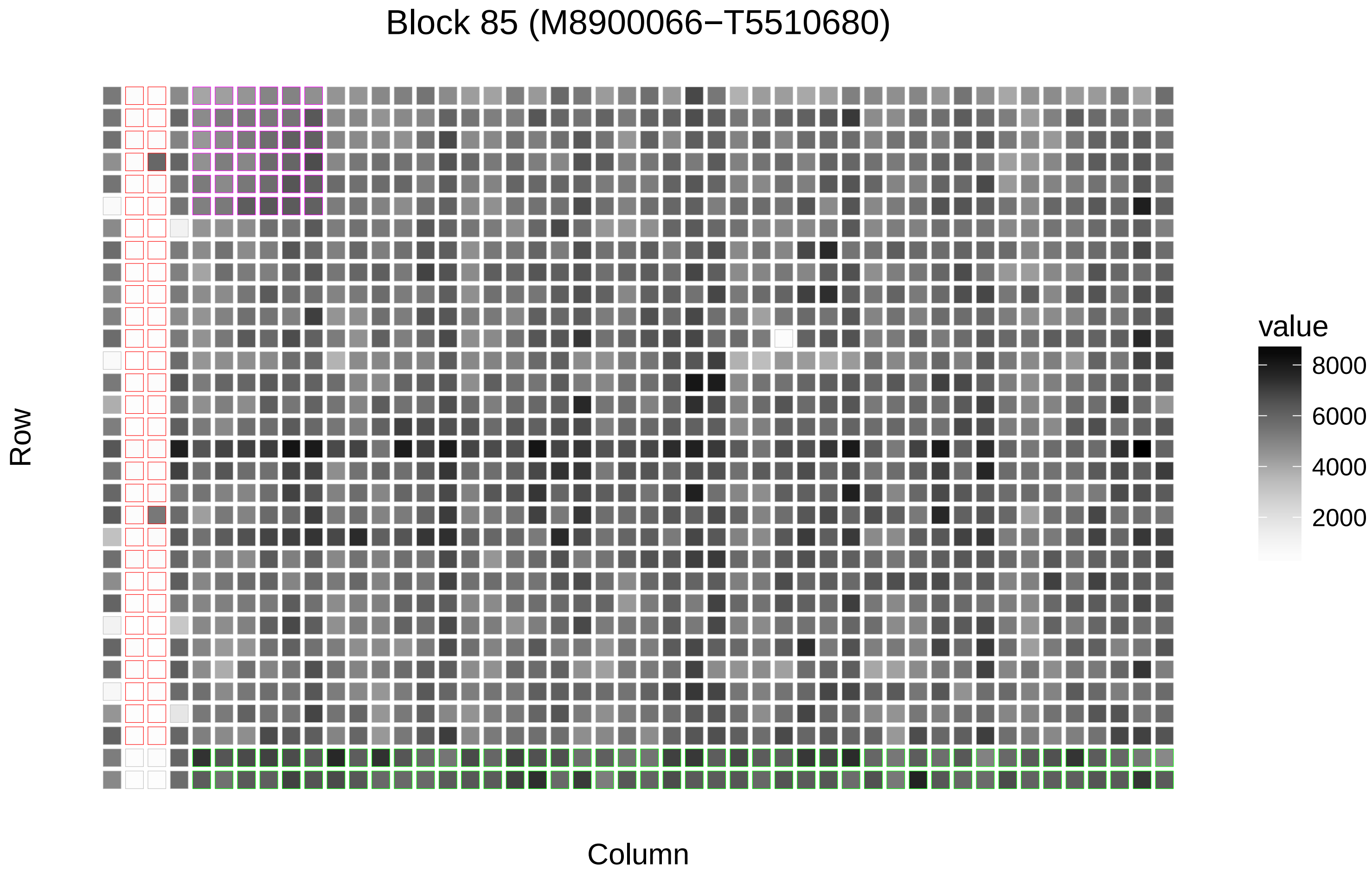

Block 85 (M8900066−T5510680)
Row
Column
value
8000
6000
4000
2000

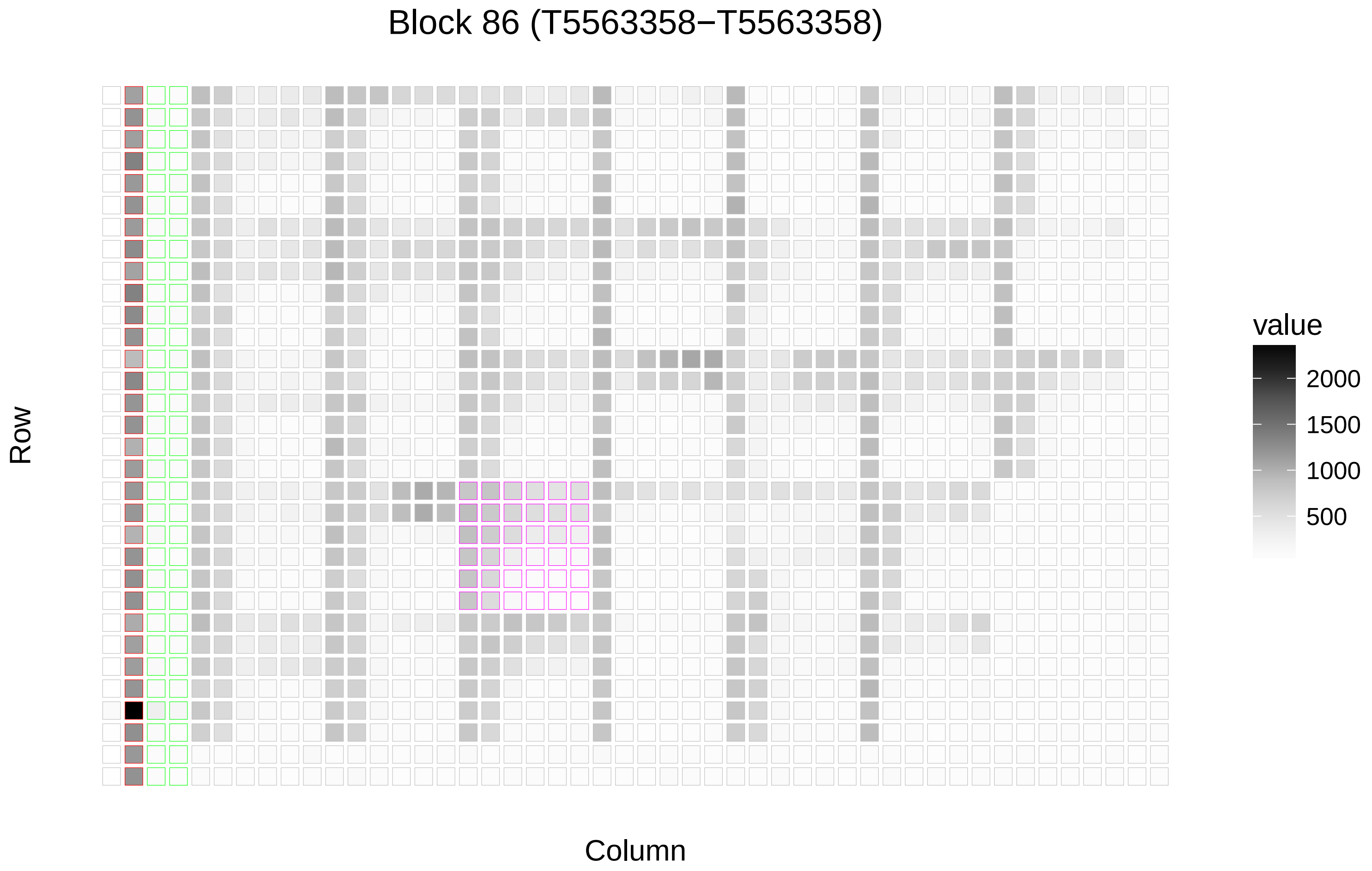

Block 86 (T5563358−T5563358)
Row
Column
value
2000
1500
1000
500

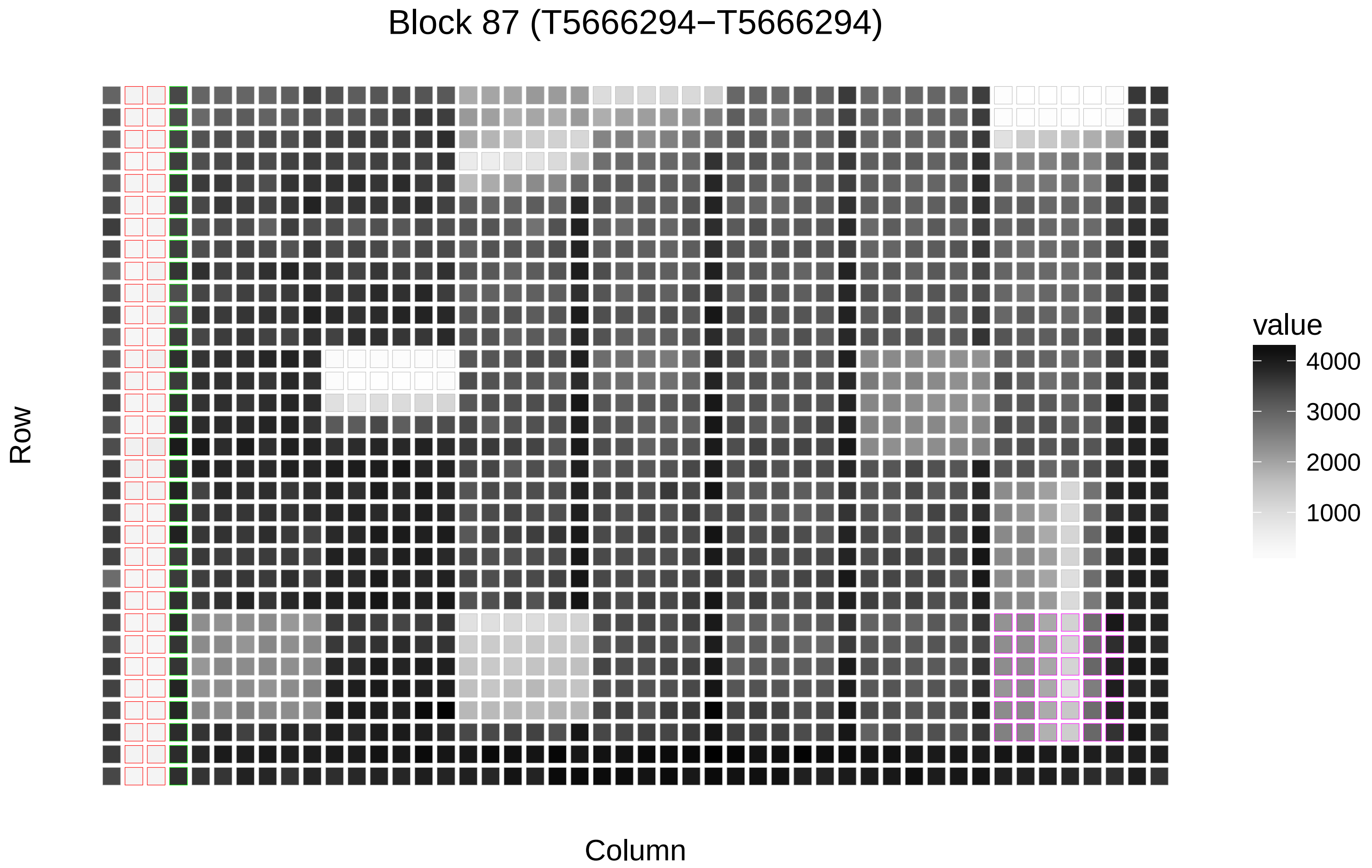

Block 87 (T5666294–T5666294)
Row
Column
value
4000
3000
2000
1000

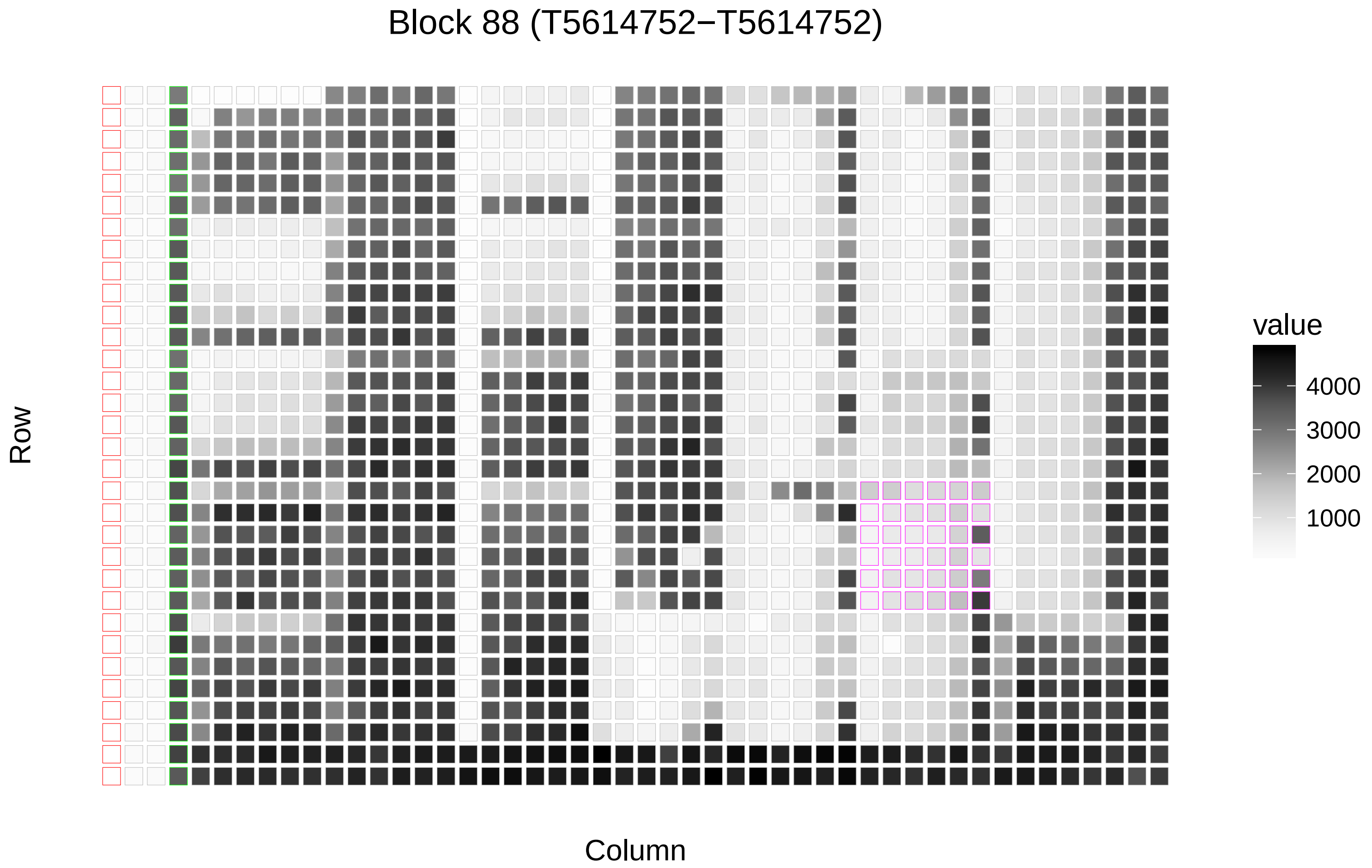
Block 88 (T5614752−T5614752)
Row
Column
value
4000
3000
2000
1000

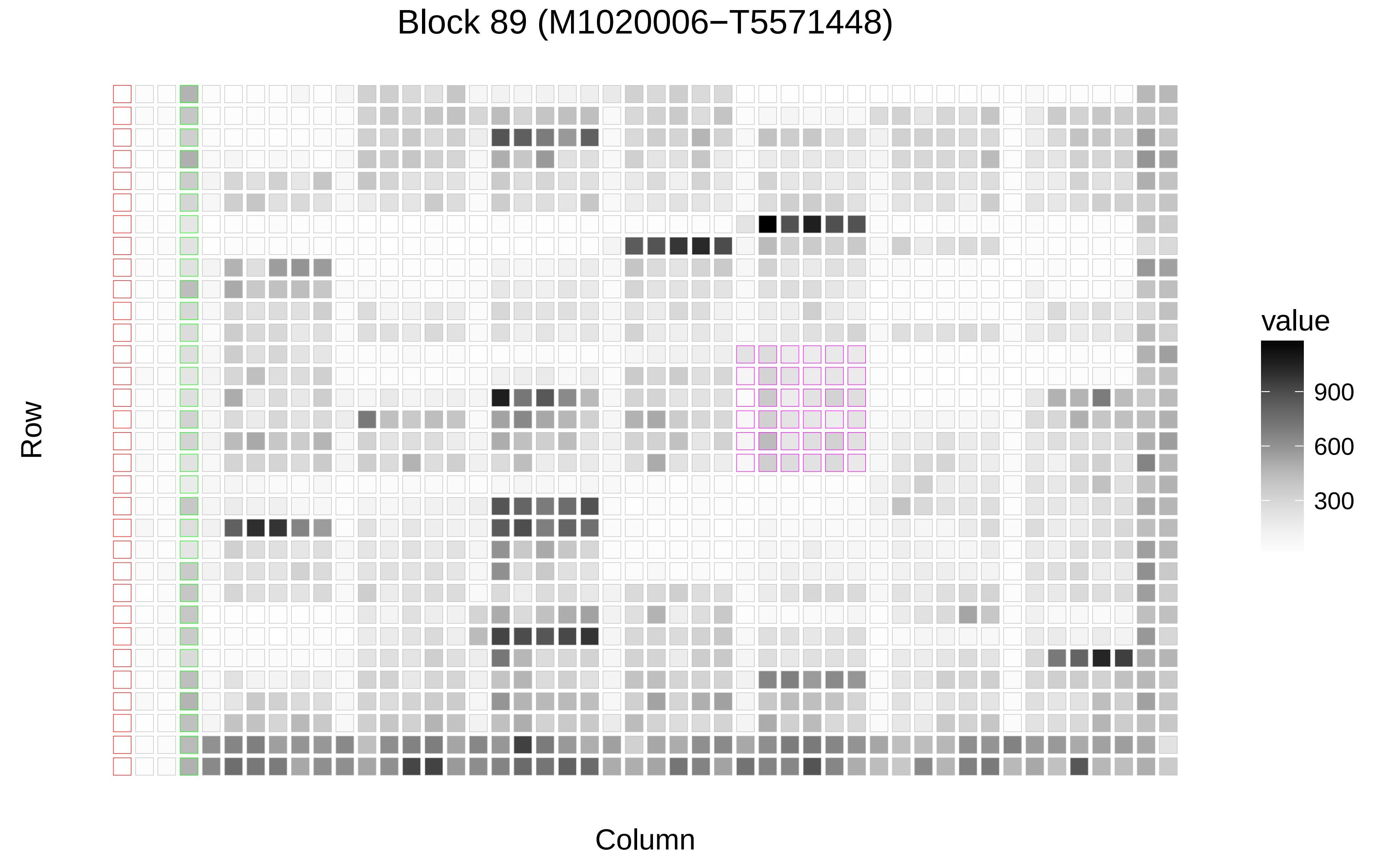
Block 89 (M1020006−T5571448)
Row
Column
value
900
600
300

Block 90 (M0300042−T5423700)

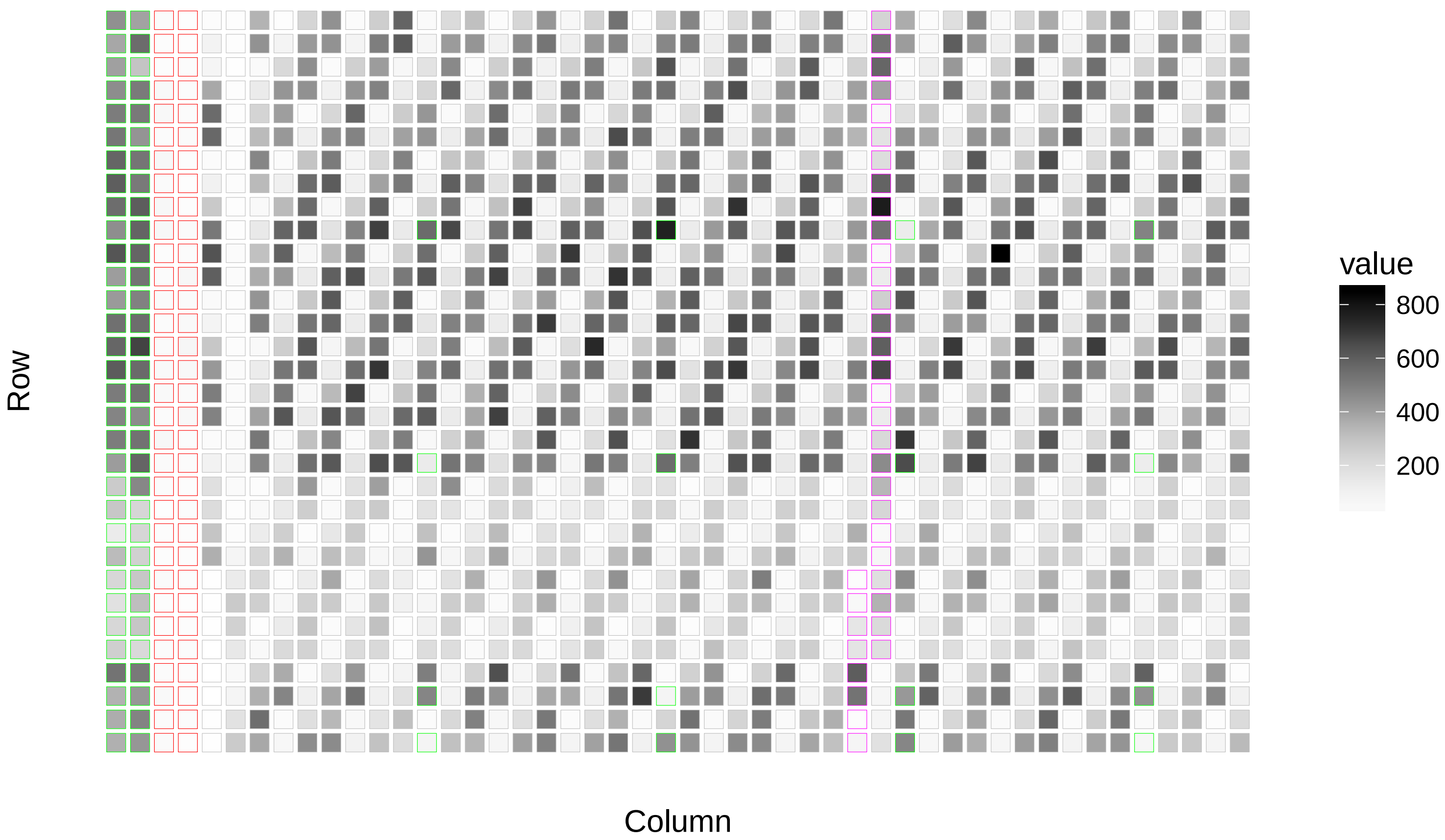

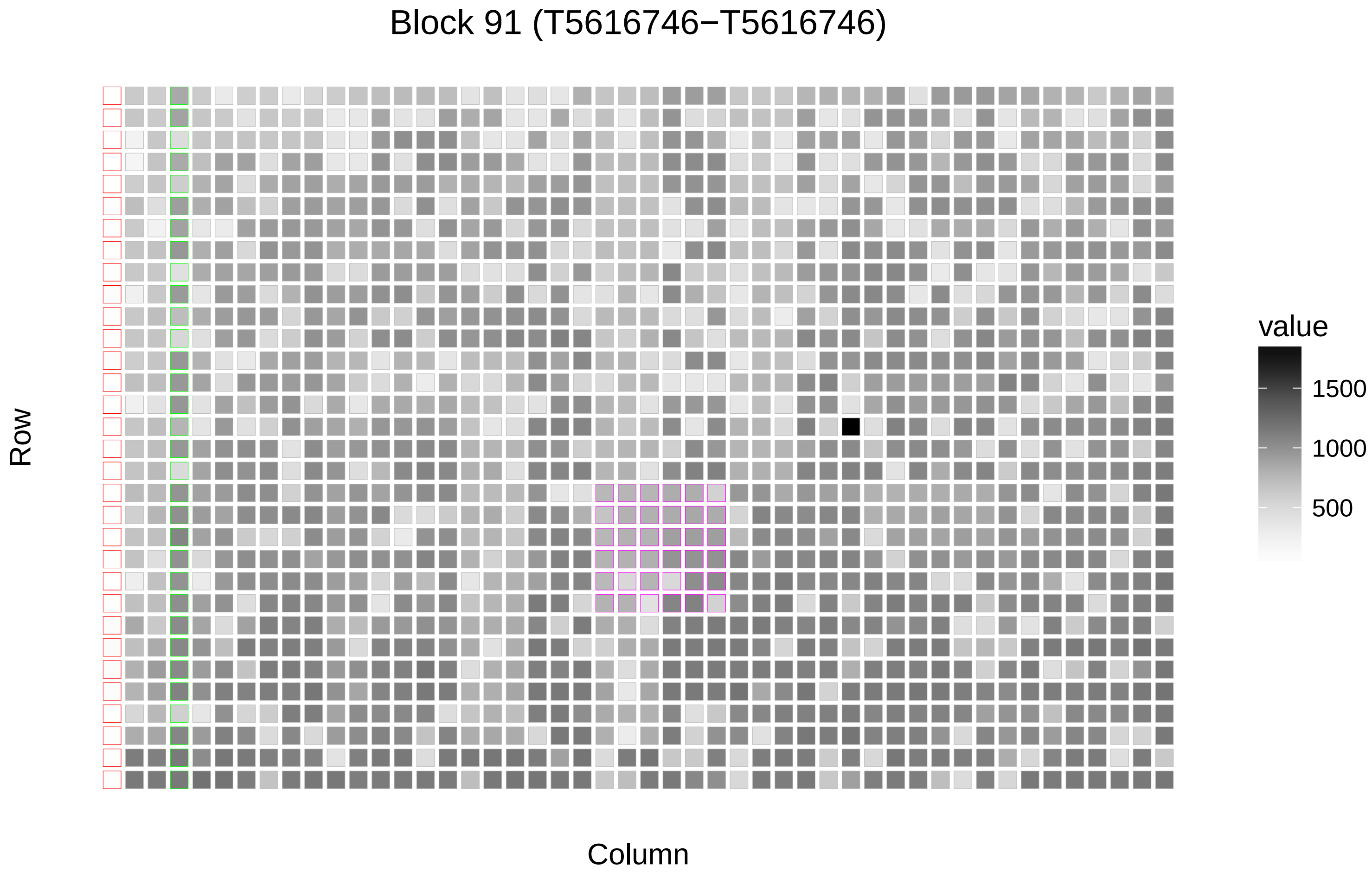

Block 91 (T5616746−T5616746)
Row
Column
value
1500
1000
500

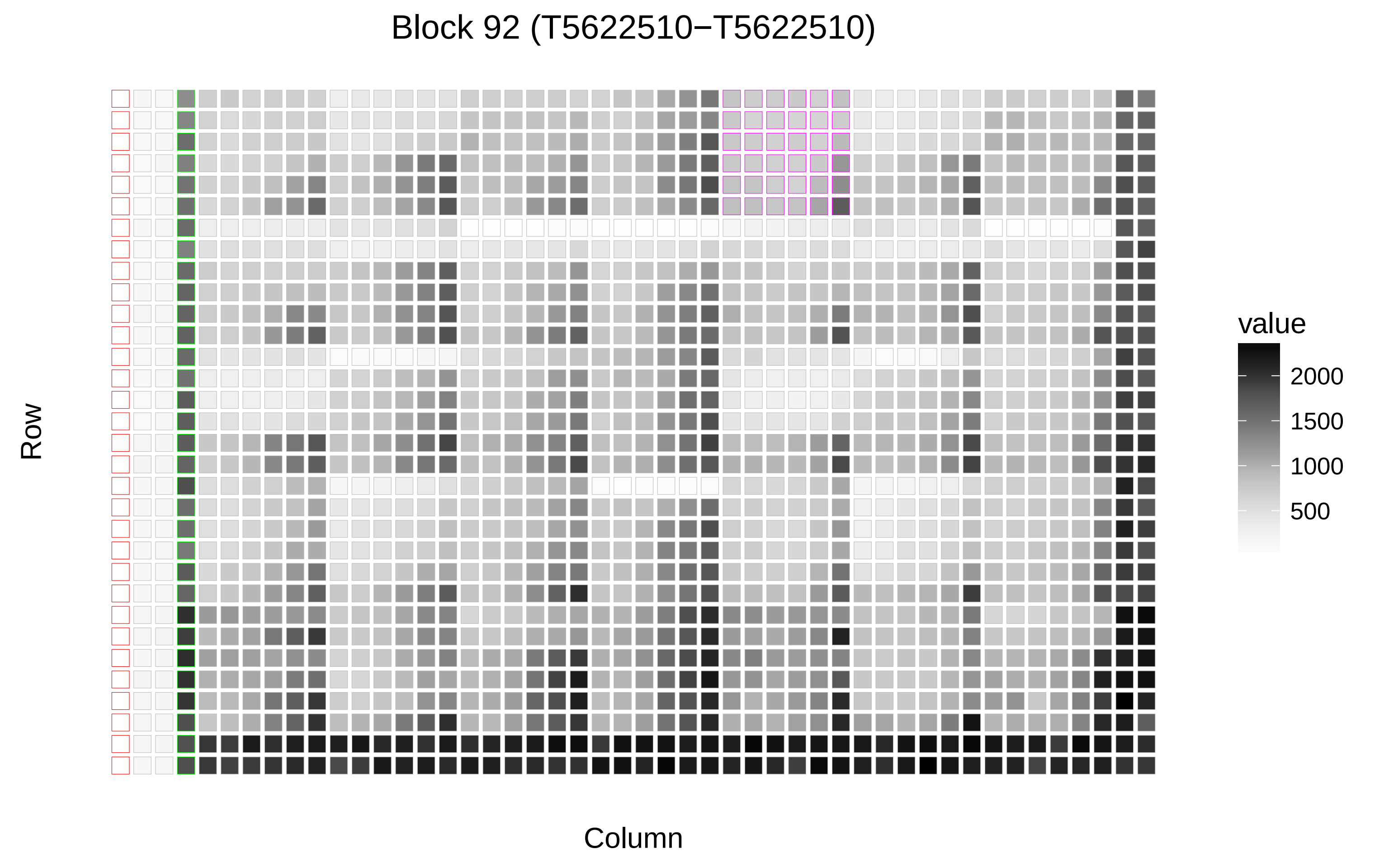
Block 92 (T5622510−T5622510)
Row
Column
value
2000
1500
1000
500

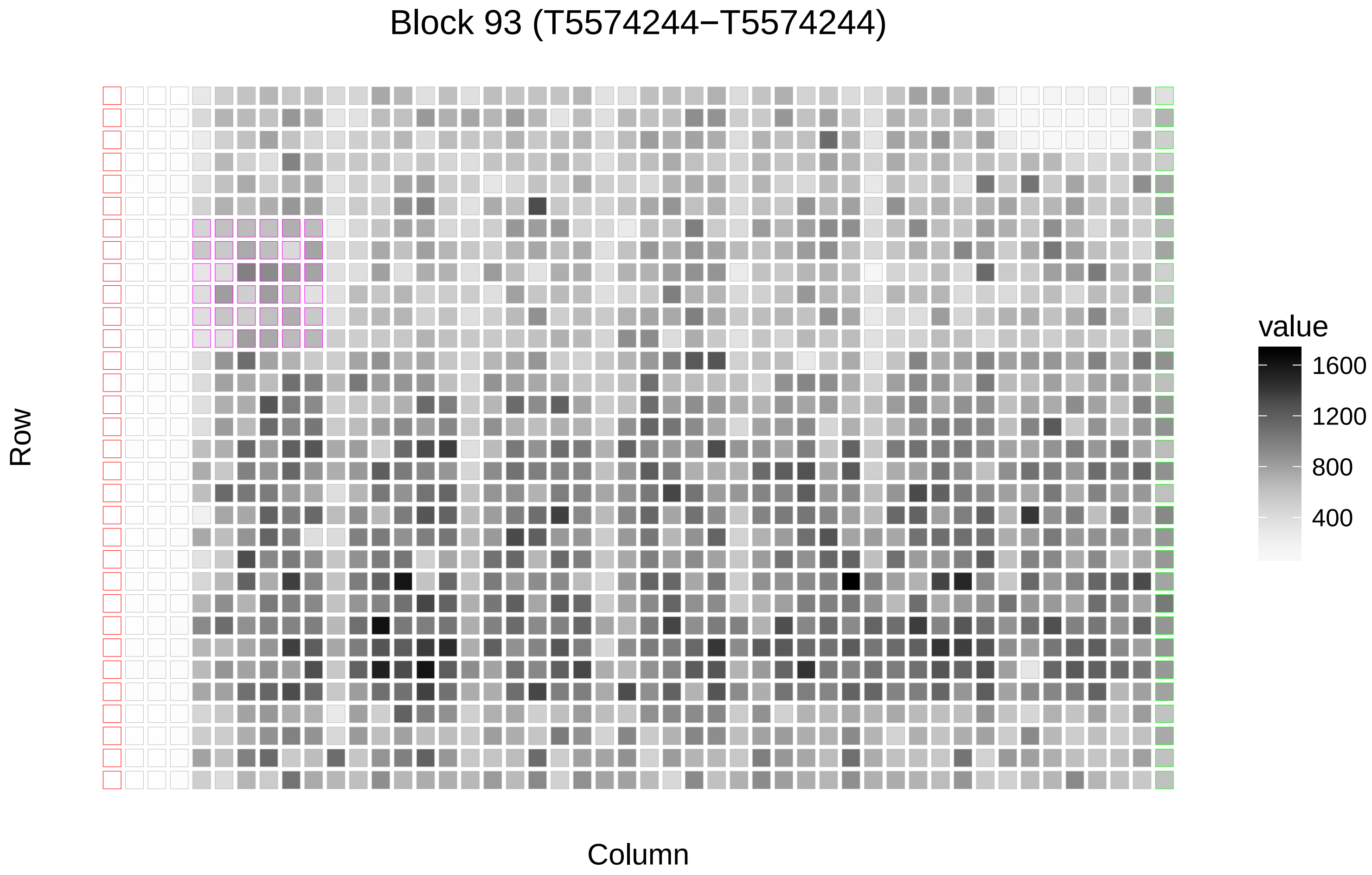
Block 93 (T5574244−T5574244)
Row
Column
value
1600
1200
800
400

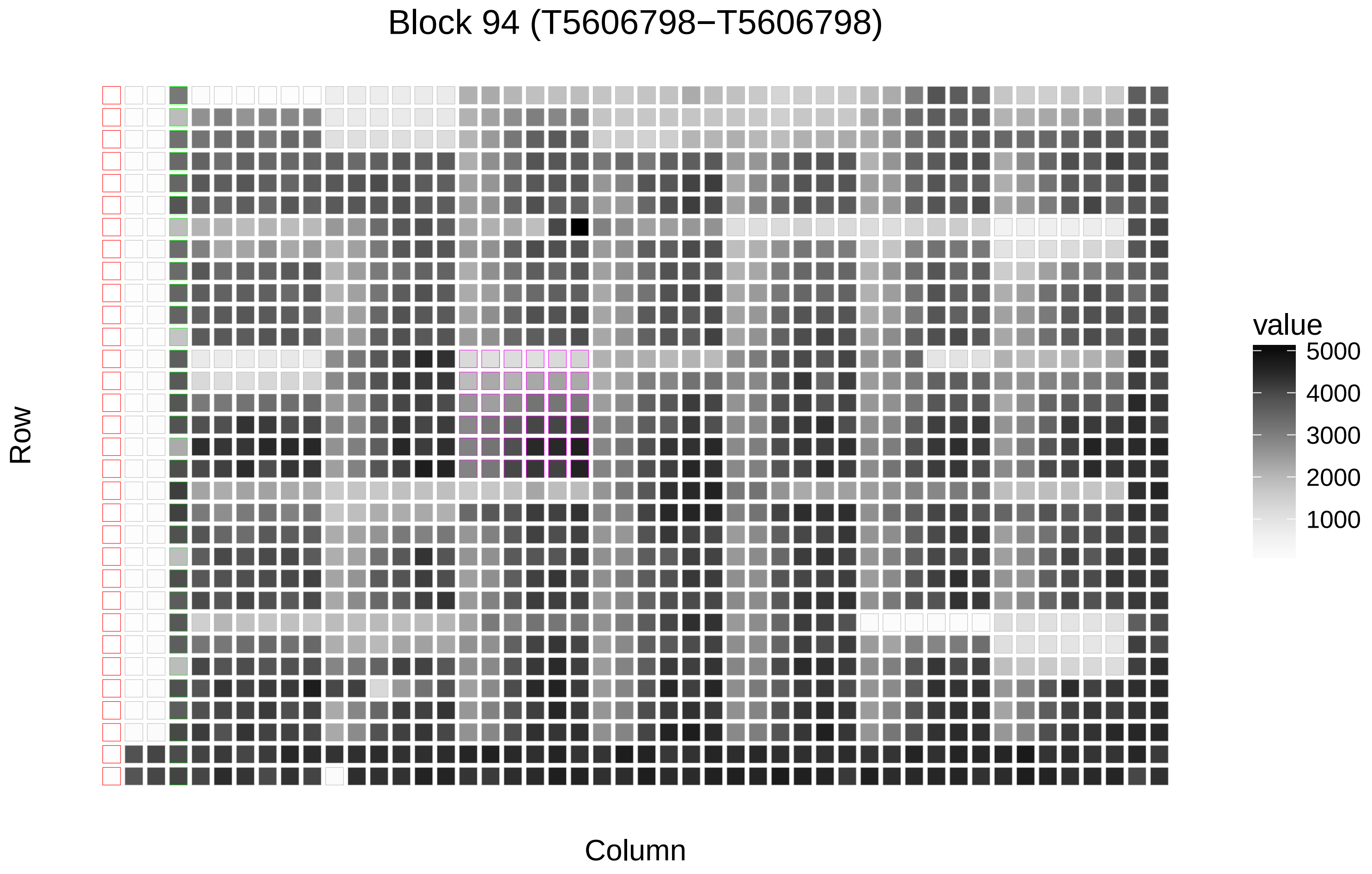
Block 94 (T5606798−T5606798)
Row
Column
value
5000
4000
3000
2000
1000

Block 95 (M0300026−T5470338)

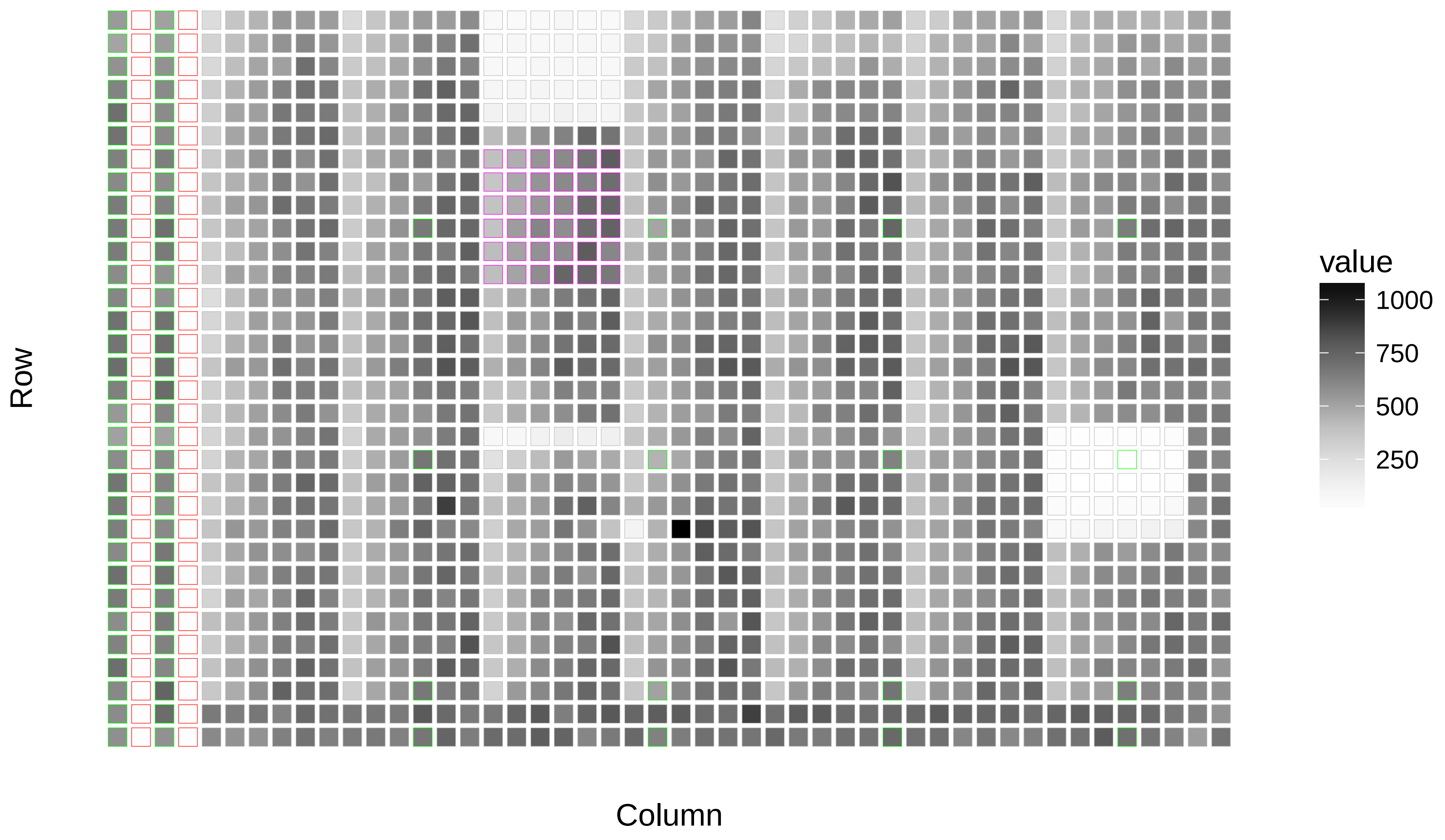

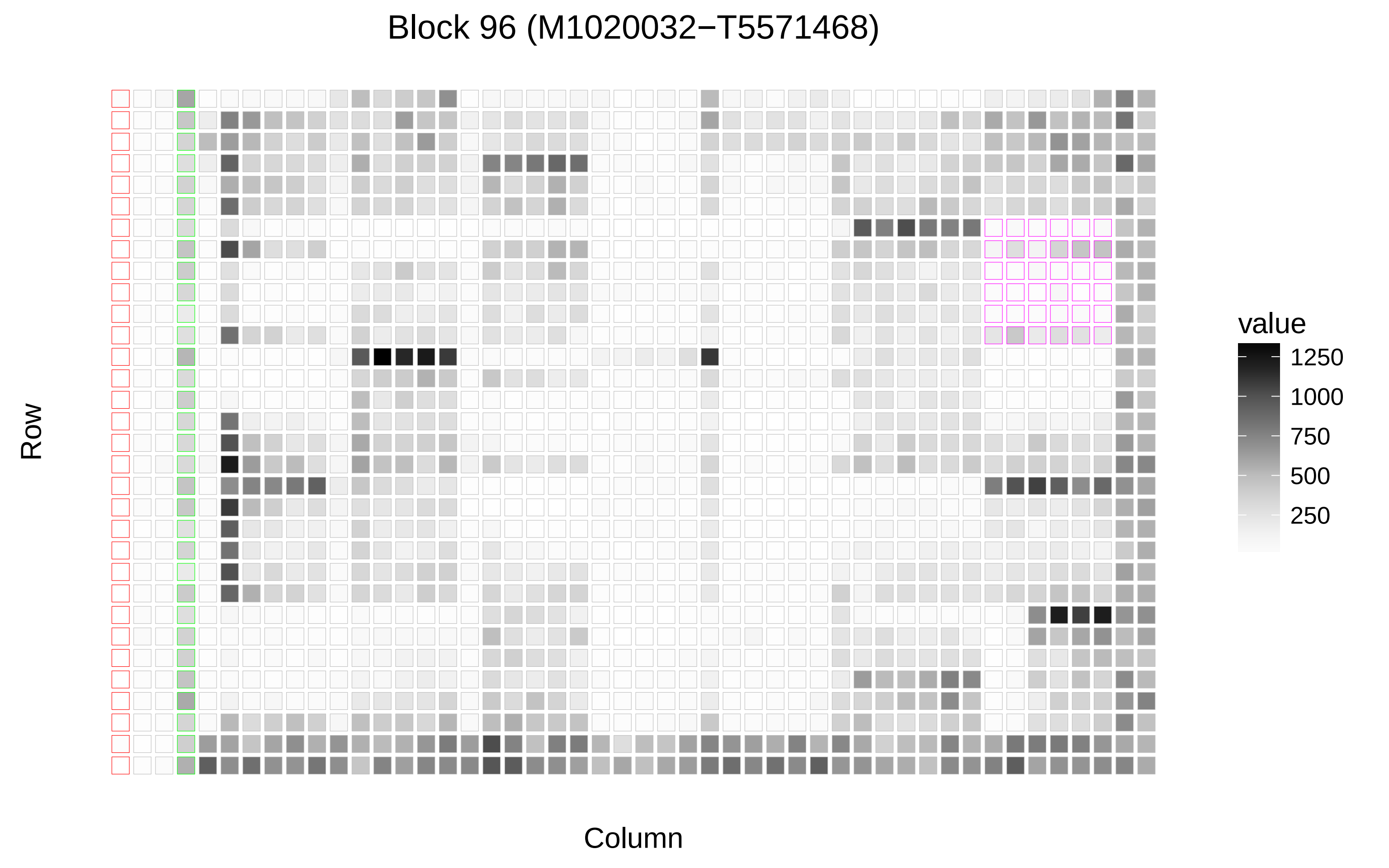

Block 96 (M1020032−T5571468)
Row
Column
value
1250
1000
750
500
250

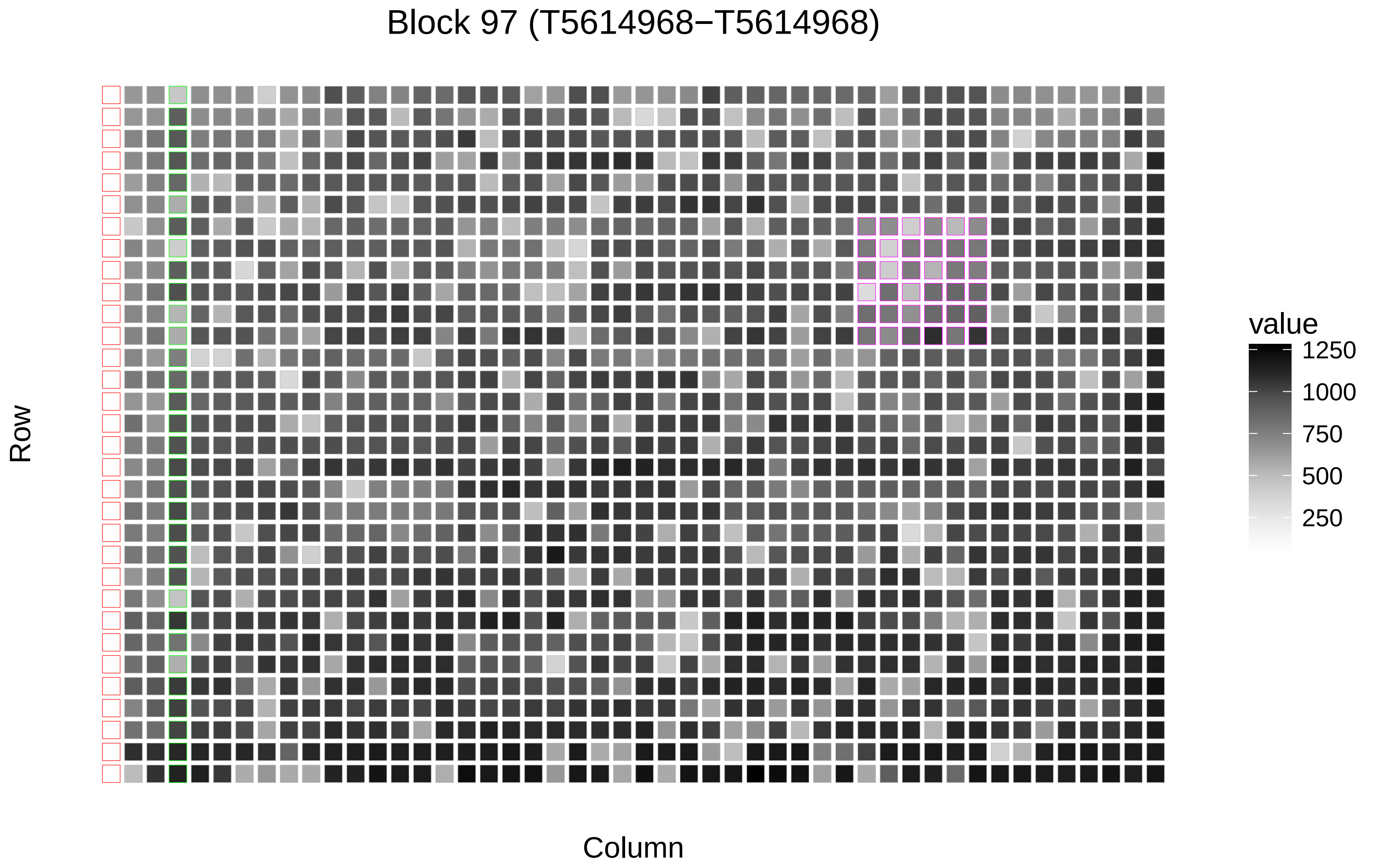
Block 97 (T5614968−T5614968)
Row
Column
value
1250
1000
750
500
250

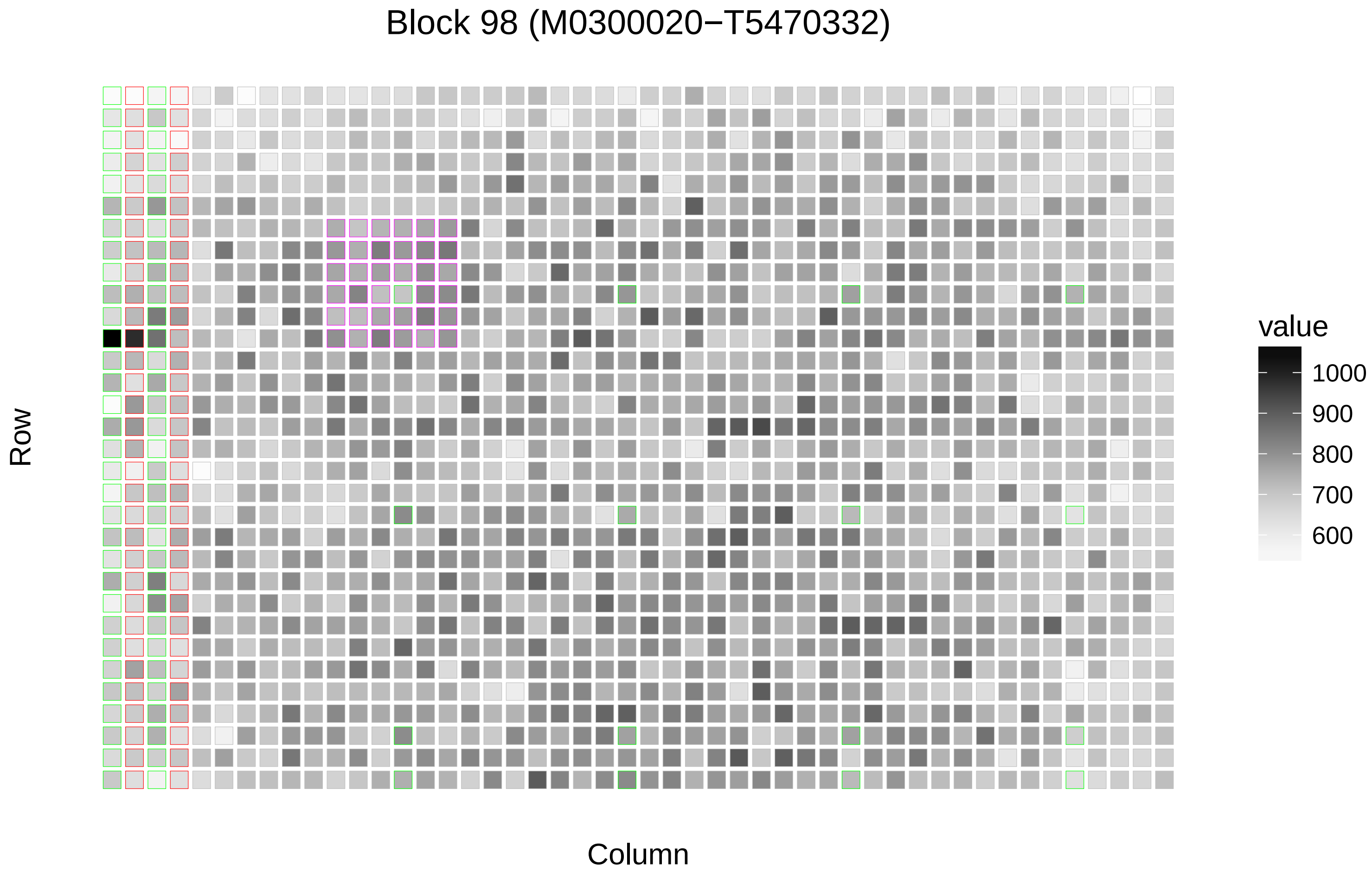
Block 98 (M0300020−T5470332)
Row
Column
value
1000
900
800
700
600

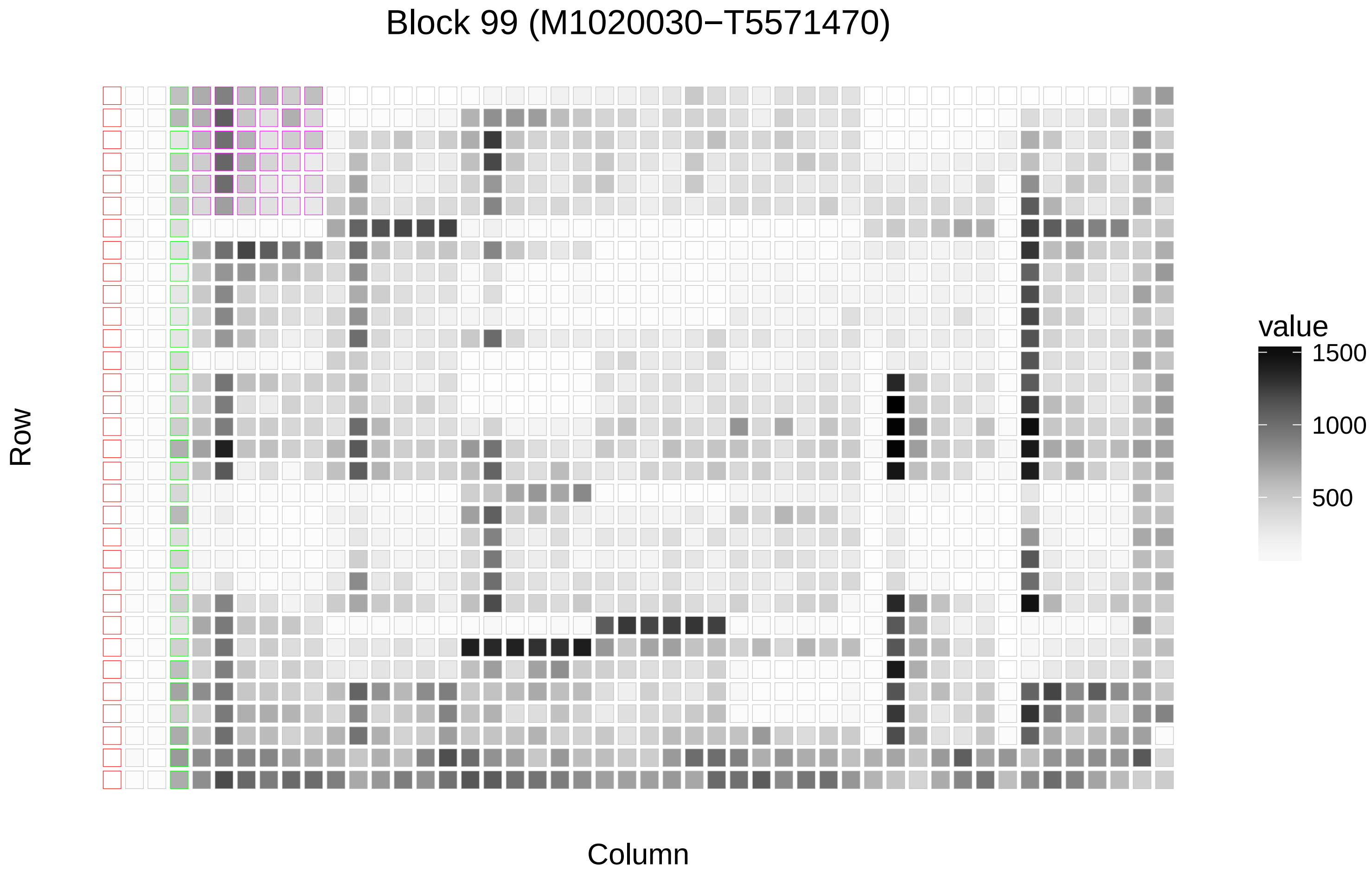
Block 99 (M1020030−T5571470)
Row
Column
value
1500
1000
500

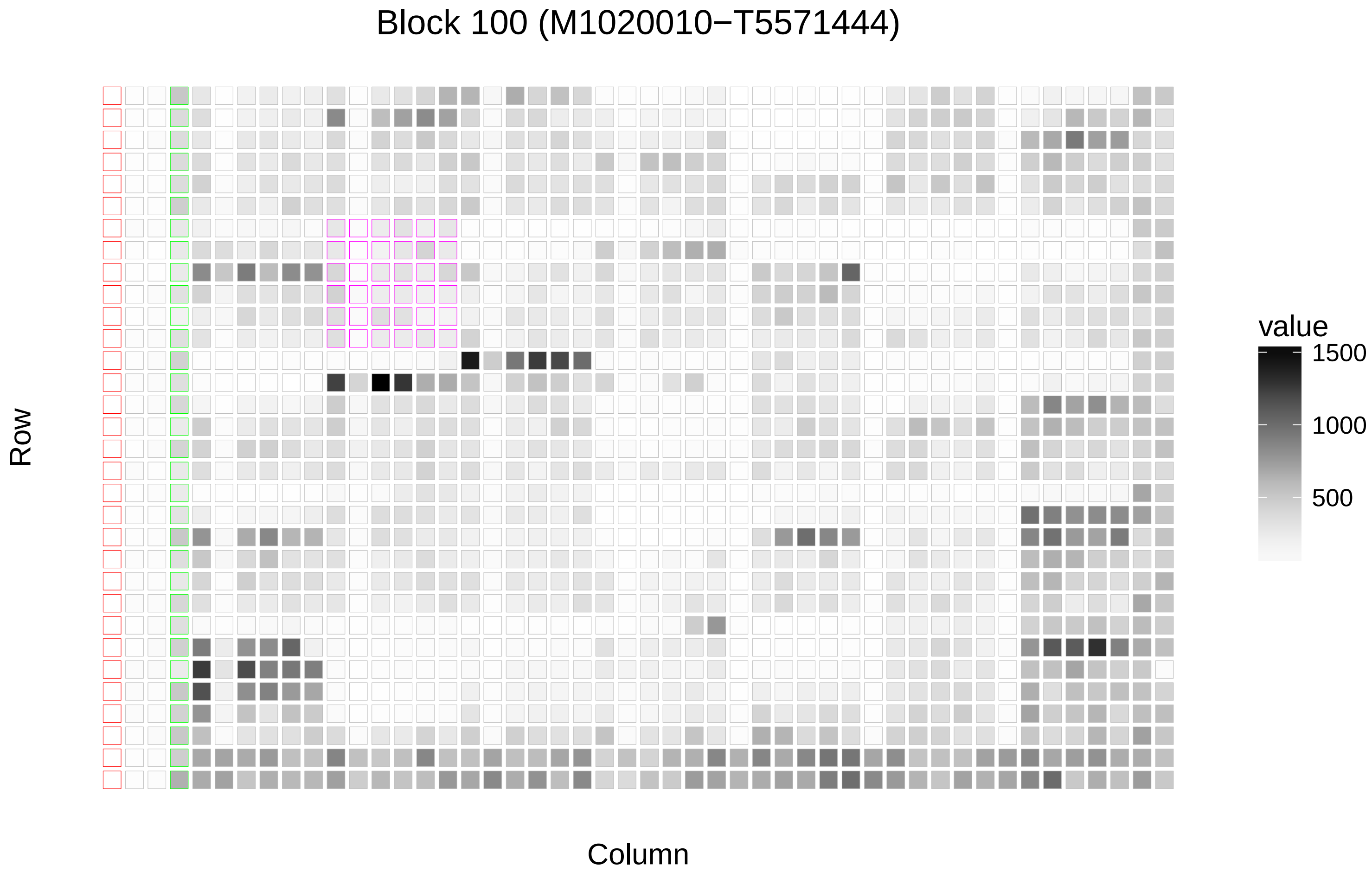
Block 100 (M1020010−T5571444)
Row
Column
value
1500
1000
500

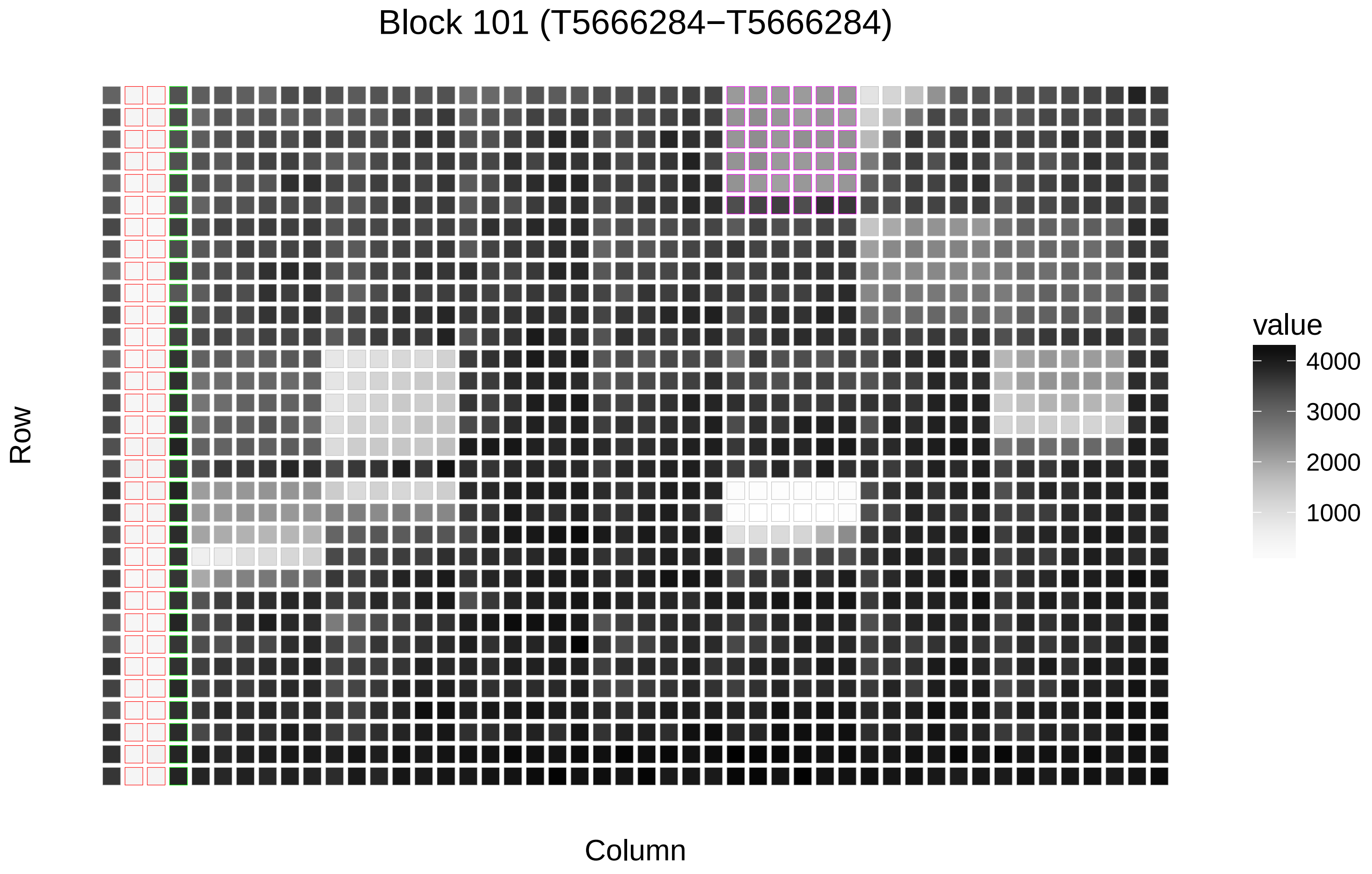
Block 101 (T5666284−T5666284)
Row
Column
value
4000
3000
2000
1000

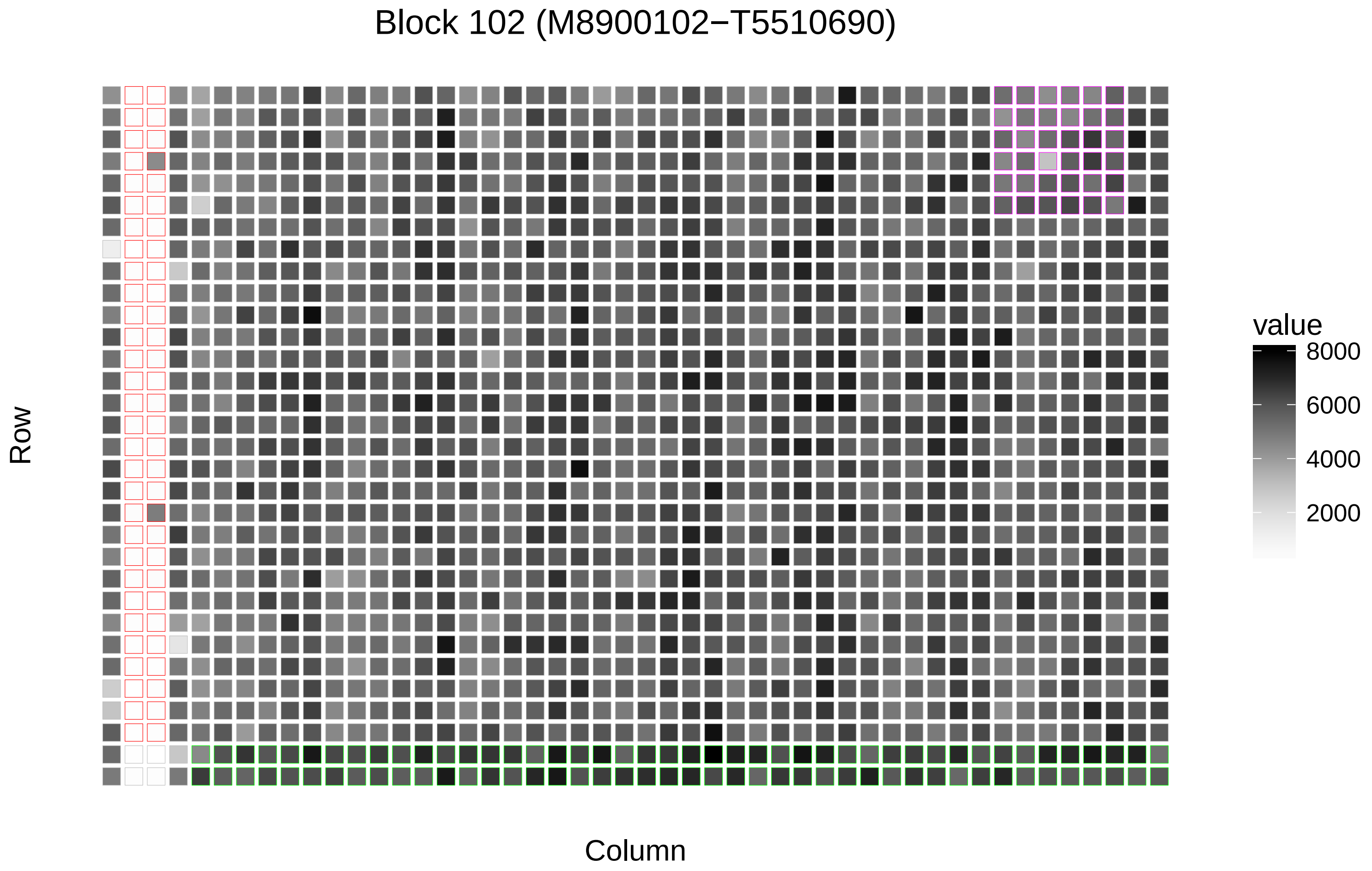
Block 102 (M8900102−T5510690)
Row
Column
value
8000
6000
4000
2000

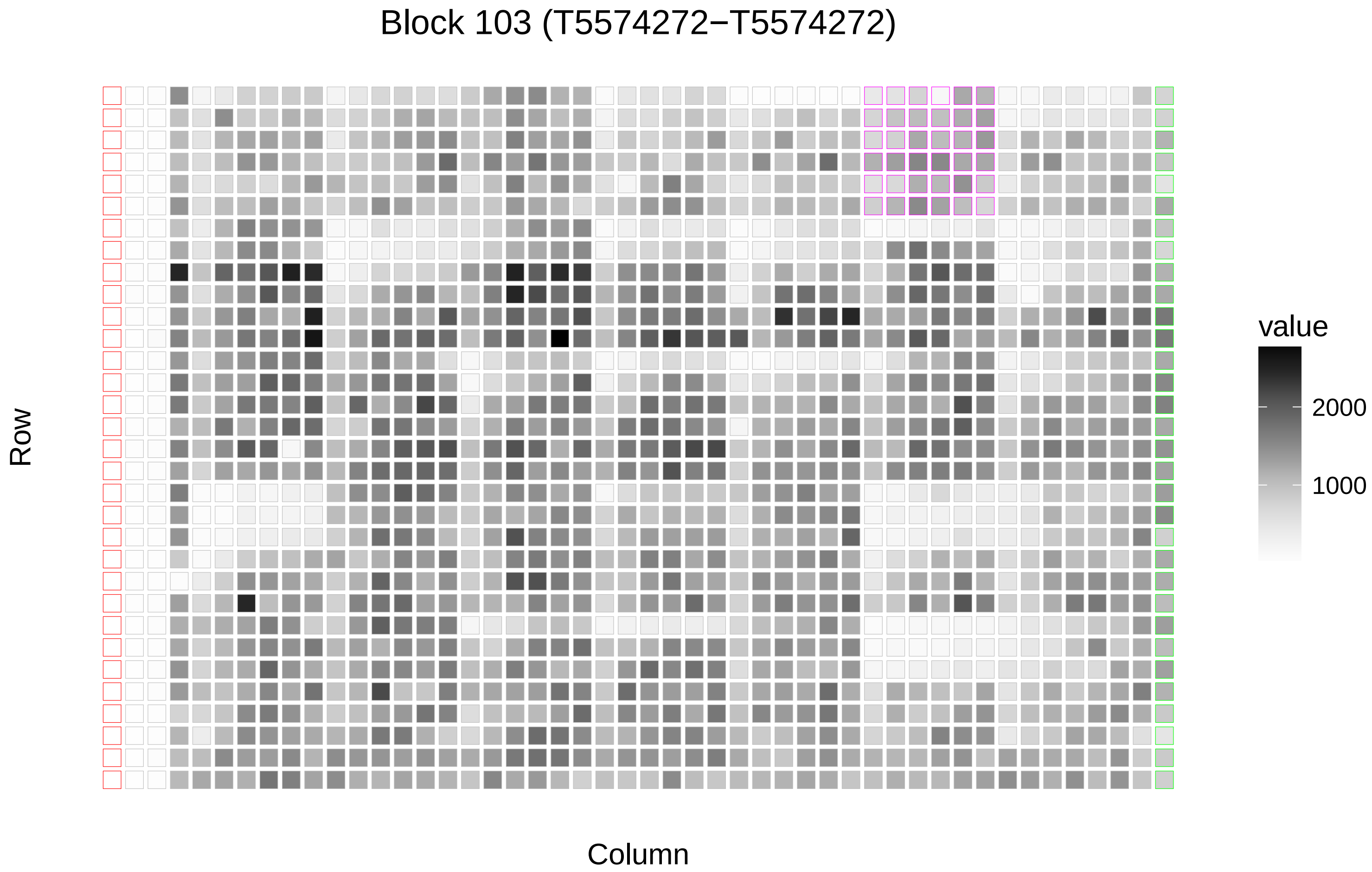
Block 103 (T5574272−T5574272)
Row
Column
value
2000
1000

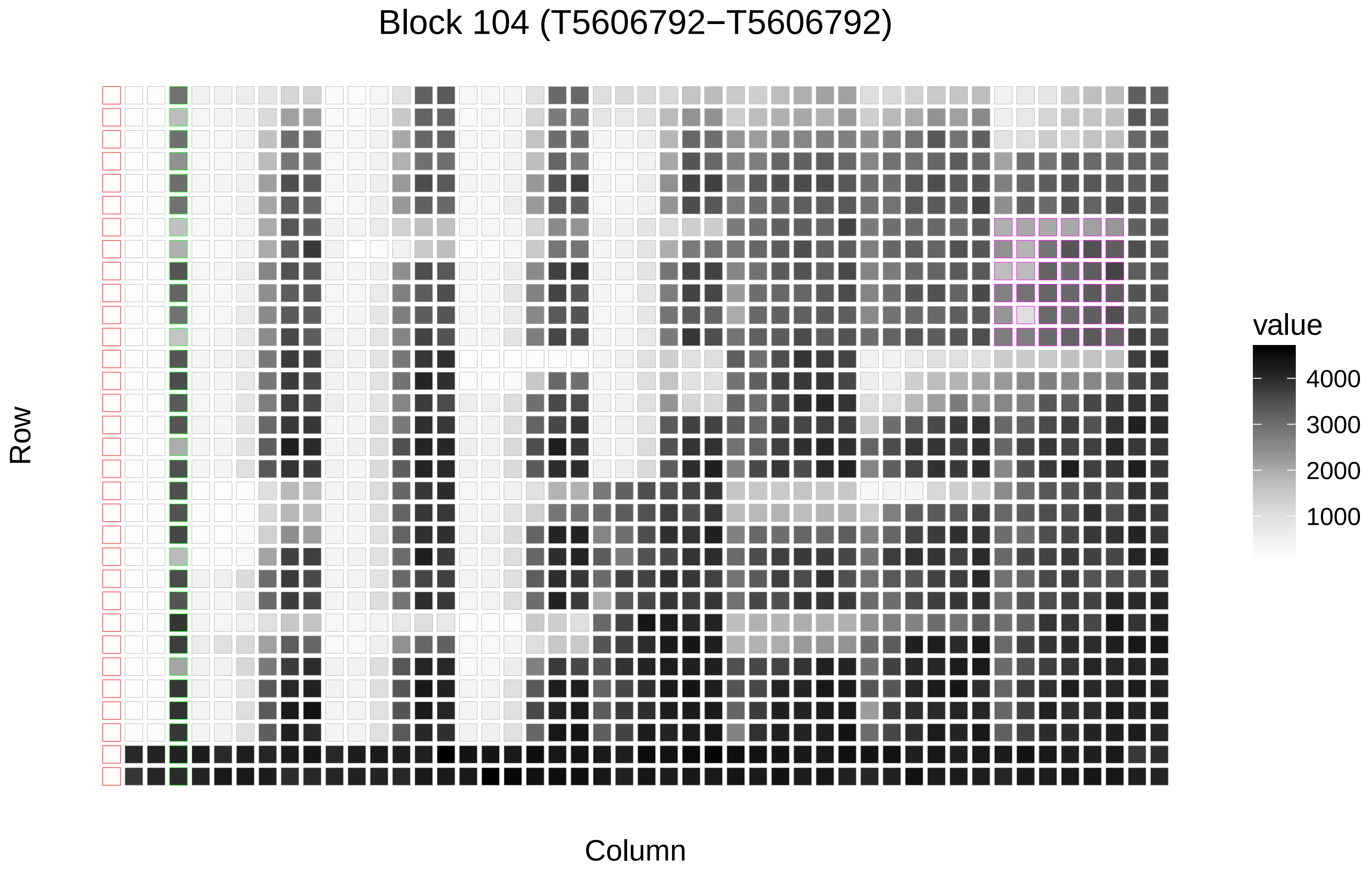
Block 104 (T5606792−T5606792)
Row
Column
value
4000
3000
2000
1000

Block 105 (T5574254−T5574254)

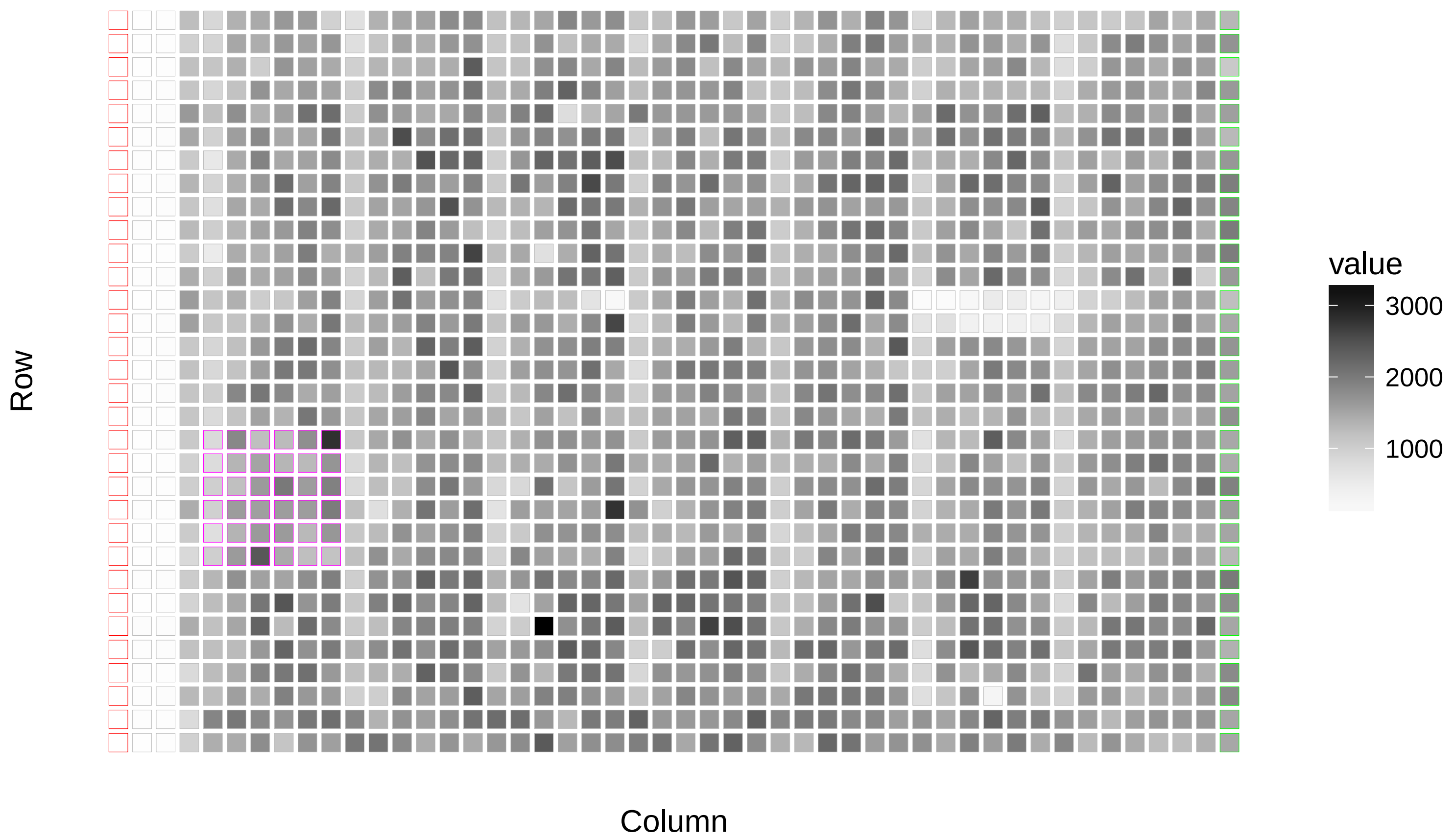

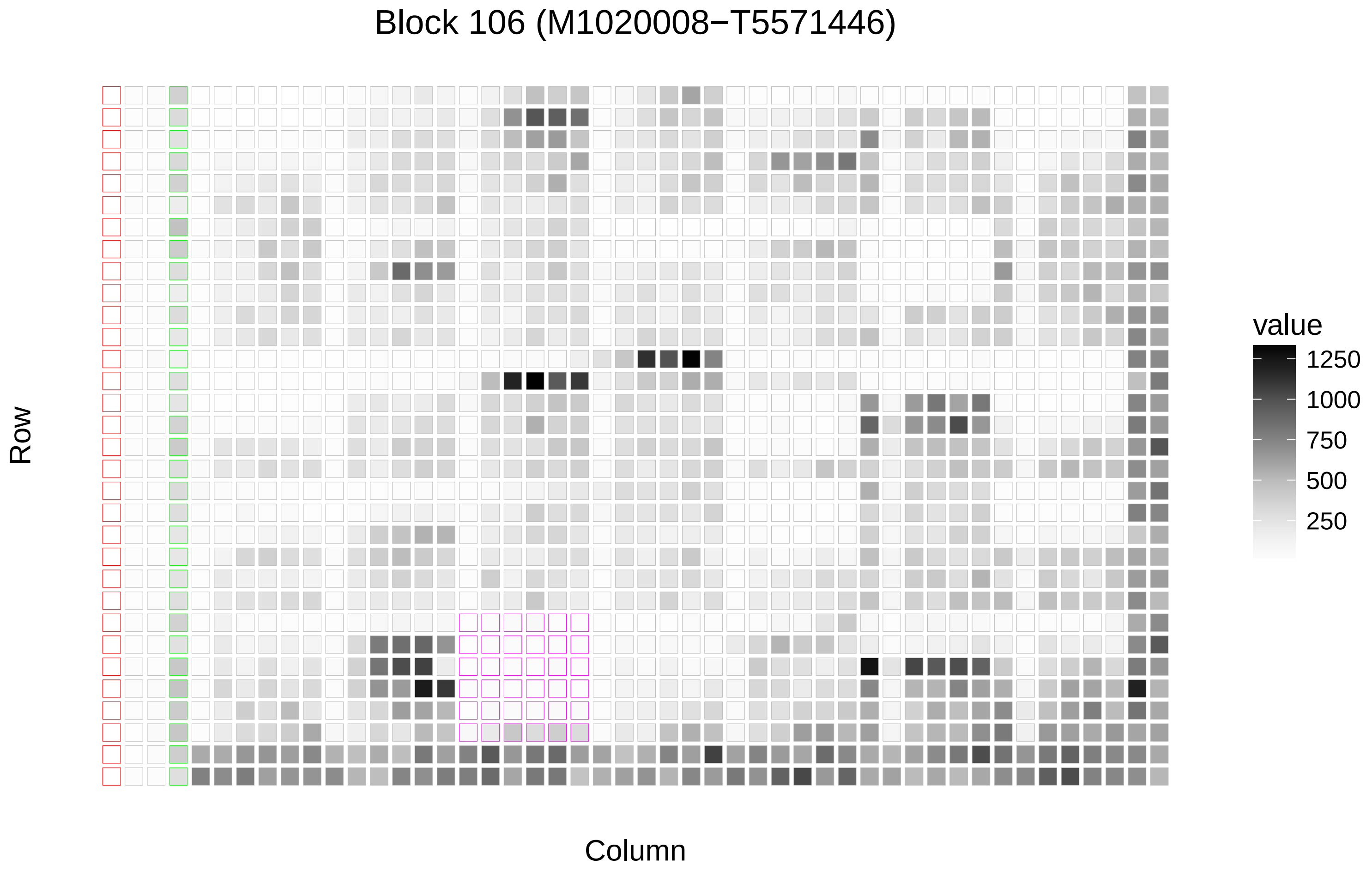

Block 106 (M1020008−T5571446)
Row
Column
value
1250
1000
750
500
250

Block 107 (T5622508−T5622508)

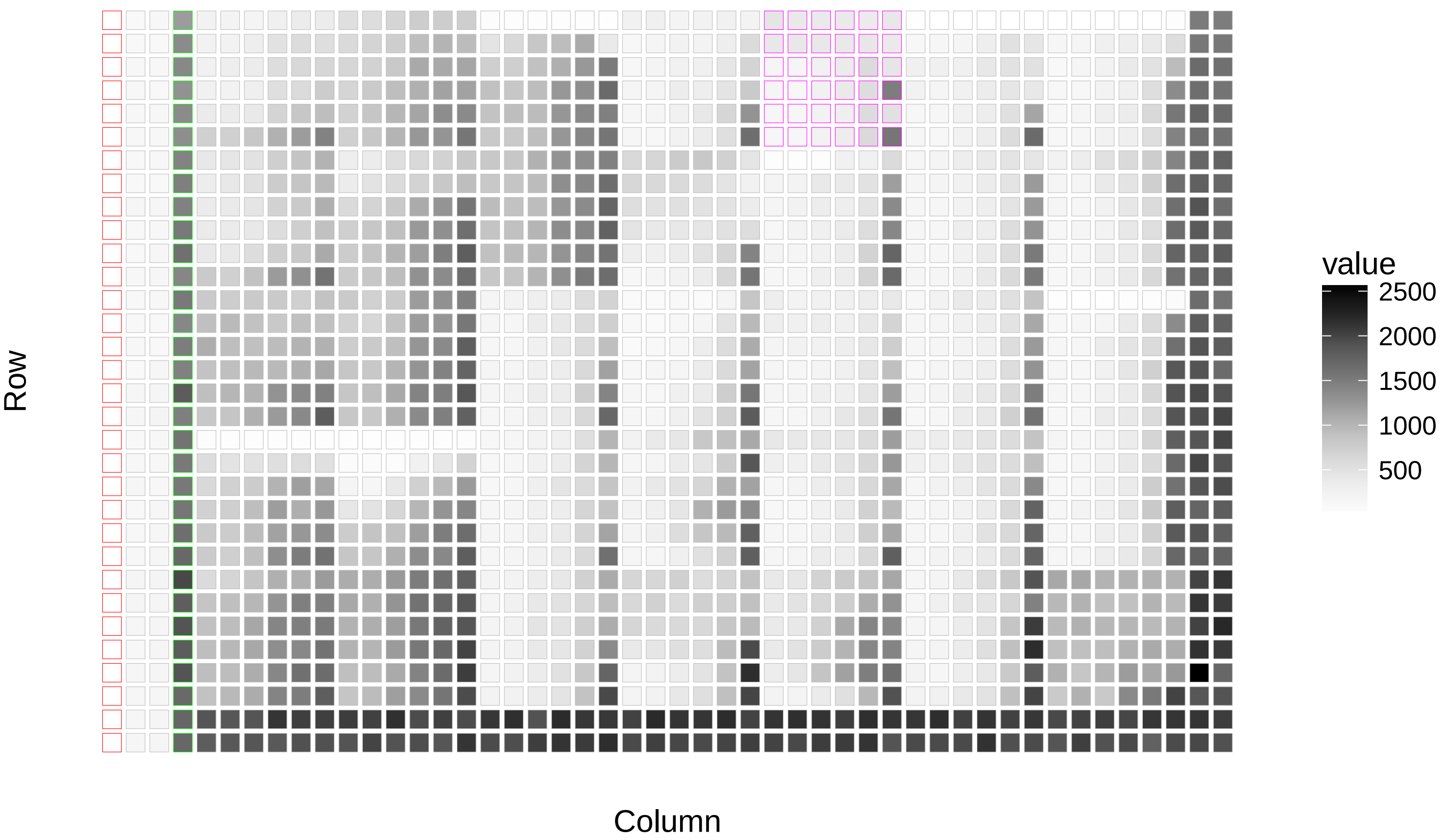

Block 108 (M1400012−T5436672)

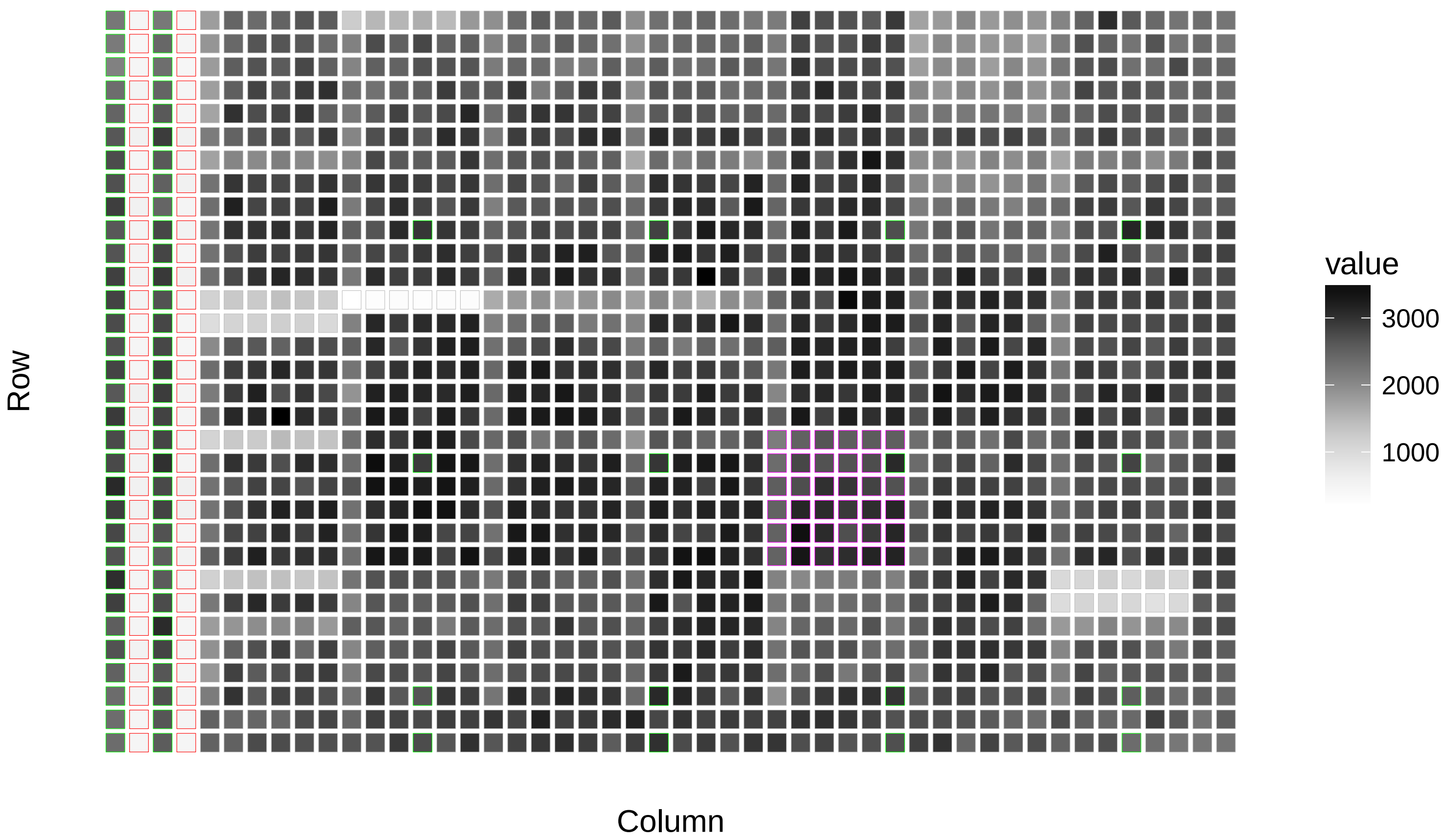

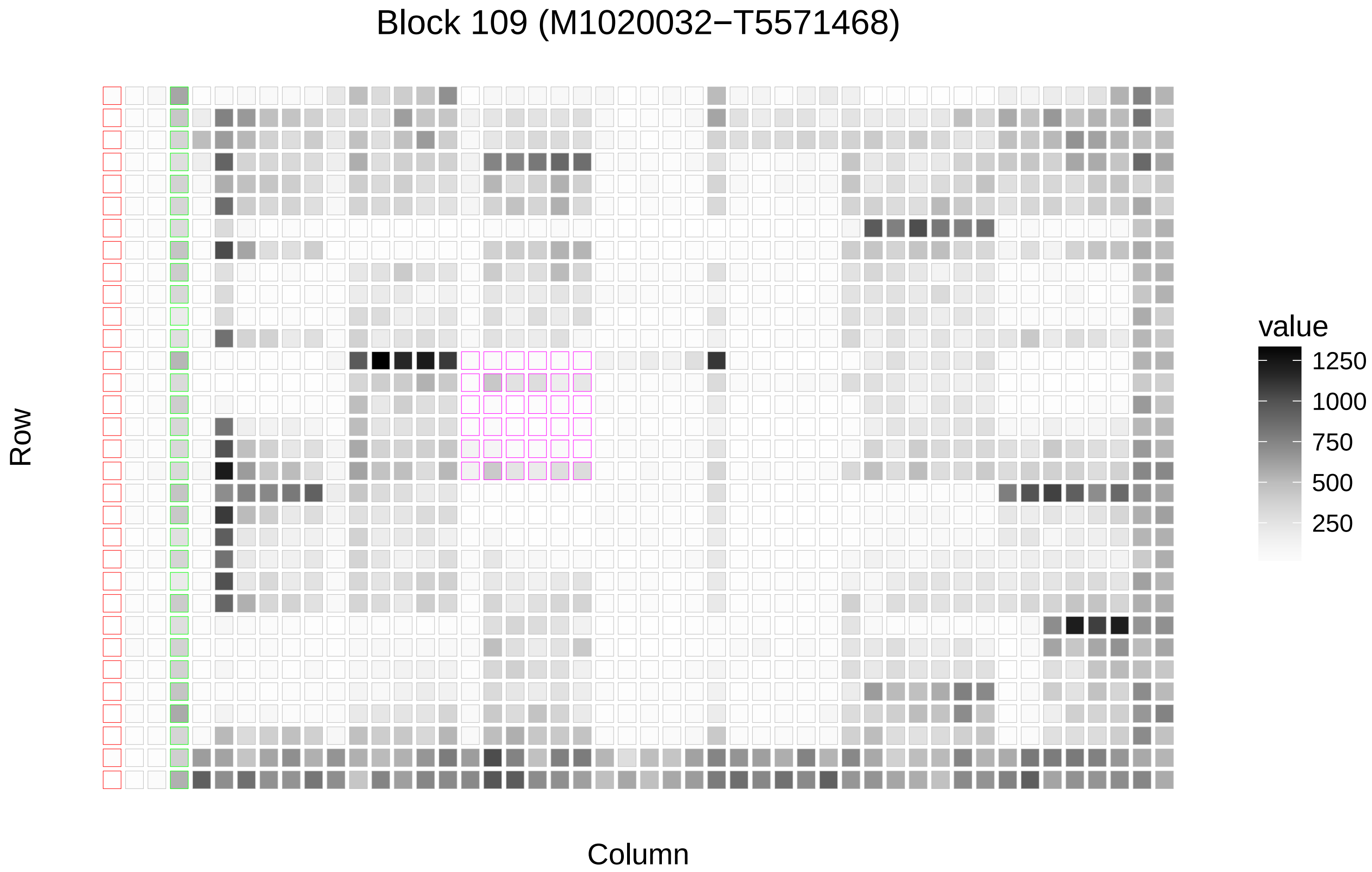

Block 109 (M1020032−T5571468)
Row
Column
value
1250
1000
750
500
250

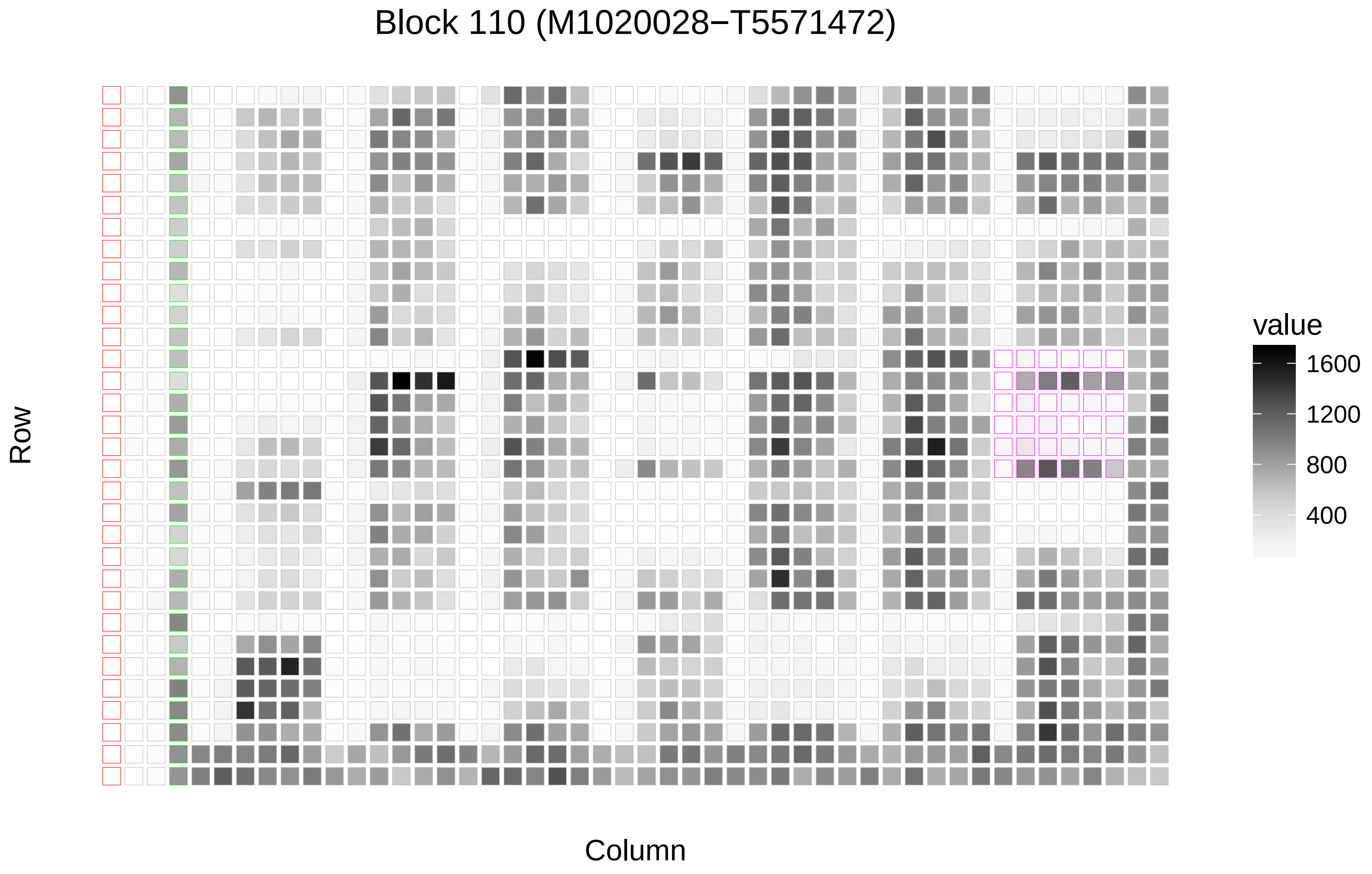
Block 110 (M1020028−T5571472)
Row
Column
value
1600
1200
800
400

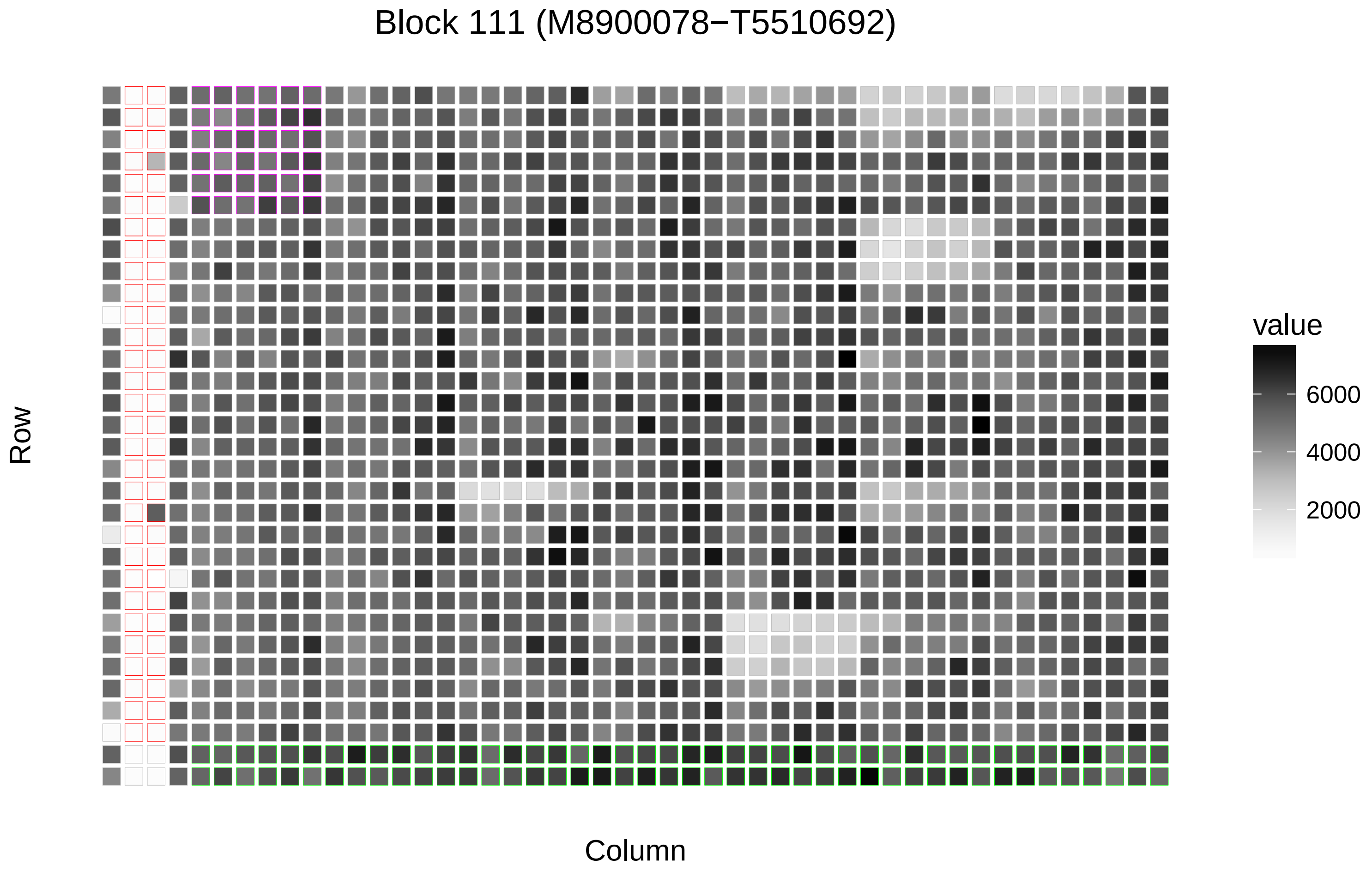

Block 111 (M8900078−T5510692)
Row
Column
value
6000
4000
2000

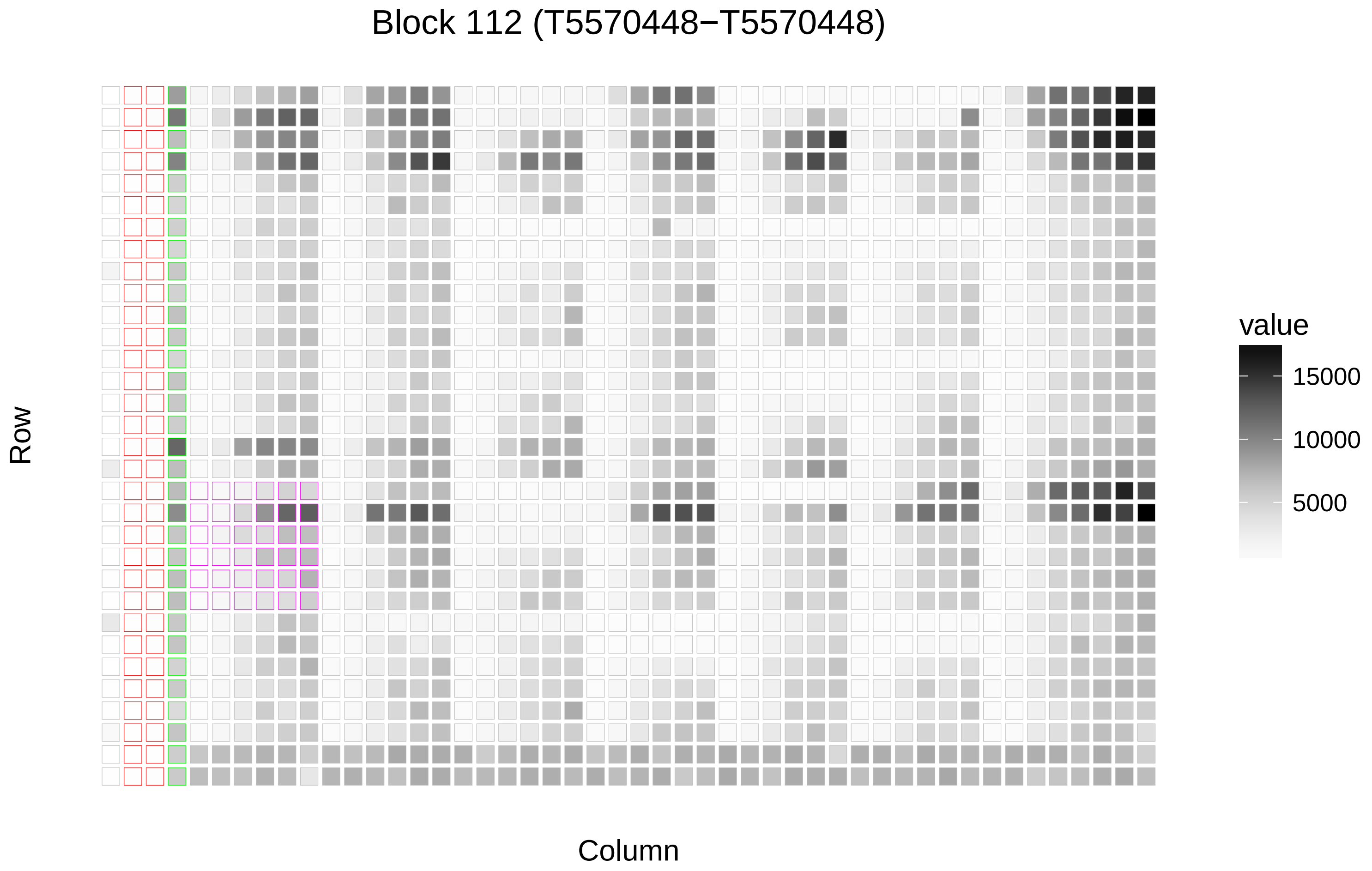
Block 112 (T5570448−T5570448)
Row
Column
value
15000
10000
5000

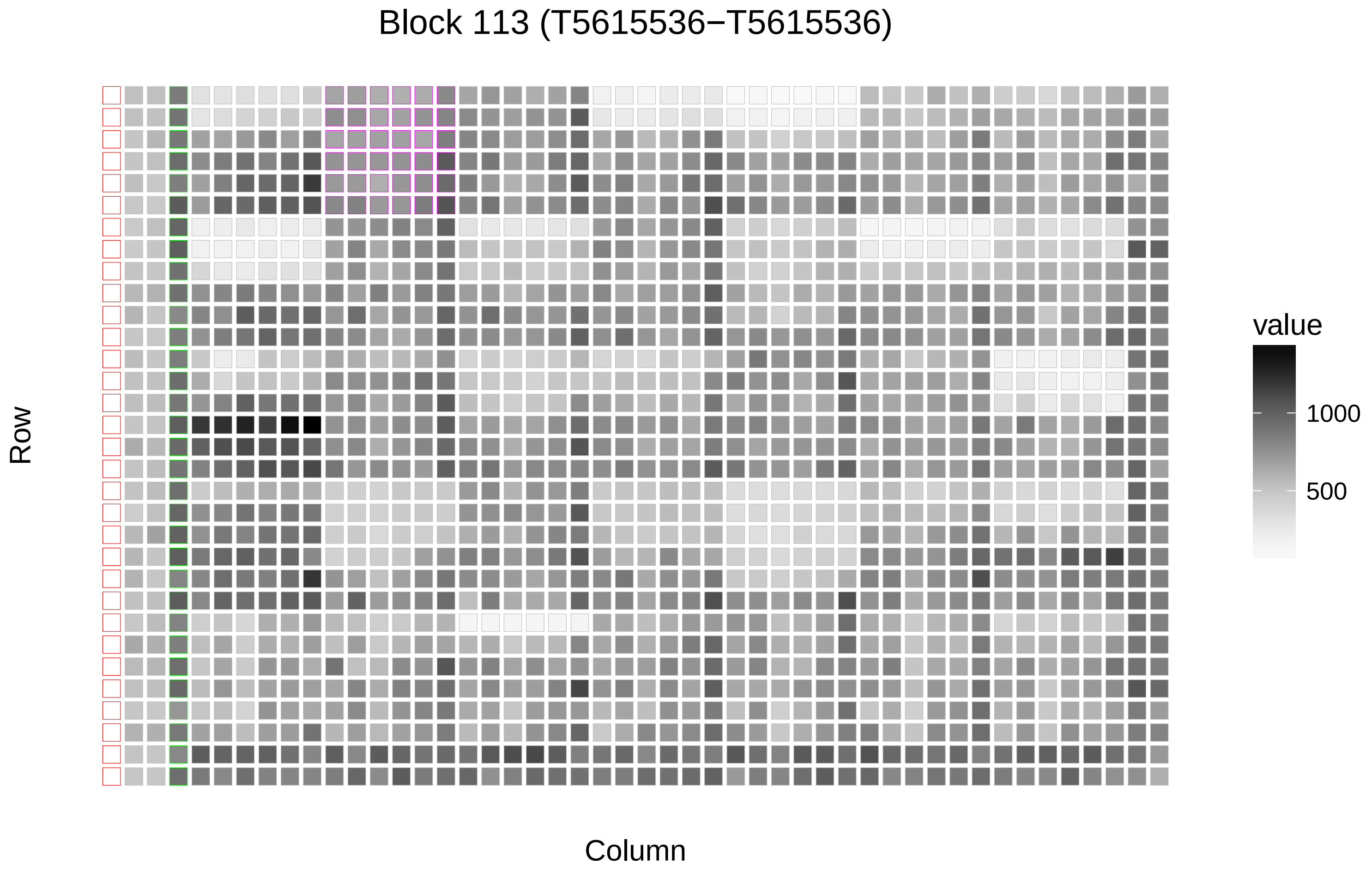
Block 113 (T5615536−T5615536)
Row
Column
value
1000
500

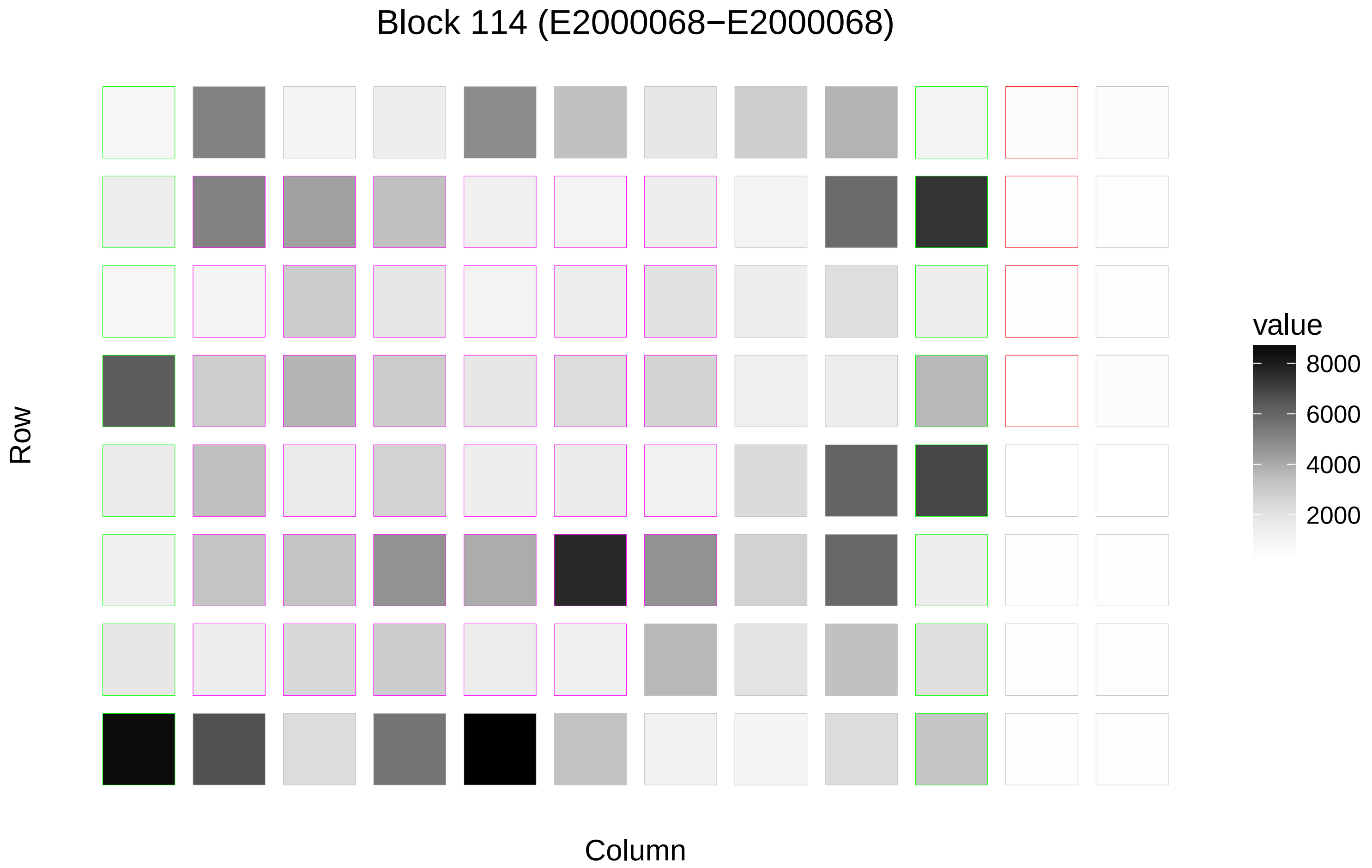

Block 114 (E2000068−E2000068)
Row
Column
value
8000
6000
4000
2000

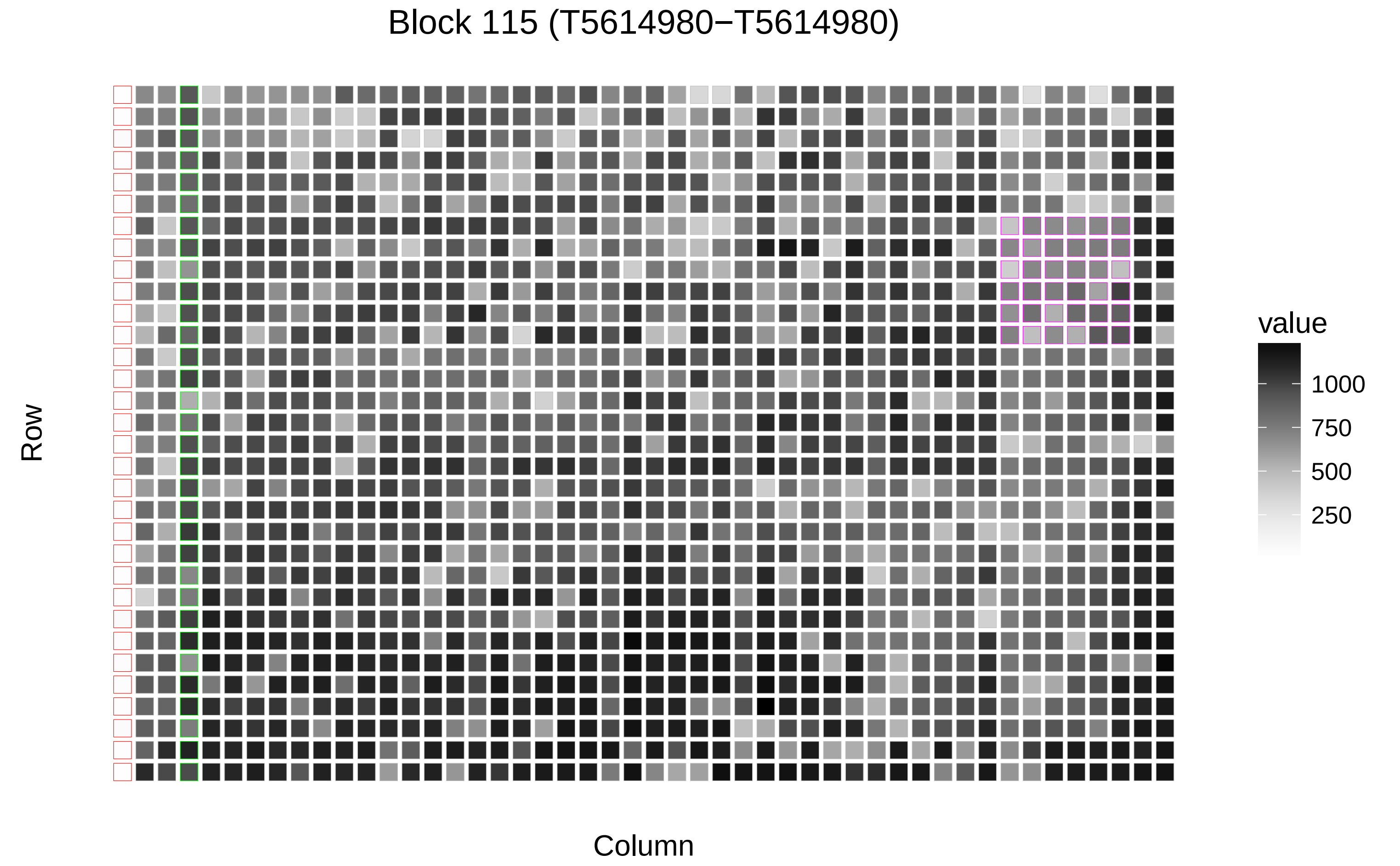
Block 115 (T5614980−T5614980)
value
1000
750
500
250
Row
Column

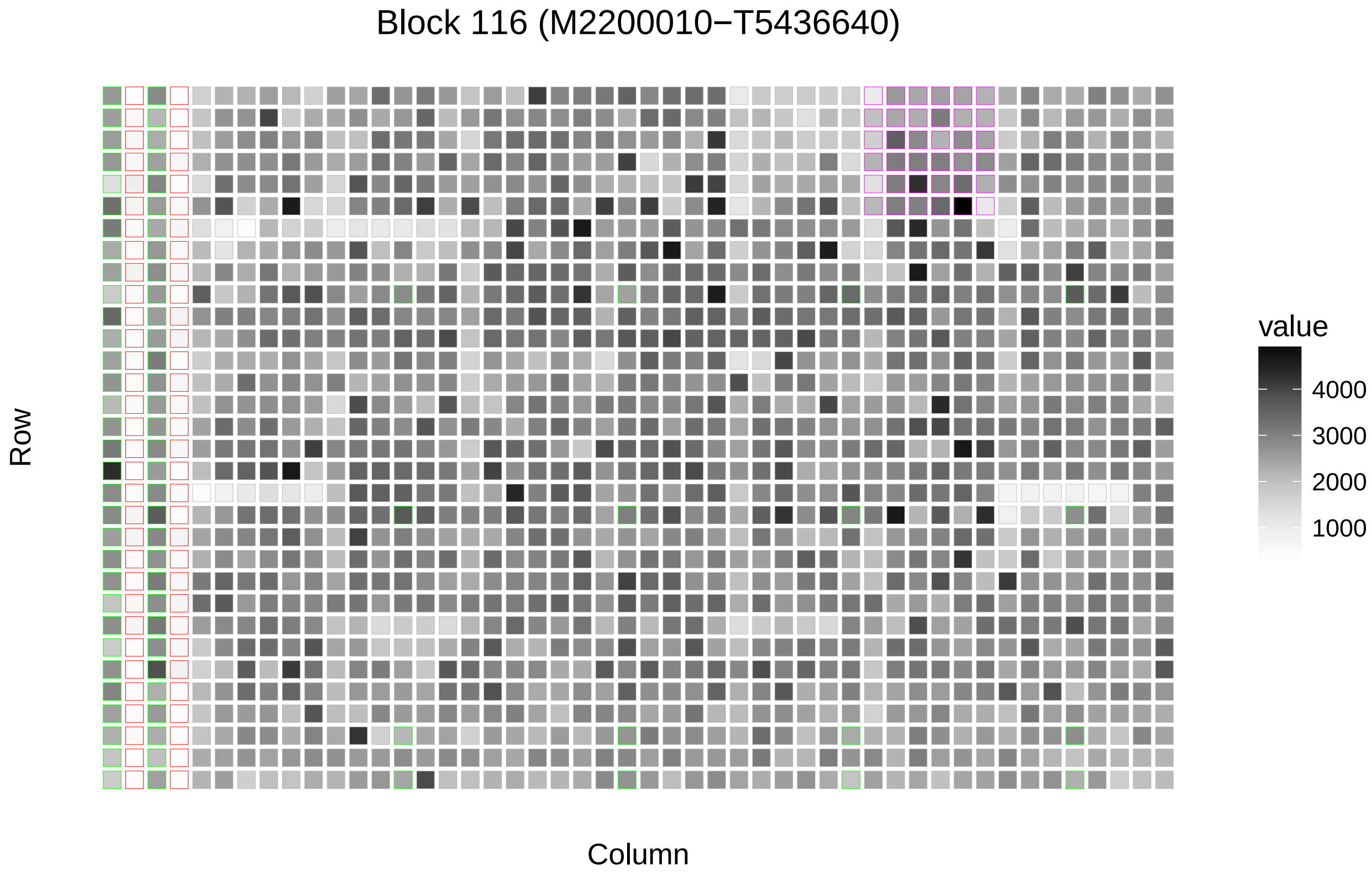

Block 116 (M2200010−T5436640)
Row
Column
value
4000
3000
2000
1000

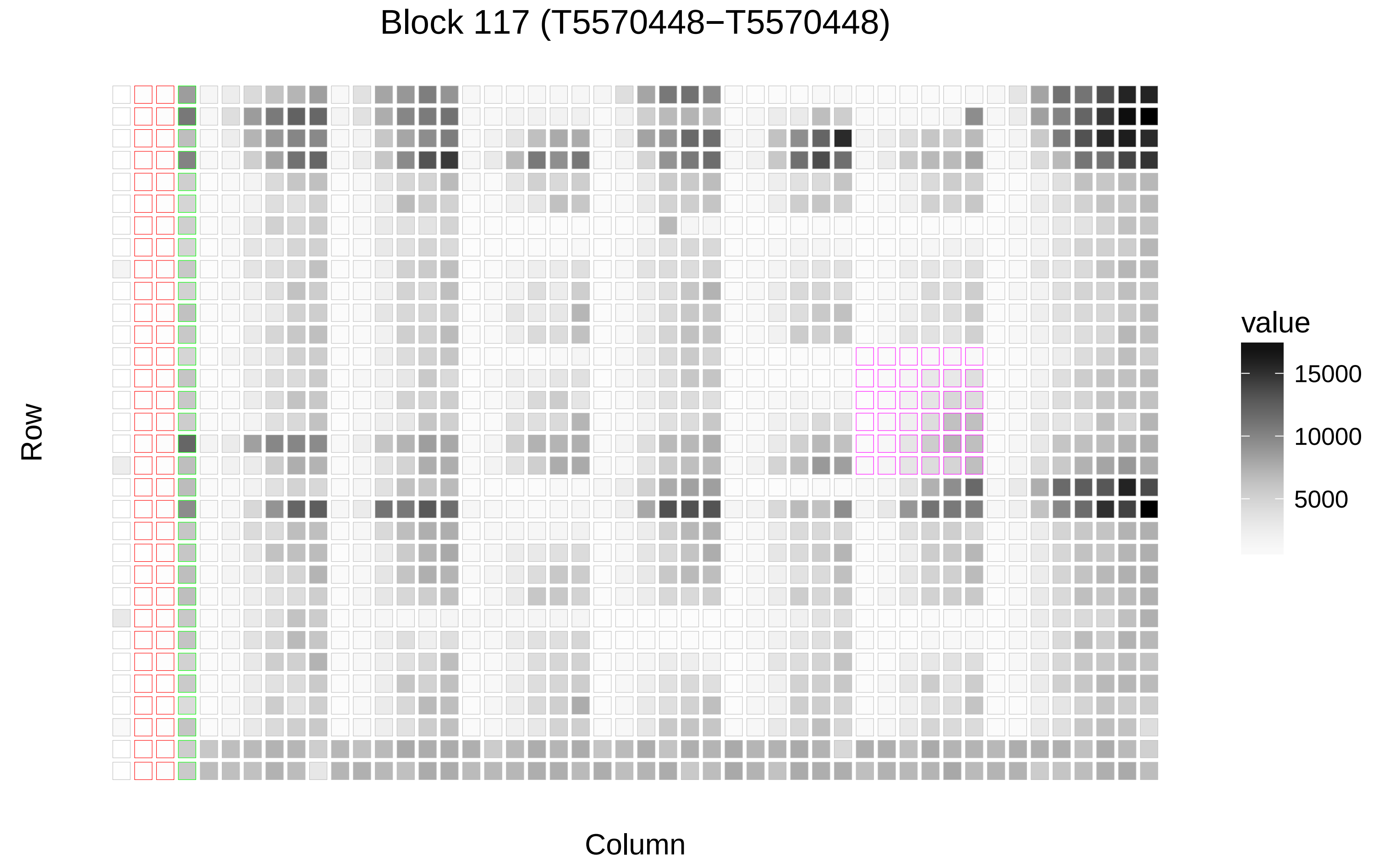
Block 117 (T5570448−T5570448)
Row
Column
value
15000
10000
5000

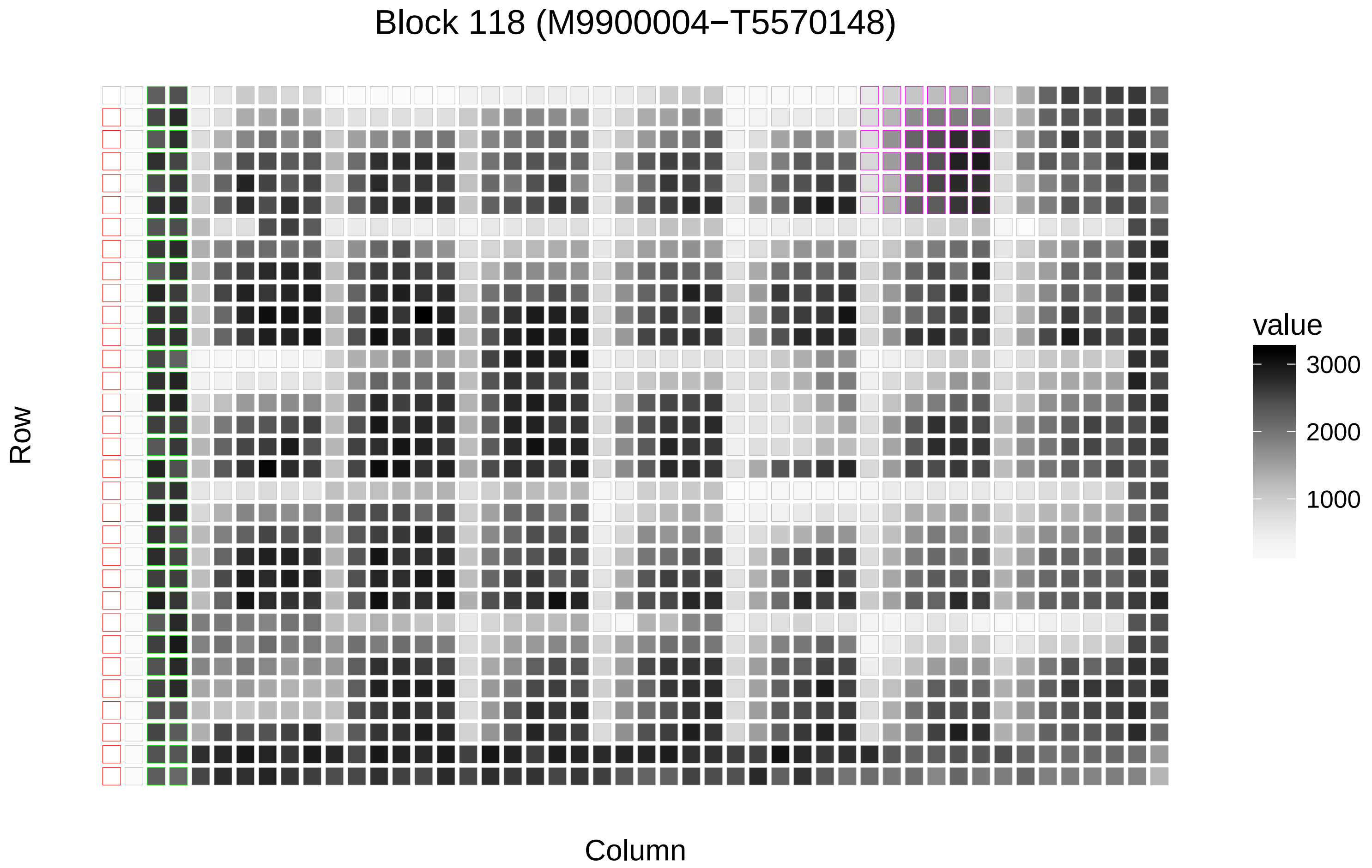
Block 118 (M9900004−T5570148)
Row
Column
value
3000
2000
1000

Block 119 (M1400026−T5436718)

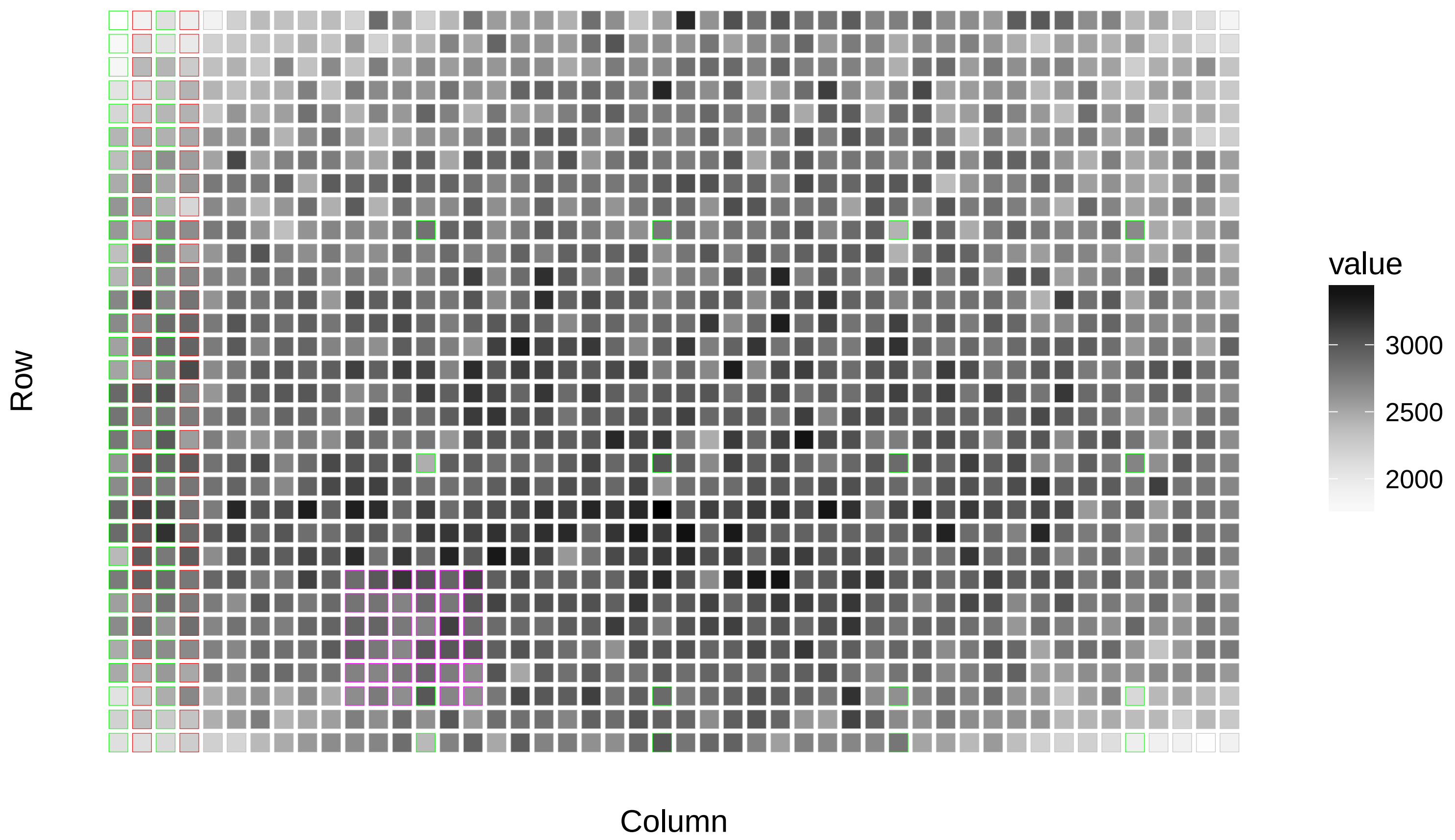

Block 120 (M9900048−T5574670)

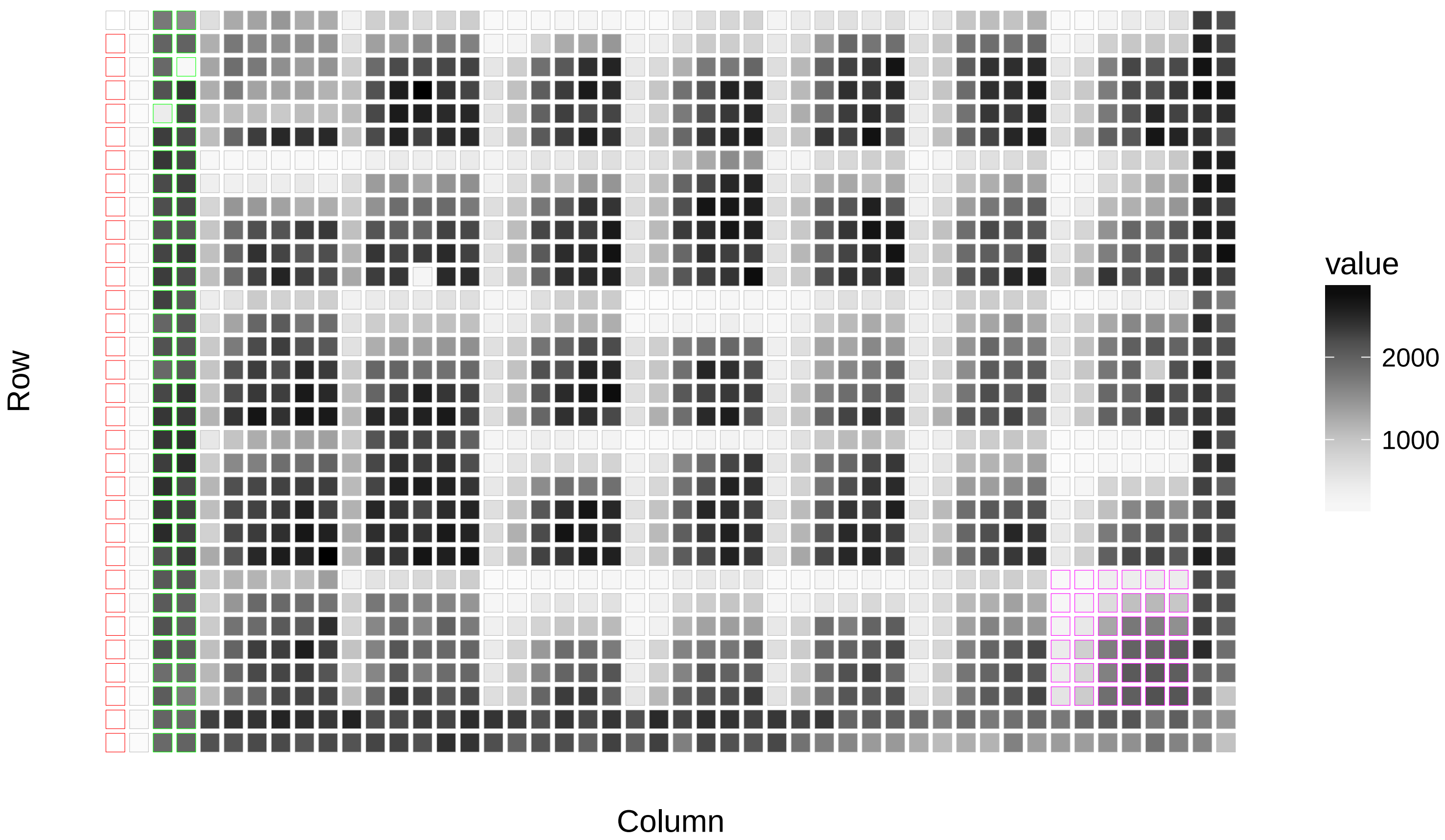

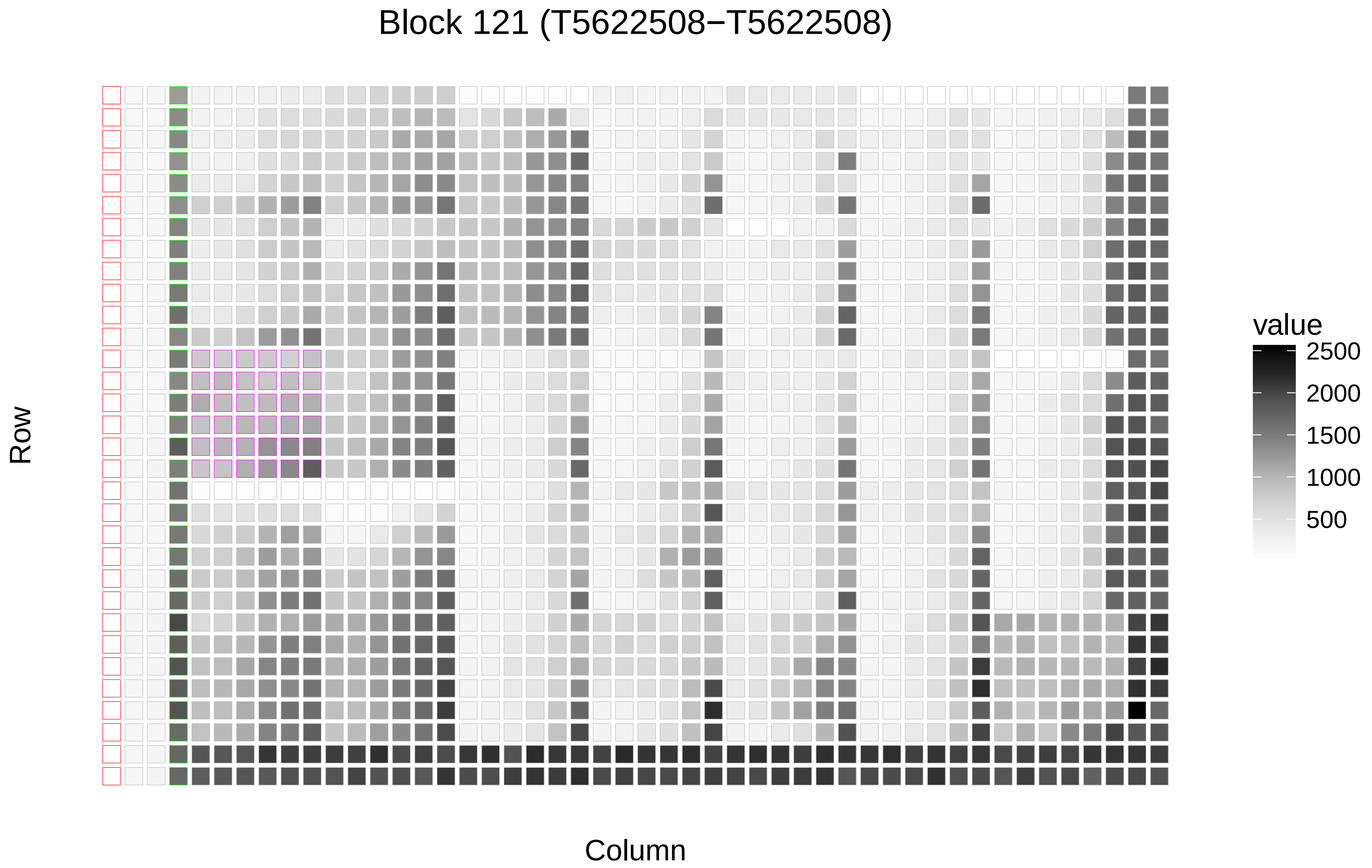

Block 121 (T5622508−T5622508)
Row
Column
value
2500
2000
1500
1000
500

Block 122 (M8900088−T5510634)

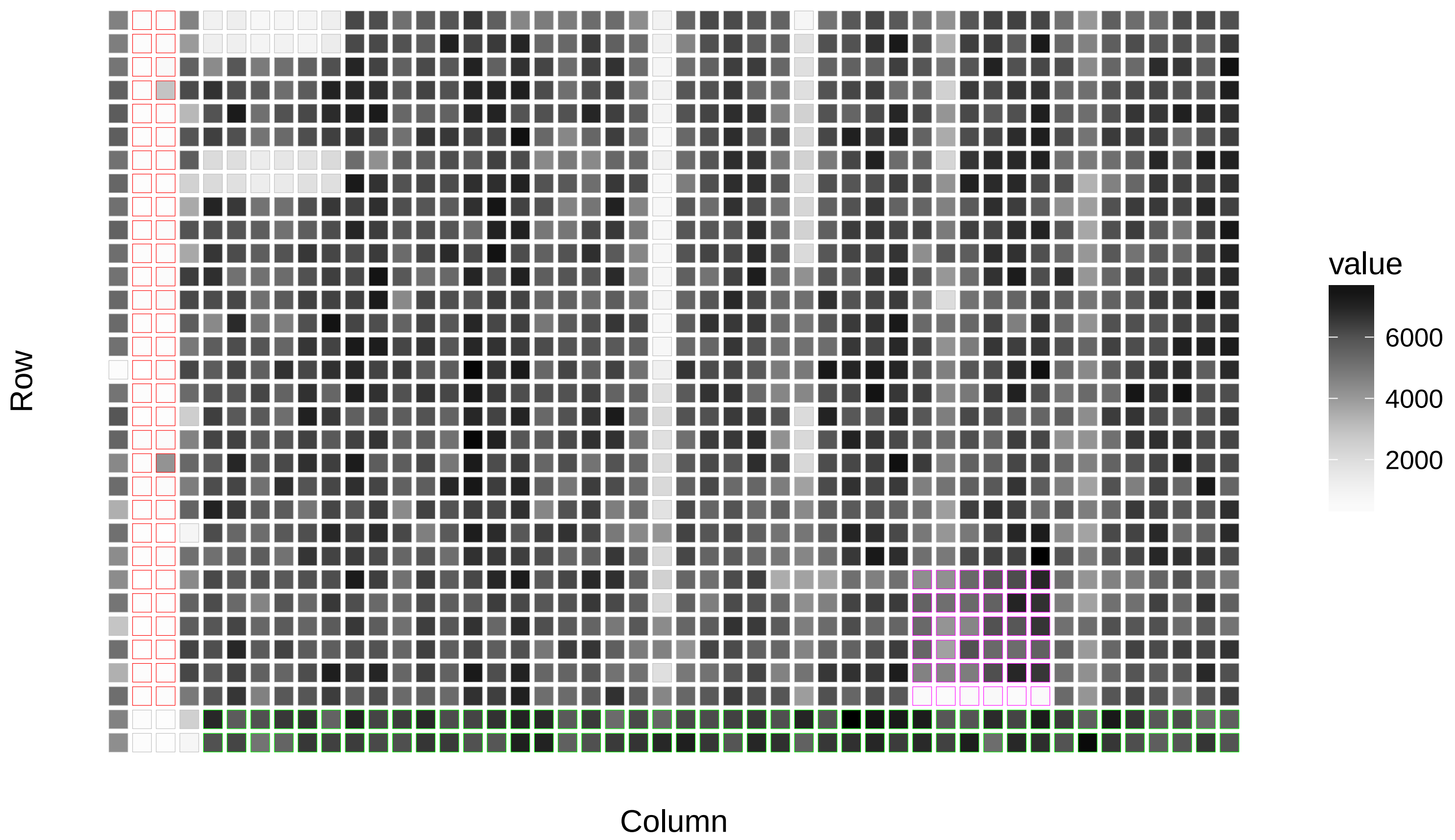

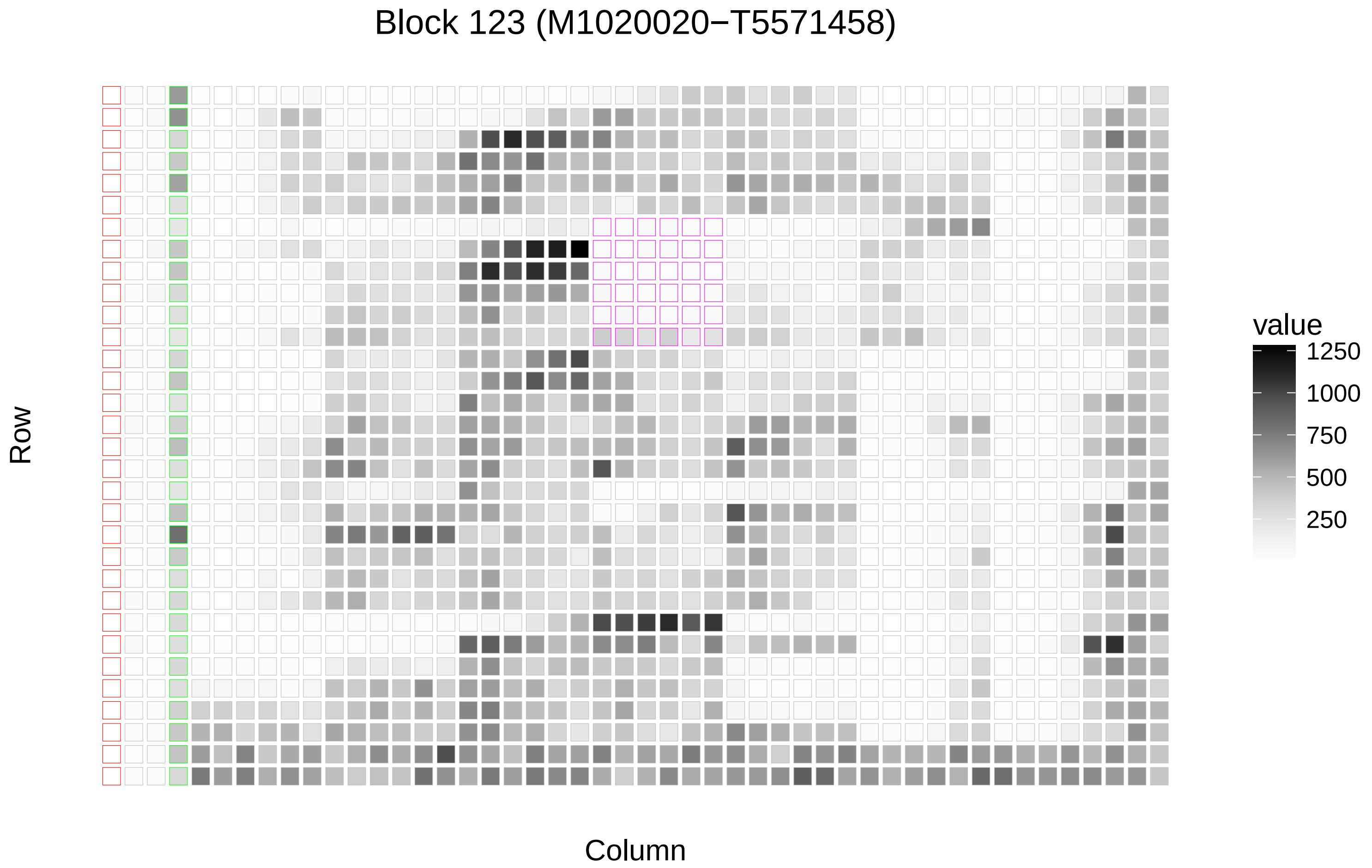

Block 123 (M1020020−T5571458)
Row
Column
value
1250
1000
750
500
250

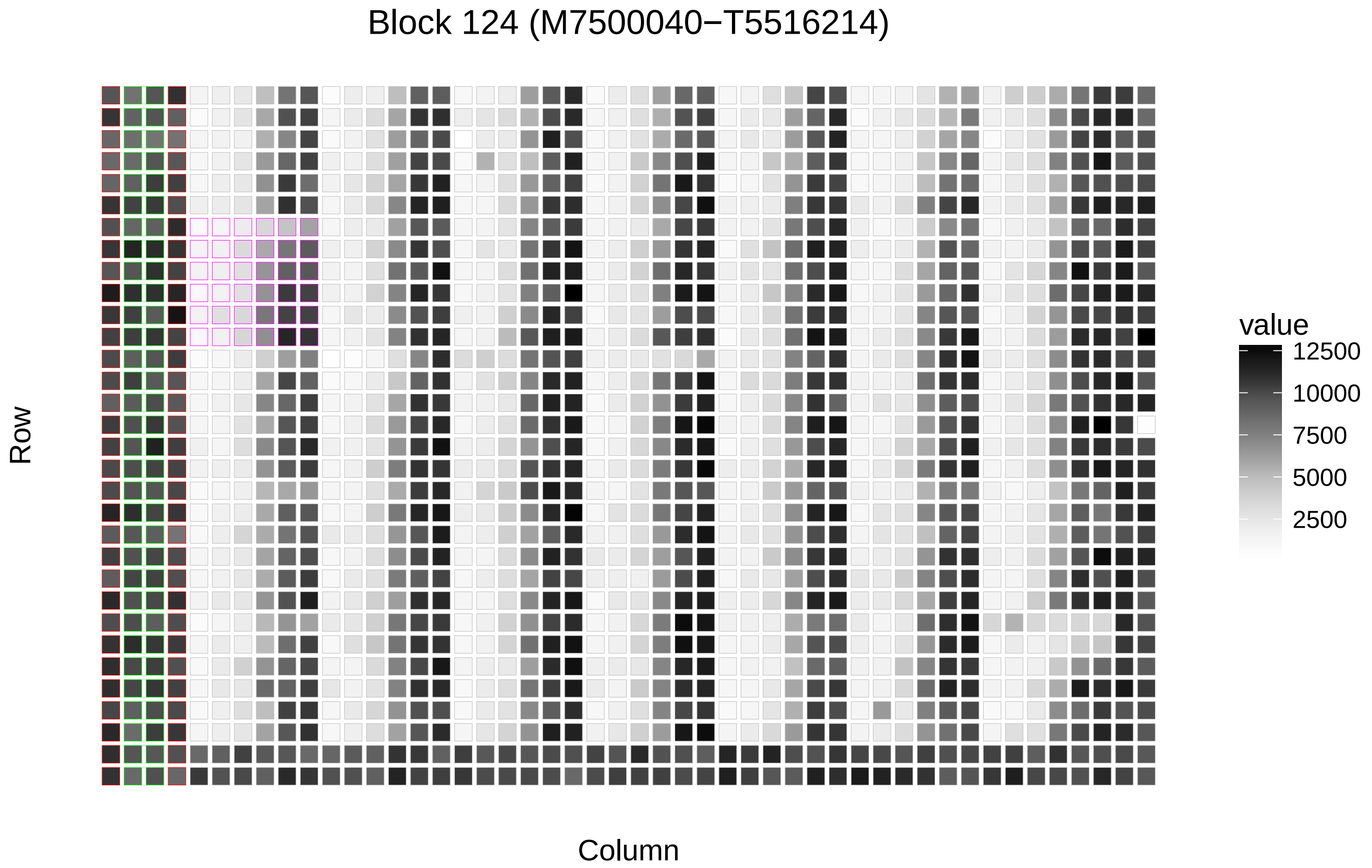

Block 124 (M7500040−T5516214)
Row
Column
value
12500
10000
7500
5000
2500

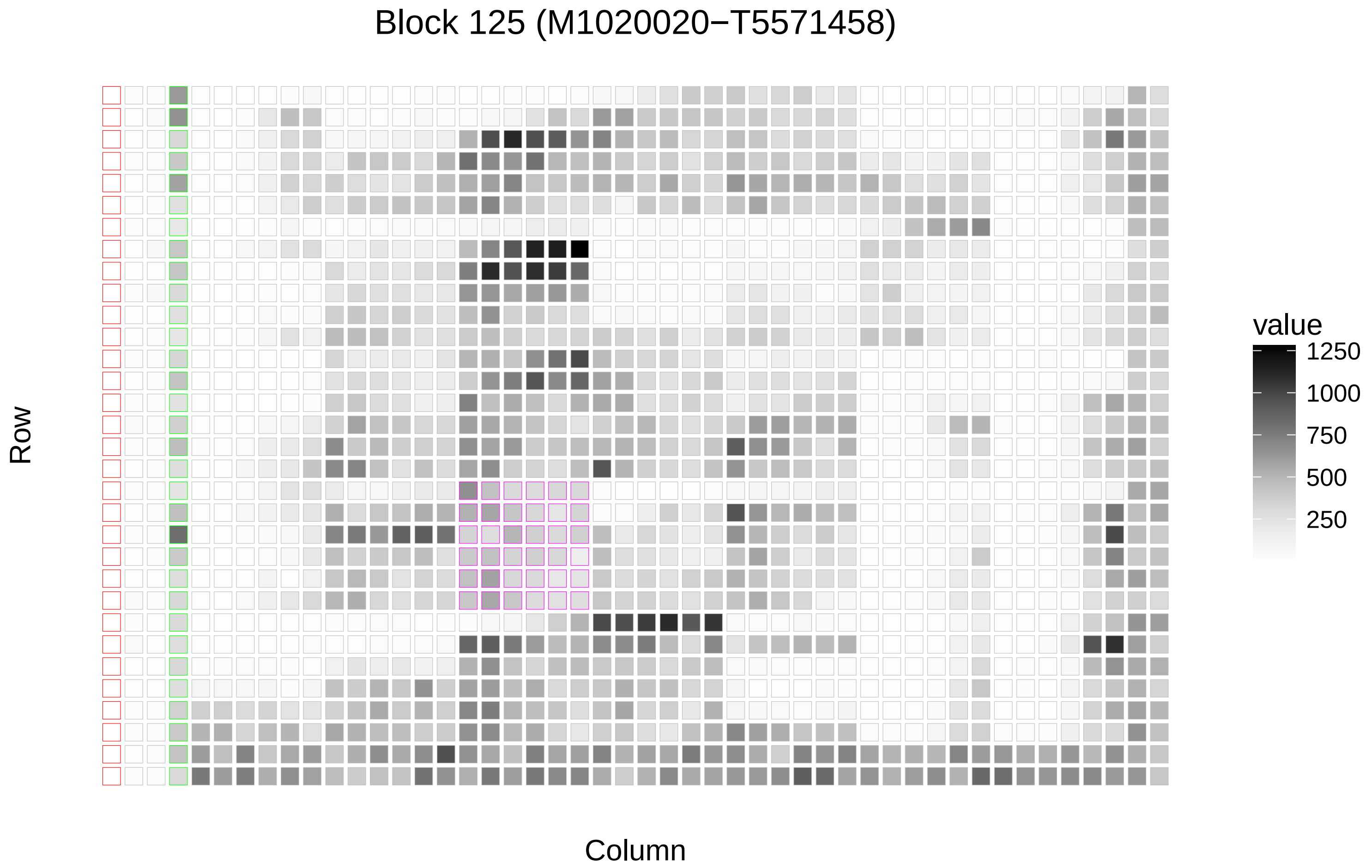

Block 125 (M1020020−T5571458)
Row
Column
value
1250
1000
750
500
250

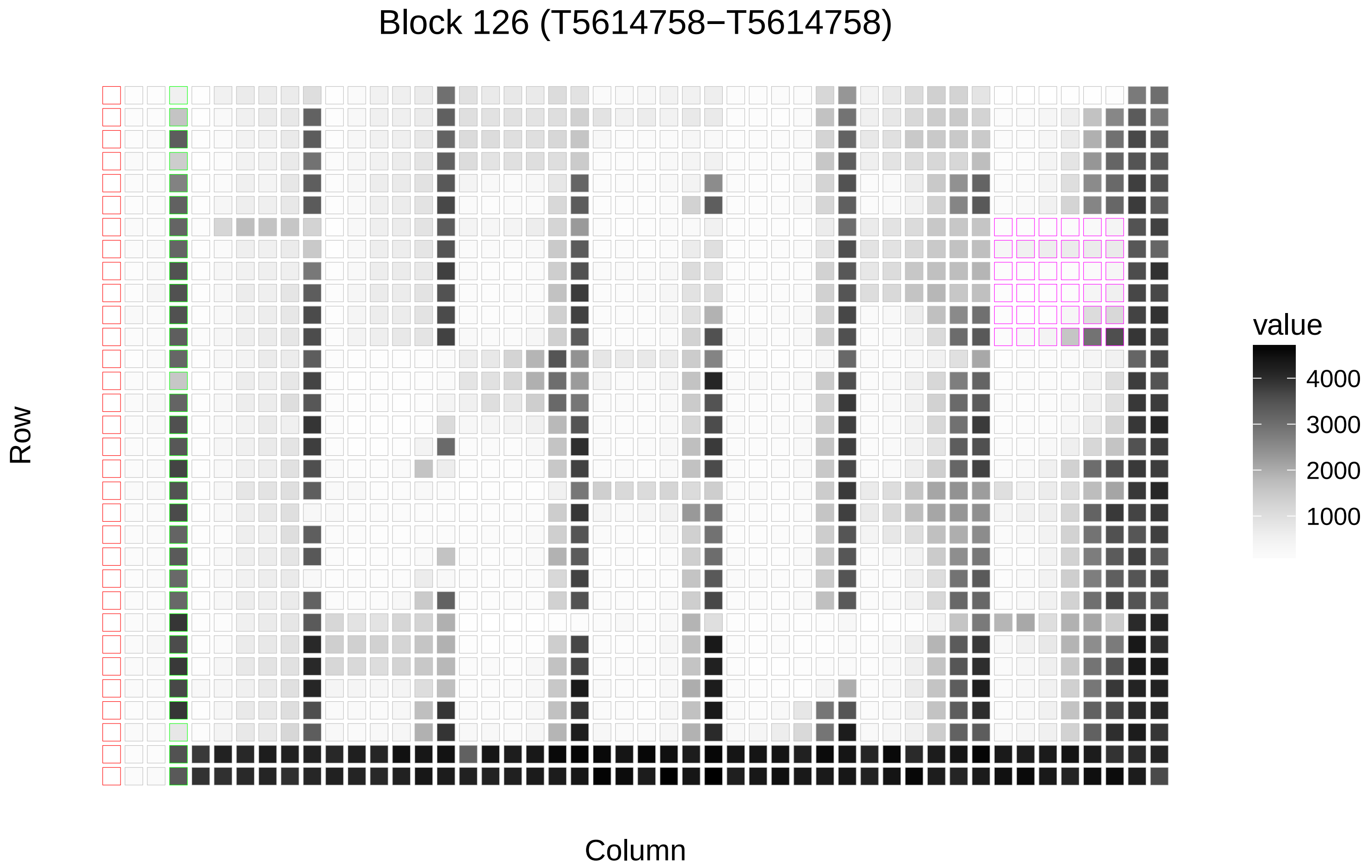
Block 126 (T5614758−T5614758)
Row
Column
value
4000
3000
2000
1000

Block 127 (M0300032−T5423732)

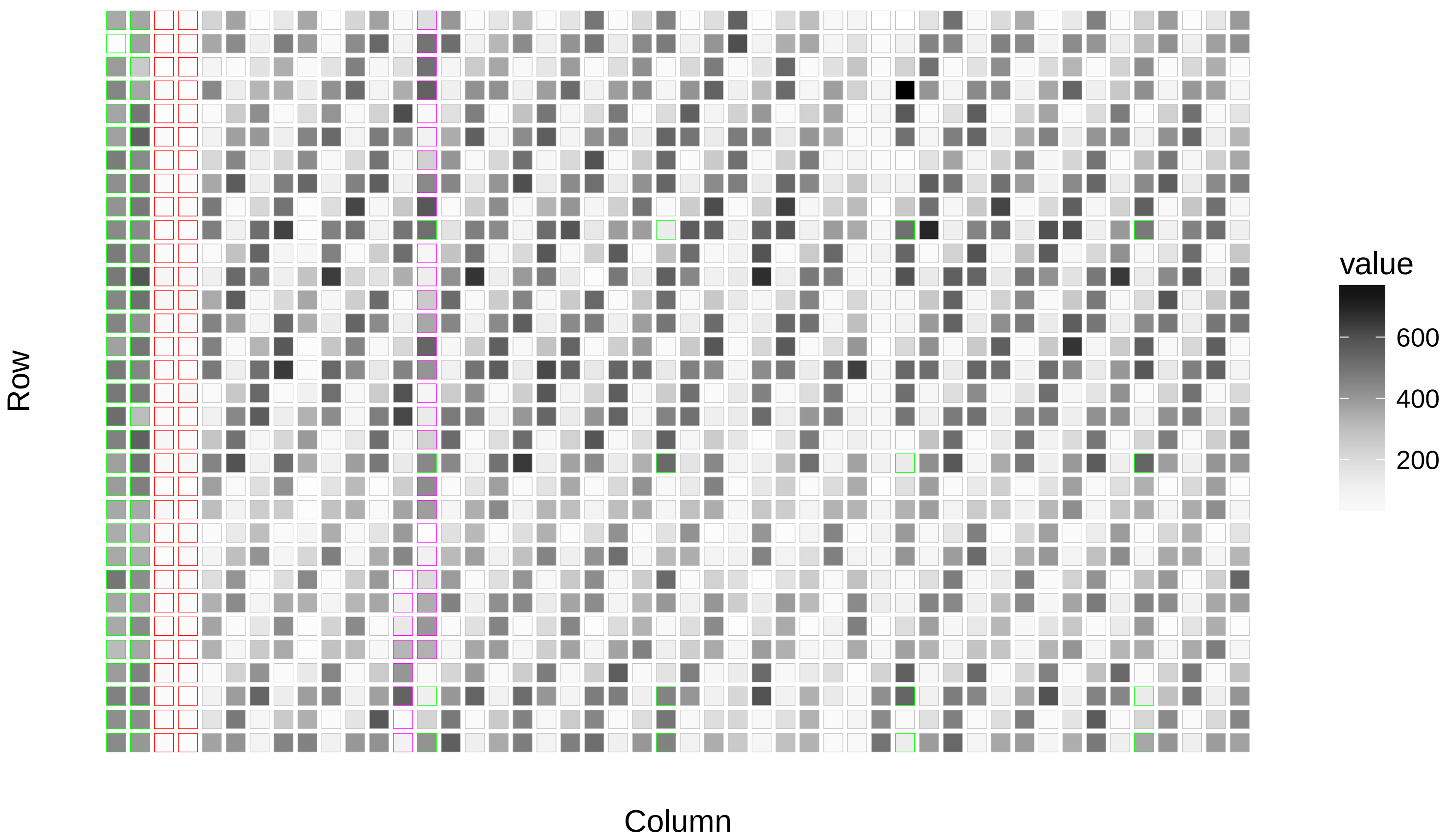

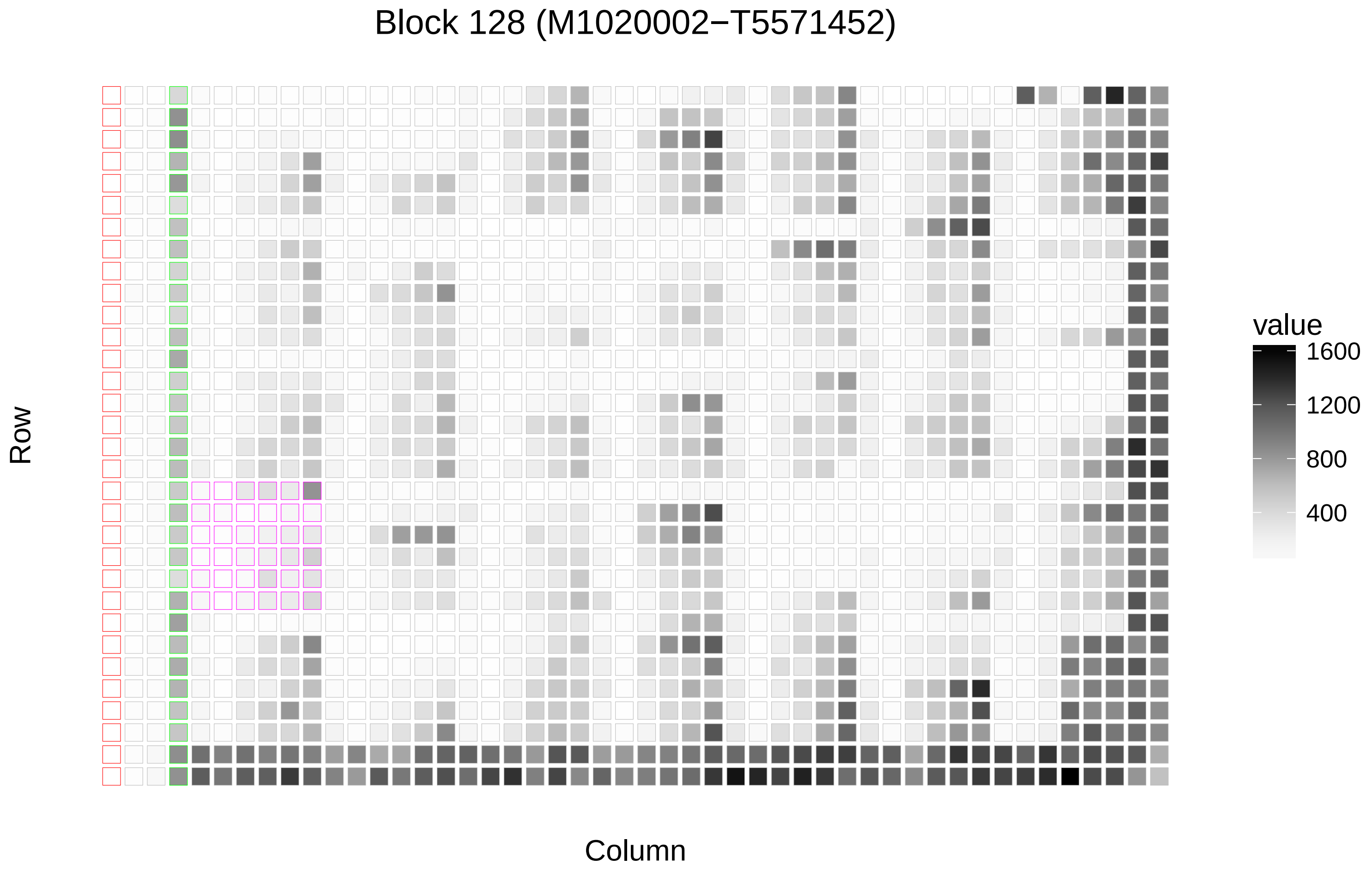
Block 128 (M1020002−T5571452)
Row
Column
value
1600
1200
800
400

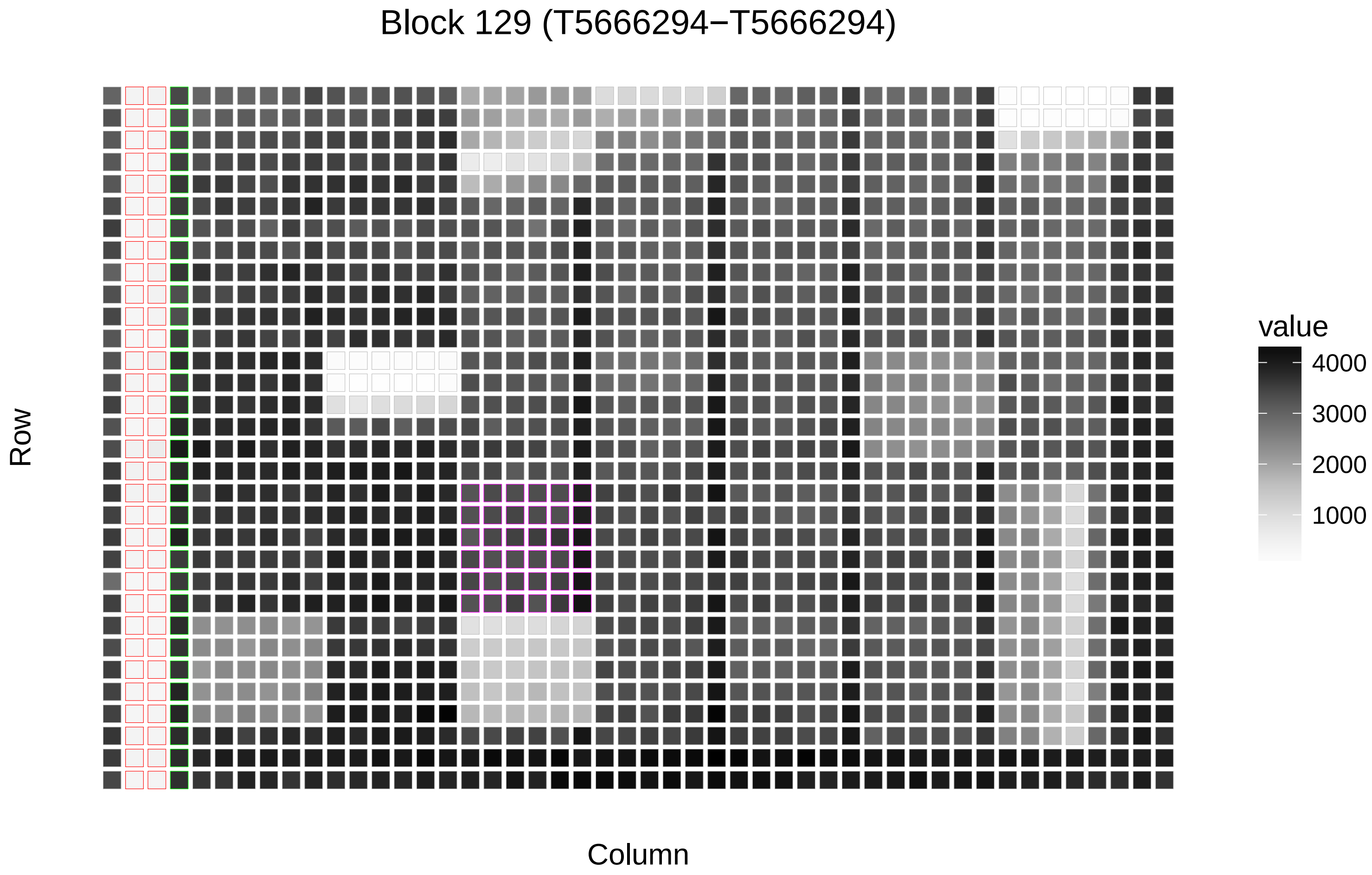
Block 129 (T5666294−T5666294)
Row
Column
value
4000
3000
2000
1000

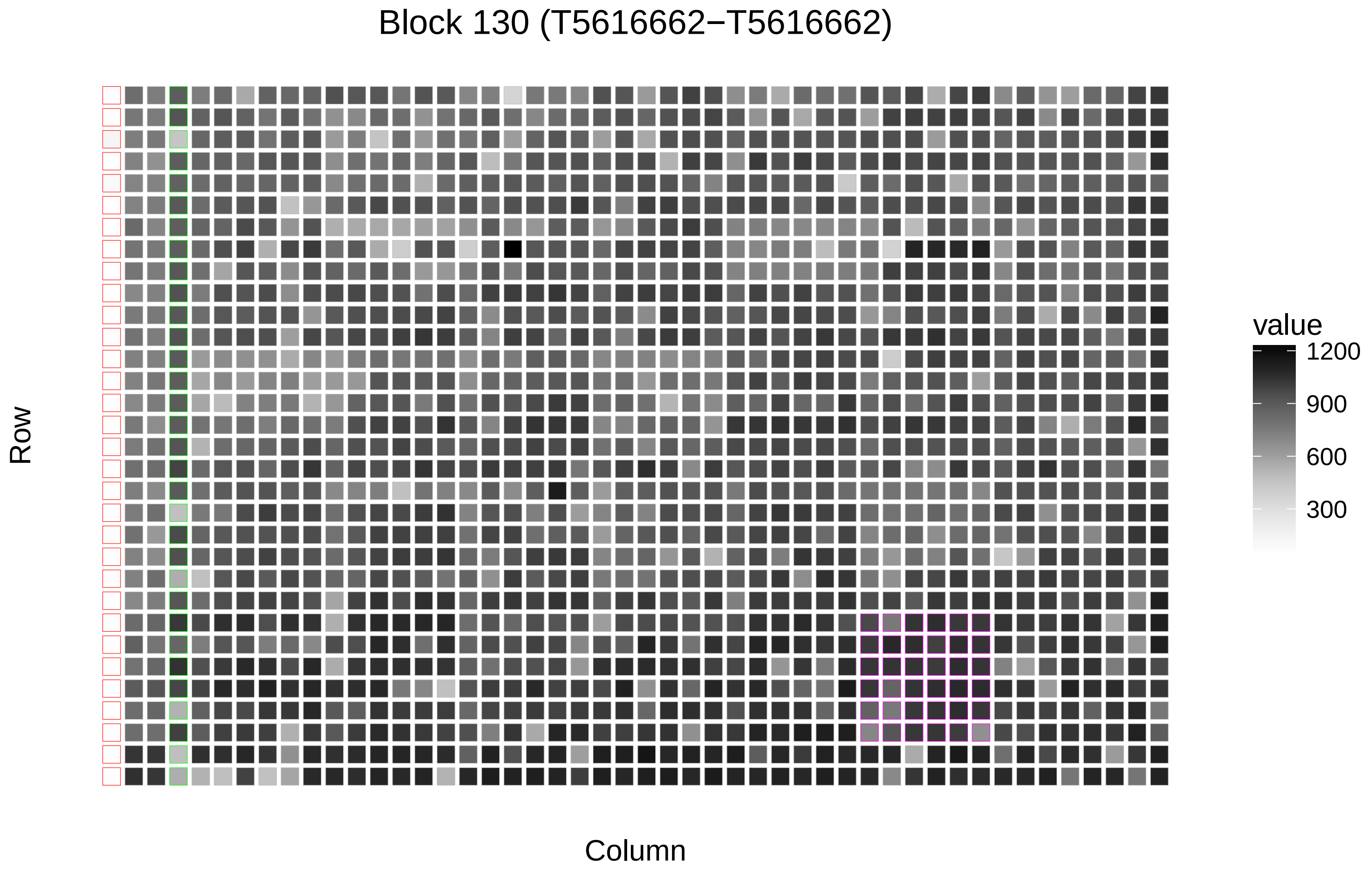
Block 130 (T5616662−T5616662)
Row
Column
value
1200
900
600
300

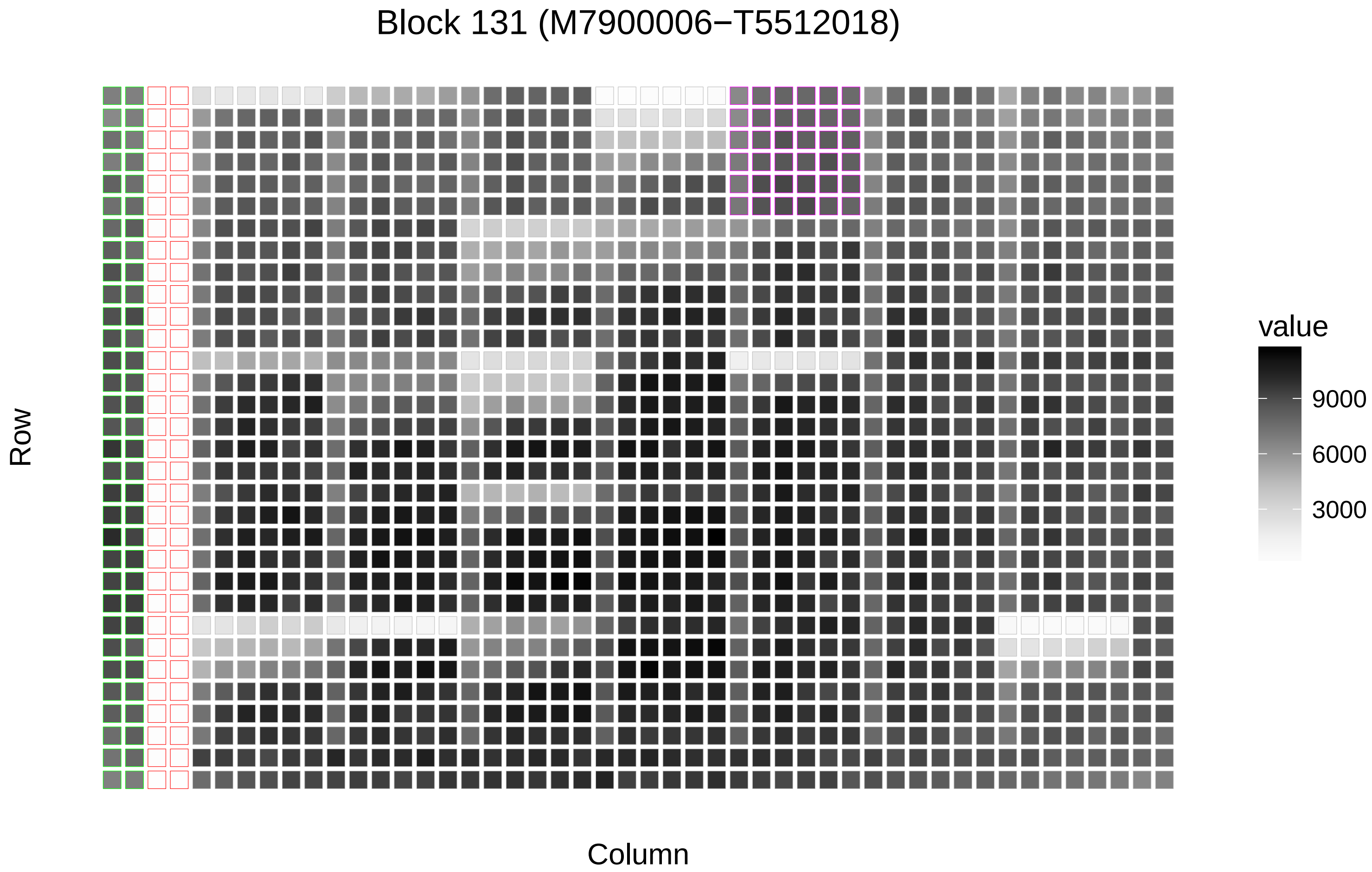
Block 131 (M7900006−T5512018)
Row
Column
value
9000
6000
3000

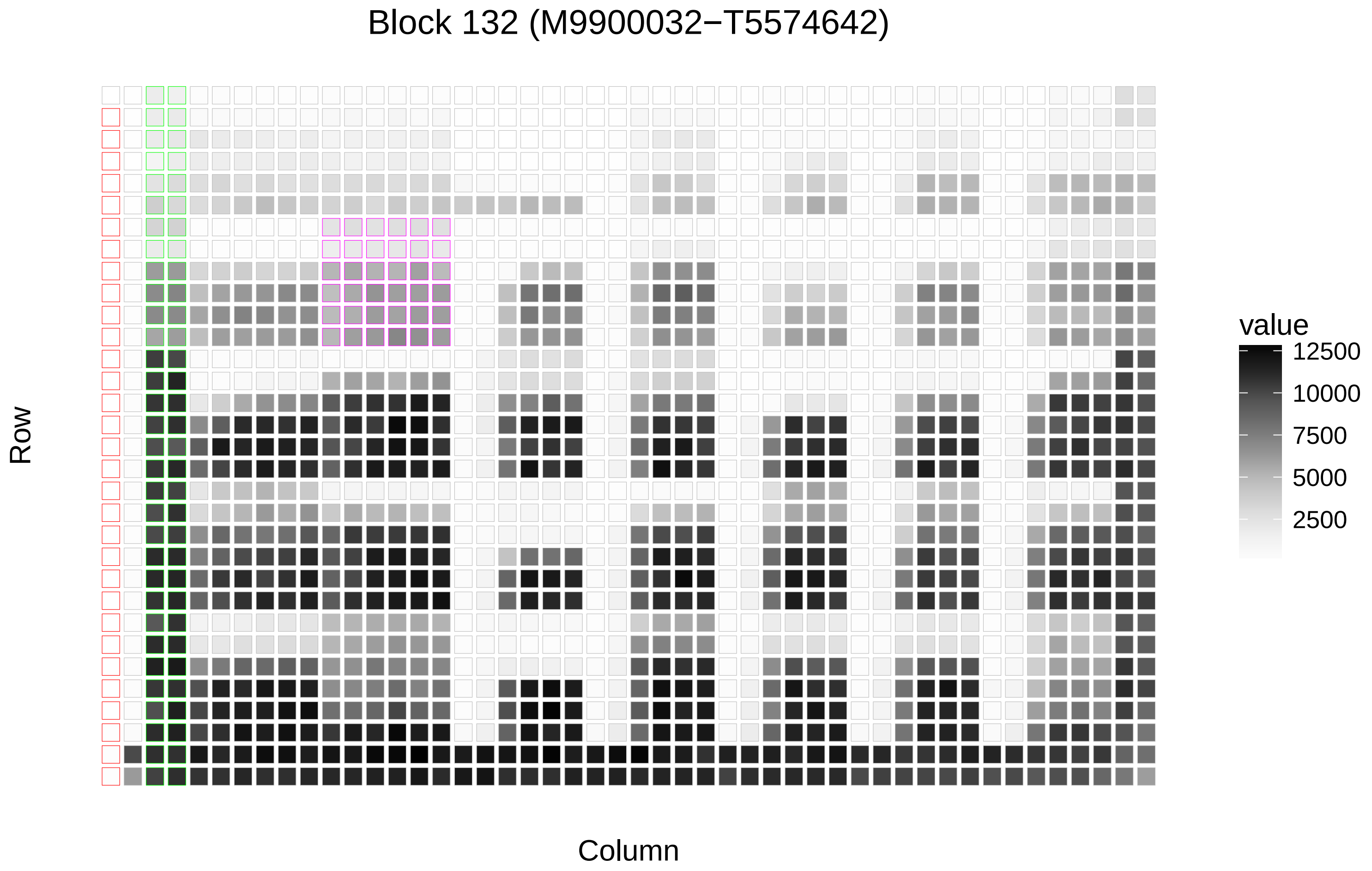
Block 132 (M9900032−T5574642)
Row
Column
value
12500
10000
7500
5000
2500

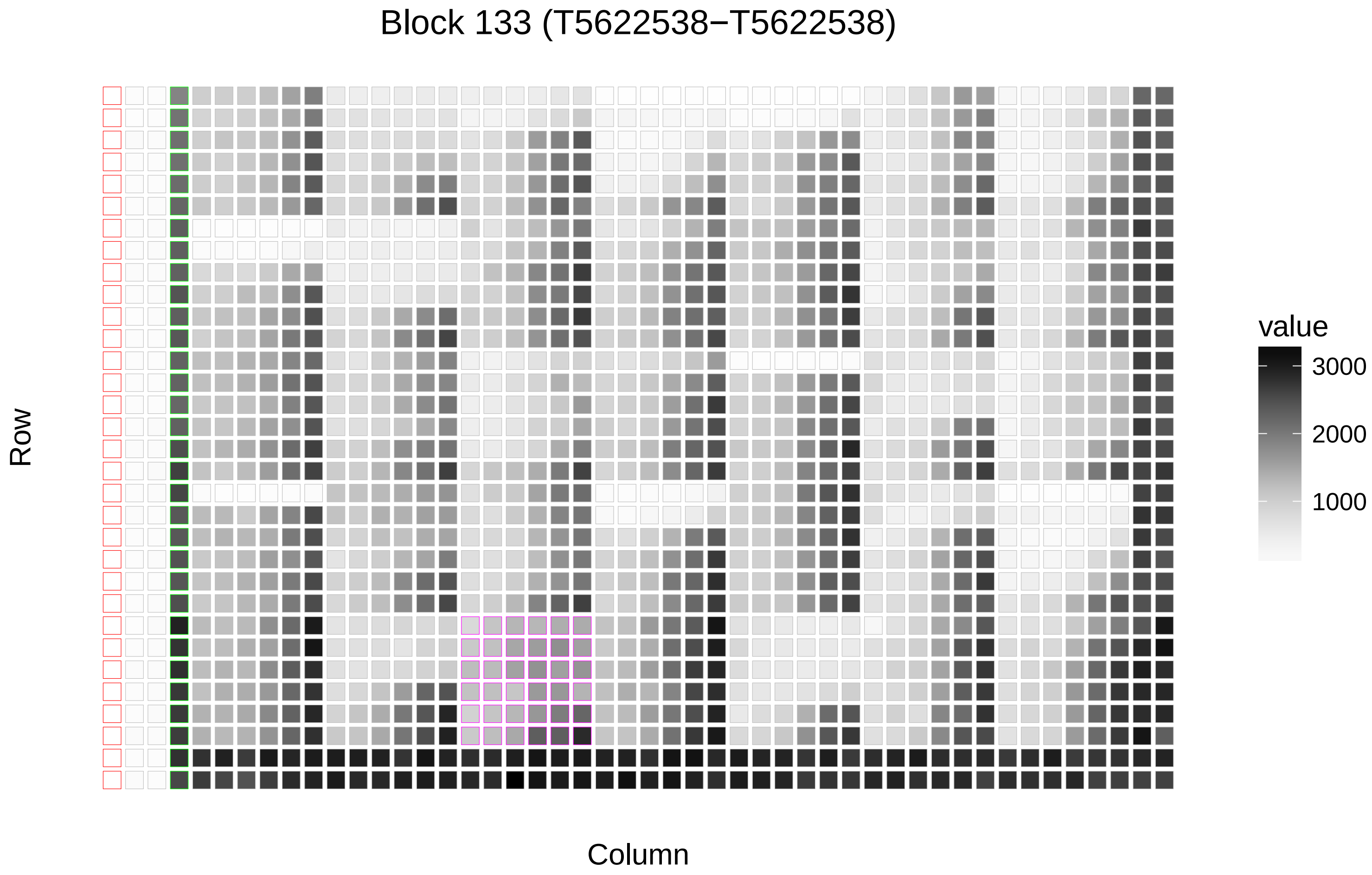
Block 133 (T5622538−T5622538)
Row
Column
value
3000
2000
1000
